# Supplementary material for: ER stress induces caspase‐2‐tBID‐GSDME‐dependent cell death in neurons lytically infected with herpes simplex virus type 2
Source: EMBO J. 2023 Aug 30;42(19):e113118. doi: 10.15252/embj.2022113118 (PMC10548179; doi:10.15252/embj.2022113118)
Supplement: Supplementary file 1 — Appendix [file EMBJ-42-e113118-s006.pdf]

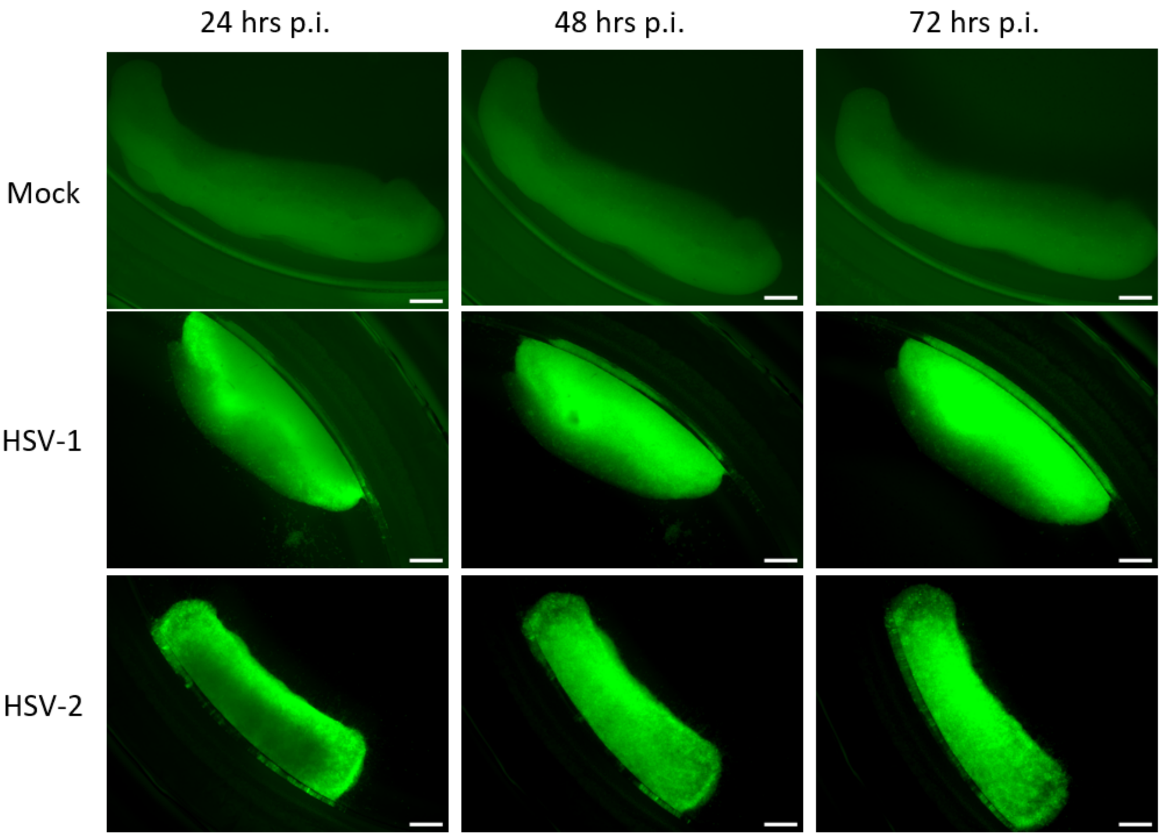

Appendix Figure S1. Replication of HSV-1 and 2 in hfOBSCs
hfOBSC were infected for 72 h with HSV-1 (1×10^6 PFU/ml) and HSV-2 (1×10^6 PFU/ml) expressing green fluoresce protein (GFP). The spread of HSV-1 and HSV-2 infection were initiated from one region of the brain tissue to throughout the whole brain slice. Images of the HSV infected brain slices were monitored with a fluorescent microscope (Zeiss AXIO inverted microscope, 2.5x objective) at indicated time points and reconstituted by using Fiji ImageJ software 1.53t. The data shown are representative for at least 3 independent experiments. Scale bar: 500 µm.

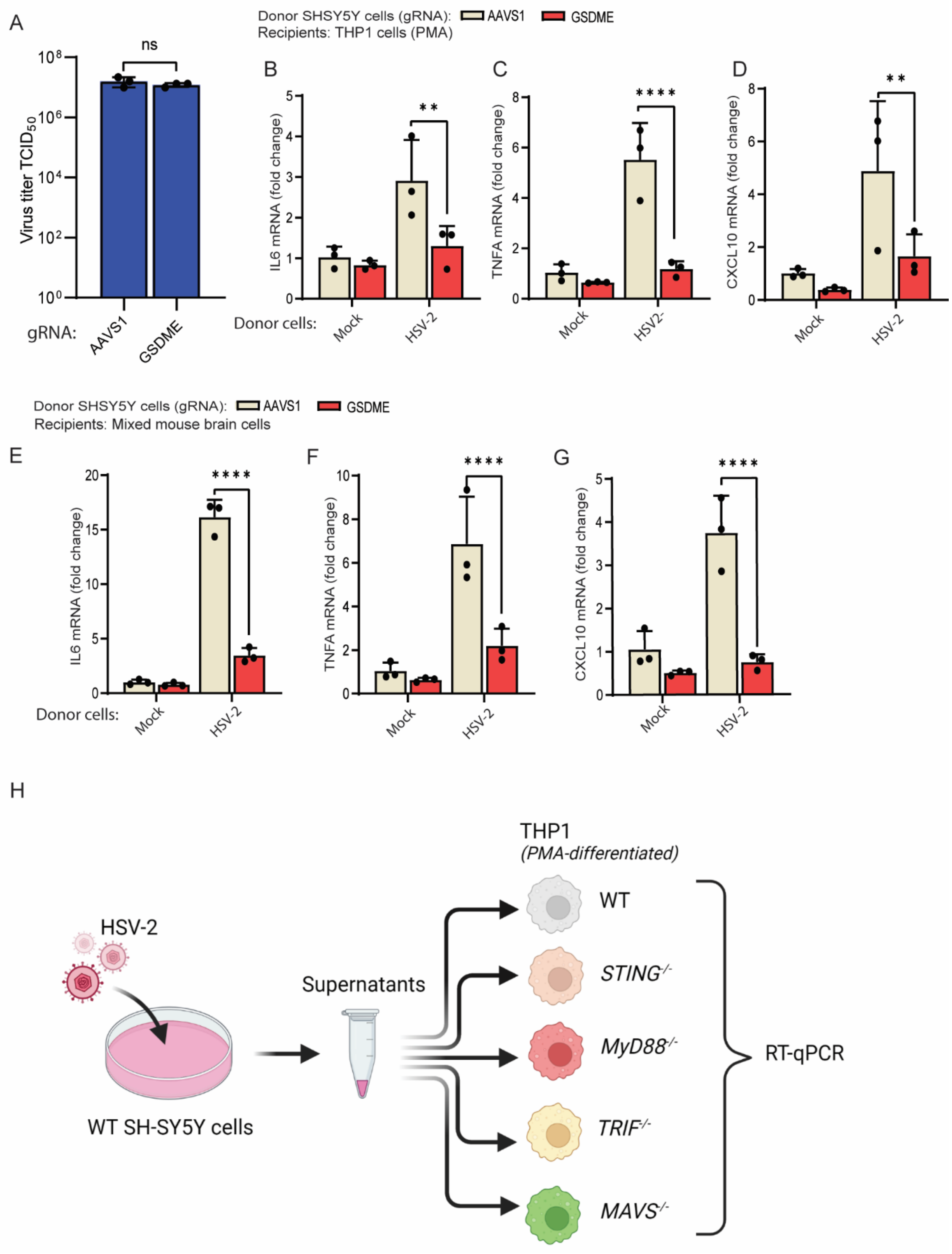

Appendix Figure S2. Necrotic neurons induce inflammatory gene signatures in microglia

(**A**) Supernatants from control and GSDME depleted SH-SY5Y cells infected with HSV-2 (MOI=1) for 24 h infection were subjected to virus titer assay.

(**B-D**) THP1 cells were treated for 8 h with conditioned medium from HSV-2-infected control and GSDME-depleted SH-SY5Y cells. Total RNA was isolated and analyzed for IL6, TNFA, and CXCL10 by RT-qPCR.

(**E-G**) Mixed mouse brain cells were treated with conditioned medium from HSV-2-infected control and GSDME-depleted SH-SY5Y cells for 8 h. Total RNA was isolated and analyzed for expression of Il6, Tnfa, and Cxcl10 by RT-qPCR.

(**H**) Illustration of the experimental setup to evaluate PRR pathways involved in the inflammatory response evoked by pyroptotic HSV-2-infected SH-SY5Y cells. Image was generated by Biorender.

Data information: All data shown are representative of at least 3 independent experiments. Data are presented as mean ± s.d. in all graphs. $*p \leq 0.05$; $**p \leq 0.01$; $****p \leq 0.0001$ (Mann-Whitney test, two-tailed in A, two-way ANOVA in B-G).
