# Supplementary material for: ER stress induces caspase‐2‐tBID‐GSDME‐dependent cell death in neurons lytically infected with herpes simplex virus type 2
Source: EMBO J. 2023 Aug 30;42(19):e113118. doi: 10.15252/embj.2022113118 (PMC10548179; doi:10.15252/embj.2022113118)
Supplement: Supplementary file 2 — Expanded View Figures PDF [file EMBJ-42-e113118-s009.pdf]

Expanded View Figures

Figure EV1. HSV induces GSDME-dependent pyroptotic cell death in neuron-like cells.

A SH-SY5Y cells were infected with HSV-2 (MOI = 1) for 8 and 16 h, and inspected by microscopy for morphological changes. Boxes indicate areas highlighted in the zoomed images. Arrows indicate ballooning or syncytial cells.

B SH-SY5Y cells were treated with UV-inactivated and infectious HSV-2 (MOI = 1, 16 h). Lysates were isolated and immunoblotted for CL-CASP3, GSDME, and VP5 as indicated.

C–E SH-SY5Y cells were pretreated with CHX (10 μg/ml) for 1 h and infected with HSV-2 (MOI = 1). Lysates were isolated and immunoblotted for apoptotic markers, GSDME, and VP5 as indicated, and supernatants were analyzed for LDH release and HMGB1 content.

F–I SH-SY5Y cells were treated with ACV (50 μM) for 1 h and infected with HSV-2 (MOI = 1) for 24 h. Supernatants were analyzed for HSV-2 viral load and LDH release (F, I). Lysates and supernatant from the infected ACV-pretreated SH-SY5Y cells (16 h) were further immunoblotted for CL-CASP3, CL-PARP, GSDME, and HMGB1(G, I).

J–M SH-SY5Y cells pretreated with Z-VAD (20 μM, 1 h) and/or treated with gRNA-Cas9 RNP complexes targeting CASP3 and 7 were infected with HSV-2 (MOI = 1) for 16 and 24 h. Supernatants isolated 24 h post-infection were used to evaluate LDH release (J), and cell lysates and supernatants isolated 16 h post-infection were immunoblotted with the indicated antibodies (K–M). For CRISPR/Cas knockout experiments, a gRNA targeting the safe-harbor locus AAVS1 was included as a negative control.

Data information: All data shown are representative of at least three independent experiments. Data are presented as mean ± s.d. in all graphs. ***$P \leq 0.001$; ****$P \leq 0.0001$ (Mann–Whitney test, two-tailed in F, two-way ANOVA in D, H, and J).
Source data are available online for this figure.

© 2023 The Authors

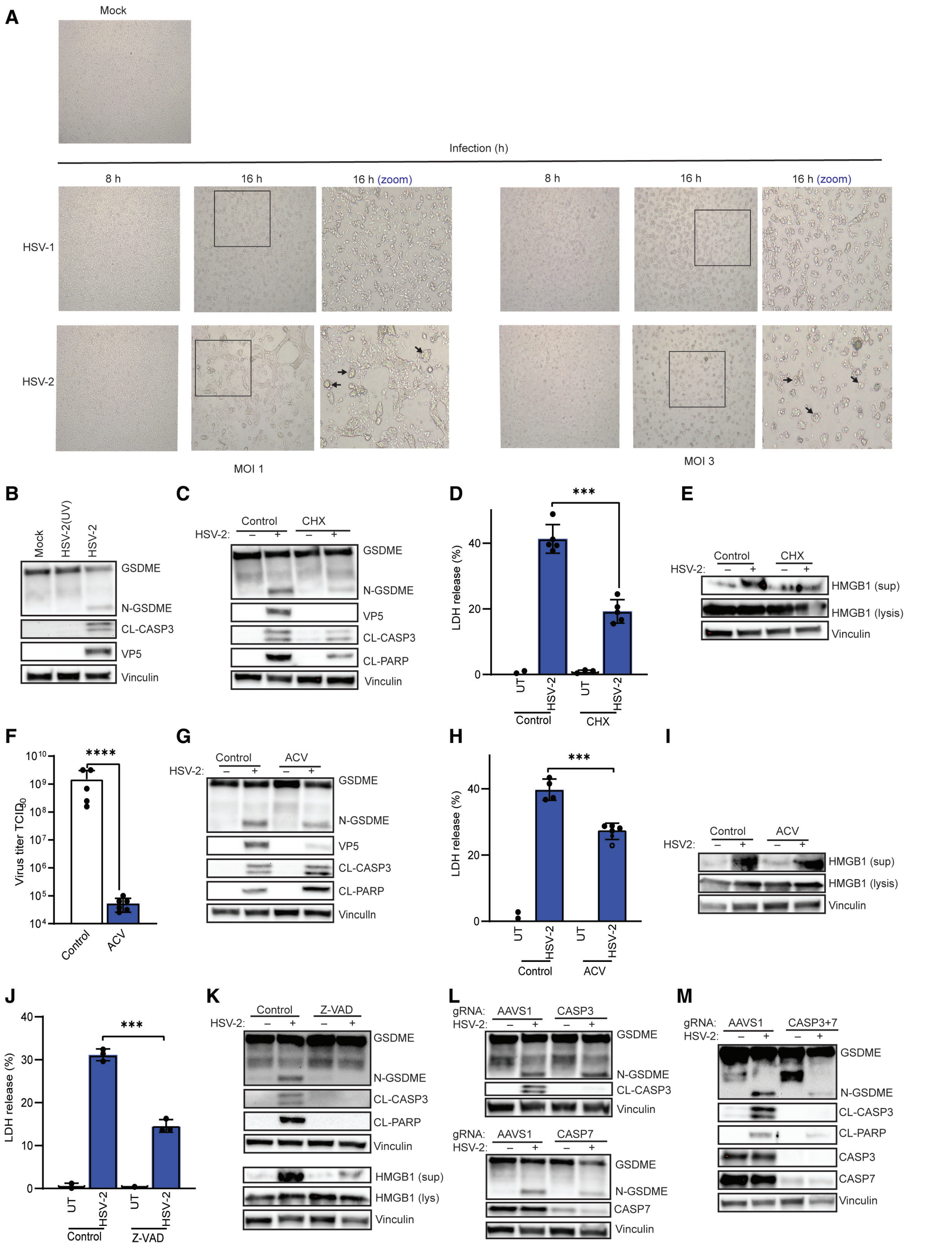

Figure EV1.

© 2023 The Authors

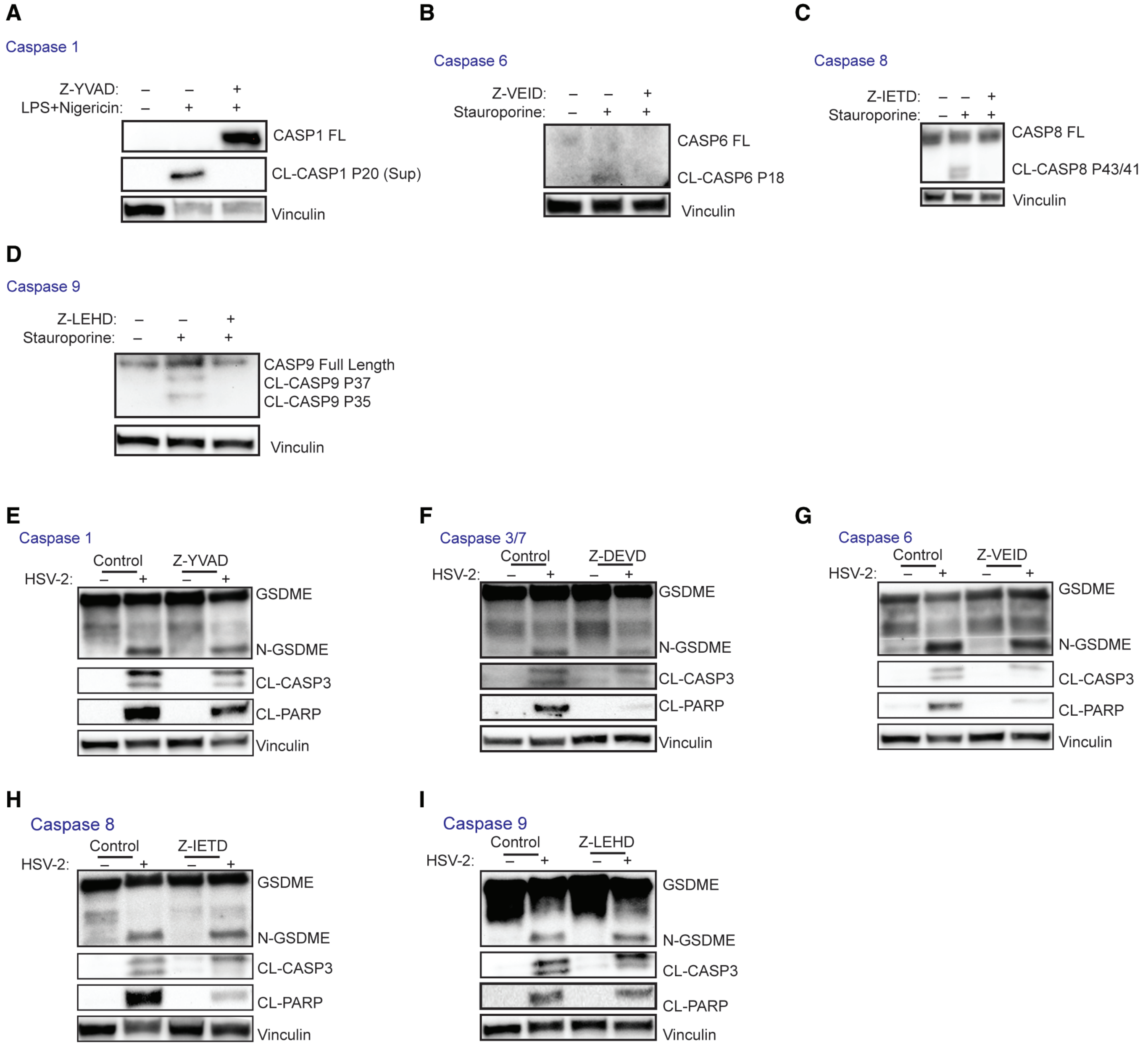

Figure EV2. Effect of inhibition of a panel of caspases on HSV-2-induced cleavage of GSDME.

A–D THP1 cells were pretreated with the indicated caspase inhibitors for 1 h and stimulated with different compounds as positive controls. LPS (1 μg/ml, 4 h) plus nigericin (10 μM, 1 h) were used to induce caspase 1 activity. Staurosporine (1 μM, 6 h) was used to activate caspase 3/6/7/8/9. (A) Z-YVAD, caspase 1 inhibitor; (B) Z-VEID, caspase 6 inhibitor; (C) Z-IETD, caspase 8 inhibitor; (D) Z-LEHD, caspase 9 inhibitor.

E–I SH-SY5Y cells were pretreated with various caspase inhibitors and infected with HSV-2 (MOI =1) for 16 h. Lysate was immunoblotted for CL-CASP3, CL-PARP, and GSDME. (E)Z-YVAD, caspase 1 inhibitor; (F) Z-DEVD, caspase 3/7 inhibitor; (G) Z-VEID, caspase 6 inhibitor; (H) Z-IETD, caspase 8 inhibitor; (I) Z-LEHD, caspase 9 inhibitor.

Source data are available online for this figure.

© 2023 The Authors

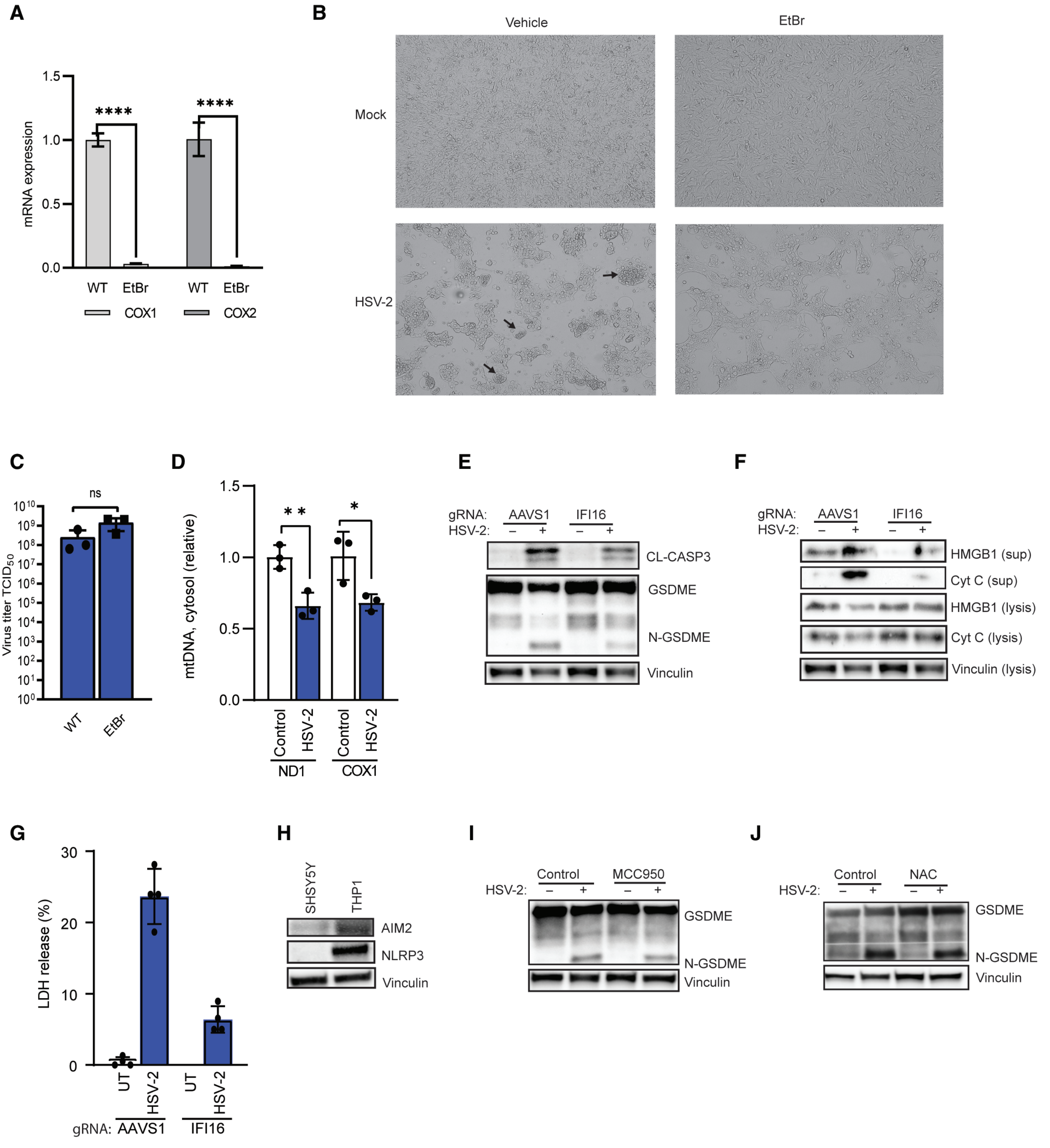

Figure EV3.

© 2023 The Authors

Figure EV3. HSV-induced pyroptosis is dependent on mitochondrial DNA and cytosolic DNA sensing.

A–C SH-SY5Y cells were treated with ethidium bromide treatment (EtBr, 2 μg/ml) for 1 month, and medium was changed every 3 days. mtDNA was quantified by mtDNA by PCR amplification of COX 1 and COX 2 DNA (A). SH-SY5Y cells treated with ethidium bromide treatment were infected with HSV-2 (MOI = 1) for 16 h, and inspected for morphological changes by microscopy. Arrows indicate ballooning or syncytial cells (B). HSV-2 titer in supernatants 24 h post-infection (MOI = 1) (C).

D SH-SY5Y cells were infected with HSV-1 (MOI = 1) for 16 h, and mtDNA release into cytoplasm was quantified by PCR amplification of mtDNA ND1 and COX 1 from DNA isolated from cleared cytosolic fractions.

E–G SH-SY5Y cells treated with AAVS1 or IFI16-targeting gRNA-Cas9 RNP complexes were infected with HSV-2 (MOI = 1) for 16 h and 24 h. Cell lysate and supernatant (16 h with HSV-2 infection) were immunoblotted for GSDME and HMGB1 (E, F), and LDH release assay was performed on supernatants (24 h post-infection, G).

H Lysates from SH-SY5Y cells immunoblotted for AIM2, NLRP3, and vinculin.

I, J SH-SY5Y cells were pretreated with MCC950 or NAC and infected with HSV-2 for 16 h. Lysates were immunoblotted for GSDME and vinculin

Data information: All data shown are representative of at least three independent experiments. Data are presented as mean ± s.d. in all graphs. *$P \le 0.05$; **$P \le 0.01$; ****$P \le 0.0001$ (Mann–Whitney test, two-tailed in A, C, and D, two-way ANOVA in G).
Source data are available online for this figure.

Figure EV4. HSV-2-induced ER stress occurs upstream of pyroptosis.

A SH-SY5Y cells were treated with etoposide (50 μM, 16 h) and thapsigargin (TG, 20 μM) for 16 h. Lysates were immunoblotted for p-eIF2α, tBID, and vinculin.

B SH-SY5Y cells were pretreated with CHX (10 μg/ml) for 1 h and infected with HSV-2 (MOI = 1, 16 h). Lysate was immunoblotted for MCL-1 and vinculin.

C–E (C, E) SH-SY5Y cells were treated with TG (20 μM) and CHX (10 ng/ml) for 24 h. NADPH reductase activity was measured to evaluate mitochondria function. (D) SH-SY5Y transfected with AAVS1 or BID gRNA-Cas9 RNPs were treated with TG (10 μM) for 16 h. Lysates were immunoblotted for GSDME, BID, and vinculin.

F SH-SY5Y cells were infected with HSV-2 in the indicated time intervals or treated with TG (20 μM, 16 h) or CHX (10 ng/ml, 24 h). Coomassie blue staining of SDS–PAGE of protein lysates was used to evaluate protein levels.

G SH-SY5Y cells were pretreated with TUDCA (500 ng/ml) for 1 h and infected with HSV-2 (MOI = 1, 16 h). Lysate was immunoblotted for p-IRE1α, p-JNK, and vinculin.

H SH-SY5Y cells were pretreated with TUDCA (500 ng/ml) for 1 h and infected with HSV-2 (MOI = 1, 16 h). Lysate was assayed for CASP3 activity.

I SH-SY5Y cells were pretreated with TUDCA (500 ng/ml, 1 h) and infected with HSV-2 (MOI = 1) for 16 h, and inspected for morphological changes by microscopy. Arrows indicate ballooning or syncytial cells

Data information: All data shown are representative of at least three independent experiments. Data are presented as mean ± s.d. in all graphs. ****$P \le 0.0001$ (Mann–Whitney test, two-tailed in C and E, two-way ANOVA in H).
Source data are available online for this figure.

© 2023 The Authors

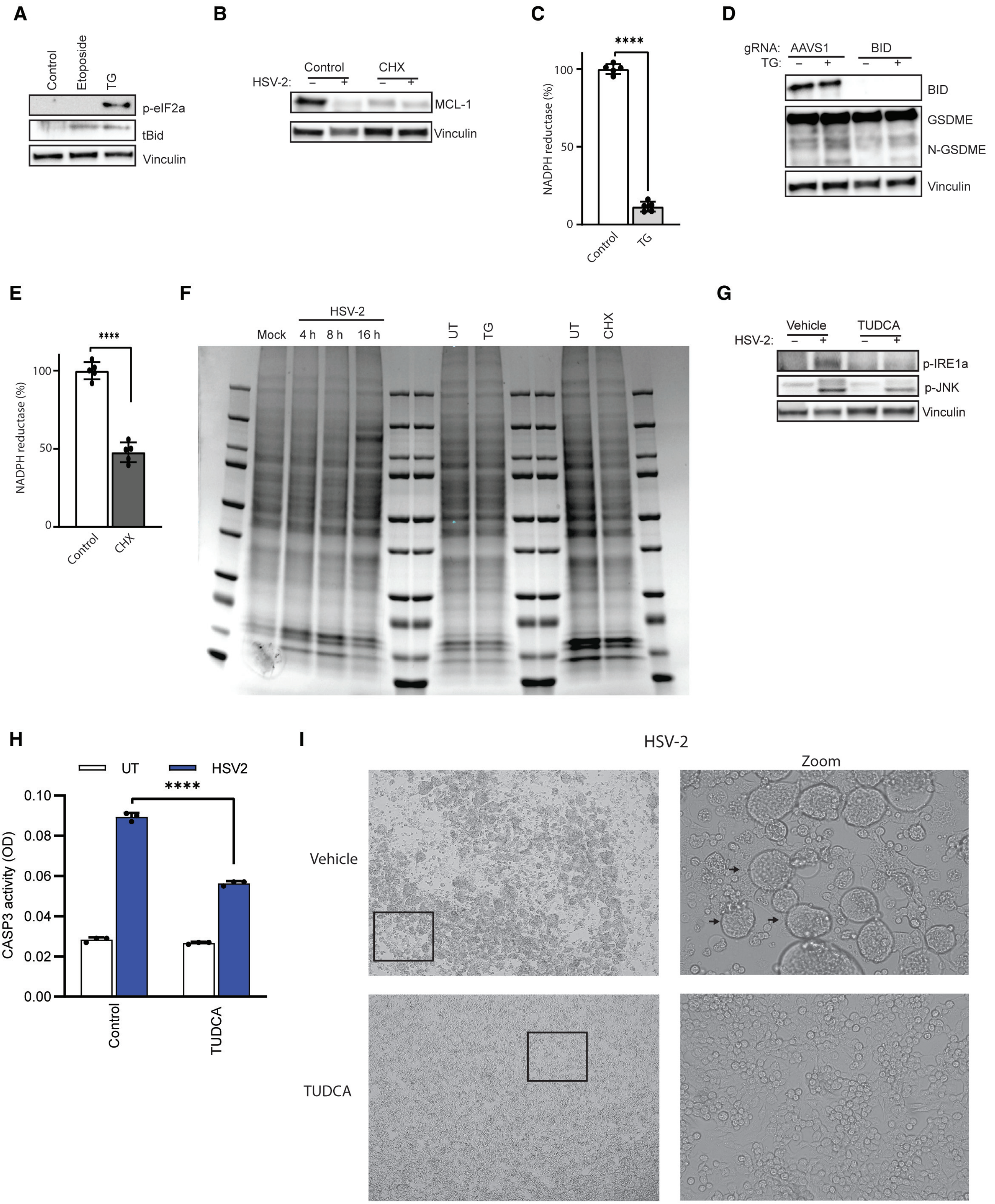
A
Control
Etoposide
TG
p-eIF2a
tBid
Vinculin
B
Control
CHX
HSV-2:
MCL-1
Vinculin
C
****
NADPH reductase (%)
100
50
0
Control
TG
D
gRNA:
AAVS1
BID
TG:
BID
GSDME
N-GSDME
Vinculin
E
****
NADPH reductase (%)
100
50
0
Control
CHX
F
HSV-2
Mock
4 h
8 h
16 h
UT
TG
UT
CHX
G
Vehicle
TUDCA
HSV-2:
p-IRE1a
p-JNK
Vinculin
H
UT
HSV2
****
CASP3 activity (OD)
0.10
0.08
0.06
0.04
0.02
0.00
Control
TUDCA
I
HSV-2
Zoom
Vehicle
TUDCA

Figure EV4.

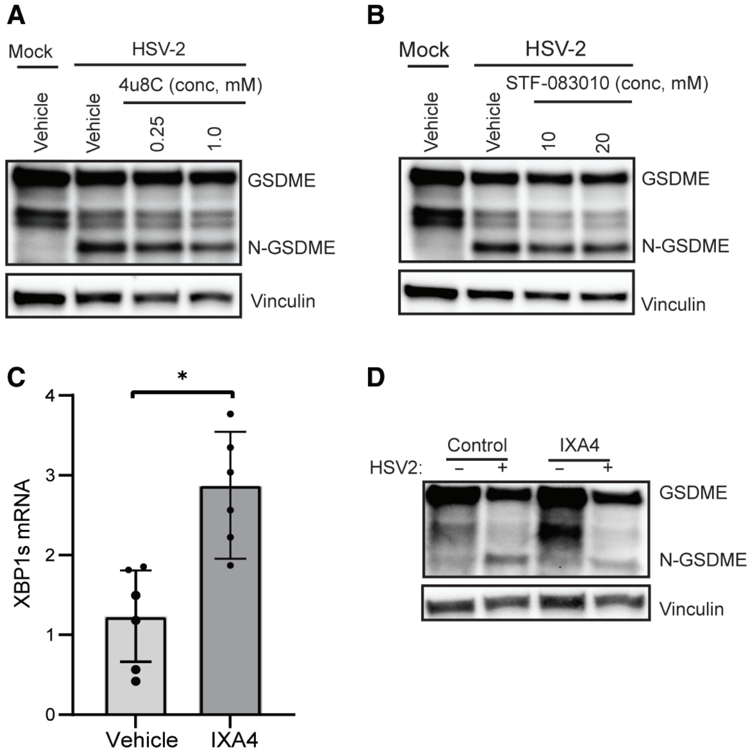

Figure EV5. ER stress-induced IRE1-α activation contributes to HSV-2-induced pyroptosis.

A, B SH-SY5Y cells were pretreated with 4u8c or STF-083010 for 1 h in the indicated concentrations and infected with HSV-2 (16 h). Lysate was immunoblotted for GSDME and vinculin.

C SH-SY5Y cells were pretreated with IXA4 for 1 h and infected with HSV-2 for 16 h, and RNA was isolated to quantify XBP1s mRNA by PCR.

D SH-SY5Y cells were pretreated with IXA4 (10 μM) for 1 h and infected with HSV-2 (MOI = 1, 16 h). Lysates were immunoblotted for GSDME and vinculin.

Data information: All data shown are representative of at least three independent experiments. Data are in (C) presented as mean ± s.d. in all graphs. *$P \le 0.05$ (Mann–Whitney test, two-tailed).
Source data are available online for this figure.

© 2023 The Authors
