# Supplementary material for: ER stress induces caspase‐2‐tBID‐GSDME‐dependent cell death in neurons lytically infected with herpes simplex virus type 2
Source: EMBO J. 2023 Aug 30;42(19):e113118. doi: 10.15252/embj.2022113118 (PMC10548179; doi:10.15252/embj.2022113118)
Supplement: Supplementary file 4 — PDF+ [file EMBJ-42-e113118-s012.pdf]

Article

SOURCE DATA | TRANSPARENT PROCESS | OPEN ACCESS

THE EMBO JOURNAL

ER stress induces caspase-2-tBID-GSDME-dependent cell death in neurons lytically infected with herpes simplex virus type 2

Fanghui Ren[1], Ryo Narita[1], Ahmad S Rashidi[2], Stefanie Fruhwürth[3], Zongliang Gao[1], Rasmus O Bak[1], Martin K Thomsen[1], Georges MGM Verjans[2], Line S Reinert[1] & Søren R Paludan[1,4,*]

Abstract

Neurotropic viruses, including herpes simplex virus (HSV) types 1 and 2, have the capacity to infect neurons and can cause severe diseases. This is associated with neuronal cell death, which may contribute to morbidity or even mortality if the infection is not controlled. However, the mechanistic details of HSV-induced neuronal cell death remain enigmatic. Here, we report that lytic HSV-2 infection of human neuron-like SH-SY5Y cells and primary human and murine brain cells leads to cell death mediated by gasdermin E (GSDME). HSV-2-induced GSDME-mediated cell death occurs downstream of replication-induced endoplasmic reticulum stress driven by inositol-requiring kinase 1α (IRE1α), leading to activation of caspase-2, cleavage of the pro-apoptotic protein BH3-interacting domain death agonist (BID), and mitochondria-dependent activation of caspase-3. Finally, necrotic neurons released alarmins, which activated inflammatory responses in human iPSC-derived microglia. In conclusion, lytic HSV infection in neurons activates an ER stress-driven pathway to execute GSDME-mediated cell death and promote inflammation.

Keywords Herpes simplex virus; IRE1α; neurons; organelle stress; pyroptosis
Subject Categories Autophagy & Cell Death; Microbiology, Virology & Host Pathogen Interaction; Neuroscience
DOI 10.15252/embj.2022113118 | Received 22 November 2022 | Revised 14 August 2023 | Accepted 14 August 2023 | Published online 30 August 2023
The EMBO Journal (2023) 42: e113118

Introduction

A number of viruses can cause infections in the central nervous system (CNS) and lead to diseases, such as meningitis, meningoencephalitis, and encephalitis. This includes herpesviruses, enteroviruses, and arboviruses (Tyler, 2018). For most of these diseases, outcome can be fatal, and risk of severe sequelae is high (Tyler, 2018). Alpha-herpesviruses, including herpes simplex virus (HSV) type 1 and 2, are neurotropic viruses, which have the ability to establish latent infections in dorsal root ganglia in the peripheral nervous system (Bloom, 2016). Although there is a high prevalence of HSV seropositivity in the adult population, the occurrence of HSV-caused diseases in the central nervous system (CNS) is rare. Such diseases include herpes simplex encephalitis (HSE) and recurrent HSV-2 meningitis (Min & Baddley, 2014; Kaewpoowat *et al*, 2016). A central determinant for HSV-induced CNS diseases is lack of immunological control of virus replication in neurons in the CNS. This leads to excessive virus-mediated cytopathic effects in neurons and pathological inflammatory responses.

Histopathologically, a characteristic feature of HSE is necrotizing encephalitis involving the temporal lobe HSE (DeBiasi *et al*, 2002; Gnann & Whitley, 2017). Cortical necrosis has been confirmed in experimental studies in mice, including focal neuronal necrosis and axonal degradation (Armien *et al*, 2010). It has been reported that neuronal cell death during viral infections can be induced directly by viral infections (Girard *et al*, 1999; Nargi-Aizenman & Griffin, 2001; Samuel *et al*, 2007), as well as by excessive inflammatory responses (Ghoshal *et al*, 2007; Lum *et al*, 2017). Such studies have uncovered the double-edge sword-like nature of inflammatory responses in the brain by revealing that microglia can promote potent antiviral activity in neurons (Reinert *et al*, 2016, 2021), but also sensitize them for cell death (Ghoshal *et al*, 2007; Lum *et al*, 2017). Neuronal cell death aggravates the outcome of acute infections (Samuel *et al*, 2007; Orvedahl *et al*, 2010) and impairs long-term recovery (DeBiasi *et al*, 2002; Fricker *et al*, 2018). Therefore, neuronal cell death during viral infections is a central factor in the pathogenesis of viral CNS infections. At this stage, it remains unresolved how HSV induces neuronal cell death.

Cell death can occur in two principal formats, namely apoptosis and necrosis. In the former, the cell shrinks without release of

1 Department of Biomedicine, Aarhus University, Aarhus C, Denmark
2 Department of Viroscience, Erasmus Medical Centre, Rotterdam, The Netherlands
3 Department of Psychiatry and Neurochemistry, Institute of Neuroscience and Physiology, Sahlgrenska Academy at the University of Gothenburg, Gothenburg, Sweden
4 Department of Rheumatology and Inflammation Research, Institute of Medicine, Sahlgrenska Academy, University of Gothenburg, Gothenburg, Sweden
*Corresponding author. Tel: +45 28992066; E-mail: srp@biomed.au.dk

© 2023 The Authors. Published under the terms of the CC BY 4.0 license.

intracellular material and is phagocytosed by macrophages, thus clearing cellular debris and preventing activation of inflammation (Lemke, 2019; Singh *et al*, 2019). In the latter, the plasma membrane is disrupted, and intracellular content is released to promote downstream inflammatory activities (Jorgensen *et al*, 2017). In physiological or biological settings, there is often a combination of the different death processes occurring in parallel, thus giving a more complex picture. For instance, apoptotic responses can be associated with secondary necrosis, if cells are not rapidly cleared (Krysko *et al*, 2006). Both apoptosis and necrosis can be induced through specific molecular mechanisms, and thus termed programmed cell death (PCD). Apoptosis is effectuated by the proteases caspases (CASP) 3 and 7, which can be activated by two main pathways. These are the extrinsic pathway where extracellular ligands engage death receptors to activate CASP8/10 and the intrinsic pathway where mitochondrial depolarization leads to assembly of an apoptosome and activation of CASP9 (Singh *et al*, 2019). Several forms of PCD lead to necrosis, most notably, necroptosis and pyroptosis (Jorgensen *et al*, 2017). In necroptosis, cell lysis is mediated by pores in the plasma membrane formed by phosphorylated MLKL, which is executed by RIPK1 and 3, often as a consequence of CASP8 inhibition (Jorgensen *et al*, 2017). Pyroptosis was first defined as CASP1-dependent cell death and later developed to cover inflammasome- and gasdermin (GSDM)-D-dependent cell death when cleaved GSDMD was discovered to form the CASP1-cleaved pore-mediating cell death (Jorgensen *et al*, 2017). GSDMD is cleaved by activated inflammasomes composed of a pattern recognition receptor, ASC, and one of the CASPs 1, 4/5, or 11 (Kayagaki *et al*, 2015; Shi *et al*, 2015). More recently, additional GSDMs, which can be cleaved by specific proteases to form death-inducing pores, have been discovered (Broz *et al*, 2020; Liu *et al*, 2021). Specifically, GSDME, GSDMA, GSDMB, and GSDMC have been reported to be involved in physiologically important processes, including secondary necroptosis, bacteria-induced necrosis, lymphocyte cytotoxicity, and metabolic priming for cell death, respectively (Rogers *et al*, 2017; Zhou *et al*, 2020; Deng *et al*, 2022). GSDME can be cleaved by CASP3, thus illustrating yet another crosstalk between mechanisms involved in apoptosis and necrosis (Rogers *et al*, 2017; Wang *et al*, 2017). At present, there is no full agreement in the field as to whether the term pyroptosis should define inflammasome-mediated cell death or GSDM-mediated cell death. In the present article, we use pyroptosis to describe the latter.

Alteration of homeostasis leads to cellular stress responses (Chovatiya & Medzhitov, 2014). For instance, accumulation of unfolded proteins, impaired protein translation, hypoxia, and DNA damage activate specific cellular signaling pathways, which seek to reestablish homeostasis through elimination, repair, and adaptation. This is seen during a variety of conditions, including virus infections. For instance, endoplasmic reticulin (ER) stress is a hallmark of many virus infections (Li *et al*, 2015). ER stress occurs when the protein entry into the ER exceeds the capacity of the organelle to support folding, leading to accumulation of incompletely folded proteins (Marciniak *et al*, 2022). This unfolded protein response (UPR) leads to degradation of proteins, inhibition of translation, synthesis of chaperone proteins, and linkage to PCD pathways, including intrinsic apoptosis pathway and pyroptosis (Szegezdi *et al*, 2006; Bronner *et al*, 2015; Han *et al*, 2022; Marciniak *et al*, 2022). However, there is limited information on the role of ER stress, and cellular stress responses in general, in the pathogenesis of viral infections.

HSV-1/2 are a large DNA virus that establishes life-long infections in sensory neurons, and it is therefore no surprise that the viruses encode numerous transcripts and proteins modulating host cells (Paludan *et al*, 2011). This includes a number of mechanisms to circumvent PCD and cell stress (You *et al*, 2017). For instance, HSV-1 counteracts both the intrinsic and the extrinsic apoptosis pathways by mechanisms involving numerous viral gene products, including the infectious cell protein (ICP) 22 and US3 (You *et al*, 2017). HSV-1 also targets necroptosis and GSDMD-mediated pyroptosis through infected cell proteins 6 and 10, and VP22 and ICP0, respectively (Johnson *et al*, 2013; Guo *et al*, 2015; Maruzuru *et al*, 2018). In addition, HSV-1 and 2 evade part of the ER stress response by mediating dephosphorylation of eIF2α through ICP34.5 (Cassady *et al*, 1998; Wylie *et al*, 2009). Thus, all major canonical PCD pathways are modulated by HSV-1 infection, but much less is known about how HSV-2 interferes with cell death and cell stress pathways. Importantly, it remains unknown how lytic HSV infections can cause cell death in neurons.

In this work, we have examined the mechanism through which HSV infection induces cell death in neurons. We report that both mouse and human neuronal cell lines, primary neurons, and human fetal organotypic brain slices (hfOBSC) undergo GSDME-dependent pyroptotic cell death upon infection with HSV-1 and 2. Focusing on HSV-2, we find that this was mediated by infection-driven ER stress signaling through the ER stress sensor IRE1α. The induced programmed cell death pathway involved activation of CASP2, cleavage of the BCL2 family member BID, and permeabilization of the mitochondria, thus triggering CASP3/7-driven GSDME cleavage. Finally, the necrotic neurons release alarmins to activate inflammatory responses in microglia. Thus, excessive lytic viral replication in neurons can lead to both loss of neurons and promotion of neuroinflammation, thereby triggering acute disease and potentially also accelerating development of neurodegenerative diseases.

Results

Lytic HSV infection induces necrotic cell death in human SH-SY5Y neuroblastoma cells

Human SH-SY5Y neuroblastoma cells undergo morphological changes upon HSV infection, including obvious ballooning morphological change in cell death observed at 16 hpi (Fig EV1A). To investigate the release of cytosolic content into the culture supernatant, as markers for cell membrane disruption, we focused on lactate dehydrogenase (LDH) and high mobility group box 1 (HMGB1). Extracellular HMGB1, as an alarmin, aggregates the inflammatory cytokine storm and subsequently a necrotic inflammatory response in neurons (Faraco *et al*, 2007; Kim *et al*, 2018). Importantly, LDH was dramatically increased after infection with both HSV-1 and -2 as well as the two neurotropic RNA viruses, vesicular stomatitis virus and encephalomyocarditis virus (Fig 1A), and kinetics experiments showed that LDH and HMGB1 significantly accumulated in conditioned medium at 16 h post-HSV-2 infection (Fig 1B and C). These data indicate that lytic HSV infection of neuron-like cells induces necrotic cell death. However, human neuroblastoma SH-

 © 2023 The Authors

SY5Y cells did not express GSDMD (Fig 1D), and infection did not induce phosphorylation of MLKL, a necroptosis marker, in SH-SY5Y cells (Fig 1E). Instead, the infection led to cleavage and activation of CASP3, and cleavage of PARP, all markers of apoptosis (Fig 1F and G). Moreover, HSV-2 infection increased cytosolic release of cytochrome c (Fig 1H), an indicator of intrinsic apoptosis induced by mitochondria out membrane permeability (MOMP) dysfunction (Luo *et al*, 1998). As both secondary necrosis (late apoptosis) and pyroptosis contribute to cell membrane disruption and HMGB1 release (Harris *et al*, 2012), we conducted Annexin V/PI staining to

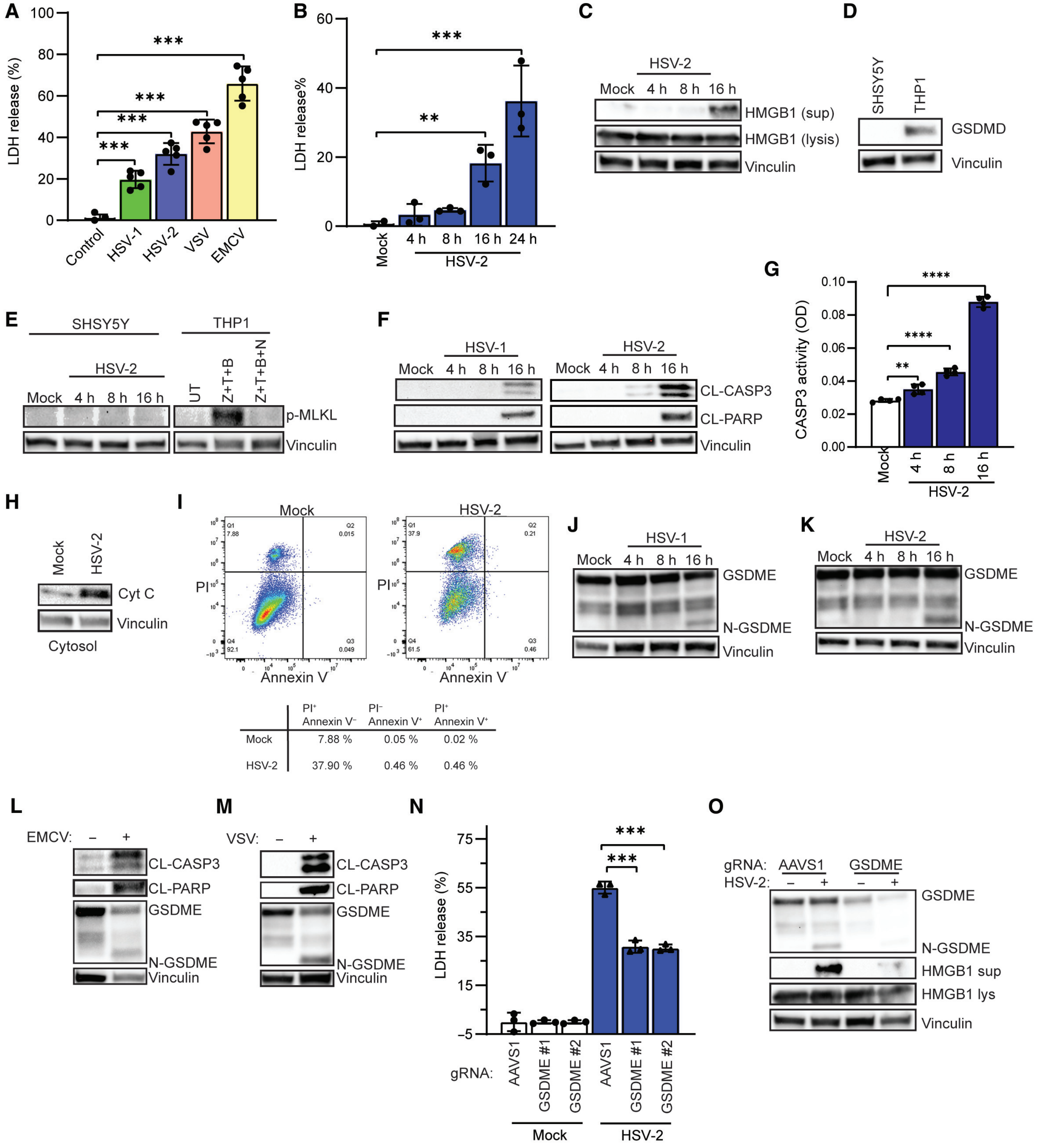

Figure 1.

Figure 1. Lytic HSV infection induces necrotic cell death in human SH-SY5Y neuroblastoma cells.

A SH-SY5Y cells were infected with HSV-1 (MOI = 3), HSV-2, VSV, and EMCV (all MOI = 1) for 24 h, and levels of LDH were measured in the culture supernatants.

B, C SH-SY5Y cells were infected with HSV-2 (MOI, 1), for the indicated time intervals, and culture supernatants were isolated for measurement of LDH (B) or HMBG1 (C).

D Lysates from SH-SY5Y and THP1 cells were immunoblotted for GSDMD and vinculin.

E SH-SY5Y cells were infected with HSV-2 (MOI = 1) for the indicated time intervals, and cell lysates were immunoblotted for p-MLKL and vinculin. For comparison, we used lysates from THP1 cells treated with Z-VAD (Z, 20 μM), TNF-α (T, 100 ng/ml), birinapant (B, 100 nM), and necrostain-1 (NEC-1, 10 μM) as indicated.

F, G SH-SY5Y cells were infected with HSV-1 and HSV-2 (both MOI = 1) for the indicated time intervals. Cell lysates were evaluated for CASP3 activity (G) and immunoblotted for cleaved caspase 3 (CL-CASP3), cleaved PARP (CL-PARP), and vinculin (F).

H, I SH-SY5Y cells were infected with HSV-2 for 16 h, and cytochrome c release from mitochondria into cytoplasm was immunoblotted (H). Annexin V staining and PI uptake were evaluated by flow cytometry (I).

J, K SH-SY5Y cells were infected with HSV-1 (MOI = 3) and HSV-2 (MOI = 1) for the indicated time intervals, and cell lysates were immunoblotted for GSDME and vinculin.

L, M SH-SY5Y cells were infected with EMCV and VSV (both MOI = 1) for 16 h, and cell lysates were immunoblotted for GSDME and vinculin.

N, O SH-SY5Y cells were transfected with GSDME gRNA-Cas9 RNPs (two different gRNAs were used) or AAVS1 gRNA-Cas9 RNPs as negative control and infected with HSV-2. Lysates and supernatants were isolated 16 h post-infection. Lysates were immunoblotted for GSDME, HMGB1, and vinculin. Supernatants were analyzed for LDH release (ELISA) and HMGB1 (immunoblotting).

Data information: All data shown are representative of at least three independent experiments. Data are presented as mean ± s.d. in all graphs. $^{**}P \leq 0.01$; $^{***}P \leq 0.001$; $^{****}P \leq 0.0001$. (Mann–Whitney test, two-tailed in A, B, and G, two-way ANOVA in N).
Source data are available online for this figure.

differentiate late apoptosis (Annexin V positive/PI positive) and pyroptosis (Annexin V negative/PI positive). Surprisingly, HSV-2 infection dramatically induced PI uptake rather than Annexin V binding (Fig 1I), which suggested that gasdermin-dependent pyroptosis could be involved in the HMGB1 release. Interestingly, although GSDMD was not expressed in SH-SY5Y cells (Fig 1D), HSV infections in SH-SY5Y cells induced cleavage of GSDME (Fig 1J and K), correlating with accumulation of HMGB1 in conditioned medium (Fig 1C). This was also observed after infection with EMCV and VSV (Fig 1L and M). Importantly, depletion of GSDME by CRISPR/Cas9 genome-editing showed significantly reduced release of LDH from HSV-2-infected cells (Fig 1N) and largely abrogated release of HGMB1 (Fig 1O). These data demonstrate that infection of human SH-SY5Y neuroblastoma cells with neurotropic viruses induces GSDME cleavage, which mediates pyroptosis and release of alarmins upon HSV-2 infection.

To characterize the mechanism through which HSV-2-induced cell death, the virus was treated with ultraviolet light (UV) before addition to the cells to prevent viral replication while keeping the virions intact. Furthermore, SH-SY5Y cells were also treated with the antiviral drug acyclovir (ACV) or with cycloheximide (CHX), which inhibit viral DNA and protein synthesis prior to infection, respectively. Importantly, both lytic HSV infection and protein synthesis were required for GSDME cleavage in SH-SY5Y cells (Fig EV1B and C). Furthermore, inhibition of *de novo* protein synthesis blocked LDH and HMGB1 release (Fig EV1D and E). Surprisingly, however, ACV treatment, which inhibited HSV-2 replication (Fig EV1F) and LDH release (Fig EV1H), had only a minor effect on GSDME cleavage (Fig EV1G) and release of HMGB1 (Fig EV1I). These data demonstrate that HSV-2 infection in SH-SY5Y cells induces GSDME-dependent programmed cell death through events dependent on a functional viral genome and protein synthesis. Importantly, in order to identify the proteases involved in mediating signaling to GSDME cleavage, we first tested the pan-caspase inhibitor Z-VAD and observed that it inhibited GSDME cleavage and release of LDH and HMGB1 (Fig EV1J and K). CASP3 was previously reported to execute GSDME cleavage (Aizawa *et al*, 2020). In the SH-SY5Y neuroblastoma cells system, CRISPR/Cas9-mediated genome editing of both *CASP3* and *CASP7* was required to abolish GSDME cleavage (Fig EV1L and M). This suggests an essential role for a panel of caspases rather than solely CASP3 in inducing HSV-induced PCD in neurons.

HSV infection of primary human neuronal cells and brain slice cultures induces cleavage of GSDME

To explore whether the observations in SH-SY5Y cells resemble responses in primary human neuronal cells, we first differentiated human embryonal stem cells into neural progenitor cells (NPCs) and infected with HSV-1 and -2 (Fig 2A). Similar to what was observed in SH-SY5Y cells, the NPCs did not express GSDMD but induced significant cleavage of CASP3, PARP, and GSDME following infection (Fig 2B), and this led to necrotic cell death (Fig 2C). To further explore this phenomenon, we generated and cultured hfOBSC and infected these cultures with both HSV-1 and -2 (Fig 2D and E and Appendix Fig S1). In the hfOBSC system, we observed more pronounced infection with HSV-2 than HSV-1 (Fig 2E and Appendix Fig S1), which was associated with cleavage of CASP3, PARP, and GSDME. The PCD response to lytic HSV-1 infection was less evident, despite a similar multiplicity of infection in HSV-2 (Fig 2F). Accordingly, HSV-2 triggered significant LDH release from the human brain slices at 72 h post-infection, which was not seen following HSV-1 infection (Fig 2G). Finally, in mixed mouse brain cells, we observed that HSV-2 infection induced the cleavage of GSDME and release of LDH (Fig 2H–J). Collectively, HSV-2 infection in both human and mouse neuron systems induced GSDME cleavage and subsequent necrotic cell death.

HSV-2-induced pyroptotic cell death in SH-SY5Y cells depends on CASP2

The data shown above suggested that HSV-2 induces pyroptosis in neurons more potently than HSV-1, and we therefore decided to focus the mechanistic work on HSV-2. First, we wanted to explore more broadly the role of caspases in inducing the pyroptotic response, given the pronounced effect of pan-caspase inhibition of HSV-2-induced GSDME cleavage (Fig EV1K). Caspases can be categorized as inflammasome caspases, executioner caspases, and

 © 2023 The Authors

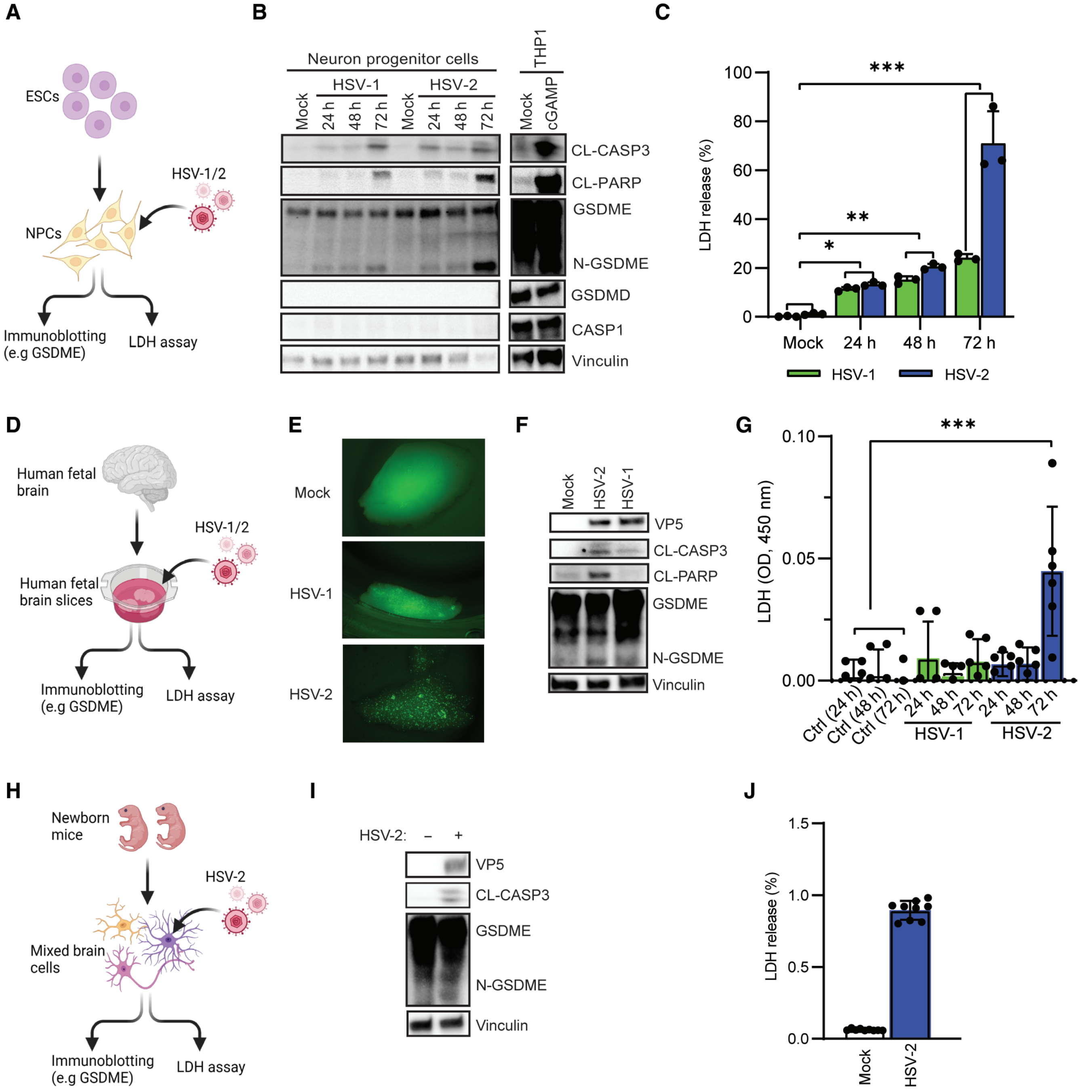

Figure 2. HSV infection of primary human neurons and brain slice cultures induces cleavage of GSDME.

A–C ESC-derived neuron progenitor cells were infected with HSV-1 and HSV-2 (both MOI = 1) for the indicated time intervals, and lysates and supernatants were isolated and analyzed as indicated by (B) immunoblotting and (C) ELISA, respectively.

D–G Human brain slices were infected with HSV-1-GFP (1×10^6 PFU/ml) and HSV-2-GFP (1×10^6 PFU/ml) for 72 h, visualized microscopically, and lysed for analysis of GSDME and vinculin. Levels of LDH in the culture supernatant were evaluated by ELISA, and shown as measured levels of absorbance at 450 nm.

H–J Mouse mixed brain cells were infected with HSV-2 (MOI = 10) for 36 h and lysed for analysis of GSDME and vinculin by immunoblotting. (I). LDH release was measured by ELISA (J).

Data information: All data shown are representative of at least three independent experiments. Data are presented as mean ± s.d. in all graphs. *$P \leq 0.05$; **$P \leq 0.01$ (Mann–Whitney test, two-tailed in C, G, and J). Images shown in panels (A), (D), and (H) were generated in Biorender.
Source data are available online for this figure.

initiator caspases (McIlwain *et al*, 2013). We first immunoblotted for caspase protein levels in SH-SY5Y cells, and monocyte THP1 cells as a positive control. Of the inflammasome caspases CASP1, 4, and 5, only CASP5 was expressed in SH-SY5Y cells (Fig 3A), and pre-treatment with the CASP1 inhibitor Z-YVAD did not affect GSDME cleavage (Fig EV2A and E). The executioner CASP3 and 7 were both expressed at high levels, and pretreatment of CASP3/7 inhibitor Z-DEVD inhibited cleavage of CASP3, PARP, and GSDME (Fig EV2F). These data were consistent with the genome-editing data shown in Fig EV1L and M, showing that CASP3/7 double knockout inhibited GSDME cleavage. The third executioner caspase CASP6 was also expressed in SH-SY5Y cells (Fig 3A), but treatment with the CASP6 inhibitor Z-VEID had no effect on HSV-2-induced GSDME cleavage although some effect was observed in CASP3 and PARP cleavage (Fig EV2B and G). Finally, among the two initiator caspases CASP8 and 9, only CASP9 was expressed, but treatment with inhibitors against either caspase did not affect cleavage of GSDME (Fig EV2C, D, H and I). These data indicate the central role of CASP3 and 7 in HSV-induced GSDME cleavage, but not the other caspases tested.

CASP2 is a caspase that is not easily categorized into the above classes and has been described to be involved in both pore formation-mediated apoptosis and pyroptosis (Kumar, 2009; Imre *et al*, 2012; Bronner *et al*, 2013). In addition, it is reported that the pan-caspase inhibitor Z-VAD does inhibit CASP2 activity in SH-SY5Y cells (Chauvier *et al*, 2007). We first observed that SH-SY5Y cells did express CASP2 (Fig 3A). Treatment with the CASP2 inhibitor Z-VDVAD clearly inhibited HSV-2-induced GSDME cleavage (Fig 3B and Dataset EV1). Notably, depletion of CASP2 expression largely abrogated HSV-2-induced cleavage and activation of CASP3, and cleavage of GSDME (Fig 3C and D). Furthermore, kinetic experiments showed that HSV-2 infection stimulated CASP2 activity in a timely manner (Fig 3E). Finally, depletion of CASP2 reduced LDH release, largely abrogated HMGB1 release, and necrosis-associated morphological changes in HSV-2-infected cells (Fig 3F–H). Collectively, these data suggest that CASP2 is essential for HSV-2-induced GSDME-dependent pyroptosis in human SH-SY5Y neuroblastoma cells.

CASP2 induces BID cleavage to mediate mitochondria-dependent pyroptosis

To examine for possible CASP2 downstream targets involved in GSDME-mediated pyroptosis, we first focused on the reported ability to cleave the protein BID to a truncated pro-apoptotic version (tBID). tBID promotes pore formation in mitochondria via oligomerization of other Bcl-2 family members, especially BAK and BAX (Korsmeyer *et al*, 2000; Wagner *et al*, 2004; Bonzon *et al*, 2006). tBID started to accumulate at 16 h post-infection with HSV-2 (Fig 4A), which correlated with GSDME cleavage (Fig 1K). Furthermore, CRISPR/Cas-mediated disruption of the BID gene inhibited accumulation of cleaved CASP3, PARP, and GSDME and inhibited CASP3 activity, as well as the release of LDH and HMGB1 into the culture medium (Fig 4B–E). Importantly, disruption of the *CASP2* gene blocked accumulation of tBID in HSV-2-infected cells (Fig 4F). These data indicate that BID serves downstream of CASP2 to activate GSDME-mediated pyroptosis. As described above, tBID targets the mitochondria to promote depolarization and the intrinsic apoptosis cell death pathways (Bock & Tait, 2020; Xu *et al*, 2021). Accordingly, we observed that HSV-2 infection strongly impaired mitochondrial function as measured by NADPH reductase activity (Fig 4G).

To further explore the role of mitochondria in mediating GSDME-dependent pyroptosis, we performed mitochondria DNA (mtDNA) deletion with ethidium bromide (EtBr) treatment, which did indeed lead to loss of two mitochondrial genes COX I and COX II (Fig EV3A). As seen in Fig EV3B, mtDNA deletion reduced the degree of the morphological changes under HSV-2 infection, but did not affect virus replication (Fig EV3C). Interestingly, EtBr treatment inhibited GSDME cleavage and HMGB1 release following HSV-2 infection (Fig 4H and I). Recent studies indicated that mtDNA release from mitochondria drives gasdermin-dependent pyroptosis and subsequently inflammatory response (de Torre-Minguela *et al*, 2021; Zhang *et al*, 2021). Hence, we hypothesized that mtDNA release induced by HSV infection contributes to GSDME-regulated pyroptosis. Interestingly, we observed that transfection of synthesis dsDNA and dsRNA into SH-SY5Y cells led to the cleavage of GSDME (Fig 4J), which suggests that mtDNA release may be involved in GSDME cleavage and further pyroptosis. However, we did not observe accumulation of mtDNA in cytoplasm of HSV-2-infected cells (Fig EV3D). In support of a role for mitochondrial nucleic acids in mediating HSV-2-induced pyroptosis, we found that deletion of the DNA-binding protein IFI16 attenuated GSDME cleavage in HSV-2-infected cells and blocked release of cytochrome c, HMGB1, and LDH (Fig EV3E, F, and G). NLRP3, AIM2, and reactive oxygen species (ROS) have been reported to play central roles in mitochondria damage-related cell death (Guo *et al*, 2013; Mangan *et al*, 2018). However, NLRP3 or AIM2 was not expressed in SH-SY5Y cells (Fig EV3H), and pretreatment with inhibitors targeting NLRP3 or ROS did not affect GSDME cleavage (Fig EV3I and J). In summary, CASP2 is essential for cleavage of BID to tBID in HSV-2-infected SH-SY5Y cells to mediate mitochondria-dependent pyroptosis, involving release of nucleic acids into the cytoplasm.

HSV-2-induced endoplasmic reticulum stress occurs upstream of pyroptosis

It has been reported that HSV-1 infection in normal human epidermal keratinocytes cells triggers translational shutdown leading to depletion of BCL-2 family members and mitochondrial depolarization upstream of GSDME (Orzalli *et al*, 2021). ER stress has also been shown to trigger CASP2-tBID-dependent disruption of mitochondrial function (Upton *et al*, 2008; Bronner *et al*, 2015). Among the three main ER stress pathways, that is, IRE1α, PERK, and ATF6, HSV-2 infection led to clear activation of IRF1α (phosphorylation) but not PERK (phosphorylation) or ATF6 (cleavage) (Fig 5A). Despite the lack of detectable phosphorylation of PERK, we did observe phosphorylation of eIF2α, which can be performed by several kinases including PERK, and induction of the down-stream gene C/EBP homologous protein (CHOP) (Fig 5B). However, no CHOP protein was found to accumulate in infected cells, and even ATF6 levels were dramatically decreased (Fig 5A). In agreement with the previous work on HSV-1 infection in human epidermal keratinocytes (Orzalli *et al*, 2021), we observed that HSV-2 infection in SH-SY5Y cells led to depletion of the anti-apoptotic protein MCL-1 and dephosphorylation of the pro-apoptotic protein BAD (Fig 5C).

To test whether ER stress or protein synthesis inhibition induced pyroptosis in SH-SY5Y cells, we treated cells with thapsigargin (TG)

 © 2023 The Authors

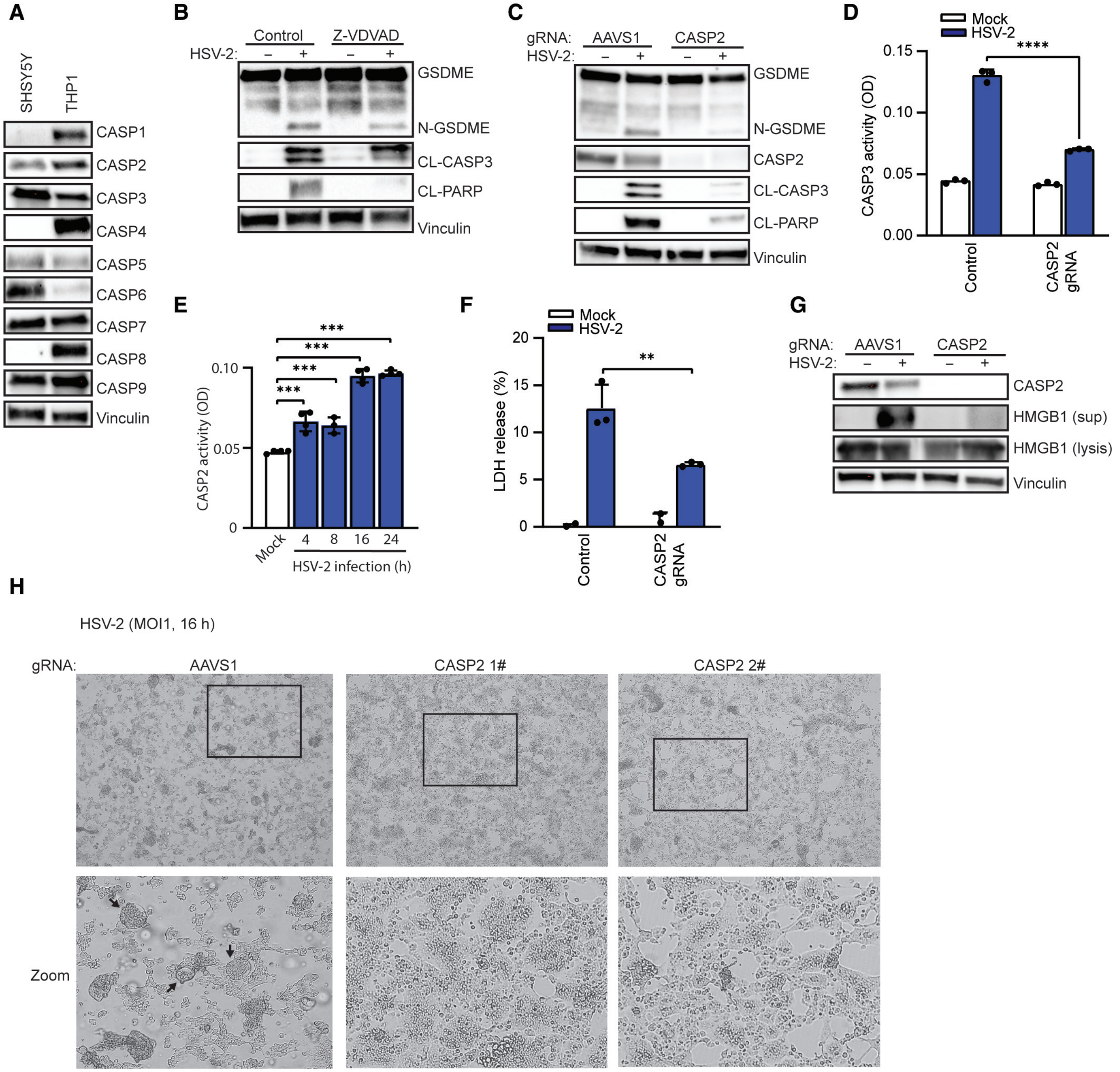

Figure 3. HSV-2-induced pyroptotic cell death in SH-SY5Y cells depends on CASP2.

A Lysates from SH-SY5Y and THP1 cells were immunoblotted for a panel of caspases and vinculin.

B SH-SY5Y cells were pretreated with CASP2 inhibitor Z-VDVAD (20 μM) for 1 h and infected with HSV-2 (MOI = 1). Lysates were isolated 16 hpi for immunoblot analysis.

C, D SH-SY5Y cells were transfected with CASP2 gRNA-Cas9 RNPs or AAVS1 gRNA-Cas9 RNPs and infected with HSV-2. Lysates were isolated 16 h post-infection and assayed for CASP3 activity (D, ELISA) and immunoblotted for GSDME, CASP2, cleaved caspase 3 (CL-CASP3), cleaved PARP (CL-PARP), and vinculin (C).

E SH-SY5Y cells were infected with HSV-2 (MOI = 1) for the indicated time points and lysates were analyzed for CASP2 activity.

F, G SH-SY5Y cells were infected with HSV-2. Supernatant and lysates were isolated for measurement of LDH (F, 22 h) and identification of HMGB1 (G, 16 h).

H SH-SY5Y cells transfected with AAVS1 or CASP2 gRNA-Cas9 RNPs and infected with HSV-2 for 16 h and examined for morphological changes by microscopy. Boxes indicate areas highlighted in the zoomed images. Arrows indicate ballooning or syncytial cells.

Data information: All data shown are representative of at least three independent experiments. Data are presented as mean ± s.d. in all graphs. $^{**}P \leq 0.01$; $^{***}P \leq 0.001$; $^{****}P \leq 0.0001$ (Mann–Whitney test, two-tailed in D, E, and F).
Source data are available online for this figure.

© 2023 The Authors

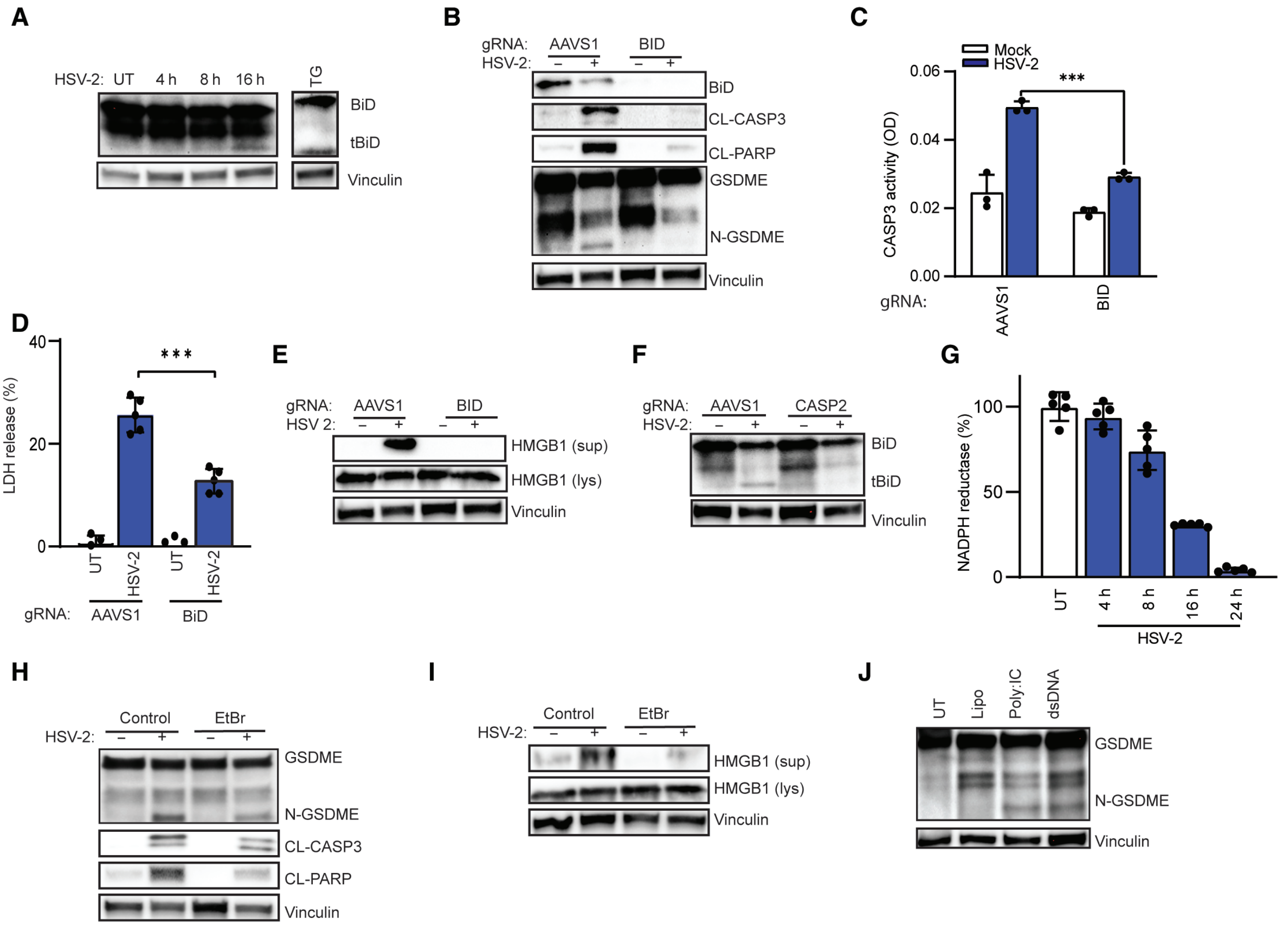

Figure 4. CASP2 induces BID cleavage to mediate mitochondria-dependent pyroptosis.

A SH-SY5Y cells were infected with HSV-2 (MOI = 1) for the indicated time intervals or stimulated with TG (20 μM, 16 h). Lysates were immunoblotted for BID and vinculin. tBID, truncated BID.

B, C SH-SY5Y cells transfected with AAVS1 or BID gRNA-Cas9 RNPs were infected with HSV-2. Lysates were isolated 16 h post-infection and immunoblotted for GSDME, CL-CASP3, and CL-PARP (B), and assayed for CASP3 activity (C).

D, E SH-SY5Y control and BID-depleted cells were infected with HSV-2 (MOI = 1). Supernatant and lysates were evaluated for LDH (24 h) and HMGB1 (16 h) release. Vinculin blot panel E is identical to the one in panel B.

F SH-SY5Y control and CASP2-depleted cells were infected with HSV-2 (MOI = 1, 16 h) and immunoblotted for truncated BID, tBID.

G NADPH reductase activity was measured to evaluate mitochondria function in SH-SY5Y cells infected with HSV-2 (MOI, 1) for the indicated time intervals.

H–J SH-SY5Y cells were treated with ethidium bromide (2 μg/ml) and infected with HSV-2 (MOI = 1, 16 h). Lysate and supernatant were for immunoblotting for cell death markers GSDME and HMGB1 (H, I). SH-SY5Y cells were transfected with Poly I:C (50 μg/ml) or dsDNA (20 μg/ml) for 24 h (J).

Data information: All data shown are representative of at least three independent experiments. Data are presented as mean ± s.d. in all graphs. ***$P \leq 0.001$ (Mann–Whitney test, two-tailed in G, two-way ANOVA in C and D).

Source data are available online for this figure.

or CHX. TG, an ER stress inducer, has been found to induce CASP2/3/7-dependent cell death in SH-SY5Y cells (Dahmer, 2005). Treatment with TG leads to ER stress and tBID accumulation, similar to the observations upon HSV-2 infection (Fig EV4A), and CHX treatment inhibited MCL-1 translation, similar to HSV-2 infection (Fig EV4B). Noticeably, TG treatment alone caused mitochondrial dysfunction and stimulated cleavage of CASP3, PARP, and GSDME and accompanying necrotic cell death (Figs 5D and E, and EV4C). This response was dependent on BID (Fig EV4D). In contrast to this, CHX treatment had a more modest effect on mitochondrial function, GSDME cleavage, and necrosis (Figs 5D and F, and EV4E). Although HSV-2 infection led to reduced levels of MCL-1 in SH-SY5Y cells (Fig EV4B), it did not cause general decrease in protein levels, which was observed after CHX treatment (Fig EV4F).

To further explore the effect of ER stress on cell death induced by HSV-2 infection, we inhibited this response using tauroursodeoxycholic acid (TUDCA). TUDCA augments ER chaperone activity upon ER stress (Vang *et al*, 2014). Importantly, we found that pretreatment with TUDCA inhibited HSV-2-induced cleavage of CASP3, PARP, and GSDME, as well as release of HMGB1 and cytochrome c

© 2023 The Authors

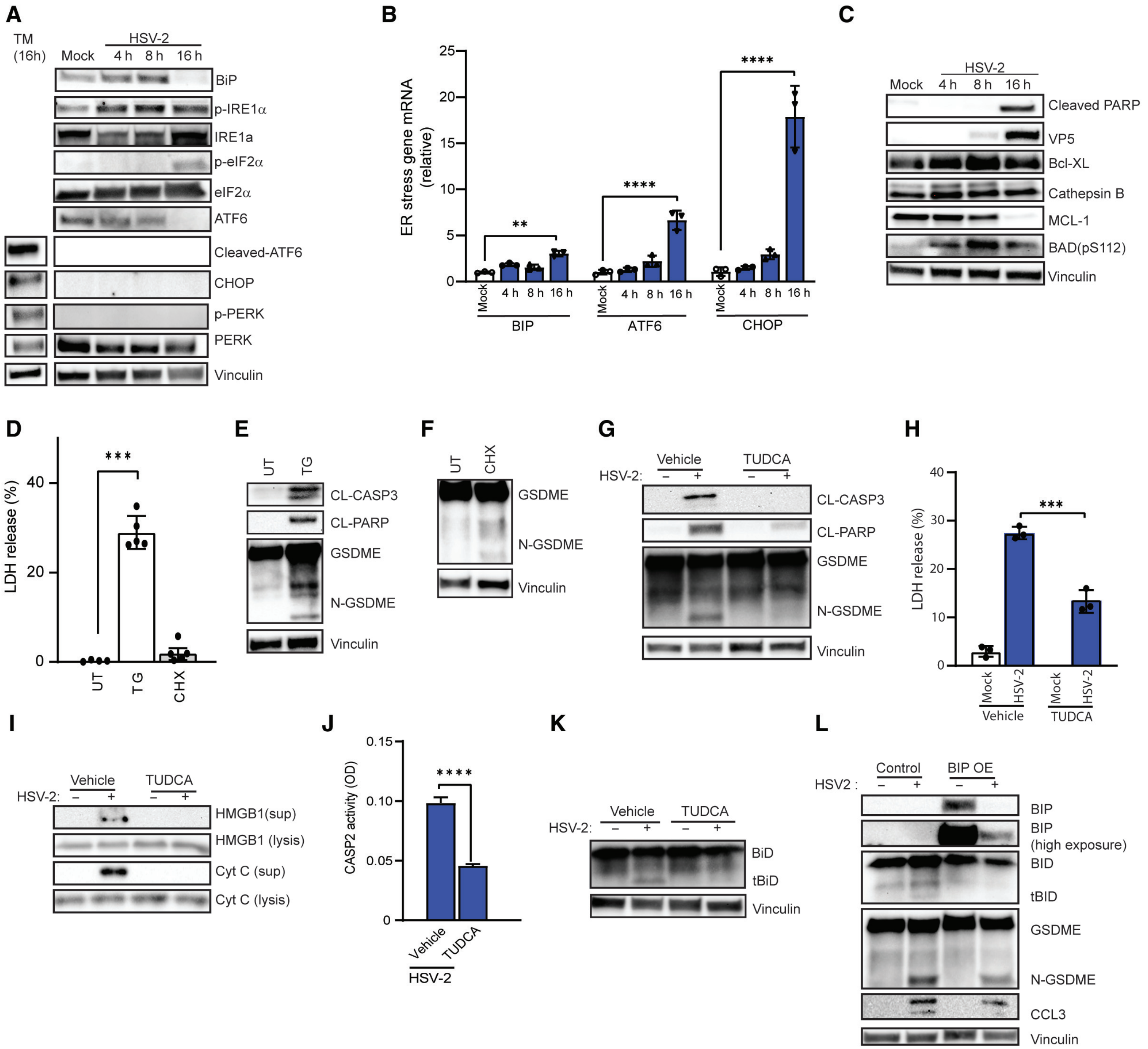

Figure 5. HSV-2-induced ER stress occurs upstream of pyroptosis.

A, B SH-SY5Y cells were infected with HSV-2 (MOI = 1) for the indicated time intervals, and immunoblotted for ER stress markers (BIP, phospho-IRE1α, phospho-eIF2α, cleaved-ATF6, and CHOP) (A), and analyzed for mRNA levels of the ER stress response genes BIP, ATF6, and CHOP by RT–qPCR (B).

C Lysate from SH-SY5Y cells with HSV-2 infection (MOI = 1) were immunoblotted for BCL2 family proteins MCL-1, Bcl-XL, and phospho-BAD (S112). Vinculin was used as loading control.

D–F SH-SY5Y cells were stimulated with thapsigargin (TG, 20 μM, 24 h) and cycloheximide (CHX, 10 μg/ml, 24 h) and evaluated for LDH release (D) and immunoblotted for GSDME cleavage (E, F).

G–K SH-SY5Y cells were pretreated with TUDCA (500 ng/ml) for 1 h and infected with HSV-2 (MOI = 1). Lysates and supernatant were isolated after 16 h of HSV-2 infection and subjected to immunoblotting for CL-CASP3, CL-PARP, GSDME, HMGB1 (G, I), and tBID (K). The lysates (16 h) were also assayed for CASP2 activity (J), and supernatants (24 h) were analyzed for LDH release (H, ELISA).

L SH-SY5Y cells transduced with Lenti-plasmid BIP or empty vector control were infected with HSV-2 (MOI = 1, 16 h). Lysates were isolated and immunoblotted for CL-CASP3, CL-PARP, GSDME, and tBID.

Data information: All data shown are representative of at least three independent experiments. Data are presented as mean ± s.d. in all graphs. **$P \leq 0.01$; ***$P \leq 0.001$; ****$P \leq 0.0001$ (Mann–Whitney test, two-tailed in B, D, and J two-way ANOVA in H).
Source data are available online for this figure.

© 2023 The Authors

(Fig 5G–I). TUDCA treatment also inhibited HSV-2-induced phosphorylation of IRE1α, CASP2 activity, cleavage of BID, and CASP3 activity (Figs 5J and K, and EV4G and H). Accordingly, TUDCA treatment significantly mitigated the morphological changes and cell loss induced by HSV-2 infection (Fig EV4I). Moreover, overexpression of BIP, which exerts negative regulation of the ER stress sensors and was down-regulated in HSV-2-infected cells (Fig 5A), inhibited cleavage of CASP3 and GSDME (Fig 5L). Collectively, these data support that HSV-2-induced cell death in lytically infected neuronal cells is initiated by ER stress leading to activation of the CASP2-tBID axis, linking to mitochondrial dysfunction and CASP3/7-mediated GSDME cleavage.

ER stress-induced IRE1α activation contributes to HSV-2-induced pyroptosis

The data described above suggested that lytic HSV-2 infection both activates and interferes with the ER stress response, which plays a central role in pyroptosis. Since we observed clear activation of the IRE1α pathway (Fig 5A), but not the PERK or ATF6 pathways, we further examined the activity of IRE1α pathway in HSV-2-induced pyroptosis. The cytoplasmic part of IRE1α contains RNase and kinase activity, with the RNase activity exerting XBP1 RNA splicing (XBP1s) and IRE1α-dependent decay of mRNA (RIDD), and kinase activity triggering, for example, phosphorylation of JNK (Fig 6A). The different IRE1α-induced pathways exert dissimilar effects on cell survival (Maurel *et al*, 2014; Junjappa *et al*, 2018). First, we tested which of the IRE1α-induced pathways were activated by lytic HSV-2 infection. HSV-2 infection induced XBP1s splicing (Fig 6B) and activation of JNK (Fig 6C), but not activation of RIDD, as measured by degradation of the RIDD-associated mRNA Blos1 (Fig 6D). Notably, depletion of IRE1α attenuated GSDME cleavage, cytochrome c, and HMGB1 release (Fig 6E). Treatment with inhibitors that selectively block the RNase (i.e., 4u8c and STF-083010) *versus* kinase (i.e. APY029) activities of IRE1α showed that blocking of the kinase activity with strongly reduced HSV-2-induced GSDME cleavage (Fig 6F), whereas inhibiting IRE1α RNase had a neglected effect (Fig EV5A and B). In agreement with the XBP1s pathway not being involved in activation of pyroptosis, we found that activation of the IRE1α-XBP1s pathway using IXA4 (Fig EV5C) did not augment HSV-2-induced GSDME cleavage, and even modestly inhibited it (Fig EV5D). These data are in line with previous reports on a pro-survival role for XBP1s (Guo *et al*, 2012). Finally, we examined whether the kinase activity of IRE1α was responsible for HSV-2-induced activation of JNK, and whether JNK was involved in the virus-induced pyroptotic response. Blockage of the kinase activity of IRE1α with APY029 inhibited HSV-2-induced activation of JNK (Fig 6G), whereas blockage of JNK activation with SP600125 inhibited GSDME cleavage and release of HMGB1 (Fig 6H). Overall, the results indicate that the IRE1α-JNK pathway is the major driver of the GSDME-dependent pyroptosis in productively HSV-2-infected neuroblastoma cells.

Necrotic neurons induce inflammatory gene expression in microglia

Neuroinflammatory processes are considered an essential factor in pathology of various neuronal diseases, in which excessive cytokine production leads to unrecoverable brain damage. Cell death can also contribute to host defense in the context of viral infections (Orzalli *et al*, 2021). We found that GSDME deficiency did not affect HSV-2 replication (Appendix Fig S2A). To test whether material released from necrotic neuronal cells can trigger inflammatory immune responses, we set up an *in vitro* model where supernatants from wide-type and GSDME-deficient neuron-like cells were used to stimulate human iPSC-derived microglia, monocytic THP1 cells, and primary mouse mixed brain cells (Fig 7A). While supernatants from HSV-2-infected SH-SY5Y cells induced expression of inflammatory cytokines in iPSC-derived microglia, this was significantly lower if the cells were treated with supernatants from infected GSDME-depleted SH-SY5Y cells (Fig 7B–D). Notably, similar phenomena were seen in THP1 (Appendix Fig S2B–D) and mouse mixed brain cells (Appendix Fig S2E–G), indicating that HSV-2-induced pyroptosis augments the inflammatory immune response. Next, the pattern recognition receptors (PRRs) involved were determined by treating a panel of THP1 cell lines deficient for specific PRR signaling adaptors with supernatant from HSV-2-infected SH-SY5Y (Appendix Fig S2H). Deficiency of STING and MYD88 attenuated the transcription of the inflammatory cytokines *IL6*, *TNFA*, and *CXCL10* (Fig 7E–G). These data suggest that both the Toll-like receptors and the cGAS-STING pathway can induce inflammation in the brain in response to HSV-2-induced pyroptosis.

Discussion

Viral infections in the CNS may lead to tissue damage and disturbance of homeostasis. This may cause acute disease and also impair brain development as well as long-term cognitive and functional disability. For many neurotropic viruses, the infection of neurons can lead to cell death. For instance, this is seen during infections with Zika virus, West Nile virus, Sindbis virus, and HSV (Nargi-Aizenman & Griffin, 2001; DeBiasi *et al*, 2002; Samuel *et al*, 2007; Oh *et al*, 2017). In the case of HSV, the virus harbors proteins to actively prevent apoptotic responses in neurons (You *et al*, 2017), but neuronal cell death does occur and is a major player in disease pathogenesis (Whitley, 2015). Despite this, the mechanism through which HSV triggers neuronal cell death has not been known. In this study, we report that HSV-2 infection of neurons leads to cell death through a mechanism driven by GSDME. This is dependent on the CASP2-tBID mitochondrial axis and is induced following the virus-induced IRE1α-dependent ER stress response.

We confirmed that neuronal cell death occurs following infection with a broad range of viruses. By focusing on HSV-2-induced necrosis, we observed that the modality of PCD was primarily pyroptosis, and not to any major extent apoptosis or necroptosis. The definition of pyroptosis used in this work is GSDM-induced cell death (Broz *et al*, 2020; Liu *et al*, 2021). GSDMs execute pyroptosis following cleavage of the full-length proteins to release an N-terminal cleavage product, which forms pore in the plasma cell membrane (Broz *et al*, 2020). This leads to cell swelling and lysis, thus not only releasing intracellular content but also preventing microbial replication (Broz *et al*, 2020). Different GSDMs are cleaved by different cellular proteases. GSDMD is a substrate of the inflammasome-activated CASP1, 4/5 (human), and 11 (mice; Kayagaki *et al*, 2015; Shi *et al*, 2015), GSDMB is cleaved by granzyme A

 © 2023 The Authors

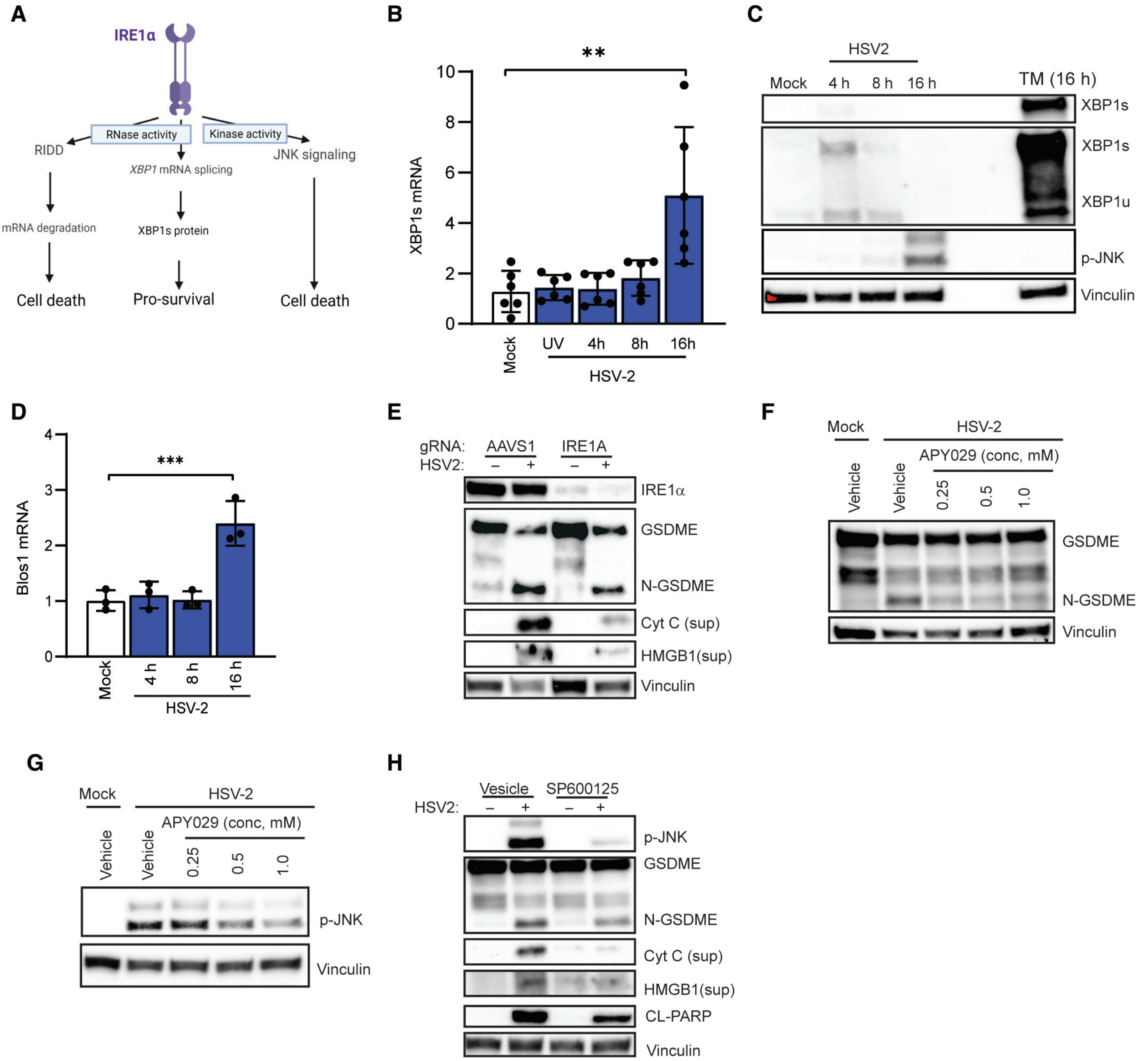

Figure 6. ER stress-induced IRE1-α activation contributes to HSV-2-induced pyroptosis.

A ER stress response induced downstream of IRE1α. The image was generated in Biorender.

B–D SH-SY5Y cells were infected with HSV-2 for the indicated time intervals. Total RNA was analyzed for spliced XBP1 (XBP1s) and Blos1 (RIDD target gene) by RT–qPCR (B and D). Lysates were immunoblotted for vinculin, phosphor-JNK and XBP1s (C).

E SH-SY5Y cells transfected with AAVS1 or IRE1A gRNA-Cas9 RNPs were infected with HSV-2 (MOI = 1, 16 h). Lysates and supernatant were isolated and immunoblotted for IRE1α, GSDME, cytochrome c, and HMGB1 release.

F, G SH-SY5Y cells were pretreated with APY29 for 1 h and following HSV-2 infection (16 h). Lysate was immunoblotted for GSDME cleavage and p-JNK.

H SH-SY5Y cells were pretreated with SP600125 (10 μM) for 1 h infected with HSV-2 (MOI = 1, 16 h). Lysates and supernatant were immunoblotted for GSDME, cytochrome c, HMGB1, and vinculin.

Data information: All data shown are representative of at least three independent experiments. Data are presented as mean ± s.d. in all graphs. $^{**}P \le 0.01$; $^{***}P \le 0.001$ (Mann–Whitney test, two-tailed in B and D).
Source data are available online for this figure.

(Zhou *et al*, 2020), while GSDME is cleaved by CASP3 (Rogers *et al*, 2017; Wang *et al*, 2017). The human neuroblastoma cell line SH-SY5Y used in this study did not express CASP1 and GSDMD, thus excluding canonical inflammatory pyroptosis. Rather, HSV-induced cell death in neurons was dependent on GSDME occurring downstream of CASP3 and 7. These two caspases have been

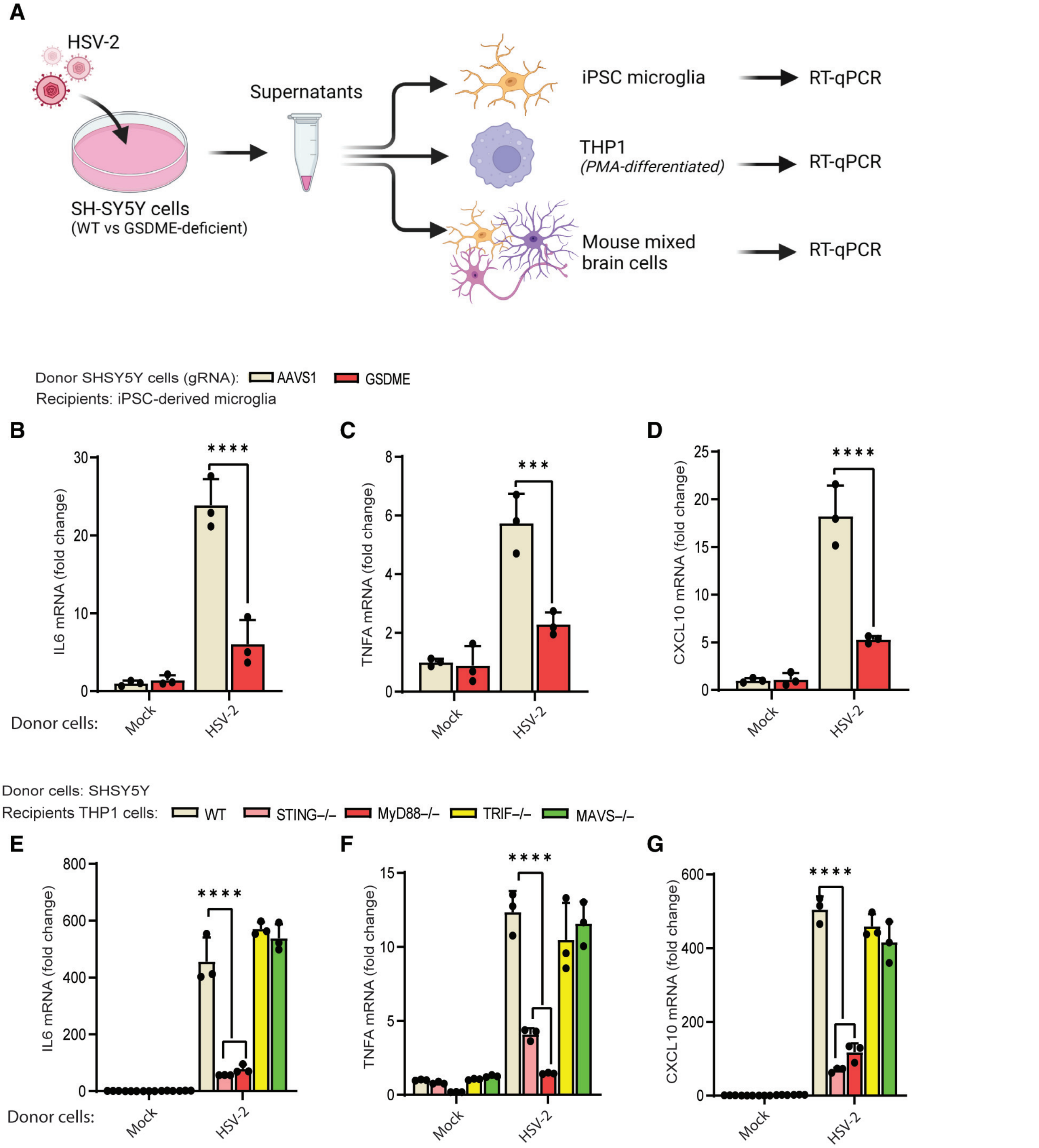

Figure 7. Necrotic neurons induce inflammatory gene expression in microglia.

A Supernatants from SH-SY5Y control and GSDME-depleted cells infected with HSV-2 (MOI = 1, 24 h) were diluted 1:10 and transferred to iPSC-derived microglia cells, THP1 cells, and mouse mixed brain cells to analysis for induction of inflammatory gene expression via RT–qPCR. Image was generated in Biorender.

B–D iPSC-derived microglia cells were treated with the conditioned medium from HSV-2-infected control and GSDME-depleted SH-SY5Y cells for 8 h. Total RNA was isolated and analyzed for expression of IL6, TNFA, and CXCL10 by RT–qPCR.

E–G Conditioned medium from SH-SY5Y cells with HSV-2 infection (MOI = 1, 24 h) was transferred to THP1 cells (WT, $STING^{-/-}$, $MYD88^{-/-}$, $TRIF^{-/-}$, and $MAVS^{-/-}$). Total RNA was isolated 8 h later and analyzed for expression of IL6 (E), TNFA (F), and CXCL10 (G) by RT–qPCR.

Data information: All data shown are representative of at least three independent experiments. Data are presented as mean ± s.d. in all graphs. ****P* ≤ 0.001; *****P* ≤ 0.0001 (two-way ANOVA in B–D, Wilcoxon matched-pairs test in E–G).

© 2023 The Authors

reported to work in redundant and non-redundant manners in different biological processes (Lakhani *et al*, 2006), and our data suggest that they have overlapping functions in HSV-2-induced pyroptosis in neuronal cells. Importantly, our finding in the SH-SY5Y cells was confirmed in primary mouse CNS cell cultures, human stem cell-derived NPCs, and hfOBSC. In agreement with this, Neel *et al* (2023) recently discovered that mitochondria toxins drive CASP3-GSDME-dependent neuron cell death via mitochondria damage and neurite loss (Neel *et al*, 2023). Thus, mitochondrial damage and downstream GSDME-mediated cell death are induced by HSV-2 infection as well as other cell stressors in neurons, and represent a common pathway for neuronal cell death.

As to the mechanism that triggers HSV-2-induced neuronal pyroptosis, we found this to be dependent on viral replication. Moreover, depletion of CASP2, BID, or mitochondrial DNA from SH-SY5Y cells reduced cleavage of CASP3 and GSDME as well as reduced release of alarmins. This was dependent on the DNA-binding protein IFI16, which has been reported to promote induction of apoptosis and pyroptosis by both RNA and DNA (Gugliesi *et al*, 2010; Monroe *et al*, 2014; Mishra *et al*, 2022). However, we did not detect elevated levels of mitochondrial DNA in the cytoplasm of HSV-2-infected cells, but this may well be explained by the ability of HSV to eliminate mitochondrial DNA with the nucleases UL12/UL12.5 (Saffran *et al*, 2007; Duguay *et al*, 2014). In addition, we found that HSV-2 infection led to strong activation of the ER stress, depletion of the anti-apoptotic protein MCL-1, and inactivation of BCL-xL. The latter was recently reported to trigger GSDME-dependent pyroptotic cell death in HSV-1-infected keratinocytes (Orzalli *et al*, 2021). Importantly, we observed that alleviation of ER stress with TUDCA, which acts as an ER stress inhibitor, reduced HSV-induced CASP2 activation, cleavage of BID, CASP3, and GSDME, and also release of alarmins. Overexpression of the ER chaperone and negative regulator of ER stress sensors BIP also reduced cleavage of CASP3 and GSDME. Conversely, induction of ER stress independent of infection through treatment with TG induced GSDME cleavage and necrosis in SH-SY5Y cells. Previous work has implicated CASP2, 3, and 7 in TG-induced cell death in SH-SY5Y cells (Dahmer, 2005). CASP2 has previously been shown to be activated through an NLRP3 inflammasome in response to ER stress, and dependent on mitochondrial reactive oxygen species, promoting NLRP3 association with mitochondria and downstream mitochondrial damage (Bronner *et al*, 2015). In HSV-2-infected neurons, NLRP3 and ROS, were not found to be involved in the induction of pyroptosis. Together, this suggests that HSV infection induces ER stress to activate the CASP2-tBID pathway to induce GSDME-mediated pyroptosis in neurons.

Among the various ER stress-activated cellular pathways, we found that HSV-2 infection strongly activated IRE1α, but not the PERK and ATF6 pathways. We did not detect accumulation of phosphorylated PERK, but did find accumulation of p-eIF2α and CHOP mRNA, which can be induced by PERK and other kinases, including protein kinase R (Lozon *et al*, 2011). However, since we did not observe accumulation of CHOP protein, and also found degradation of ATF6, the data imply that HSV-2 blocks the PERK and ATF6 pathways, thus giving rise to an ER stress response mainly driven by the IRE1α pathway. The viral inhibition of ER stress responses has previously been reported in HSV-1-infected cells (Burnett *et al*, 2012). The mechanisms through which HSV-2 inhibits the PERK and ATF6 pathways remain to be identified, but the known eIF2α phosphatase-enhancing activity of ICP34.5 and the E3 ubiquitin ligase activity of ICP0, respectively (Halford *et al*, 2010; Tang *et al*, 2013), are prime candidates to investigate. Notably, the IRE1α-driven cell death of HSV-2-infected cells is mediated by both the kinase activity of this ER stress sensor and the downstream kinase JNK. Since JNK is well-known to be involved in cell death (Chen *et al*, 2021), also in response to multiple viruses known to induce ER stress and PCD in neurons (Kim *et al*, 2004; Zhang *et al*, 2010), it is of interest to determine whether the herein identified mechanism is a general phenomenon of virus-induced neuronal cell death.

Neuronal cell death is a central event in a number of acute and chronic diseases. However, the PCD programs and signaling pathways remain poorly described. This includes the question as to whether there is overlap between mechanisms governing neuronal death in clinically different neurological diseases. Interestingly, death of hippocampal neurons in response to methamphetamine is mediated by GSDME and proceeds through the ER stress pathway (Liu *et al*, 2020), which is also highly active prior to neuronal necrotic cell death upon hypoxia–ischemia (Chavez-Valdez *et al*, 2016). In addition, the filaments derived from alpha-synuclein, polyQ-expanded huntingtin, and beta-amyloid, which are associated with the neurodegenerative disorders such as Parkinson's, Huntington's, and Alzheimer's diseases, respectively, also trigger ER stress in neurons (Smith *et al*, 2005; Kim *et al*, 2008; Leitman *et al*, 2013). Therefore, neurons may be particularly sensitive to ER stress-induced necrotic cell death, and the mechanism that we have uncovered in this study in the context of HSV-2 infection, involving ER stress-dependent IRE1α-induced CASP2-tBID-driven pyroptosis, may be involved in driving neuronal cell death in a broad range of pathologies. In this respect, it is interesting that a decrease in CASP2 expression in the brain reverses memory deficits in one model for Alzheimer's-like disease (Zhao *et al*, 2016), and caspase is also required for the cognitive decline observed in another model for the same disease (Pozueta *et al*, 2013). Moreover, mice lacking CASP2 are protected from behavioral changes in a model of Huntington's disease (Carroll *et al*, 2011). This urges more work to explore whether the mechanism described in this work is more broadly involved in neuronal cell death.

Necrotic forms of cell death lead to release of intracellular content and hence release of immunostimulatory molecules, including high-mobility group box 1, interleukin 1α, and ASC (Yang & Oppenheim, 2017). This can lead to enhancement of immunological processes, which may exacerbate pathological inflammation. In addition to the direct cytopathic effect of HSV, local inflammatory responses contribute to the pathogenesis of HSV CNS infections (Rouse & Sehrawat, 2010), and treatment with anti-inflammatory agents improves prognosis (Kamei *et al*, 2005). On the other hand, molecules released upon necrosis may contribute to antiviral defense, as reported for pyroptosis release of interleukin-1α in keratinocytes (Orzalli *et al*, 2021). The process of pyroptosis can also directly block viral replication by killing the virus-producing cells (Yogarajah *et al*, 2017; Orzalli *et al*, 2021). We found that pyroptosis of HSV-infected neurons did not alter viral replication. However, culture supernatants from infected wild-type, but not GSDME deficient, neuron-like cells potently induced a proinflammatory gene program in iPSC-derived microglia. This response was also observed in macrophage-like THP1 cells and was dependent on the pattern

recognition receptor signaling adaptor proteins MyD88 and STING. These pathways are known to play central roles in protective and deleterious responses to virus infections in the brain (Reinert *et al*, 2016; Ghita *et al*, 2021; Tang *et al*, 2021). For instance, the cGAS-STING pathway is vital in sensing cytosolic DNA to enhance antiviral immune response by inducing type I IFN and inflammatory responses (Kim *et al*, 2023). Additionally, it has been reported that cytoplasmic accumulation of TDP-43, associated with amyotrophic lateral sclerosis, leads to mitochondrial DNA release and activation of cGAS-STING pathway, which promotes excessive inflammatory response and neuronal loss (Yu *et al*, 2020). Thus, our work suggests that neuronal cell death in virus replication foci in the HSV-infected brain may promote inflammatory responses, which could amplify disease progression.

In summary, we report that productive HSV-2 replication in neurons can lead to ER stress and triggering of IRE1α-dependent pyroptotic cell death. This is mediated by GSDME, which is cleaved by CASP 3/7 downstream of the CASP2-tBID mitochondrial axis. The neuronal cell death leads to release of alarmins that induce a potent proinflammatory response in microglia. This novel mechanism of HSV-induced programmed cell death in neurons holds promise as target of new intervention strategies in viral CNS infections.

Materials and Methods

Cell culture and virus infection

Human neuroblastoma SH-SY5Y cells and monocyte THP1 cells were individually cultured in Dulbecco's modified Eagle's medium (DMEM) and RPMI 1640 medium with 10% fetal calf serum (FCS) and 1% penicillin–streptomycin at 37°C and 5% CO_2. All HSV infections were performed using HSV-1 KOS strain and HSV-2 333 strain with MOI 1 and MOI 3 in kinetics (4, 8, 16, and 24 h).

Human pluripotent stem cell culture

H9 (WA-09, WiCell) and iPSC-CCD (reprogrammed from human Foreskin fibroblasts, ATCC) cell lines were cultured as previously described (Denham & Dottori, 2011). Briefly, hESCs and hPSCs were cultured on irradiated human foreskin fibroblasts (HFF) in KSR media consisting of DMEM/nutrient mixture F-12, supplemented with b-mercaptoethanol 0.1 mM, non-essential amino acids 1%, glutamine 2 mM, penicillin 25 U/ml, streptomycin 25 μg/ml, and knockout serum replacement 20% (all from Life Technologies), supplemented with FGF-2 10 ng/ml (Peprotech) and activin A 10 ng/ml (R&D systems). All cells were cultured at 37°C 5% CO_2. Colonies were mechanically dissected every 7 days and transferred to freshly prepared HFF. Media were changed every second day.

Neural stem cell differentiation

hESCs or hPSCs were mechanically dissected into pieces ~ 0.5 mm in diameter and transferred to vitronectin-coated organ culture plates in N2B27 medium containing 1:1 mix of neurobasal medium with DMEM/F12 medium, supplemented with insulin/transferrin/selenium 1%, N2 1%, retinol-free B27 1%, glucose 0.3%, penicillin 25 U/ml, and streptomycin 25 μg/ml (all from Life Technologies) for 13 days. From day 0 to day 6, the cells were grown in N2B27 media with SB431542 10 μM (Tocris) and LDN-193189 100 nM (Stemgent). From day 6 to day 13, the cells were grown in N2B27 media with FGF-2 20 ng/ml (Peprotech). From day 13, the cells were re-plated on culture plate coated with poly-L-ornithine, fibronectin, and laminin (all from Sigma) and grown in neurobasal media supplemented with B27 1%, penicillin 25 U/ml, streptomycin 25 μg/ml, and glutamax 0.5%. The media were changed every 2nd day and the cells were stimulated at day 21.

hiPSC-derived microglia

WTSIi015-A iPS cells (EBiSC through Sigma-Aldrich) were maintained on Matrigel (Corning) in mTeSR1+ medium (Stemcell Technologies). IPSC colonies were dissociated into single cells using TrypLE Express (Thermo Fisher Scientific). 4*106 iPSCs were seeded per Aggrewell 800 (Stemcell Technologies) in a 24-well plate in 2 ml embryonic body medium (EBM; mTeSR1+ medium supplemented with 10 μM ROCK inhibitor, 50 ng/ml BMP-4, 20 ng/ml SCF, and 50 ng/ml VEGF-121 [all from Peprotech]). Cells were cultured for 4 days in Aggrewells to form embryonic bodies (EBs) with half media change (1 ml) every day. EBs were harvested using an inverted cell strainer (40 μm), and around 15 EBs were plated per six-well in hematopoietic medium (HM; X-VIVO 15 medium [Lonza] supplemented with 2 mM Glutamax, 100 U/ml penicillin, 100 μg/ml streptomycin, 55 μM β-mercaptoethanol, 100 ng/ml human M-CSF [Peprotech], and 25 ng/ml human IL-3 [Peprotech]). Every 7 days, 2 ml media were replaced by fresh HM. After around 30 days, primitive macrophage precursors could be harvested during the media change and plated in microglia medium (MiM; Advanced DMEM F12 medium [Gibco] supplemented with 2 mM glutamax, 100 U/ml penicillin, 100 μg/ml streptomycin, 55 μM β-mercaptoethanol, 100 ng/ml human IL-34 [Peprotech], and 10 ng/ml human GM-CSF [Peprotech]) at a density of 105 cells/cm^2. Finally, cells were differentiated in MiM for subsequent 6–9 days with full media change every other day.

hfOBSC preparation, culturing, and infection

Blood vessels and meninges were removed from the human fetal brain tissue and cut into pieces of about 0.5 × 0.5 cm. The brain tissue pieces were cut into 350-μm-thick slices using a vibratome (Leica VT1200S) in artificial cerebrospinal fluid (aCSF) under constant carbogen (95% O_2, 5% CO_2) bubbling and stored in fresh aCSF before further processing (Ravi *et al*, 2019; Reinert *et al*, 2021). Slices were transferred to 12 mm Transwell® with 0.4 μm pore polyester membrane inserts (Corning) and cultured at 37°C (95% O_2, 5% CO_2) in culture medium (Reinert *et al*, 2021). Culturing medium was replaced the day after slicing and every 2nd day.

The human organotypic brain slices were cultured for 3 days before HSV infection. Slices were infected with 1×10^6 PFU/ml of cell-free recombinant HSV-1-expressing green fluorescent protein (GFP) N-terminally tagged to VP16 (HSV-1-GFP) and recombinant HSV-2 (333) containing a GFP expression cassette in place of the US4 gene (HSV-2-GFP) for 1 h (La Boissiere *et al*, 2004; Kropp *et al*, 2020). The inoculum was discarded and the brain slices were washed with PBS and maintained in culture medium for 72 h

© 2023 The Authors

post-infection at 37°C (95% O_2, 5% CO_2). Supernatants were collected and refreshed every day. Images of the HSV-infected brain slices were monitored with a fluorescent microscope (Zeiss AXIO inverted microscope, 2.5× objective) at indicated time points and reconstituted by using Fiji ImageJ software 1.53 t.

Protein precipitation from supernatant

SH-SY5Y cells were seeded in 12-well plates at an intensity of 5×10^5 and cultivated overnight. Cells were cultivated in 500 μl reduced serum medium (opti-MEM), pretreated 1 h with various drugs, and infected with HSV. After 16 h HSV infection, supernatant was harvested and subsequently mixed with equal volume methanol and ¼ volume chloroform. The mixture was vortexed 20 s and spinned down at 20,000 *g* for 10 min at room temperature. After removing the upper liquid phase, another equal volume methanol was added and subsequently vortexed and spinned down at 20,000 *g* for 10 min. The pellet was dried at 55°C for 5 min after removing the upper liquid and resuspending in TBS.

Western blotting and Coomassie blue assay

Cells were seeded in 12-well plates at an intensity of 5×10^5, cultivated overnight, and treated with different drugs and infected with HSV as mentioned above. Cells were washed twice with cold PBS and lysed with cold RIPA buffer supplemented with NaF and benzonaze. Cell lysates and precipitated protein with the presence of 1× XT sample buffer and 1× XT reducing agent were denatured at 95°C for 5 min. Twenty milligram reduced sample was loaded and separated by SDS–PAGE and then transferred onto a PVDF membrane (Bio-Rad). Membranes were blocked with 5% milk diluted in TBST for 1 h at room temperature and then incubated with primary antibodies at 4°C overnight. Membranes were washed with TBS-T for 30 min and incubated with secondary antibodies at room temperature for 1 h. Proteins were exposed with SuperSignal chemiluminescent detection kit (Thermo Fisher Scientific, 23227). The SH-SY5Y cells were transduced by lentivirus packaged with pEGFP:T2A:Puro-hPGK-hHSPA5 (HSPA5, also named BIP). After selection, 1 μg/ml puromycin was used. The efficiency of gene delivery was analyzed by immunoblotting. Coomassie blue assay was performed by using SimplyBlue™ SafeStain (Invitrogen) according to the protocol.

MTT assay and lactate dehydrogenase activity

Cells were seeded in a 96-well plate at a density of 10×10^3 cells/well and incubated overnight with DMEM. The next day, DMEM was replaced with RPMI1640 medium. Cells were pretreated with different inhibitors for 1–2 h, and then cells were co-incubated with HSV and inhibitors. For MTT assay, 10 μl 98% thiazolyl blue tetrazolium bromide (Sigma) was added to each well and incubated for 4 h. After 4 h incubation, the medium was removed. Insoluble formazan was dissolved with DMSO. The plate was shaken for 10 min and read at 570 nm wavelength. For lactate dehydrogenase activity assay (LDH assay), 50 μl of supernatant was transferred to a new 96-well plate, and 50 μl reaction buffer was added. This was done for a period of 30 min incubation and read at 450 and 690 nm wavelength. Both assays should be protected from light. Two formulas were used to quantify LDH release:

$$\text{LDH release\%} = \left(\frac{\text{Compound}-\text{treated LDH activity}-\text{Spontaneous LDH activity}}{\text{Maximum LDH activity}-\text{Spontaneous LDH activity}}\right) * 100.$$

$$\text{LDH (OD, 450)} = A_{450,\text{test}} - A_{450,\text{blank}}$$

Flow cytometry assay for cell death

SH-SY5Y cells were seeded in 12-well plates with the same density described above and infected with HSV-2 for 16 h. After infection, cells were washed with PBS and incubated with 500 μl 1× annexin V binding buffer and 5 μl annexin V for 15 min at room temperature. Subsequently, cells were harvested and centrifuged at 300 *g* for 5 min. Cells were further washed with PBS and resuspended in 400 μl 1× annexin V buffer for flow cytometry assay.

Genome editing by CRISPR/Cas9

The single-guide RNAs (sgRNAs) used in this study were purchased from Integrated DNA Technologies (IDT). These modified sgRNAs contain 2′-O-methyl-3′-phosphorothioate modifications at the three terminal nucleotides of the 5′ and 3′ ends. Ribonucleoprotein (RNP) complexes were made by mixing 100 pmol sgRNA and 6 μg Cas9 nuclease V3 protein (IDT) in 5 μl Opti-MEM (Gibco) for incubation at room temperature for 25 min before electroporation. SH-SY5Y cells (300,000 cells) resuspended in 15 μl Opti-MEM were mixed with the RNP complexes and subjected to electroporation using the Lonza 4D electroporator (program CM138). The gene knockout efficiency was validated by Western blotting. The sgRNA spacer sequences were as follows:

AAVS1 sgRNA: 5′-GGGGCCACUAGGGACAGGAU-3′
GSDME sgRNA 1#: 5′-AAGUCCGACUCCACGACCAC-3′
GSDME sgRNA 2#: 5′-CUCCUCCAUUCCAGUGGUCG-3′
Caspase 2 sgRNA 1#: 5′-UGUAGGAUAUUGGGAGUGUG-3′
Caspase 2 sgRNA 2#: 5′-UUUAGAGUUUCCUGAUGAUG-3′
BiD sgRNA 1#: 5′-UCAACAACGGUUCCAGCCUC-3′
BiD sgRNA 2#: 5′-GAUGCACUCAUCCCUGAGGC-3′
Caspase 3 sgRNA 1#: 5′-CUAAACAGAAAGAUCAUACA-3′
Caspase 3 sgRNA 2#: 5′-GGAAGCGAAUCAAUGGACUC-3′
Caspase 7 sgRNA 1#: 5′-GCCCUGAUCAUCUGCCAUCU-3′
Caspase 7 sgRNA 2#: 5′-UCCCAGAUGGCAGAUGAUCA-3′
IFI16 sgRNA 1#: 5′-ACUGACCACAAUCAACUGUG-3′
TRIF sgRNA: 5′-CGAAGGCGCUAGGAAGUGAU-3′
MAVS sgRNA: 5′-AGGUGGCCCGCAGUCGAUCC-3′
MYD88 sgRNA: 5′-CUGUCUCUUCCCCACAGAGG-3′
STING sgRNA: 5′-CAGUCCUCCAGUAGCUGCCC-3′
IRE1α sgRNA 1#: 5′-UUCAGGAAGCGUCACUGUGC-3′
IRE1α sgRNA 2#: 5′-CAGCGUUGACACAAACAACA-3′.

Antibodies and reagents

The following primary antibodies were obtained from Cell Signaling: anti-cleaved PARP (5625, 1:1,000), anti-caspase 3 (14220s, 1:1,000), anti-cleaved caspase 3 (9664, 1:1,000),anti-caspase 6 (9762, 1:1,000), anti-caspase 7 (9492T, 1:1,000), anti-caspase 8 (4790, 1:1,000), anti-cleaved caspase 8 (9496T, 1:1,000), anti-JNK (9252,

1:1,000), anti-phospho-JNK (4668, 1:1,000), anti-phospho-Bad (Ser112) (9291), phospho-eIF2α (Ser51) (9721S, 1:1,000), anti-phospho-Bad (Ser136) (D25H8) (4366), anti-IRE1α (3294, 1:1,000), anti-XBP1s (12782, 1:1,000), anti-ATF6 (65880, 1:1,000), anti-CHOP (2895T, 1:1,000), anti-cytochrom c (11940, 1:1,000), anti-BIP (3177, 1:1,000), anti-cathepsin B (31718, 1:1,000), anti-MCL-1 (94296, 1:1,000), anti-BID (2002, 1:1,000), and anti-BCL-XL (27647, 1:1,000). Several antibodies were purchased from Abcam and Sigma: anti-caspase 1 (ab207802, 1:1,000), anti-caspase 2 (ab179520, 1:1,000), anti-caspase 4 (ab238124, 1:1,000), anti-caspase 5 (ab40887, 1:1,000), anti-caspase 9 (ab202068, 1:1,000), anti-DFNA5 also named as anti-GSDME (ab215191, 1:1,000), COX IV (ab33985, 1:1,000), caspase 7(ab255818, 1:1,000), HSV-1 + HSV-2 (ICP5) (ab6508, 1:10,000), anti-phospho-mlkl (ab196436, 1:1,000), anti-phospho-IRE1α (Abcam, ab124945), and HMGB1(ab79823,1:10,000) are from Abcam and anti-Vinculin (Sigma, 1:1,000). The secondary antibodies were purchased from Jackson ImmunoResearch: peroxidase-conjugated F(ab)2 donkey anti-mouse IgG (H + L) (1:10,000) and peroxidase-conjugated F(ab) 2 donkey anti-rabbit IgG (H + L) (1:10,000).

The reagent used in this study are as follows: Pan-caspase inhibitor Z-VAD-FMK (Sigma, V116), caspase 1 inhibitor Z-YVAD-FMK (Selleck Chemicals, S8507), caspase 2 inhibitor Z-VDVAD (R&D Systems, FMK003), caspase 3 inhibitor Z-DEVD FMK (R&D Systems, fmk004), caspase 6 inhibitor Z-VEID (R&D Systems, FMK006), caspase 8 inhibitor Z-IETD-FMK (Selleck Chemicals, S7314), caspase 9 inhibitor (Selleck Chemicals, S7313), NLRP3 inhibitor MCC950 (Invivogen, inh-mcc), nigericin (Selleck Chemicals, S6653), lipopolysaccharides (LPS) (Selleck Chemicals, L2637), staurosporine (Selleck Chemicals, S1421), IRE1α RNase inhibitor STF-083010 (Sigma, SML0409), IRE1α RNase inhibitor 4u8c (Selleck Chemicals, S7272), IRE1α kinase inhibitor APY29 (Sigma, SML2381), IRE1α/XBP1s activator IXA4 (Selleck Chemicals, S9797), phosphor-JNK inhibitor SP600125 (Invivogen, tlrl-SP60), acyclovir (Sigma, PHR1254), cycloheximide (CHX, 2112 s, Cell Signaling), acycloguanosine (Sigma, A4669), tauroursodeoxycholic acid (TUDCA, Sigma, 580549), N-acetylcysteine (NAC, Sigma, A9165), recombinant human TNF-alpha Protein (R&D, 210-TA), birinapant (MedChemExpress, HY-16591), thapsigargin (Selleck Chem, S7895), tunicamycin (VWR, 654380–10), necrostain-1 (NEC-1) (Selleck chem, S8037) (Sigma, 480073), ethidium bromide (EtBr, E1510-10ML, Sigma Aldrich), uridine (U3750, Sigma Aldrich), caspase 2 activity assay kit (Abcam, ab39830), caspase 3 activity assay kit (Abcam, ab39401), cytochrome c release assay kit (Abcam, ab65311), annexin V conjugates for apoptosis detection (Invitrogen™, A13201), and CyQUANT™ LDH cytotoxicity assay kit (Thermo Fisher Scientific, C20301).

Caspase 2 and 3 activity assays

SH-SY5Y cells were seeded in 10 mm dishes (5×10^5 cells) and infected with HSV-2 (MOI = 1) at the time intervals specified for the individual experiments. The cells were harvested, centrifuged (300 *g*, 5 min), and washed twice with cold PBS. The pellet was resuspended in 250 µl chilled cell lysis buffer supplied with the caspase 2/3 assay kits (Abcam ab39830 and ab39401) and incubated on ice for 10 min. The samples were centrifuged at 10,000 *g* for 10 min and the supernatant was transferred to a new tube. All procedures were performed at 4°C. For each assay, cytosolic extracts (100–200 µg) diluted in 50 µl cell lysis buffer were mixed with 50 µl 2× reaction buffer and 5 µl VDVAD-*p*-NA substrate (caspase 2 assay) or DEVD-*p*-NA substrate (caspase 3 assay). The mixed reaction samples were incubated at 37°C for 2 h and read using SpectraMax iD3 at 400 nm. The OD value minus background was used for data analysis.

Establishing mitochondria DNA depletion with ethidium bromide

According to the published protocol, the human neuroblastoma cells were seeded in T-75 flask. The cells were treated with EtBr (2 µg/ml) and supplemented with 100 µg/ml sodium pyruvate and 50 µg/ml uridine for 1 month. Mitochondria DNA COX 1 and COX 2 are qualified by b-actin to detect mtDNA depletion efficiency.

SYBR green qRT–PCR program for mtDNA and genome DNA gene expression

Phenol:chloroform:isoamyl solution (PH = 7,7 Cat#77617 Sigma) was used to isolate whole-genome DNA and mtDNA. DNeasy Blood & Tissue kit (Cat. No. 69506) was used to extract mtDNA from the cytosolic supernatant. Ice-cold PBS was used to wash cells simulated with thapsigargin (20 µM, 24 h), CHX (10 µg/ml, 24 h), or infected with HSV-2 (16 h), which were centrifuged at 600 *g* for 5 min at 4°C. The pellet was resuspended in 150 µl digitonin lysis buffer (NaCl 150 mM, HEPES 50 mM, and digitonin 200 µg/ml to 10 ml) on ice for 15 min and centrifuged at 1,000 *g* for 10 min to remove cell debris and organism fragments. The supernatant was centrifuged at 20,000 for 30 min to get purified cytosolic supernatant. The collected supernatant was mixed with 150 µl ethanol (96–100%) and skipped directly to step 4 in the DNeasy Blood & Tissue kit. SYBR® Green QRT–PCR Master Mix (Agilent Technologies, 600886) was used to generate cDNA. The qPCR program for mtDNA detection was performed according to the paper published by Bronner & O'Riordan (2016).

The following primers used were as follows:

Gene name	Forward	Reverse
COX-1	CCTGACTGGCATTGTATTAG	GATGGGATGTTTCATGTG
COX-2	TTCATGATCACGCCCTCATA	CGGGAATTGCATCTGTTTTTA
ND1	ATGGCCAACCTCCTACTCCT	GCGGTGATGTAGAGGGTGAT
BIP	CAACCAAAGACGCTGGAACTATT	CCCTCTTATCCAGGCCATAAGC
ATF6	ACCTCAGCCACTTTCTCCAG	GGCTCCGCTGAAGAGAGACTATT
CHOP	AGAACCAGGAAACGGAAACAGA	TCTCCTTCATGCGCTGCTTT
XBP1s	TGCTGAGTCCGCAGCAGGTG	GCTGGCAGGCTCTGGGGAAG
Blos1	TACAGGTCCAGGCTGCCCAATT	TTCCAGTGCAGTGGCAATGGTG
B-Actin	ATCATGTTTGAGACCTTCAACA	CATCTCTTGCTCGAAGTCCA
18 s	TAGAGGGACAAGTGGCGTTC	CGCTGAGCCAGTCAGTGT

qRT–PCR with TaqMan gene expression assays

HiPSC-derived cells were lysed in RLT plus buffer (Qiagen) supplemented with 4 mM DTT (Sigma) and RNA was isolated using the

 © 2023 The Authors

RNeasy Mini Kit (Qiagen). cDNA was synthesized using the high-capacity cDNA kit (Thermo Fisher). Kit was used to isolate RNA from THP1 and mouse mixed brain cells. Quantitative PCR was performed using the following TaqMan gene expression assays (Applied Biosystems): ACTB (Hs01060665_g1), IL6: Hs00174131_m1, B-Act: Hs00357333_g1, TNFa: Hs00174128_m1, IFNB: Hs01077958_s1, and CXCL10: Hs00171042_m1. mRNA levels of interest were normalized to the housekeeping gene ACTB using the ΔΔCT method.

Isolation of primary mouse mixed brain cells.

Mixed brain cells were isolated from P0 to P3 mice. The brains were collected in Hank's balanced salt solution (HBSS; Gibco), and the meninges were lifted carefully. The brains were minced and washed in an HBSS medium followed by a 2.5% trypsin (Gibco) treatment. After 15 min, the reaction was stopped by the addition of culture media (DMEM 1640 supplemented with 10% FBS, 100 U/mL penicillin, and 100 mg/mL streptomycin). The cells were triturated and filtered through a 70 mm pore size filter and seeded on poly-D-lysine (Sigma-Aldrich) and laminin (Invitrogen) pre-coated 24-well plates. The media were changed every 2 days until day 6 when it was used for assay.

Conditioned media experiments on THP1, human hiPSC-derived microglia, and mouse mixed brain cells

Cell media supernatants from AAVS1 control and GSDME KO cells were diluted 1:10 with the respective cell media. THP1 cells, human iPSC-driven microglia cells, and mouse mixed brain cells were grown in 24-well plates and incubated with diluted supernatants for 8 h. Cells were washed once with PBS, and RNA was isolated using the RNeasy micro kit (Qiagen) according to the manufacturer's instructions, including DNase digestion.

Statistical analysis

Statistical analysis and graphs were performed using GraphPad Prism9. Mean and standard deviation (SD) were used to analyze the difference among groups with one-way or two-way ANOVA tests.

Data availability

This study includes no data deposited in external repositories.

Expanded View for this article is available online.

Acknowledgements

This work was supported by the Independent Research Fund Denmark (0134-00008B; 0134-00006B); The European Research Council (ERC-AdG ENVISION; 786602); The Novo Nordisk Foundation (NNF18OC0030274; NNF20OC0063436; NNF21OC0067157); China Scholarship Council; and The Lundbeck Foundation (R276-2018-192; R359-2020-2287). We thank the FACS Core Facility, Aarhus University, and technician Ea Stoltze Andersen for technical support.

Author contributions

Søren R Paludan: Conceptualization; formal analysis; writing – original draft; writing – review and editing. **Ryo Narita:** Data curation. **Rasmus O Bak:** Supervision. **Georges MGM Verjans:** Resources; supervision. **Line, S Reinert:** Data curation. **Fanghui Ren:** Conceptualization; data curation; formal analysis; writing – original draft; writing – review and editing. **Ahmad Rashidi:** Data curation; formal analysis. **Stefanie Stefanie Fruhwürth:** Data curation. **Zongliang Gao:** Data curation. **Martin K Thomsen:** Supervision.

Disclosure and competing interests statement

The authors declare that they have no conflict of interest.

References

Aizawa E, Karasawa T, Watanabe S, Komada T, Kimura H, Kamata R, Ito H, Hishida E, Yamada N, Kasahara T *et al* (2020) GSDME-dependent incomplete pyroptosis permits selective IL-1alpha release under caspase-1 inhibition. *iScience* 23: 101070

Armien AG, Hu S, Little MR, Robinson N, Lokensgard JR, Low WC, Cheeran MC (2010) Chronic cortical and subcortical pathology with associated neurological deficits ensuing experimental herpes encephalitis. *Brain Pathol* 20: 738–750

Bloom DC (2016) Alphaherpesvirus latency: a dynamic state of transcription and reactivation. *Adv Virus Res* 94: 53–80

Bock FJ, Tait SWG (2020) Mitochondria as multifaceted regulators of cell death. *Nat Rev Mol Cell Biol* 21: 85–100

Bonzon C, Bouchier-Hayes L, Pagliari LJ, Green DR, Newmeyer DD (2006) Caspase-2-induced apoptosis requires bid cleavage: a physiological role for bid in heat shock-induced death. *Mol Biol Cell* 17: 2150–2157

Bronner DN, O'Riordan MX (2016) Measurement of mitochondrial DNA release in response to ER stress. *Bio Protoc* 6: e1839

Bronner DN, O'Riordan MX, He Y (2013) Caspase-2 mediates a Brucella abortus RB51-induced hybrid cell death having features of apoptosis and pyroptosis. *Front Cell Infect Microbiol* 3: 83

Bronner DN, Abuaita BH, Chen X, Fitzgerald KA, Nunez G, He Y, Yin XM, O'Riordan MX (2015) Endoplasmic reticulum stress activates the inflammasome via NLRP3- and caspase-2-driven mitochondrial damage. *Immunity* 43: 451–462

Broz P, Pelegrin P, Shao F (2020) The gasdermins, a protein family executing cell death and inflammation. *Nat Rev Immunol* 20: 143–157

Burnett HF, Audas TE, Liang G, Lu RR (2012) Herpes simplex virus-1 disarms the unfolded protein response in the early stages of infection. *Cell Stress Chaperones* 17: 473–483

Carroll JB, Southwell AL, Graham RK, Lerch JP, Ehrnhoefer DE, Cao LP, Zhang WN, Deng Y, Bissada N, Henkelman RM *et al* (2011) Mice lacking caspase-2 are protected from behavioral changes, but not pathology, in the YAC128 model of Huntington disease. *Mol Neurodegener* 6: 59

Cassady KA, Gross M, Roizman B (1998) The second-site mutation in the herpes simplex virus recombinants lacking the gamma134.5 genes precludes shutoff of protein synthesis by blocking the phosphorylation of eIF-2alpha. *J Virol* 72: 7005–7011

Chauvier D, Ankri S, Charriaut-Marlangue C, Casimir R, Jacotot E (2007) Broad-spectrum caspase inhibitors: from myth to reality? *Cell Death Differ* 14: 387–391

Chavez-Valdez R, Flock DL, Martin LJ, Northington FJ (2016) Endoplasmic reticulum pathology and stress response in neurons precede programmed necrosis after neonatal hypoxia-ischemia. *Int J Dev Neurosci* 48: 58–70

Chen J, Ye C, Wan C, Li G, Peng L, Peng Y, Fang R (2021) The roles of c-Jun N-Terminal Kinase (JNK) in infectious diseases. *Int J Mol Sci* 22: 9640

© 2023 The Authors

Chovatiya R, Medzhitov R (2014) Stress, inflammation, and defense of homeostasis. *Mol Cell* 54: 281–288

Dahmer MK (2005) Caspases-2, -3, and -7 are involved in thapsigargin-induced apoptosis of SH-SY5Y neuroblastoma cells. *J Neurosci Res* 80: 576–583

DeBiasi RL, Kleinschmidt-DeMasters BK, Richardson-Burns S, Tyler KL (2002) Central nervous system apoptosis in human herpes simplex virus and cytomegalovirus encephalitis. *J Infect Dis* 186: 1547–1557

Deng W, Bai Y, Deng F, Pan Y, Mei S, Zheng Z, Min R, Wu Z, Li W, Miao R *et al* (2022) Streptococcal pyrogenic exotoxin B cleaves GSDMA and triggers pyroptosis. *Nature* 602: 496–502

Denham M, Dottori M (2011) Neural differentiation of induced pluripotent stem cells. *Methods Mol Biol* 793: 99–110

Duguay BA, Saffran HA, Ponomarev A, Duley SA, Eaton HE, Smiley JR (2014) Elimination of mitochondrial DNA is not required for herpes simplex virus 1 replication. *J Virol* 88: 2967–2976

Faraco G, Fossati S, Bianchi ME, Patrone M, Pedrazzi M, Sparatore B, Moroni F, Chiarugi A (2007) High mobility group box 1 protein is released by neural cells upon different stresses and worsens ischemic neurodegeneration *in vitro* and *in vivo*. *J Neurochem* 103: 590–603

Fricker M, Tolkovsky AM, Borutaite V, Coleman M, Brown GC (2018) Neuronal cell death. *Physiol Rev* 98: 813–880

Ghita L, Spanier J, Chhatbar C, Mulenge F, Pavlou A, Larsen PK, Waltl I, Lueder Y, Kohls M, Jung K *et al* (2021) MyD88 signaling by neurons induces chemokines that recruit protective leukocytes to the virus-infected CNS. *Sci Immunol* 6: eabc9165

Ghoshal A, Das S, Ghosh S, Mishra MK, Sharma V, Koli P, Sen E, Basu A (2007) Proinflammatory mediators released by activated microglia induces neuronal death in Japanese encephalitis. *Glia* 55: 483–496

Girard S, Couderc T, Destombes J, Thiesson D, Delpeyroux F, Blondel B (1999) Poliovirus induces apoptosis in the mouse central nervous system. *J Virol* 73: 6066–6072

Gnann JW Jr, Whitley RJ (2017) Herpes simplex encephalitis: an update. *Curr Infect Dis Rep* 19: 13

Gugliesi F, De Andrea M, Mondini M, Cappello P, Giovarelli M, Shoenfeld Y, Meroni P, Gariglio M, Landolfo S (2010) The proapoptotic activity of the Interferon-inducible gene IFI16 provides new insights into its etiopathogenetic role in autoimmunity. *J Autoimmun* 35: 114–123

Guo FJ, Liu Y, Zhou J, Luo S, Zhao W, Li X, Liu C (2012) XBP1S protects cells from ER stress-induced apoptosis through Erk1/2 signaling pathway involving CHOP. *Histochem Cell Biol* 138: 447–460

Guo C, Sun L, Chen X, Zhang D (2013) Oxidative stress, mitochondrial damage and neurodegenerative diseases. *Neural Regen Res* 8: 2003–2014

Guo H, Omoto S, Harris PA, Finger JN, Bertin J, Gough PJ, Kaiser WJ, Mocarski ES (2015) Herpes simplex virus suppresses necroptosis in human cells. *Cell Host Microbe* 17: 243–251

Halford WP, Puschel R, Rakowski B (2010) Herpes simplex virus 2 ICP0 mutant viruses are avirulent and immunogenic: implications for a genital herpes vaccine. *PloS One* 5: e12251

Han J, Cheng C, Zhang J, Fang J, Yao W, Zhu Y, Xiu Z, Jin N, Lu H, Li X *et al* (2022) Myricetin activates the Caspase-3/GSDME pathway via ER stress induction of pyroptosis in lung cancer cells. *Front Pharmacol* 13: 959938

Harris HE, Andersson U, Pisetsky DS (2012) HMGB1: a multifunctional alarmin driving autoimmune and inflammatory disease. *Nat Rev Rheumatol* 8: 195–202

Imre G, Heering J, Takeda AN, Husmann M, Thiede B, zu Heringdorf DM, Green DR, van der Goot FG, Sinha B, Dötsch V *et al* (2012) Caspase-2 is an initiator caspase responsible for pore-forming toxin-mediated apoptosis. *EMBO J* 31: 2615–2628

Johnson KE, Chikoti L, Chandran B (2013) Herpes simplex virus 1 infection induces activation and subsequent inhibition of the IFI16 and NLRP3 inflammasomes. *J Virol* 87: 5005–5018

Jorgensen I, Rayamajhi M, Miao EA (2017) Programmed cell death as a defence against infection. *Nat Rev Immunol* 17: 151–164

Junjappa RP, Patil P, Bhattarai KR, Kim HR, Chae HJ (2018) IRE1alpha implications in endoplasmic reticulum stress-mediated development and pathogenesis of autoimmune diseases. *Front Immunol* 9: 1289

Kaewpoowat Q, Salazar L, Aguilera E, Wootton SH, Hasbun R (2016) Herpes simplex and varicella zoster CNS infections: clinical presentations, treatments and outcomes. *Infection* 44: 337–345

Kamei S, Sekizawa T, Shiota H, Mizutani T, Itoyama Y, Takasu T, Morishima T, Hirayanagi K (2005) Evaluation of combination therapy using aciclovir and corticosteroid in adult patients with herpes simplex virus encephalitis. *J Neurol Neurosurg Psychiatry* 76: 1544–1549

Kayagaki N, Stowe IB, Lee BL, O'Rourke K, Anderson K, Warming S, Cuellar T, Haley B, Roose-Girma M, Phung QT *et al* (2015) Caspase-11 cleaves gasdermin D for non-canonical inflammasome signalling. *Nature* 526: 666–671

Kim SM, Park JH, Chung SK, Kim JY, Hwang HY, Chung KC, Jo I, Park SI, Nam JH (2004) Coxsackievirus B3 infection induces cyr61 activation via JNK to mediate cell death. *J Virol* 78: 13479–13488

Kim HJ, Cho HK, Kwon YH (2008) Synergistic induction of ER stress by homocysteine and beta-amyloid in SH-SY5Y cells. *J Nutr Biochem* 19: 754–761

Kim ID, Lee H, Kim SW, Lee HK, Choi J, Han PL, Lee JK (2018) Alarmin HMGB1 induces systemic and brain inflammatory exacerbation in post-stroke infection rat model. *Cell Death Dis* 9: 426

Kim J, Kim HS, Chung JH (2023) Molecular mechanisms of mitochondrial DNA release and activation of the cGAS-STING pathway. *Exp Mol Med* 55: 510–519

Korsmeyer SJ, Wei MC, Saito M, Weiler S, Oh KJ, Schlesinger PH (2000) Pro-apoptotic cascade activates BID, which oligomerizes BAK or BAX into pores that result in the release of cytochrome c. *Cell Death Differ* 7: 1166–1173

Kropp KA, Lopez-Munoz AD, Ritter B, Martin R, Rastrojo A, Srivaratharajan S, Dohner K, Dhingra A, Czechowicz JS, Nagel CH *et al* (2020) Herpes simplex virus 2 counteracts neurite outgrowth repulsion during infection in a nerve growth factor-dependent manner. *J Virol* 94: e01370-20

Krysko DV, D'Herde K, Vandenabeele P (2006) Clearance of apoptotic and necrotic cells and its immunological consequences. *Apoptosis* 11: 1709–1726

Kumar S (2009) Caspase 2 in apoptosis, the DNA damage response and tumour suppression: enigma no more? *Nat Rev Cancer* 9: 897–903

La Boissiere S, Izeta A, Malcomber S, O'Hare P (2004) Compartmentalization of VP16 in cells infected with recombinant herpes simplex virus expressing VP16-green fluorescent protein fusion proteins. *J Virol* 78: 8002–8014

Lakhani SA, Masud A, Kuida K, Porter GA Jr, Booth CJ, Mehal WZ, Inayat I, Flavell RA (2006) Caspases 3 and 7: key mediators of mitochondrial events of apoptosis. *Science* 311: 847–851

Leitman J, Ulrich Hartl F, Lederkremer GZ (2013) Soluble forms of polyQ-expanded huntingtin rather than large aggregates cause endoplasmic reticulum stress. *Nat Commun* 4: 2753

Lemke G (2019) How macrophages deal with death. *Nat Rev Immunol* 19: 539–549

Li S, Kong L, Yu X (2015) The expanding roles of endoplasmic reticulum stress in virus replication and pathogenesis. *Crit Rev Microbiol* 41: 150–164

© 2023 The Authors

Liu Y, Wen D, Gao J, Xie B, Yu H, Shen Q, Zhang J, Jing W, Cong B, Ma C (2020) Methamphetamine induces GSDME-dependent cell death in hippocampal neuronal cells through the endoplasmic reticulum stress pathway. *Brain Res Bull* 162: 73–83

Liu X, Xia S, Zhang Z, Wu H, Lieberman J (2021) Channelling inflammation: gasdermins in physiology and disease. *Nat Rev Drug Discov* 20: 384–405

Lozon TI, Eastman AJ, Matute-Bello G, Chen P, Hallstrand TS, Altemeier WA (2011) PKR-dependent CHOP induction limits hyperoxia-induced lung injury. *Am J Physiol Lung Cell Mol Physiol* 300: L422–L429

Lum FM, Low DK, Fan Y, Tan JJ, Lee B, Chan JK, Renia L, Ginhoux F, Ng LF (2017) Zika virus infects human fetal brain microglia and induces inflammation. *Clin Infect Dis* 64: 914–920

Luo X, Budihardjo I, Zou H, Slaughter C, Wang X (1998) Bid, a Bcl2 interacting protein, mediates cytochrome c release from mitochondria in response to activation of cell surface death receptors. *Cell* 94: 481–490

Mangan MSJ, Olhava EJ, Roush WR, Seidel HM, Glick GD, Latz E (2018) Targeting the NLRP3 inflammasome in inflammatory diseases. *Nat Rev Drug Discov* 17: 688

Marciniak SJ, Chambers JE, Ron D (2022) Pharmacological targeting of endoplasmic reticulum stress in disease. *Nat Rev Drug Discov* 21: 115–140

Maruzuru Y, Ichinohe T, Sato R, Miyake K, Okano T, Suzuki T, Koshiba T, Koyanagi N, Tsuda S, Watanabe M *et al* (2018) Herpes simplex virus 1 VP22 inhibits AIM2-dependent inflammasome activation to enable efficient viral replication. *Cell Host Microbe* 23: 254–265.e7

Maurel M, Chevet E, Tavernier J, Gerlo S (2014) Getting RIDD of RNA: IRE1 in cell fate regulation. *Trends Biochem Sci* 39: 245–254

McIlwain DR, Berger T, Mak TW (2013) Caspase functions in cell death and disease. *Cold Spring Harb Perspect Biol* 5: a008656

Min Z, Baddley JW (2014) Mollaret's meningitis. *Lancet Infect Dis* 14: 1022

Mishra S, Raj AS, Kumar A, Rajeevan A, Kumari P, Kumar H (2022) Innate immune sensing of influenza A viral RNA through IFI16 promotes pyroptotic cell death. *iScience* 25: 103714

Monroe KM, Yang Z, Johnson JR, Geng X, Doitsh G, Krogan NJ, Greene WC (2014) IFI16 DNA sensor is required for death of lymphoid CD4 T cells abortively infected with HIV. *Science* 343: 428–432

Nargi-Aizenman JL, Griffin DE (2001) Sindbis virus-induced neuronal death is both necrotic and apoptotic and is ameliorated by N-methyl-D-aspartate receptor antagonists. *J Virol* 75: 7114–7121

Neel DV, Basu H, Gunner G, Bergstresser MD, Giadone RM, Chung H, Miao R, Chou V, Brody E, Jiang X *et al* (2023) Gasdermin-E mediates mitochondrial damage in axons and neurodegeneration. *Neuron* 111: 1222–1240.e9

Oh Y, Zhang F, Wang Y, Lee EM, Choi IY, Lim H, Mirakhori F, Li R, Huang L, Xu T *et al* (2017) Zika virus directly infects peripheral neurons and induces cell death. *Nat Neurosci* 20: 1209–1212

Orvedahl A, MacPherson S, Sumpter R Jr, Talloczy Z, Zou Z, Levine B (2010) Autophagy protects against Sindbis virus infection of the central nervous system. *Cell Host Microbe* 7: 115–127

Orzalli MH, Prochera A, Payne L, Smith A, Garlick JA, Kagan JC (2021) Virus-mediated inactivation of anti-apoptotic Bcl-2 family members promotes Gasdermin-E-dependent pyroptosis in barrier epithelial cells. *Immunity* 54: 1447–1462.e5

Paludan SR, Bowie AG, Horan KA, Fitzgerald KA (2011) Recognition of herpesviruses by the innate immune system. *Nat Rev Immunol* 11: 143–154

Pozueta J, Lefort R, Ribe EM, Troy CM, Arancio O, Shelanski M (2013) Caspase-2 is required for dendritic spine and behavioural alterations in J20 APP transgenic mice. *Nat Commun* 4: 1939

Ravi VM, Joseph K, Wurm J, Behringer S, Garrelfs N, d'Errico P, Naseri Y, Franco P, Meyer-Luehmann M, Sankowski R *et al* (2019) Human organotypic brain slice culture: a novel framework for environmental research in neuro-oncology. *Life Sci Alliance* 2: e201900305

Reinert LS, Lopusna K, Winther H, Sun C, Thomsen MK, Nandakumar R, Mogensen TH, Meyer M, Vaegter C, Nyengaard JR *et al* (2016) Sensing of HSV-1 by the cGAS-STING pathway in microglia orchestrates antiviral defence in the CNS. *Nat Commun* 7: 13348

Reinert LS, Rashidi AS, Tran DN, Katzilieris-Petras G, Hvidt AK, Gohr M, Fruhwurth S, Bodda C, Thomsen MK, Vendelbo MH *et al* (2021) Brain immune cells undergo cGAS/STING-dependent apoptosis during herpes simplex virus type 1 infection to limit type I IFN production. *J Clin Invest* 131: e136824

Rogers C, Fernandes-Alnemri T, Mayes L, Alnemri D, Cingolani G, Alnemri ES (2017) Cleavage of DFNA5 by caspase-3 during apoptosis mediates progression to secondary necrotic/pyroptotic cell death. *Nat Commun* 8: 14128

Rouse BT, Sehrawat S (2010) Immunity and immunopathology to viruses: what decides the outcome? *Nat Rev Immunol* 10: 514–526

Saffran HA, Pare JM, Corcoran JA, Weller SK, Smiley JR (2007) Herpes simplex virus eliminates host mitochondrial DNA. *EMBO Rep* 8: 188–193

Samuel MA, Morrey JD, Diamond MS (2007) Caspase 3-dependent cell death of neurons contributes to the pathogenesis of West Nile virus encephalitis. *J Virol* 81: 2614–2623

Shi J, Zhao Y, Wang K, Shi X, Wang Y, Huang H, Zhuang Y, Cai T, Wang F, Shao F (2015) Cleavage of GSDMD by inflammatory caspases determines pyroptotic cell death. *Nature* 526: 660–665

Singh R, Letai A, Sarosiek K (2019) Regulation of apoptosis in health and disease: the balancing act of BCL-2 family proteins. *Nat Rev Mol Cell Biol* 20: 175–193

Smith WW, Jiang H, Pei Z, Tanaka Y, Morita H, Sawa A, Dawson VL, Dawson TM, Ross CA (2005) Endoplasmic reticulum stress and mitochondrial cell death pathways mediate A53T mutant alpha-synuclein-induced toxicity. *Hum Mol Genet* 14: 3801–3811

Szegezdi E, Logue SE, Gorman AM, Samali A (2006) Mediators of endoplasmic reticulum stress-induced apoptosis. *EMBO Rep* 7: 880–885

Tang S, Guo N, Patel A, Krause PR (2013) Herpes simplex virus 2 expresses a novel form of ICP34.5, a major viral neurovirulence factor, through regulated alternative splicing. *J Virol* 87: 5820–5830

Tang T, Guo Y, Xu X, Zhao L, Shen X, Sun L, Xie P (2021) BoDV-1 infection induces neuroinflammation by activating the TLR4/MyD88/IRF5 signaling pathway, leading to learning and memory impairment in rats. *J Med Virol* 93: 6163–6171

de Torre-Minguela C, Gomez AI, Couillin I, Pelegrin P (2021) Gasdermins mediate cellular release of mitochondrial DNA during pyroptosis and apoptosis. *FASEB J* 35: e21757

Tyler KL (2018) Acute Viral Encephalitis. *N Engl J Med* 379: 557–566

Upton JP, Austgen K, Nishino M, Coakley KM, Hagen A, Han D, Papa FR, Oakes SA (2008) Caspase-2 cleavage of BID is a critical apoptotic signal downstream of endoplasmic reticulum stress. *Mol Cell Biol* 28: 3943–3951

Vang S, Longley K, Steer CJ, Low WC (2014) The unexpected uses of urso- and tauroursodeoxycholic acid in the treatment of non-liver diseases. *Glob Adv Health Med* 3: 58–69

Wagner KW, Engels IH, Deveraux QL (2004) Caspase-2 can function upstream of bid cleavage in the TRAIL apoptosis pathway. *J Biol Chem* 279: 35047–35052

© 2023 The Authors

Wang Y, Gao W, Shi X, Ding J, Liu W, He H, Wang K, Shao F (2017) Chemotherapy drugs induce pyroptosis through caspase-3 cleavage of a gasdermin. *Nature* 547: 99–103

Whitley RJ (2015) Herpes simplex virus infections of the central nervous system. *Continuum* 21: 1704–1713

Wylie KM, Schrimpf JE, Morrison LA (2009) Increased eIF2alpha phosphorylation attenuates replication of herpes simplex virus 2 vhs mutants in mouse embryonic fibroblasts and correlates with reduced accumulation of the PKR antagonist ICP34.5. *J Virol* 83: 9151–9162

Xu W, Che Y, Zhang Q, Huang H, Ding C, Wang Y, Wang G, Cao L, Hao H (2021) Apaf-1 pyroptosome senses mitochondrial permeability transition. *Cell Metab* 33: 424–436.e10

Yang HZ, Oppenheim JJ (2017) Alarmins and immunity. *Immunol Rev* 280: 41–56

Yogarajah T, Ong KC, Perera D, Wong KT (2017) AIM2 inflammasome-mediated pyroptosis in enterovirus A71-infected neuronal cells restricts viral replication. *Sci Rep* 7: 5845

You Y, Cheng AC, Wang MS, Jia RY, Sun KF, Yang Q, Wu Y, Zhu D, Chen S, Liu MF *et al* (2017) The suppression of apoptosis by alpha-herpesvirus. *Cell Death Dis* 8: e2749

Yu CH, Davidson S, Harapas CR, Hilton JB, Mlodzianoski MJ, Laohamonthonkul P, Louis C, Low RRJ, Moecking J, De Nardo D *et al* (2020) TDP-43 triggers mitochondrial DNA release via mPTP to activate cGAS/STING in ALS. *Cell* 183: e18

Zhang HM, Ye X, Su Y, Yuan J, Liu Z, Stein DA, Yang D (2010) Coxsackievirus B3 infection activates the unfolded protein response and induces apoptosis through downregulation of p58IPK and activation of CHOP and SREBP1. *J Virol* 84: 8446–8459

Zhang YF, Zhou L, Mao HQ, Yang FH, Chen Z, Zhang L (2021) Mitochondrial DNA leakage exacerbates odontoblast inflammation through gasdermin D-mediated pyroptosis. *Cell Death Discov* 7: 381

Zhao X, Kotilinek LA, Smith B, Hlynialuk C, Zahs K, Ramsden M, Cleary J, Ashe KH (2016) Caspase-2 cleavage of tau reversibly impairs memory. *Nat Med* 22: 1268–1276

Zhou Z, He H, Wang K, Shi X, Wang Y, Su Y, Wang Y, Li D, Liu W, Zhang Y *et al* (2020) Granzyme A from cytotoxic lymphocytes cleaves GSDMB to trigger pyroptosis in target cells. *Science* 368: eaaz7548

License: This is an open access article under the terms of the Creative Commons Attribution License, which permits use, distribution and reproduction in any medium, provided the original work is properly cited.

Expanded View Figures

Figure EV1. HSV induces GSDME-dependent pyroptotic cell death in neuron-like cells.

A SH-SY5Y cells were infected with HSV-2 (MOI = 1) for 8 and 16 h, and inspected by microscopy for morphological changes. Boxes indicate areas highlighted in the zoomed images. Arrows indicate ballooning or syncytial cells.

B SH-SY5Y cells were treated with UV-inactivated and infectious HSV-2 (MOI = 1, 16 h). Lysates were isolated and immunoblotted for CL-CASP3, GSDME, and VP5 as indicated.

C–E SH-SY5Y cells were pretreated with CHX (10 μg/ml) for 1 h and infected with HSV-2 (MOI = 1). Lysates were isolated and immunoblotted for apoptotic markers, GSDME, and VP5 as indicated, and supernatants were analyzed for LDH release and HMGB1 content.

F–I SH-SY5Y cells were treated with ACV (50 μM) for 1 h and infected with HSV-2 (MOI = 1) for 24 h. Supernatants were analyzed for HSV-2 viral load and LDH release (F, I). Lysates and supernatant from the infected ACV-pretreated SH-SY5Y cells (16 h) were further immunoblotted for CL-CASP3, CL-PARP, GSDME, and HMGB1(G, I).

J–M SH-SY5Y cells pretreated with Z-VAD (20 μM, 1 h) and/or treated with gRNA-Cas9 RNP complexes targeting CASP3 and 7 were infected with HSV-2 (MOI = 1) for 16 and 24 h. Supernatants isolated 24 h post-infection were used to evaluate LDH release (J), and cell lysates and supernatants isolated 16 h post-infection were immunoblotted with the indicated antibodies (K–M). For CRISPR/Cas knockout experiments, a gRNA targeting the safe-harbor locus AAVS1 was included as a negative control.

Data information: All data shown are representative of at least three independent experiments. Data are presented as mean ± s.d. in all graphs. ***$P \leq 0.001$; ****$P \leq 0.0001$ (Mann–Whitney test, two-tailed in F, two-way ANOVA in D, H, and J).
Source data are available online for this figure.

© 2023 The Authors

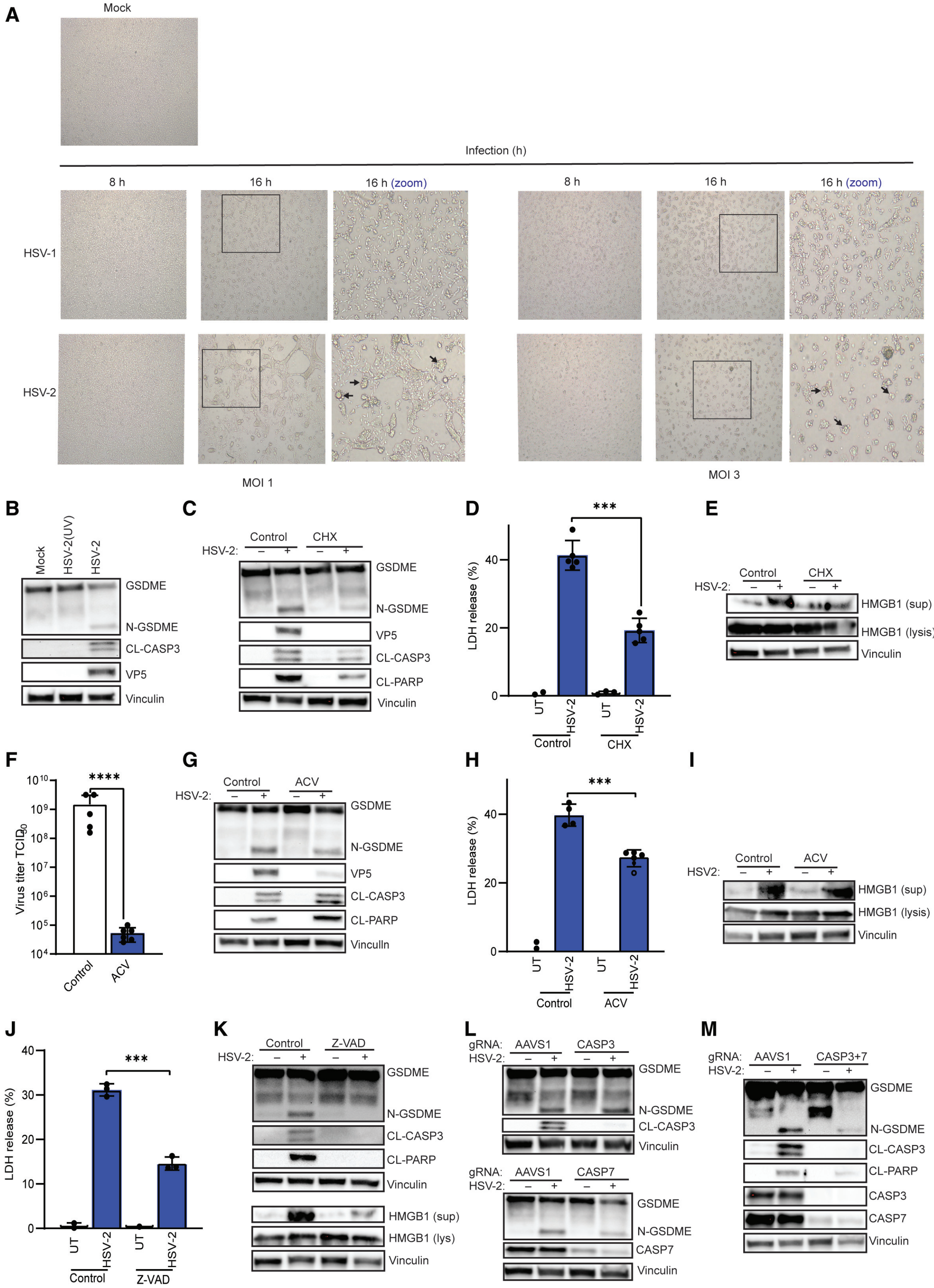
A
Mock
Infection (h)
8 h
16 h
16 h (zoom)
HSV-1
HSV-2
MOI 1
MOI 3
B
Mock
HSV-2(UV)
HSV-2
GSDME
N-GSDME
CL-CASP3
VP5
Vinculin
C
Control
CHX
HSV-2:
CL-PARP
D
LDH release (%)
UT
E
HMGB1 (sup)
HMGB1 (lysis)
F
Virus titer TCID50
ACV
G
H
I
HSV2:
J
Z-VAD
K
HMGB1 (lys)
L
gRNA:
AAVS1
CASP3
CASP7
M
CASP3+7

Figure EV1.

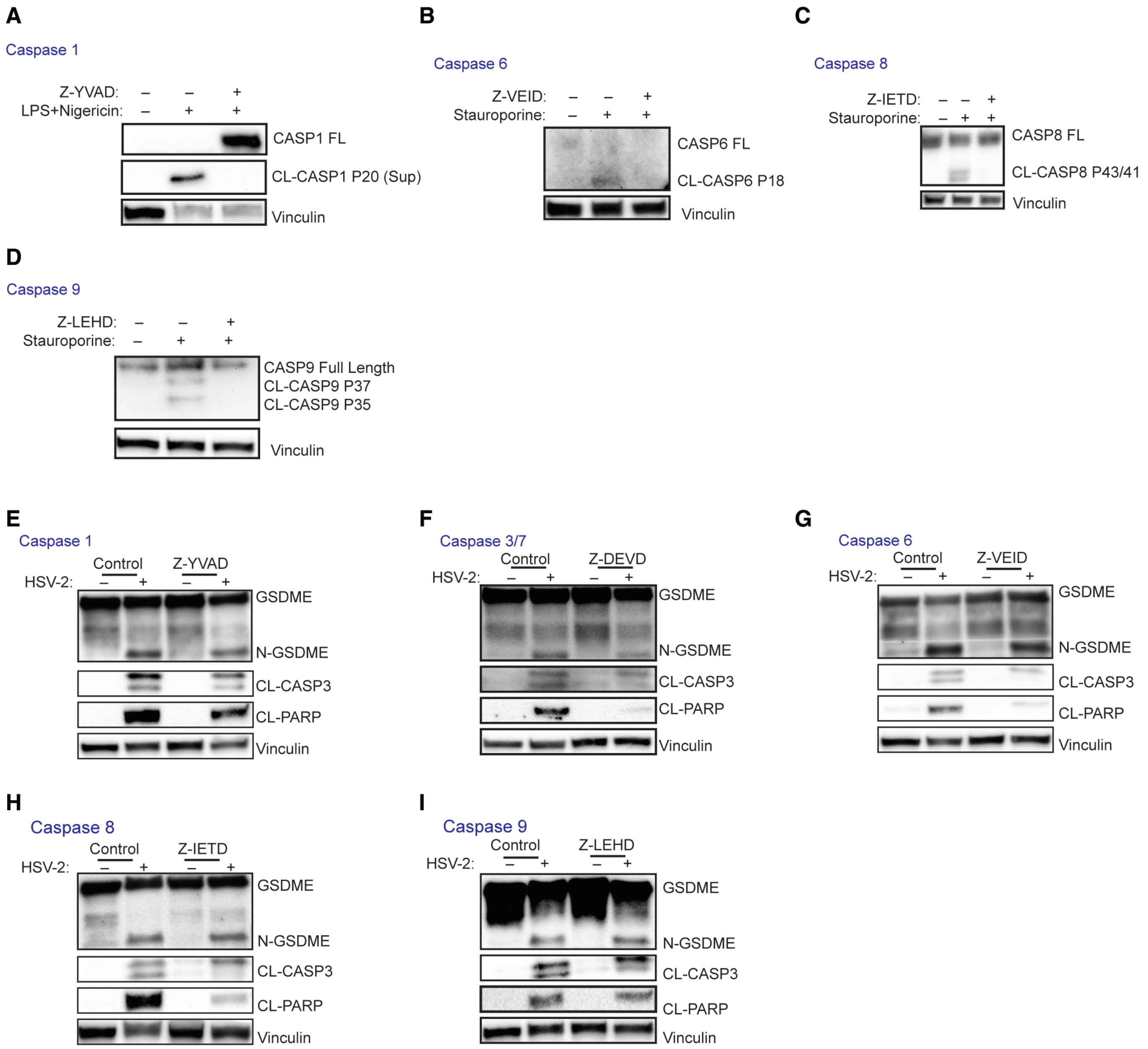

Figure EV2. Effect of inhibition of a panel of caspases on HSV-2-induced cleavage of GSDME.

A–D THP1 cells were pretreated with the indicated caspase inhibitors for 1 h and stimulated with different compounds as positive controls. LPS (1 μg/ml, 4 h) plus nigericin (10 μM, 1 h) were used to induce caspase 1 activity. Staurosporine (1 μM, 6 h) was used to activate caspase 3/6/7/8/9. (A) Z-YVAD, caspase 1 inhibitor; (B) Z-VEID, caspase 6 inhibitor; (C) Z-IETD, caspase 8 inhibitor; (D) Z-LEHD, caspase 9 inhibitor.

E–I SH-SY5Y cells were pretreated with various caspase inhibitors and infected with HSV-2 (MOI =1) for 16 h. Lysate was immunoblotted for CL-CASP3, CL-PARP, and GSDME. (E)Z-YVAD, caspase 1 inhibitor; (F) Z-DEVD, caspase 3/7 inhibitor; (G) Z-VEID, caspase 6 inhibitor; (H) Z-IETD, caspase 8 inhibitor; (I) Z-LEHD, caspase 9 inhibitor.

Source data are available online for this figure.

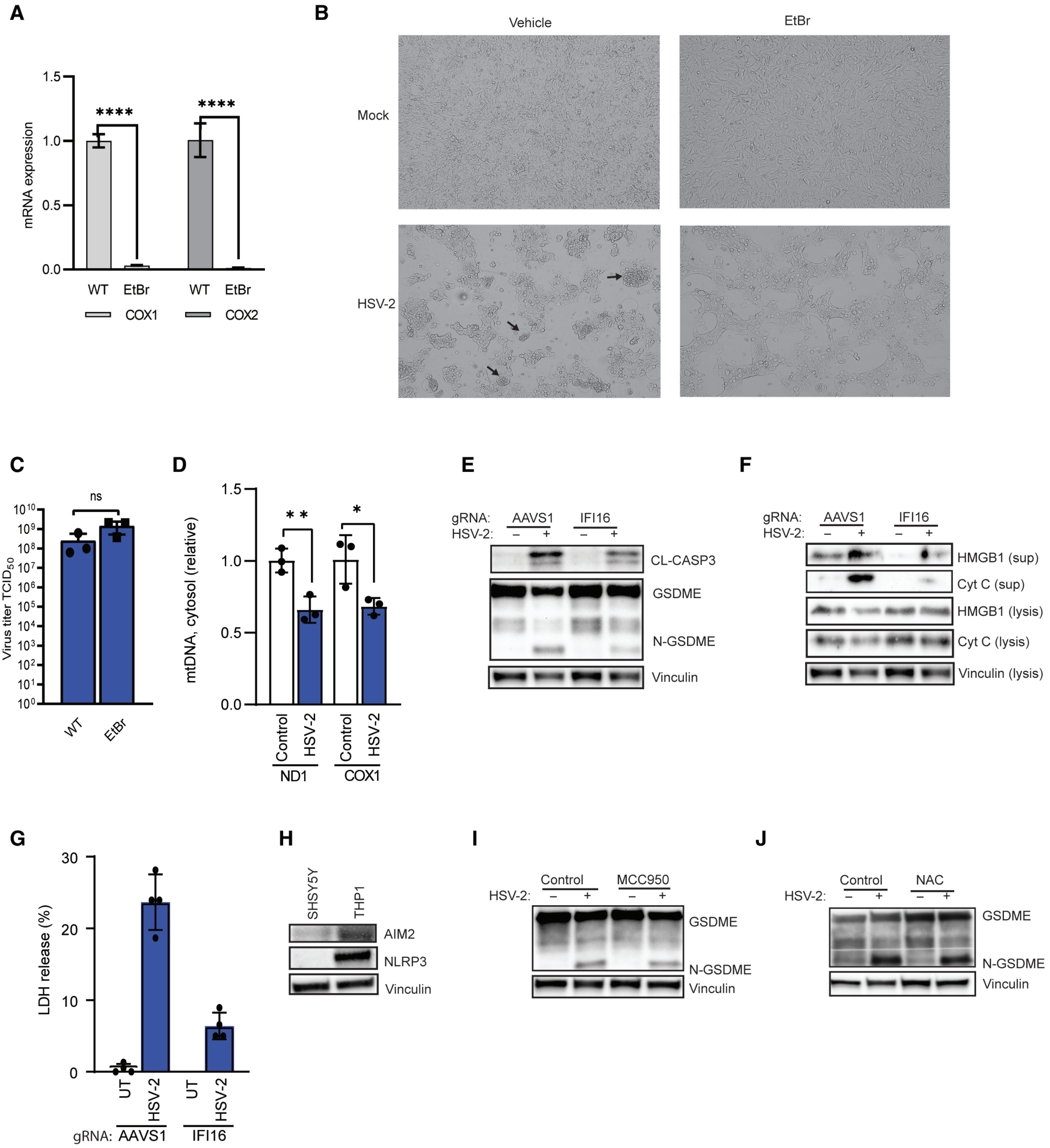

Figure EV3.

 © 2023 The Authors

Figure EV3. HSV-induced pyroptosis is dependent on mitochondrial DNA and cytosolic DNA sensing.

A–C SH-SY5Y cells were treated with ethidium bromide treatment (EtBr, 2 μg/ml) for 1 month, and medium was changed every 3 days. mtDNA was quantified by mtDNA by PCR amplification of COX 1 and COX 2 DNA (A). SH-SY5Y cells treated with ethidium bromide treatment were infected with HSV-2 (MOI = 1) for 16 h, and inspected for morphological changes by microscopy. Arrows indicate ballooning or syncytial cells (B). HSV-2 titer in supernatants 24 h post-infection (MOI = 1) (C).

D SH-SY5Y cells were infected with HSV-1 (MOI = 1) for 16 h, and mtDNA release into cytoplasm was quantified by PCR amplification of mtDNA ND1 and COX 1 from DNA isolated from cleared cytosolic fractions.

E–G SH-SY5Y cells treated with AAVS1 or IFI16-targeting gRNA-Cas9 RNP complexes were infected with HSV-2 (MOI = 1) for 16 h and 24 h. Cell lysate and supernatant (16 h with HSV-2 infection) were immunoblotted for GSDME and HMGB1 (E, F), and LDH release assay was performed on supernatants (24 h post-infection, G).

H Lysates from SH-SY5Y cells immunoblotted for AIM2, NLRP3, and vinculin.

I, J SH-SY5Y cells were pretreated with MCC950 or NAC and infected with HSV-2 for 16 h. Lysates were immunoblotted for GSDME and vinculin

Data information: All data shown are representative of at least three independent experiments. Data are presented as mean ± s.d. in all graphs. *$P \leq 0.05$; **$P \leq 0.01$; ****$P \leq 0.0001$ (Mann–Whitney test, two-tailed in A, C, and D, two-way ANOVA in G).
Source data are available online for this figure.

Figure EV4. HSV-2-induced ER stress occurs upstream of pyroptosis.

A SH-SY5Y cells were treated with etoposide (50 μM, 16 h) and thapsigargin (TG, 20 μM) for 16 h. Lysates were immunoblotted for p-eIF2α, tBID, and vinculin.

B SH-SY5Y cells were pretreated with CHX (10 μg/ml) for 1 h and infected with HSV-2 (MOI = 1, 16 h). Lysate was immunoblotted for MCL-1 and vinculin.

C–E (C, E) SH-SY5Y cells were treated with TG (20 μM) and CHX (10 ng/ml) for 24 h. NADPH reductase activity was measured to evaluate mitochondria function. (D) SH-SY5Y transfected with AAVS1 or BID gRNA-Cas9 RNPs were treated with TG (10 μM) for 16 h. Lysates were immunoblotted for GSDME, BID, and vinculin.

F SH-SY5Y cells were infected with HSV-2 in the indicated time intervals or treated with TG (20 μM, 16 h) or CHX (10 ng/ml, 24 h). Coomassie blue staining of SDS–PAGE of protein lysates was used to evaluate protein levels.

G SH-SY5Y cells were pretreated with TUDCA (500 ng/ml) for 1 h and infected with HSV-2 (MOI = 1, 16 h). Lysate was immunoblotted for p-IRE1α, p-JNK, and vinculin.

H SH-SY5Y cells were pretreated with TUDCA (500 ng/ml) for 1 h and infected with HSV-2 (MOI = 1, 16 h). Lysate was assayed for CASP3 activity.

I SH-SY5Y cells were pretreated with TUDCA (500 ng/ml, 1 h) and infected with HSV-2 (MOI = 1) for 16 h, and inspected for morphological changes by microscopy. Arrows indicate ballooning or syncytial cells

Data information: All data shown are representative of at least three independent experiments. Data are presented as mean ± s.d. in all graphs. ****$P \leq 0.0001$ (Mann–Whitney test, two-tailed in C and E, two-way ANOVA in H).
Source data are available online for this figure.

© 2023 The Authors

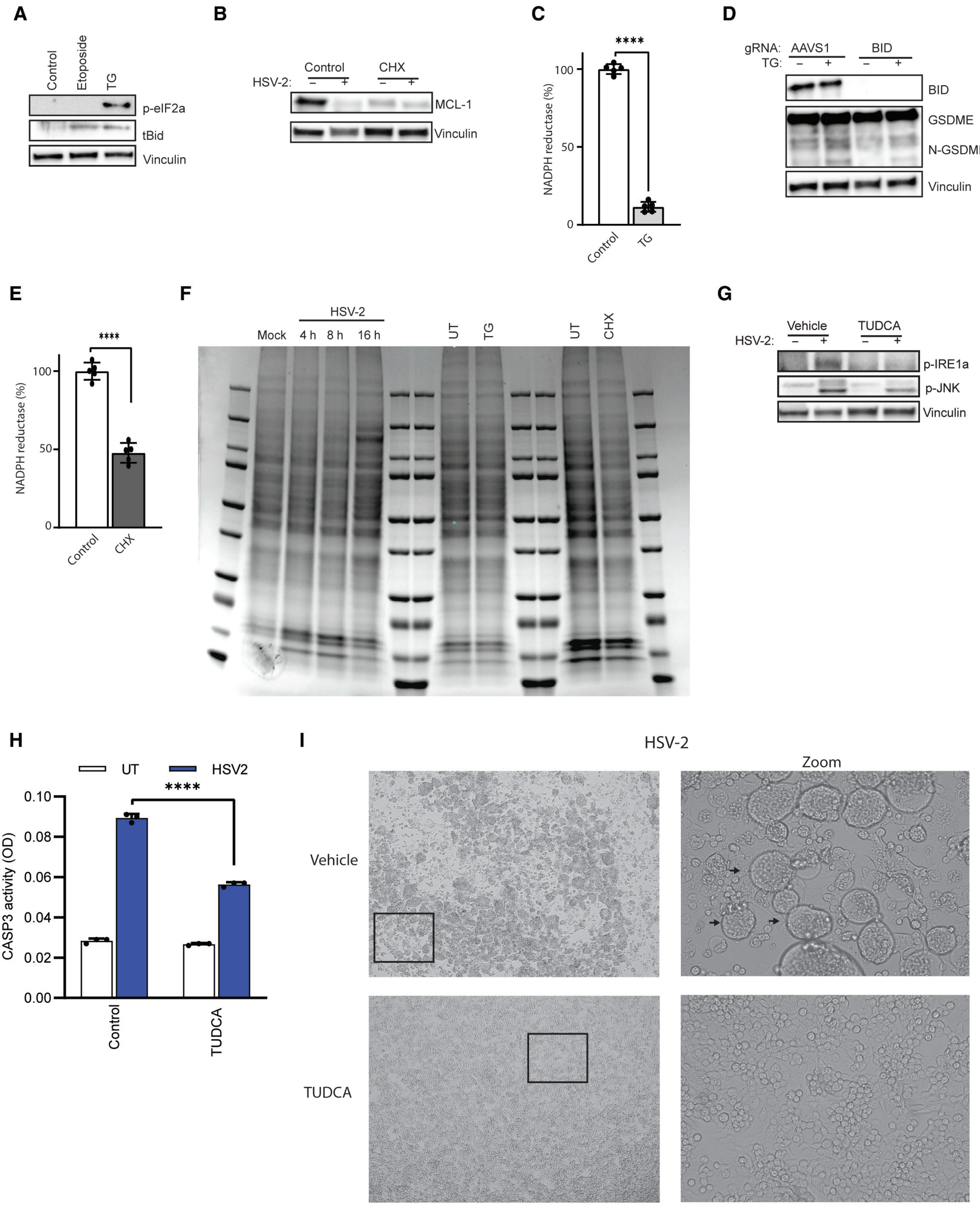

Figure EV4.

 © 2023 The Authors

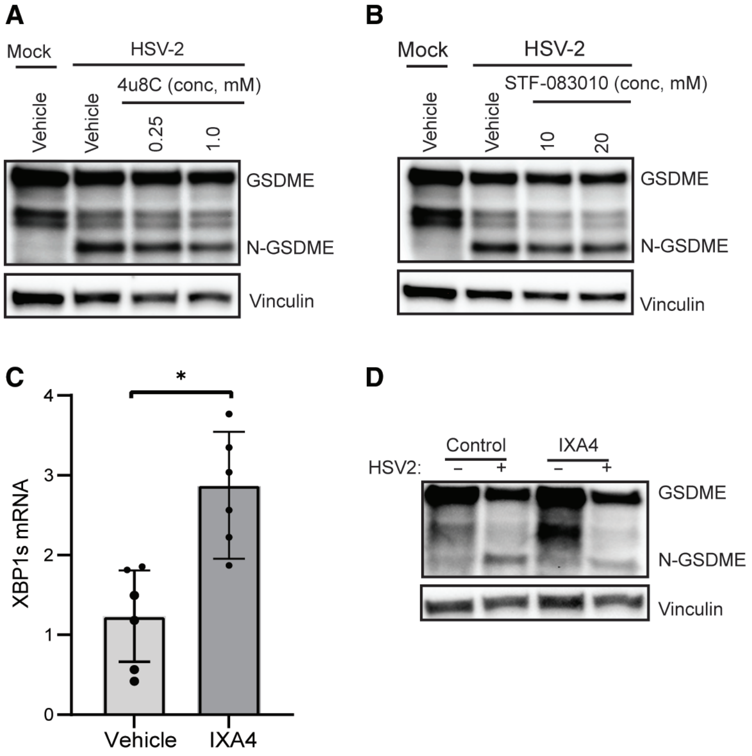

Figure EV5. ER stress-induced IRE1-α activation contributes to HSV-2-induced pyroptosis.

A, B SH-SY5Y cells were pretreated with 4u8c or STF-083010 for 1 h in the indicated concentrations and infected with HSV-2 (16 h). Lysate was immunoblotted for GSDME and vinculin.

C SH-SY5Y cells were pretreated with IXA4 for 1 h and infected with HSV-2 for 16 h, and RNA was isolated to quantify XBP1s mRNA by PCR.

D SH-SY5Y cells were pretreated with IXA4 (10 μM) for 1 h and infected with HSV-2 (MOI = 1, 16 h). Lysates were immunoblotted for GSDME and vinculin.

Data information: All data shown are representative of at least three independent experiments. Data are in (C) presented as mean ± s.d. in all graphs. *$P \leq 0.05$ (Mann–Whitney test, two-tailed). Source data are available online for this figure.

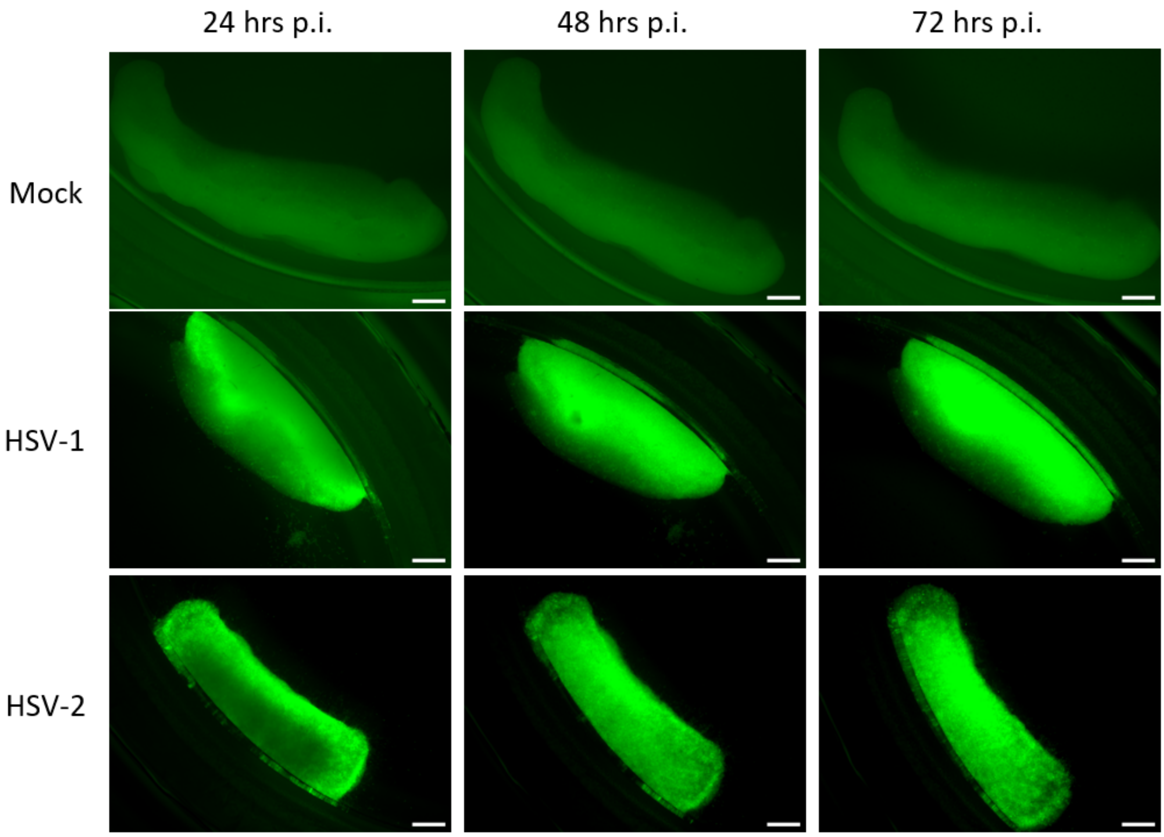

Appendix Figure S1. Replication of HSV-1 and 2 in hfOBSCs
hfOBSC were infected for 72 h with HSV-1 ($1{\times}10^6$ PFU/ml) and HSV-2 ($1{\times}10^6$ PFU/ml) expressing green fluoresce protein (GFP). The spread of HSV-1 and HSV-2 infection were initiated from one region of the brain tissue to throughout the whole brain slice. Images of the HSV infected brain slices were monitored with a fluorescent microscope (Zeiss AXIO inverted microscope, 2.5x objective) at indicated time points and reconstituted by using Fiji ImageJ software 1.53t. The data shown are representative for at least 3 independent experiments. Scale bar: 500 µm.

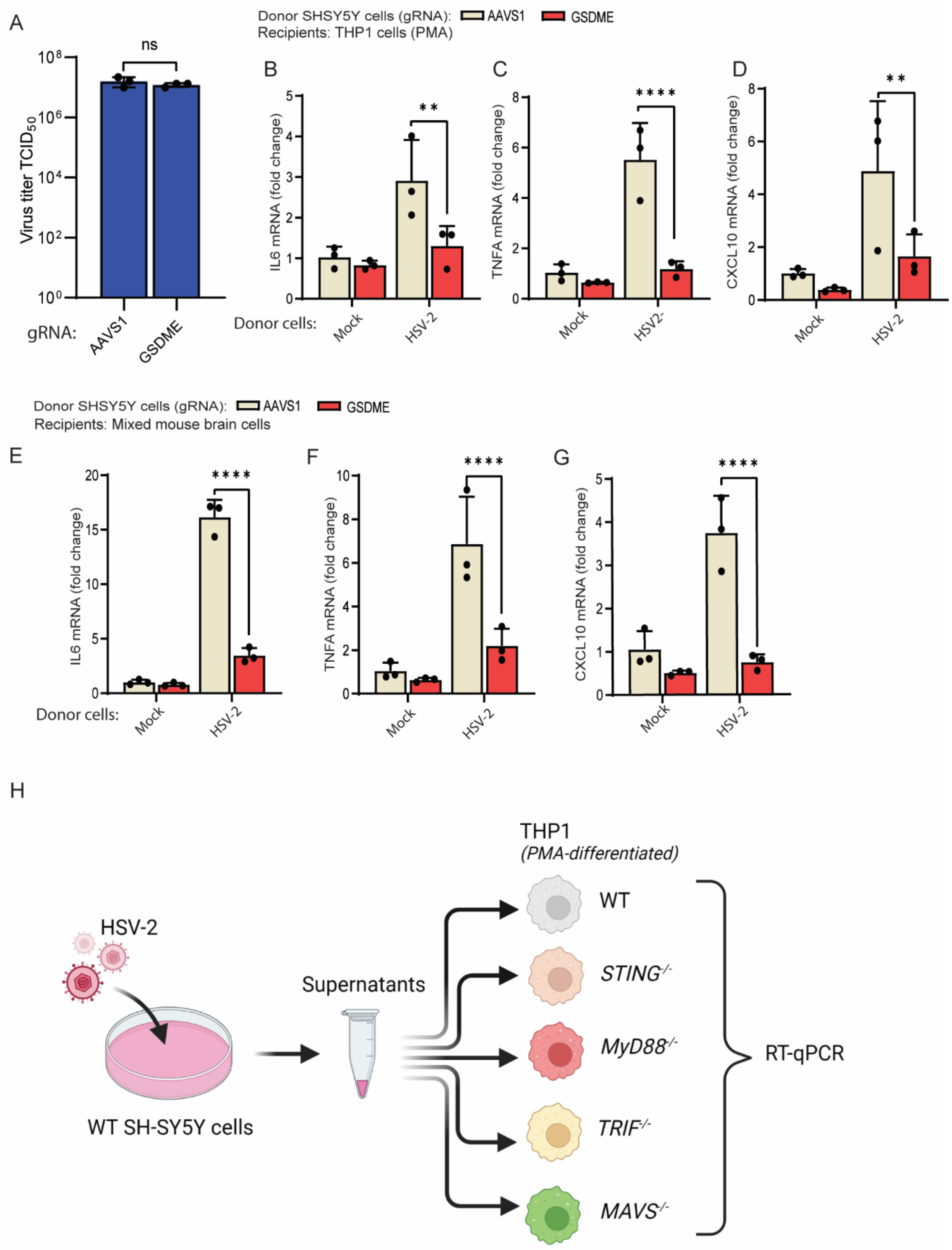

Appendix Figure S2. Necrotic neurons induce inflammatory gene signatures in microglia

(**A**) Supernatants from control and GSDME depleted SH-SY5Y cells infected with HSV-2 (MOI=1) for 24 h infection were subjected to virus titer assay.

(**B-D**) THP1 cells were treated for 8 h with conditioned medium from HSV-2-infected control and GSDME-depleted SH-SY5Y cells. Total RNA was isolated and analyzed for IL6, TNFA, and CXCL10 by RT-qPCR.

(**E-G**) Mixed mouse brain cells were treated with conditioned medium from HSV-2-infected control and GSDME-depleted SH-SY5Y cells for 8 h. Total RNA was isolated and analyzed for expression of Il6, Tnfa, and Cxcl10 by RT-qPCR.

(**H**) Illustration of the experimental setup to evaluate PRR pathways involved in the inflammatory response evoked by pyroptotic HSV-2-infected SH-SY5Y cells. Image was generated by Biorender.

Data information: All data shown are representative of at least 3 independent experiments. Data are presented as mean ± s.d. in all graphs. $*p \leq 0.05$; $**p \leq 0.01$; $****p \leq 0.0001$ (Mann-Whitney test, two-tailed in A, two-way ANOVA in B-G).
