# Supplementary figures and images for: ER stress induces caspase‐2‐tBID‐GSDME‐dependent cell death in neurons lytically infected with herpes simplex virus type 2 (part 1 of 2)
Source: EMBO J. 2023 Aug 30;42(19):e113118. doi: 10.15252/embj.2022113118 (PMC10548179; doi:10.15252/embj.2022113118)

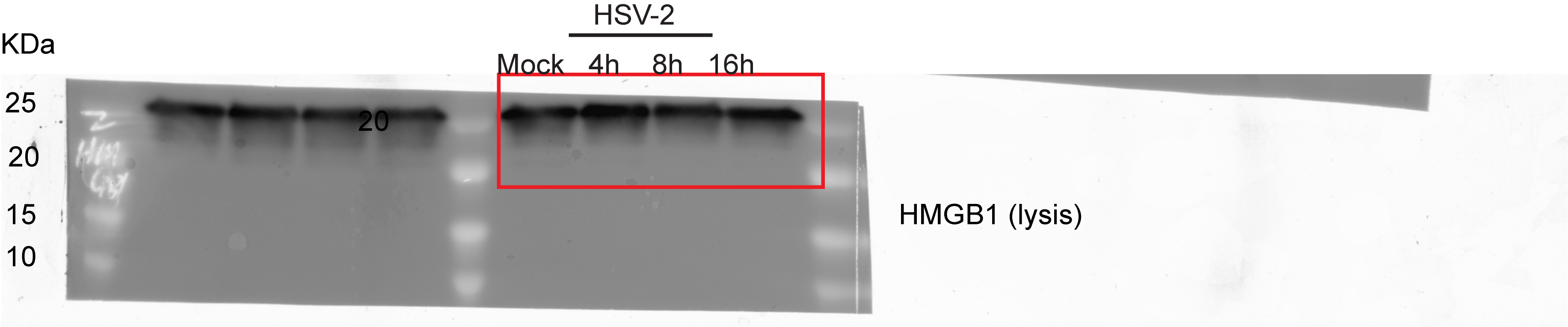

Supplement: Supplementary file 6 — Source Data for Figure 1 [file EMBJ-42-e113118-s001.zip › Source data Figure 1/1C/Western Blot HMGB1 (lysis).tif]

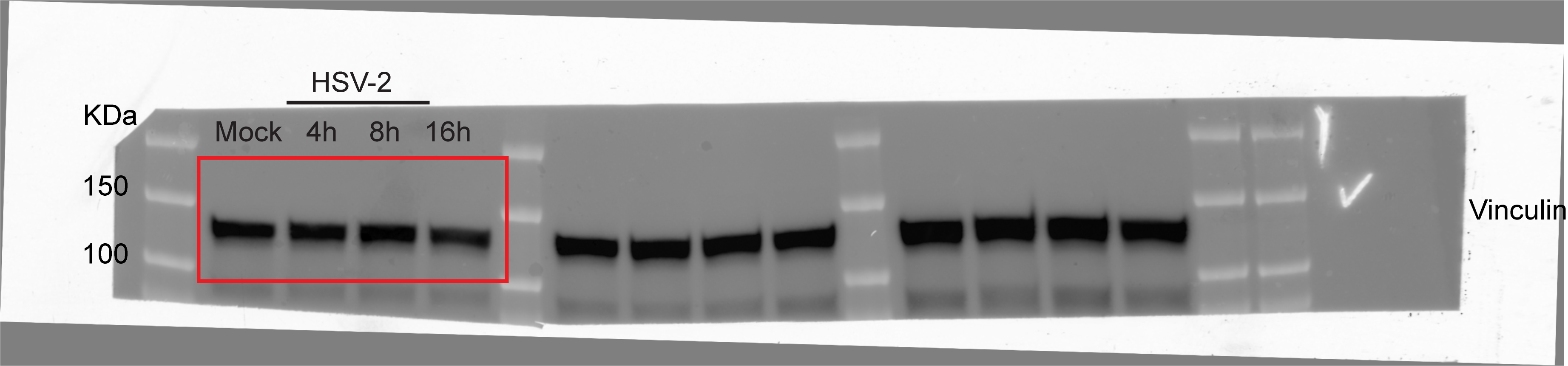

Supplement: Supplementary file 6 — Source Data for Figure 1 [file EMBJ-42-e113118-s001.zip › Source data Figure 1/1C/Western Blot Vinculin.tif]

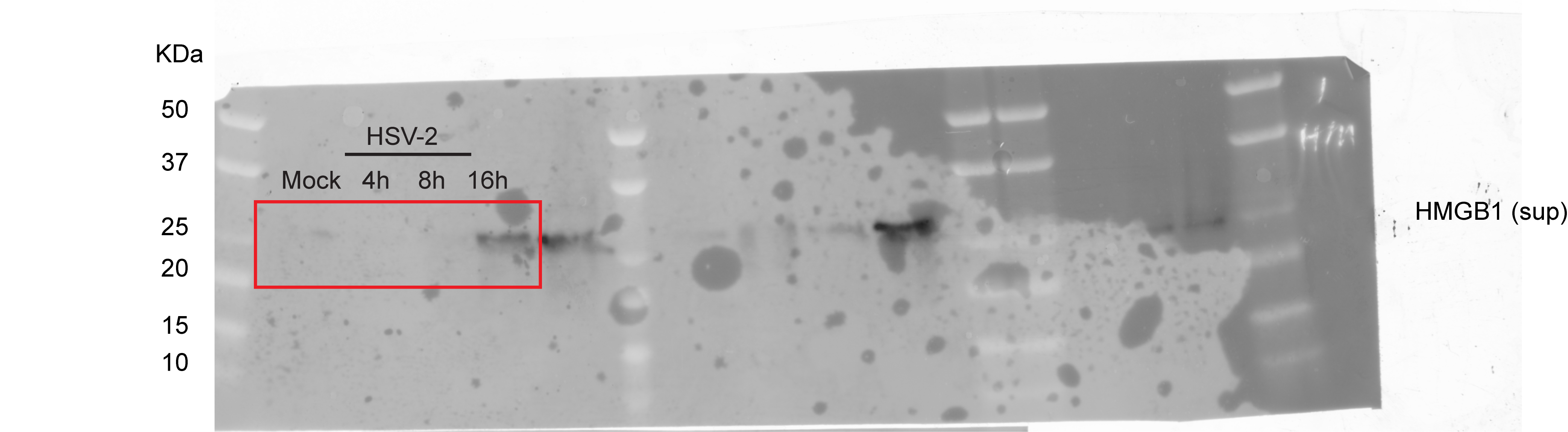

Supplement: Supplementary file 6 — Source Data for Figure 1 [file EMBJ-42-e113118-s001.zip › Source data Figure 1/1C/Western Blot HMGB1 (sup).tif]

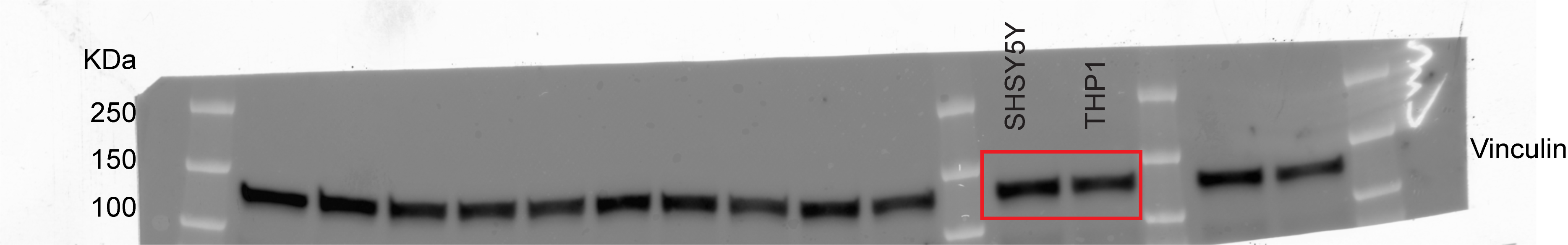

Supplement: Supplementary file 6 — Source Data for Figure 1 [file EMBJ-42-e113118-s001.zip › Source data Figure 1/1D/Western Blot Vinculin.tif]

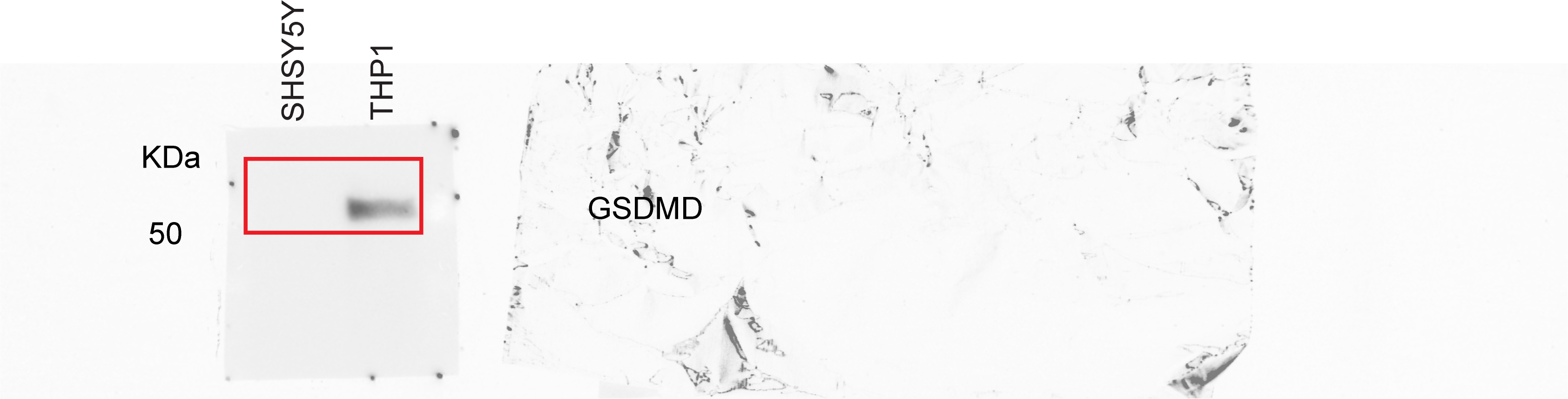

Supplement: Supplementary file 6 — Source Data for Figure 1 [file EMBJ-42-e113118-s001.zip › Source data Figure 1/1D/Western Blot GSDMD.tif]

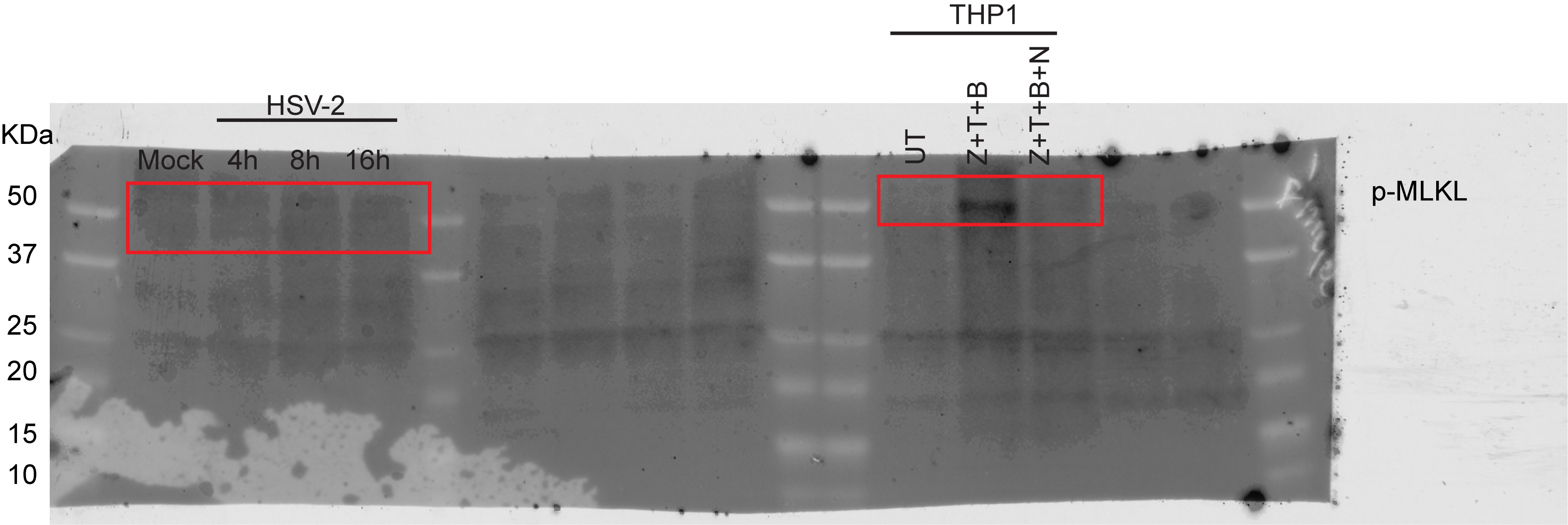

Supplement: Supplementary file 6 — Source Data for Figure 1 [file EMBJ-42-e113118-s001.zip › Source data Figure 1/1E/Western Blot p-MLKL.tif]

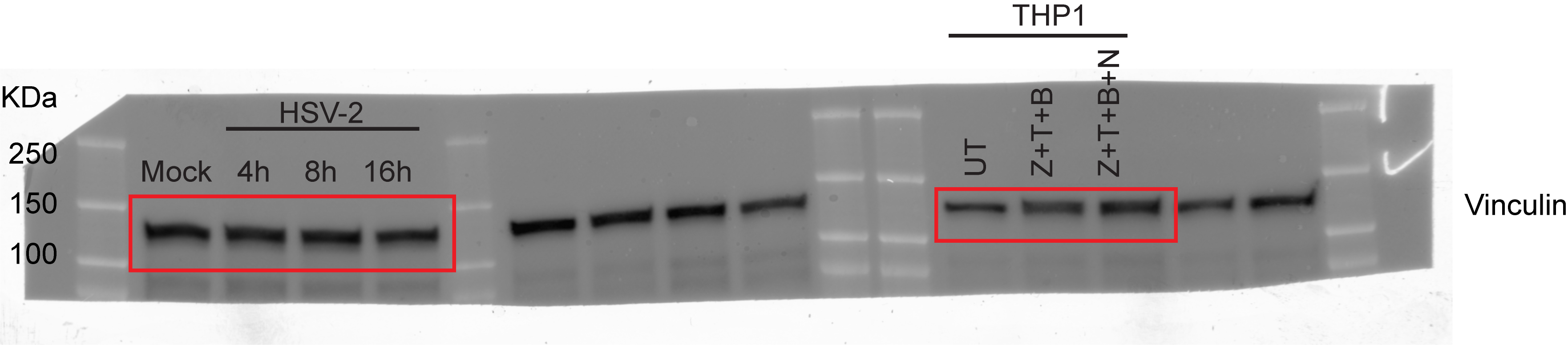

Supplement: Supplementary file 6 — Source Data for Figure 1 [file EMBJ-42-e113118-s001.zip › Source data Figure 1/1E/Western Blot Vinculin.tif]

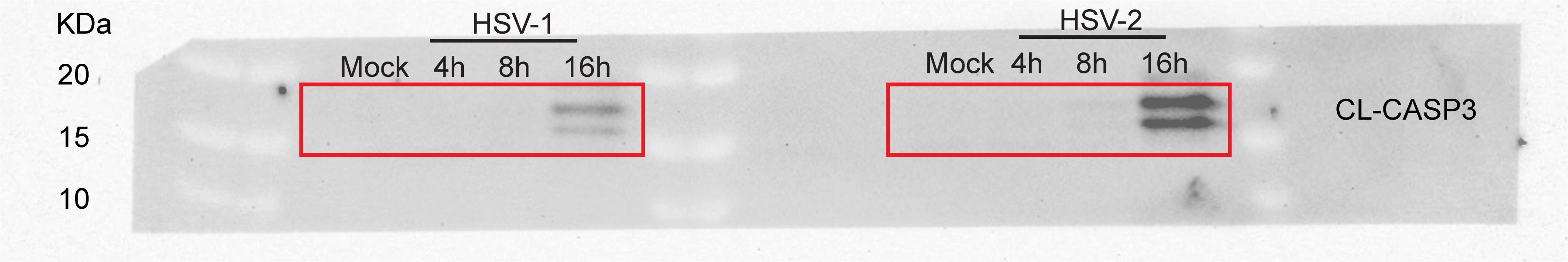

Supplement: Supplementary file 6 — Source Data for Figure 1 [file EMBJ-42-e113118-s001.zip › Source data Figure 1/1F/Western Blot CL-CASP3.tif]

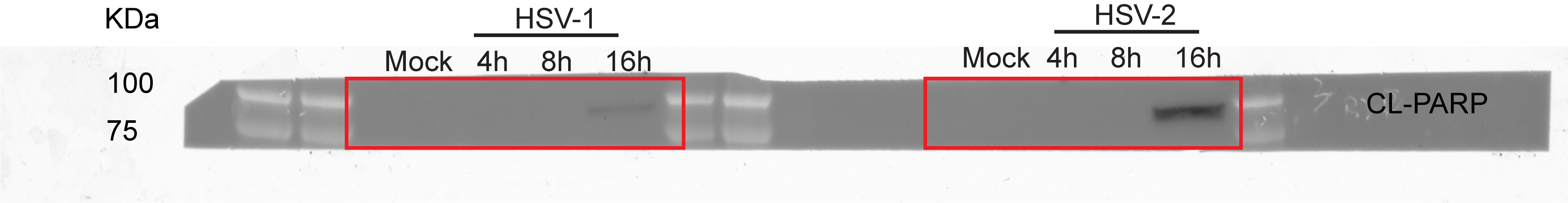

Supplement: Supplementary file 6 — Source Data for Figure 1 [file EMBJ-42-e113118-s001.zip › Source data Figure 1/1F/Western Blot CL-PARP.tif]

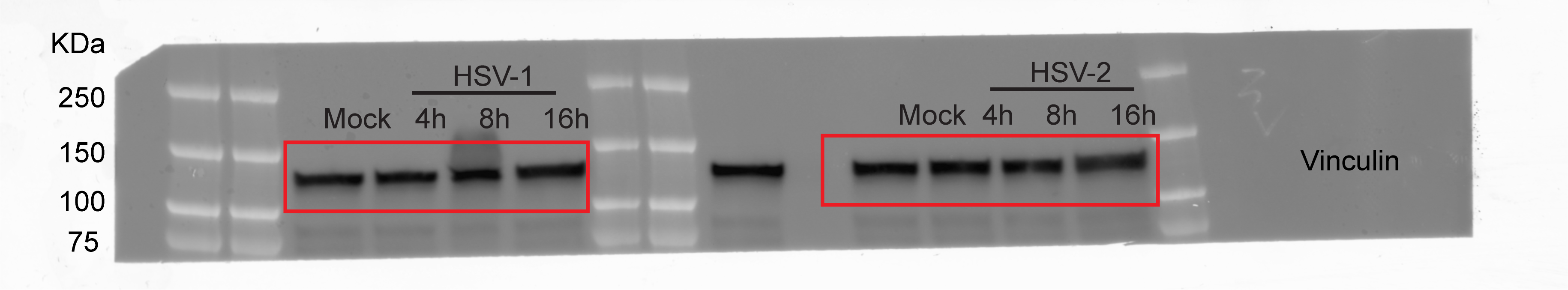

Supplement: Supplementary file 6 — Source Data for Figure 1 [file EMBJ-42-e113118-s001.zip › Source data Figure 1/1F/Western Blot Vinculin.tif]

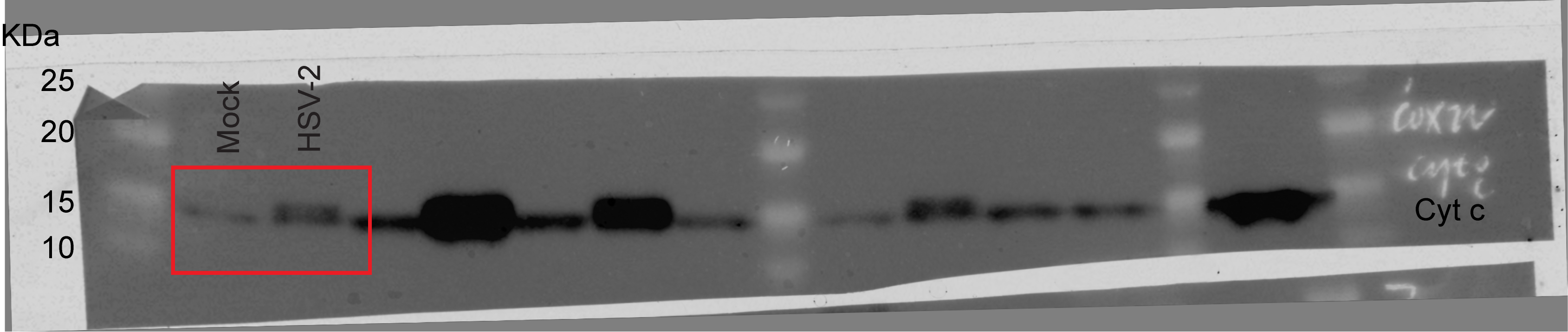

Supplement: Supplementary file 6 — Source Data for Figure 1 [file EMBJ-42-e113118-s001.zip › Source data Figure 1/1G/Western Blot Cyt c.tif]

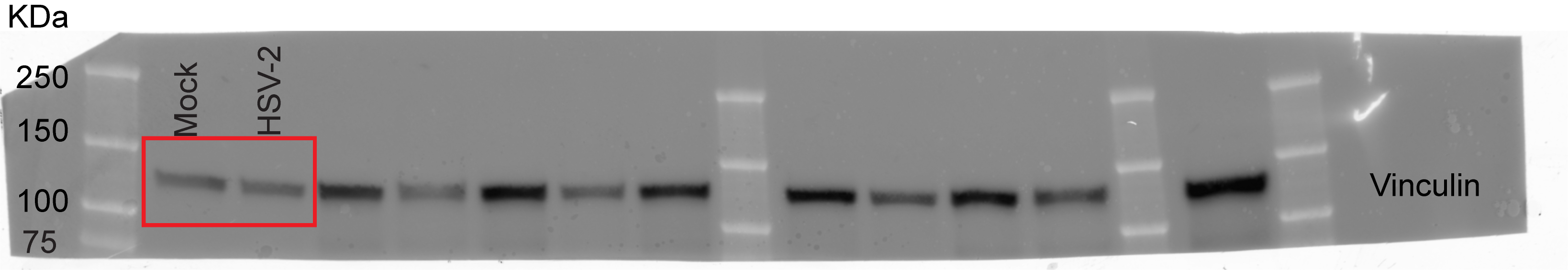

Supplement: Supplementary file 6 — Source Data for Figure 1 [file EMBJ-42-e113118-s001.zip › Source data Figure 1/1G/Western Blot Vinculin.tif]

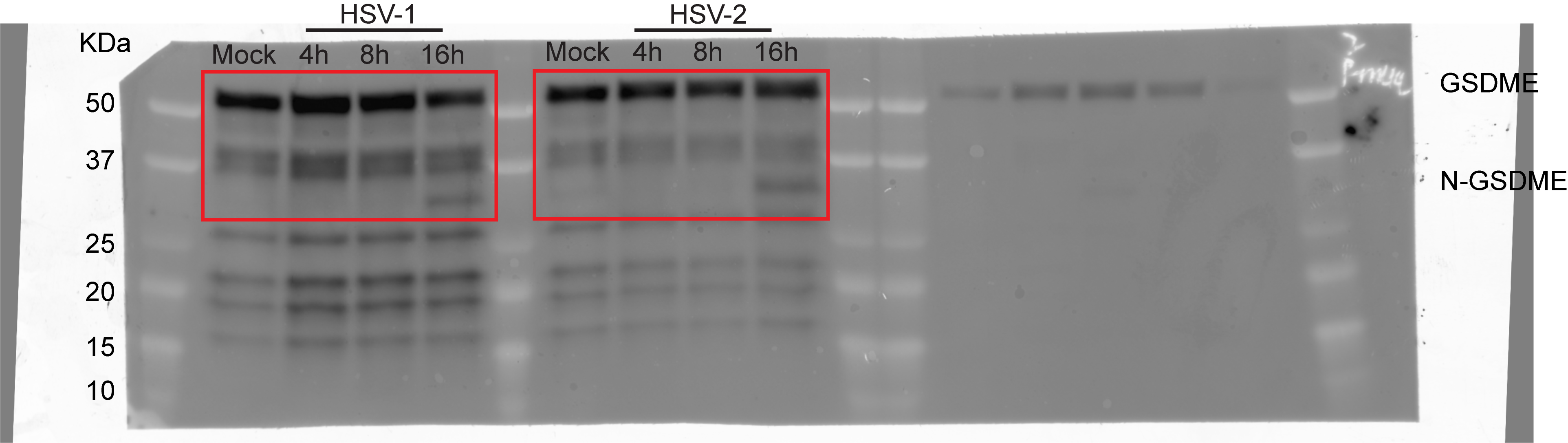

Supplement: Supplementary file 6 — Source Data for Figure 1 [file EMBJ-42-e113118-s001.zip › Source data Figure 1/1I and 1J/Western Blot GSDME.tif]

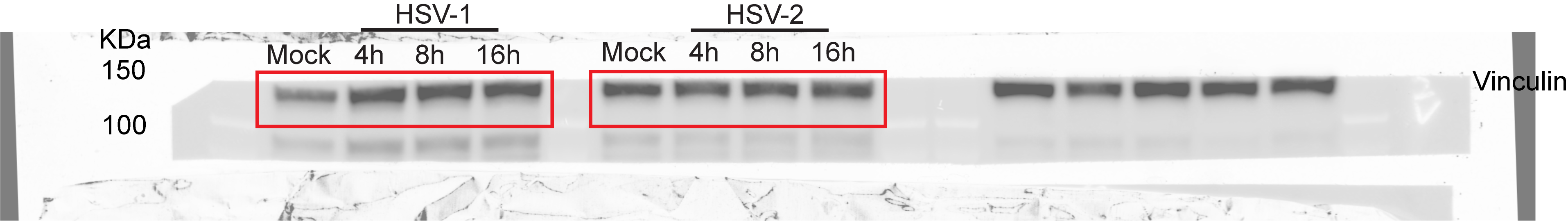

Supplement: Supplementary file 6 — Source Data for Figure 1 [file EMBJ-42-e113118-s001.zip › Source data Figure 1/1I and 1J/Western Blot Vinculin.tif]

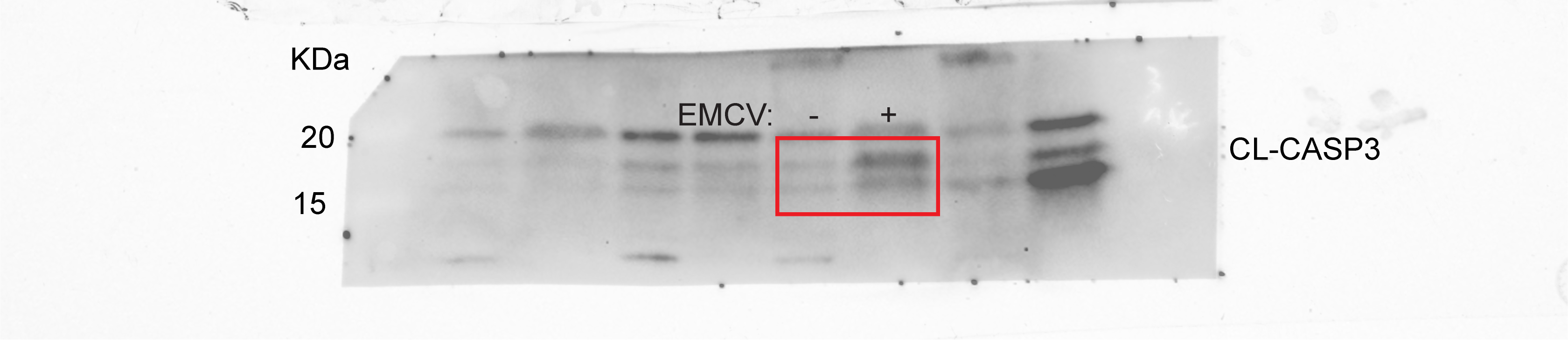

Supplement: Supplementary file 6 — Source Data for Figure 1 [file EMBJ-42-e113118-s001.zip › Source data Figure 1/1K and 1L/1K/1K/Western Blot CL-CASP3.tif]

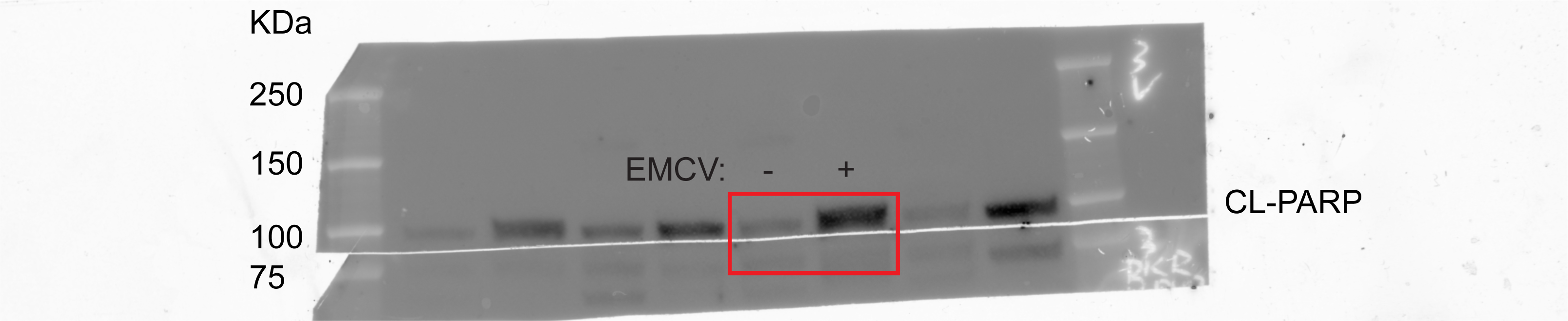

Supplement: Supplementary file 6 — Source Data for Figure 1 [file EMBJ-42-e113118-s001.zip › Source data Figure 1/1K and 1L/1K/1K/Western Blot CL-PARP.tif]

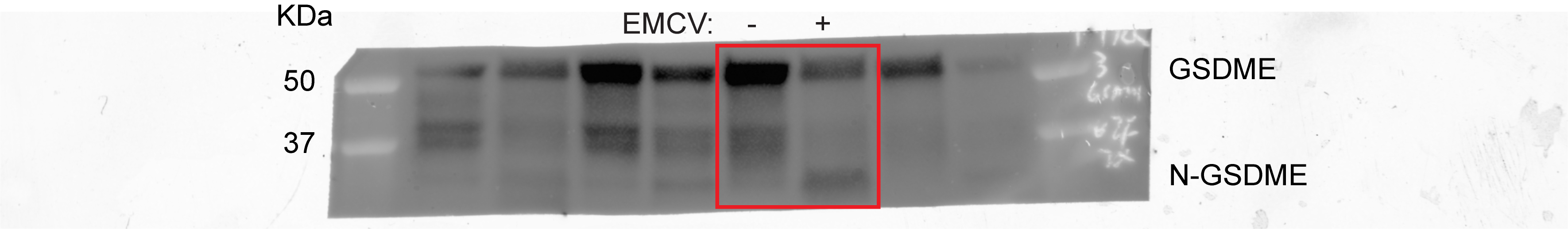

Supplement: Supplementary file 6 — Source Data for Figure 1 [file EMBJ-42-e113118-s001.zip › Source data Figure 1/1K and 1L/1K/1K/Western Blot GSDME.tif]

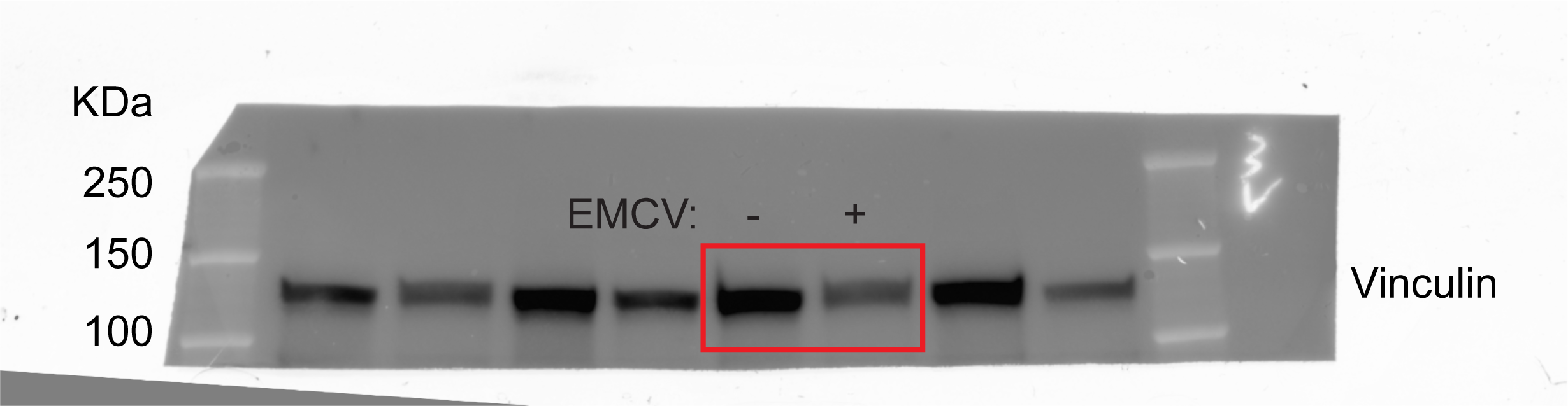

Supplement: Supplementary file 6 — Source Data for Figure 1 [file EMBJ-42-e113118-s001.zip › Source data Figure 1/1K and 1L/1K/1K/Western Blot Vinculin.tif]

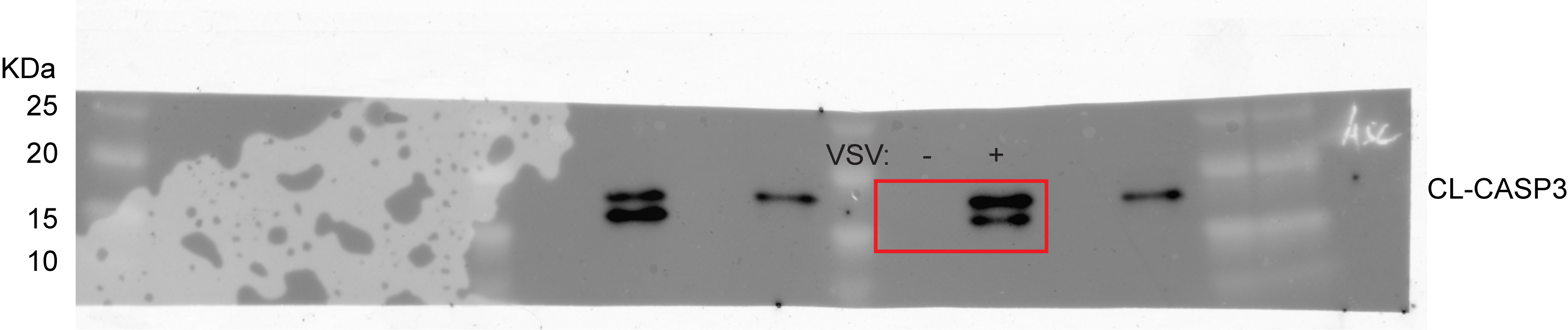

Supplement: Supplementary file 6 — Source Data for Figure 1 [file EMBJ-42-e113118-s001.zip › Source data Figure 1/1K and 1L/1L/1L/Western Blot CL-CASP3.tif]

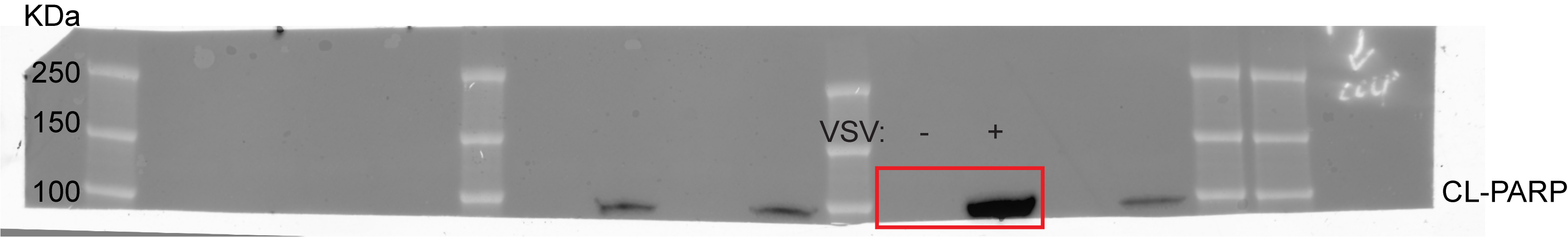

Supplement: Supplementary file 6 — Source Data for Figure 1 [file EMBJ-42-e113118-s001.zip › Source data Figure 1/1K and 1L/1L/1L/Western Blot CL-PARP.tif]

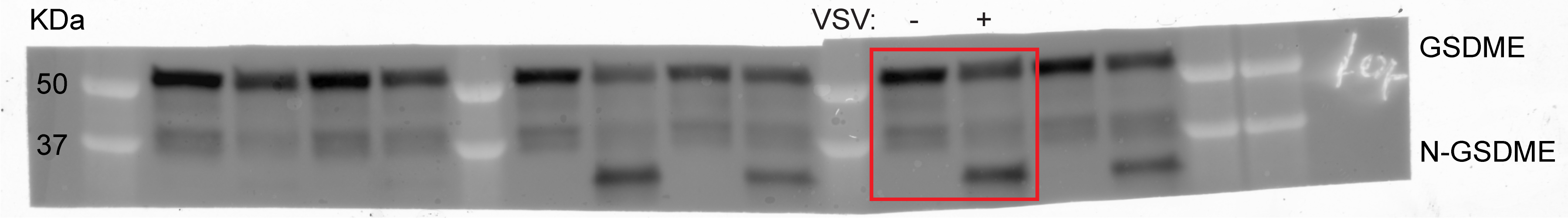

Supplement: Supplementary file 6 — Source Data for Figure 1 [file EMBJ-42-e113118-s001.zip › Source data Figure 1/1K and 1L/1L/1L/Western Blot GSDME.tif]

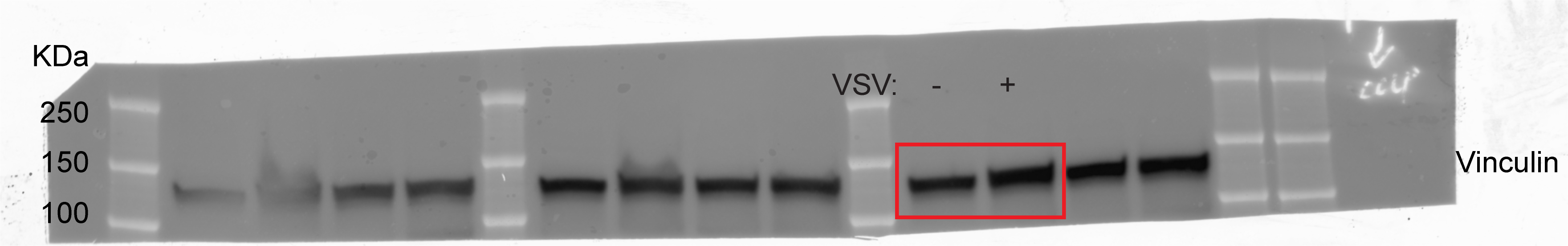

Supplement: Supplementary file 6 — Source Data for Figure 1 [file EMBJ-42-e113118-s001.zip › Source data Figure 1/1K and 1L/1L/1L/Western Blot Vinculin.tif]

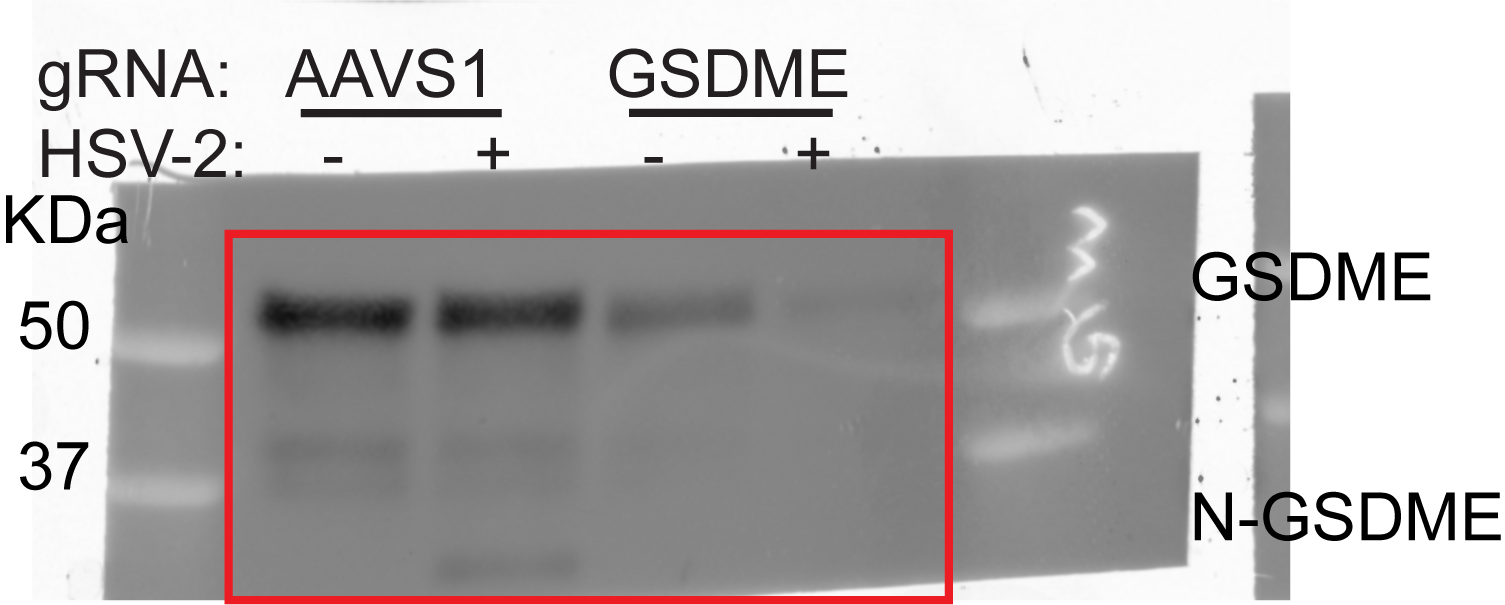

Supplement: Supplementary file 6 — Source Data for Figure 1 [file EMBJ-42-e113118-s001.zip › Source data Figure 1/1N/Western Blot GSDME.tif]

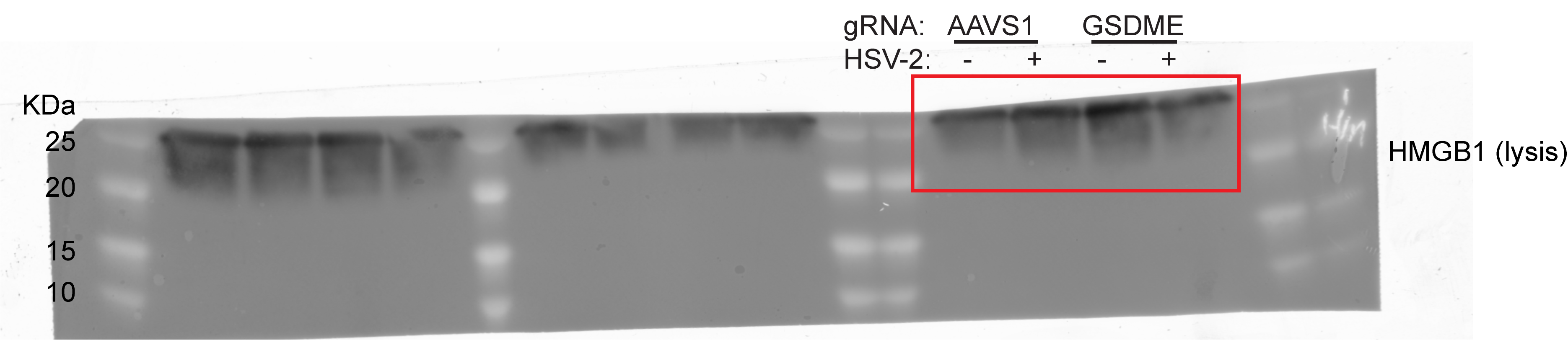

Supplement: Supplementary file 6 — Source Data for Figure 1 [file EMBJ-42-e113118-s001.zip › Source data Figure 1/1N/Western Blot HMGB1 (lysis).tif]

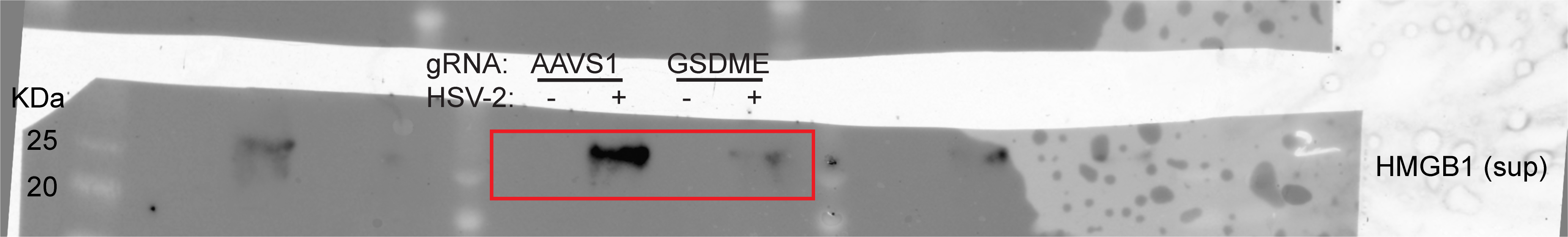

Supplement: Supplementary file 6 — Source Data for Figure 1 [file EMBJ-42-e113118-s001.zip › Source data Figure 1/1N/Western Blot HMGB1 (sup).tif]

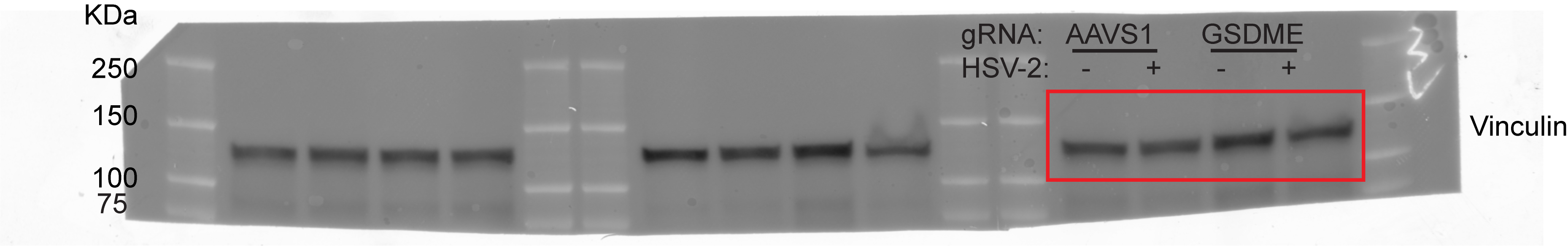

Supplement: Supplementary file 6 — Source Data for Figure 1 [file EMBJ-42-e113118-s001.zip › Source data Figure 1/1N/Western Blot Vinculin.tif]

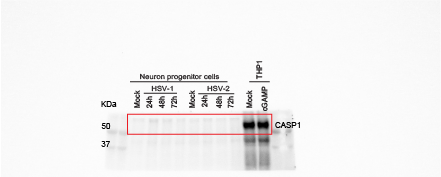

Supplement: Supplementary file 7 — Source Data for Figure 2 [file EMBJ-42-e113118-s011.zip › Source data Figure 2/2B/Western Blot CASP1.tif]

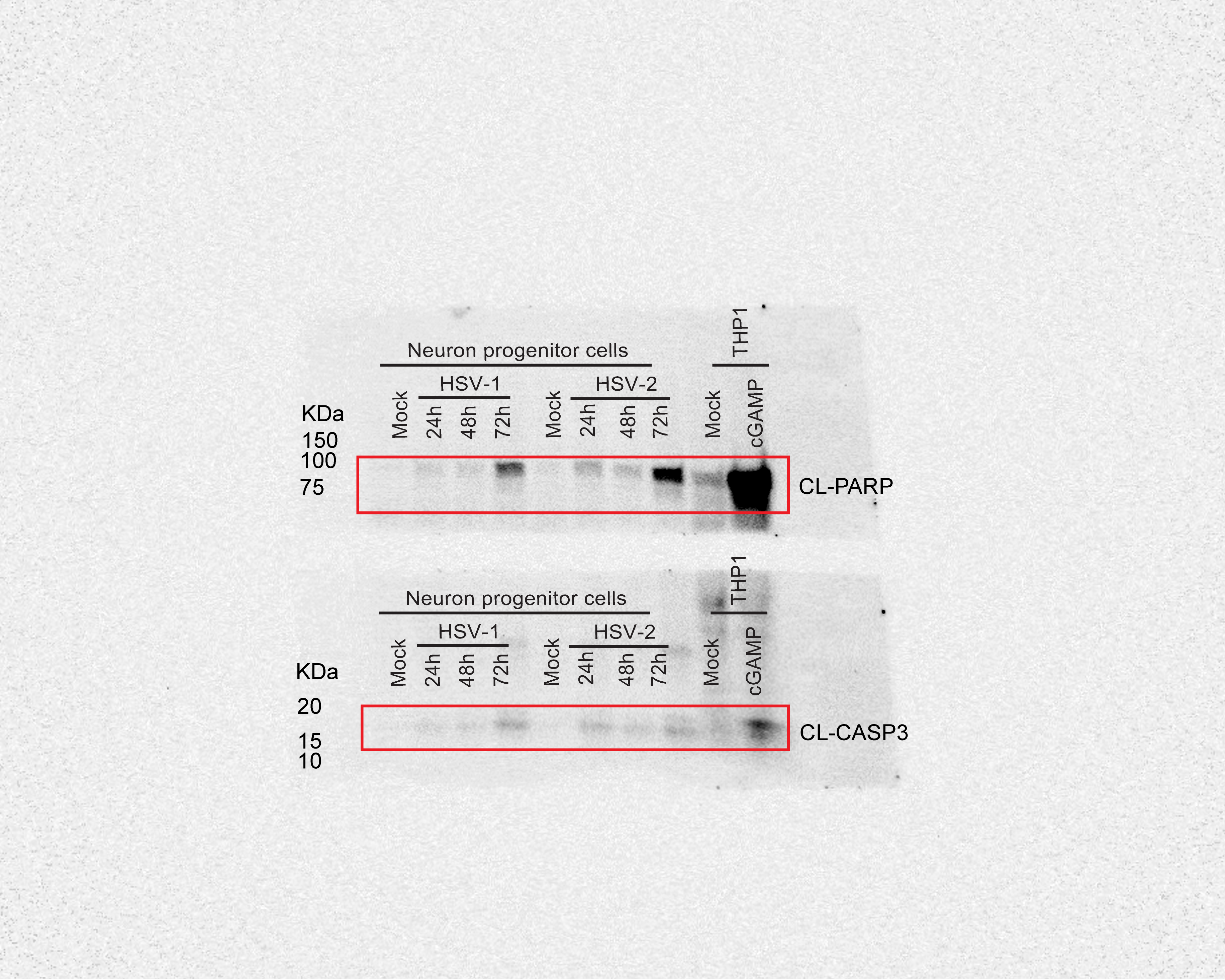

Supplement: Supplementary file 7 — Source Data for Figure 2 [file EMBJ-42-e113118-s011.zip › Source data Figure 2/2B/Western Blot CL-PARP and CL-CASP3.tif]

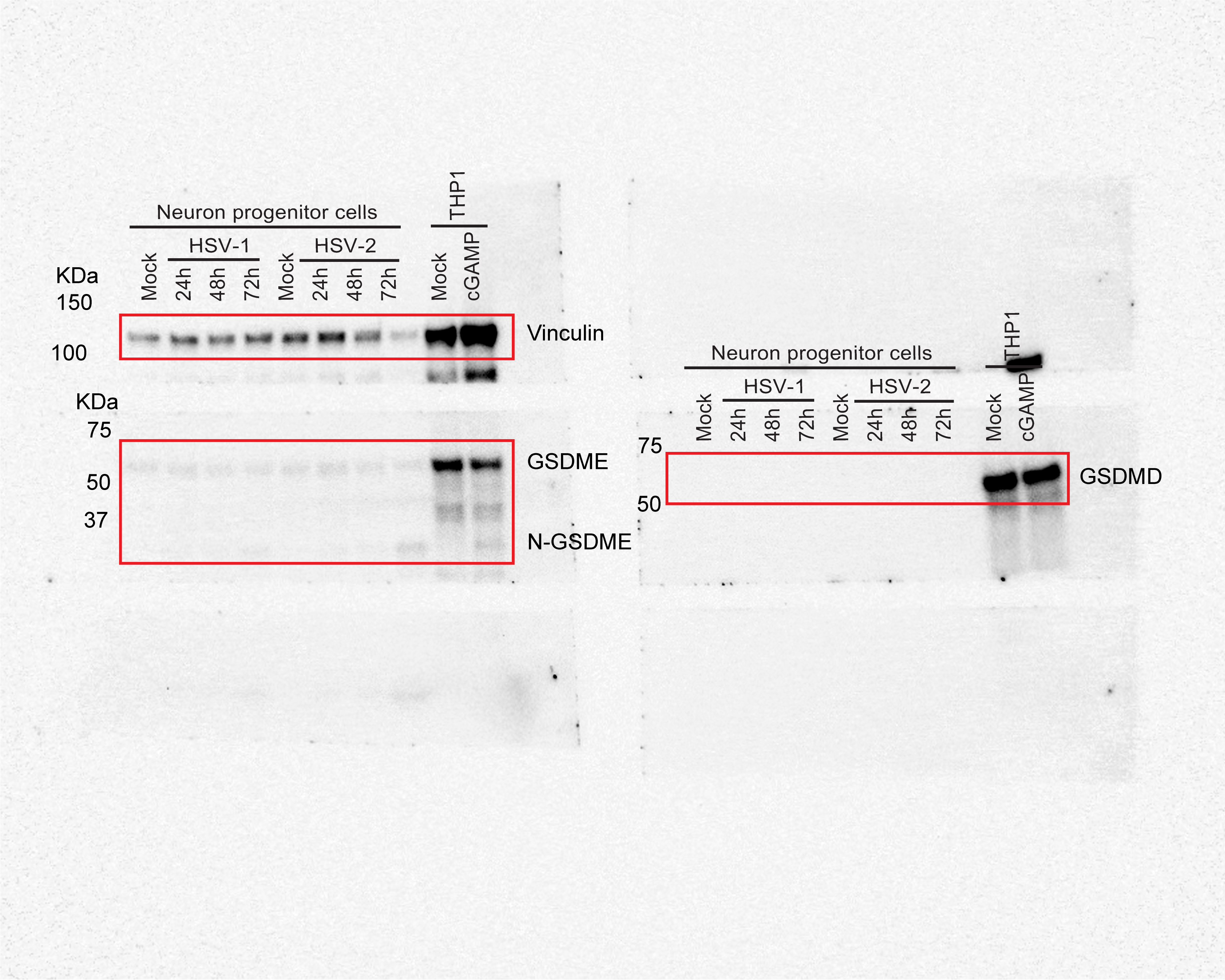

Supplement: Supplementary file 7 — Source Data for Figure 2 [file EMBJ-42-e113118-s011.zip › Source data Figure 2/2B/Western Blot Vinculin GSDME and GSDMD.tif]

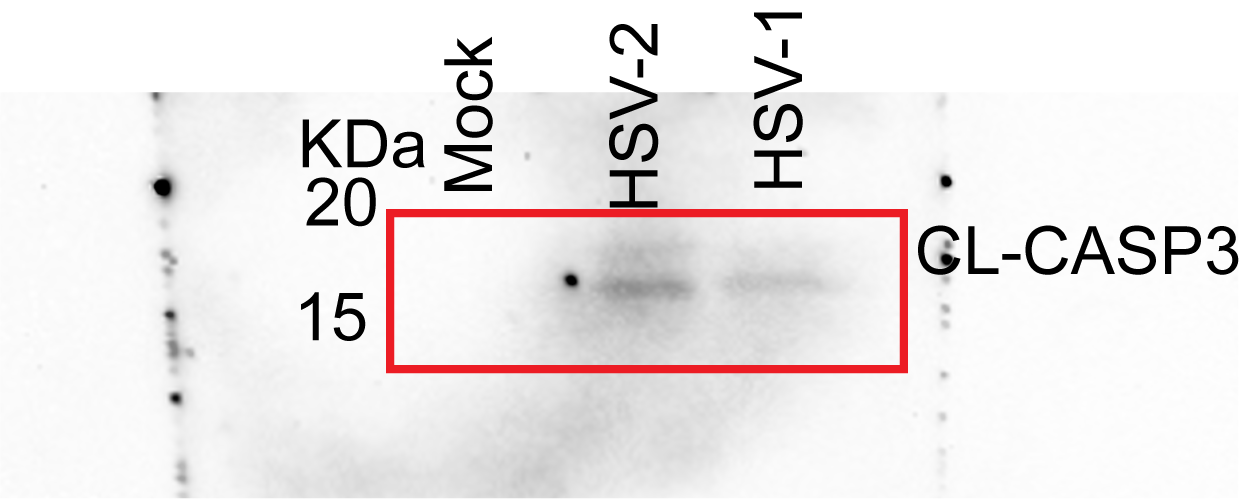

Supplement: Supplementary file 7 — Source Data for Figure 2 [file EMBJ-42-e113118-s011.zip › Source data Figure 2/2F/Western Blot CL-CASP3.tif]

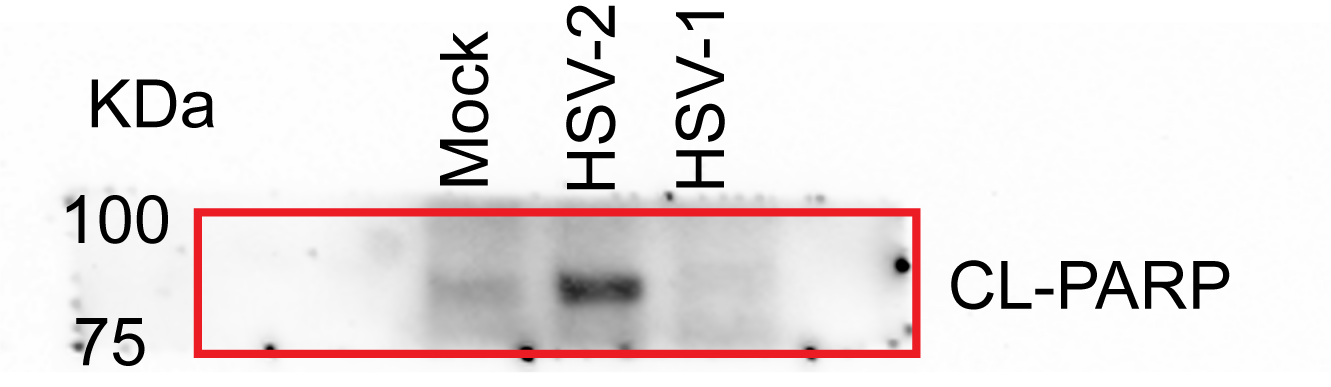

Supplement: Supplementary file 7 — Source Data for Figure 2 [file EMBJ-42-e113118-s011.zip › Source data Figure 2/2F/Western Blot CL-PARP.tif]

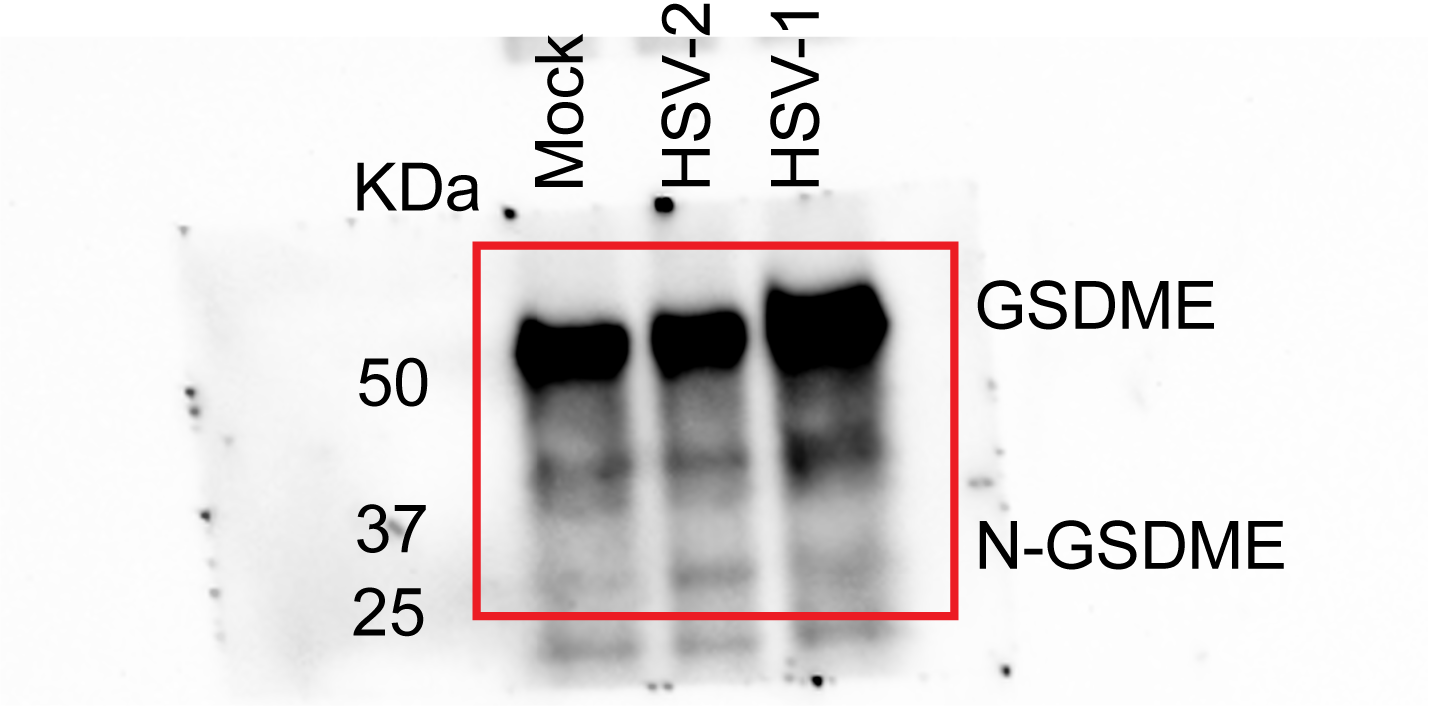

Supplement: Supplementary file 7 — Source Data for Figure 2 [file EMBJ-42-e113118-s011.zip › Source data Figure 2/2F/Western Blot GSDME.tif]

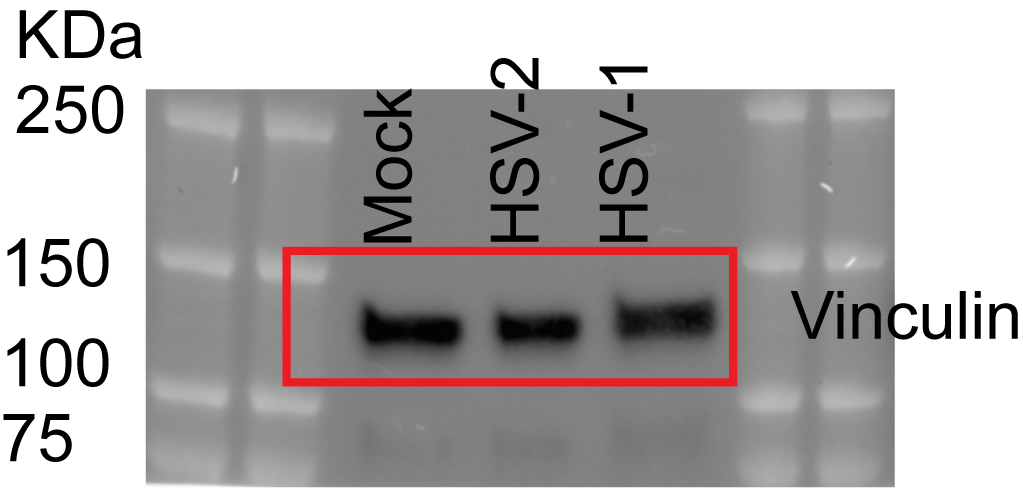

Supplement: Supplementary file 7 — Source Data for Figure 2 [file EMBJ-42-e113118-s011.zip › Source data Figure 2/2F/Western Blot Vinculin.tif]

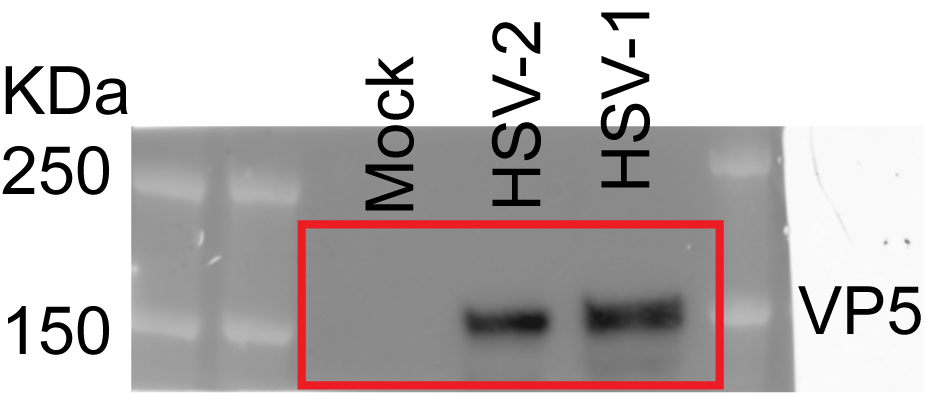

Supplement: Supplementary file 7 — Source Data for Figure 2 [file EMBJ-42-e113118-s011.zip › Source data Figure 2/2F/Western Blot VP5.tif]

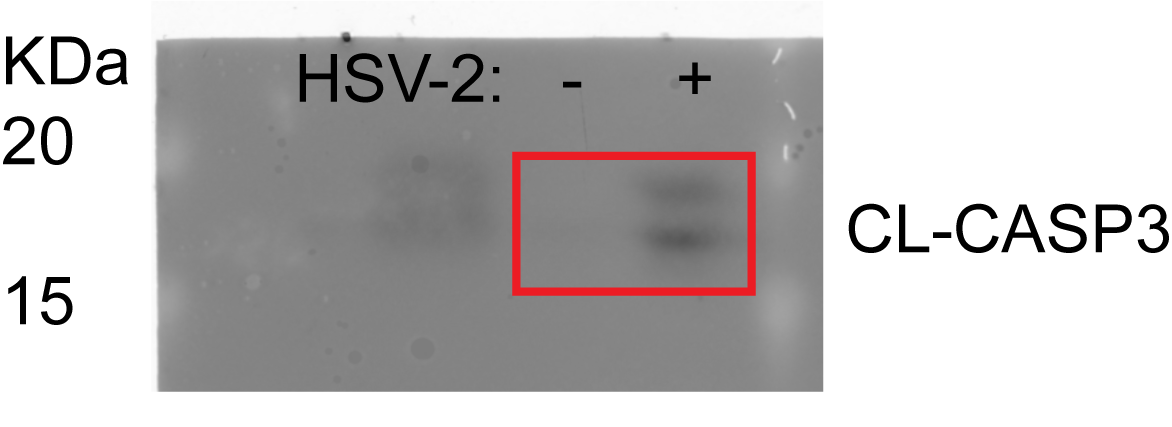

Supplement: Supplementary file 7 — Source Data for Figure 2 [file EMBJ-42-e113118-s011.zip › Source data Figure 2/2I/Western Blot CL-CASP3.tif]

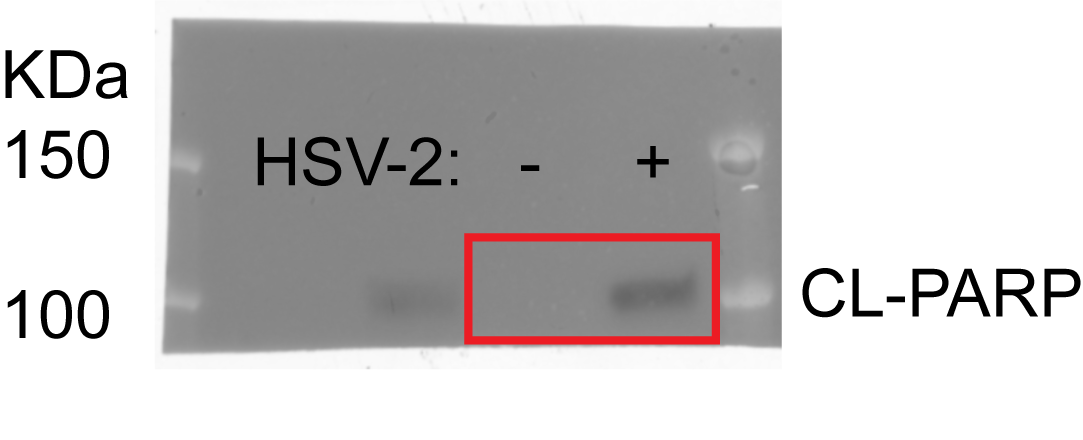

Supplement: Supplementary file 7 — Source Data for Figure 2 [file EMBJ-42-e113118-s011.zip › Source data Figure 2/2I/Western Blot CL-PARP.tif]

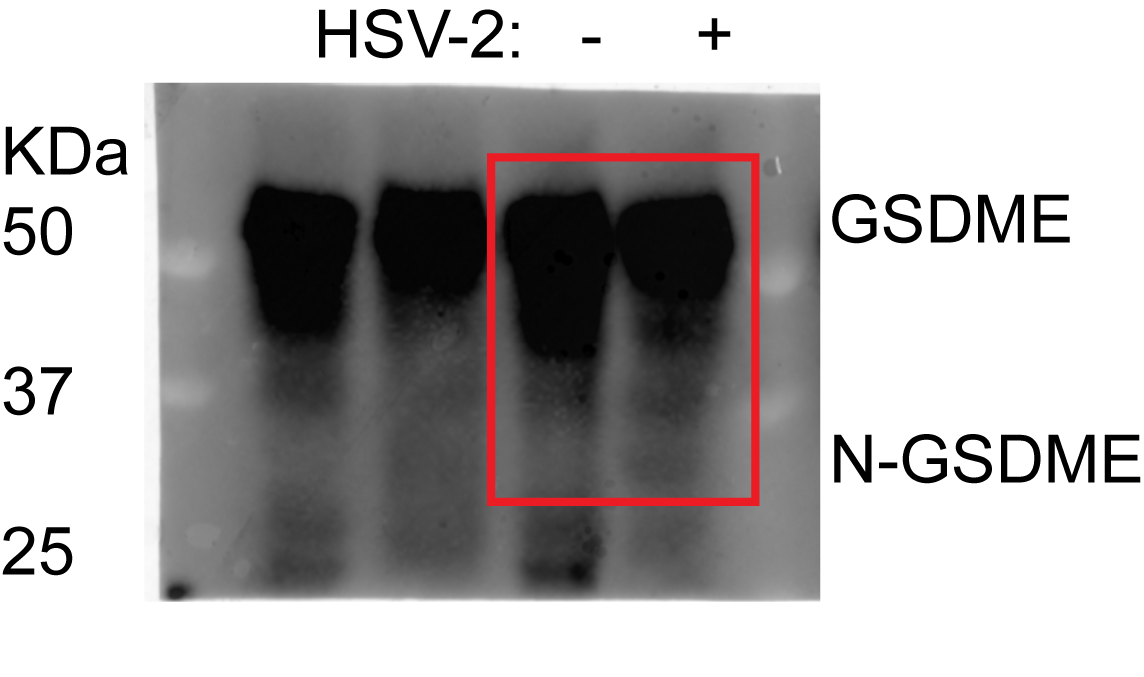

Supplement: Supplementary file 7 — Source Data for Figure 2 [file EMBJ-42-e113118-s011.zip › Source data Figure 2/2I/Western Blot GSDME.tif]

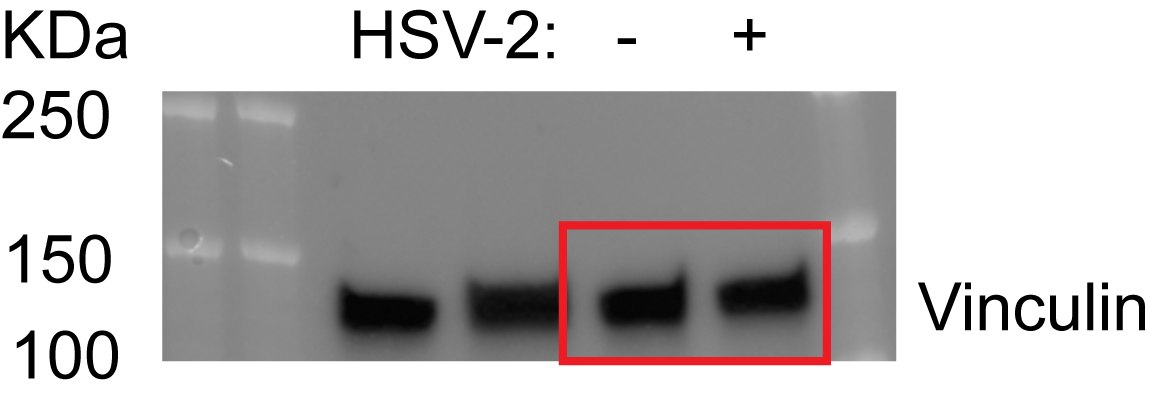

Supplement: Supplementary file 7 — Source Data for Figure 2 [file EMBJ-42-e113118-s011.zip › Source data Figure 2/2I/Western Blot Vinculin.tif]

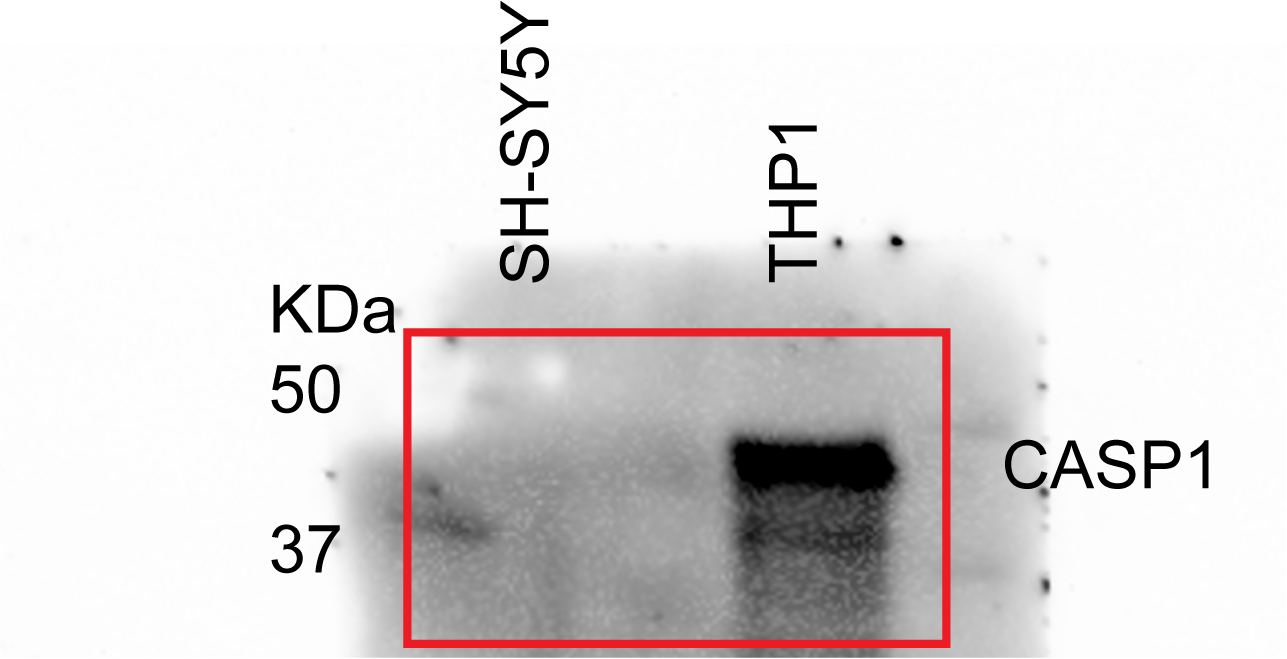

Supplement: Supplementary file 8 — Source Data for Figure 3 [file EMBJ-42-e113118-s008.zip › Source data Figure 3/3A/Western Blot CASP1.tif]

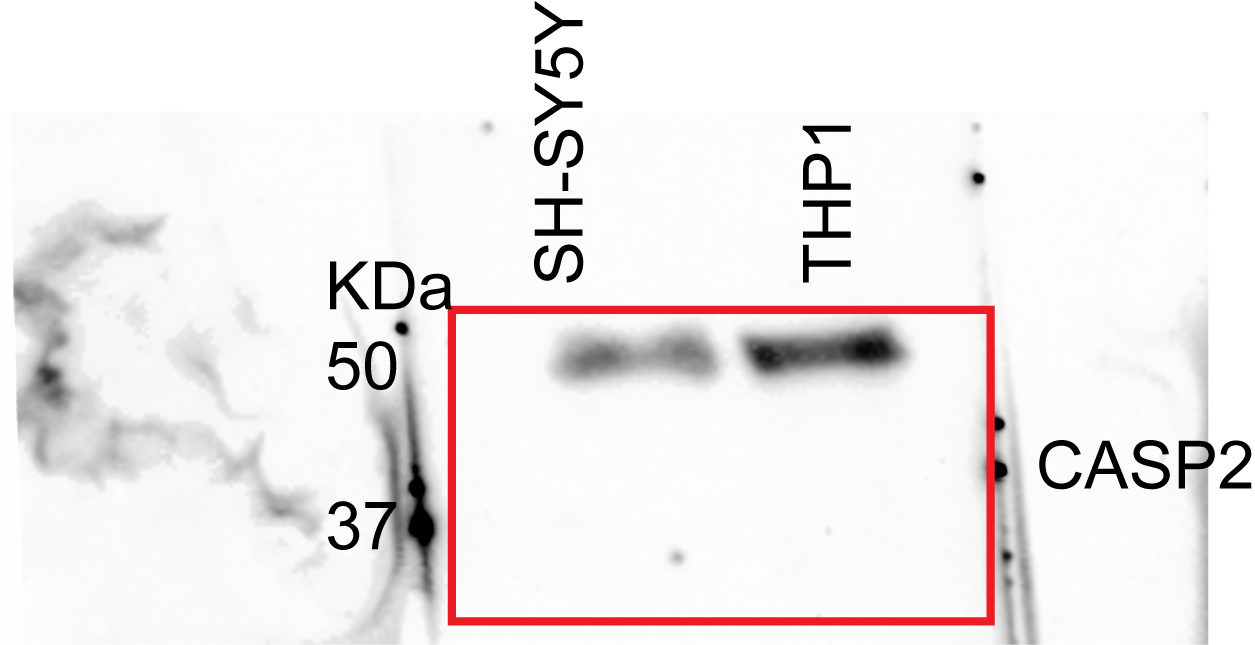

Supplement: Supplementary file 8 — Source Data for Figure 3 [file EMBJ-42-e113118-s008.zip › Source data Figure 3/3A/Western Blot CASP2.tif]

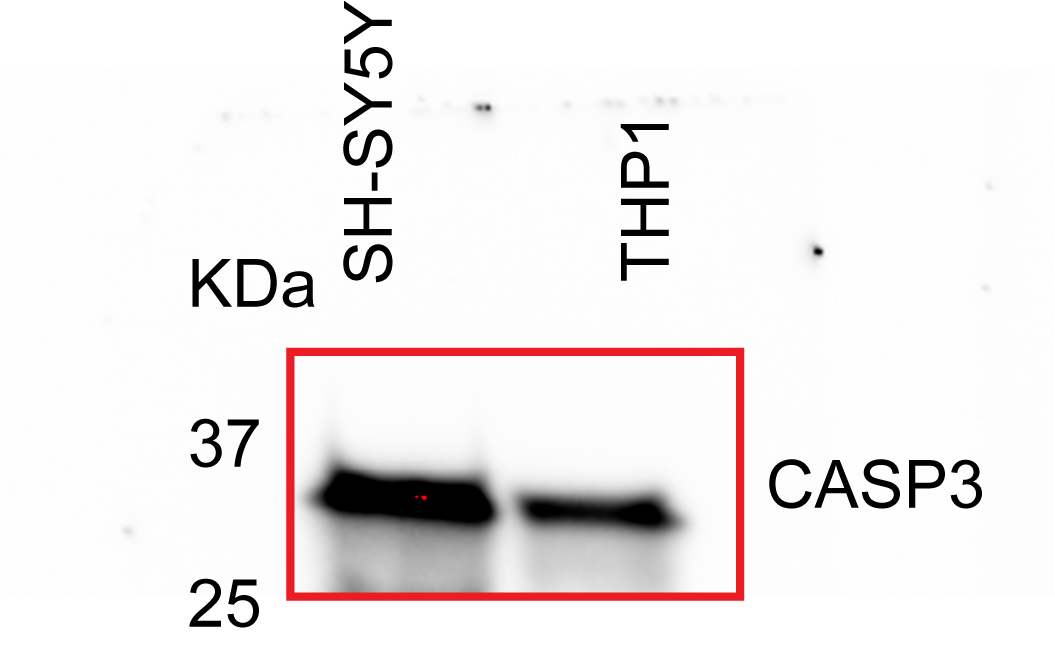

Supplement: Supplementary file 8 — Source Data for Figure 3 [file EMBJ-42-e113118-s008.zip › Source data Figure 3/3A/Western Blot CASP3.tif]

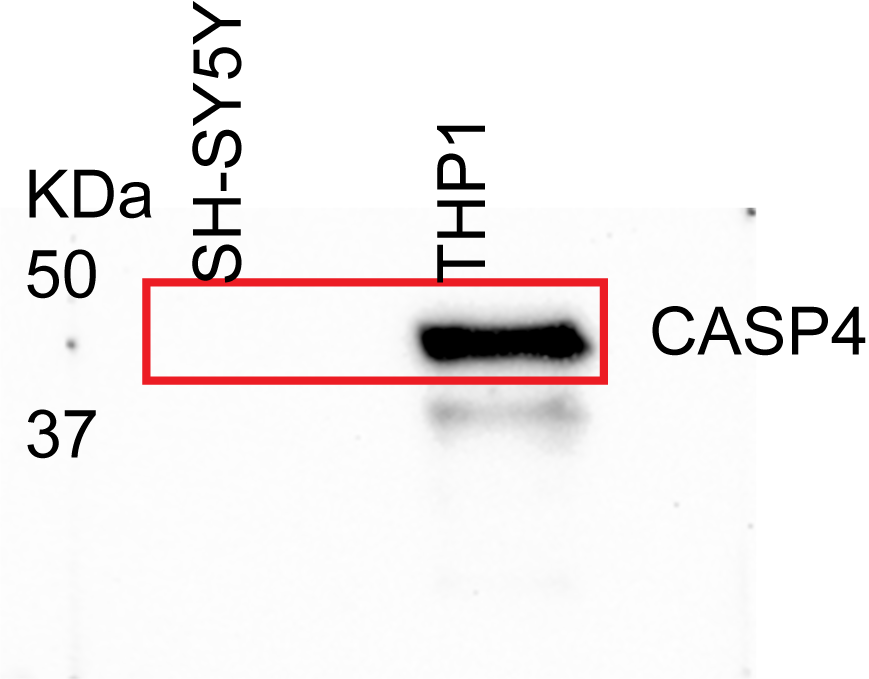

Supplement: Supplementary file 8 — Source Data for Figure 3 [file EMBJ-42-e113118-s008.zip › Source data Figure 3/3A/Western Blot CASP4.tif]

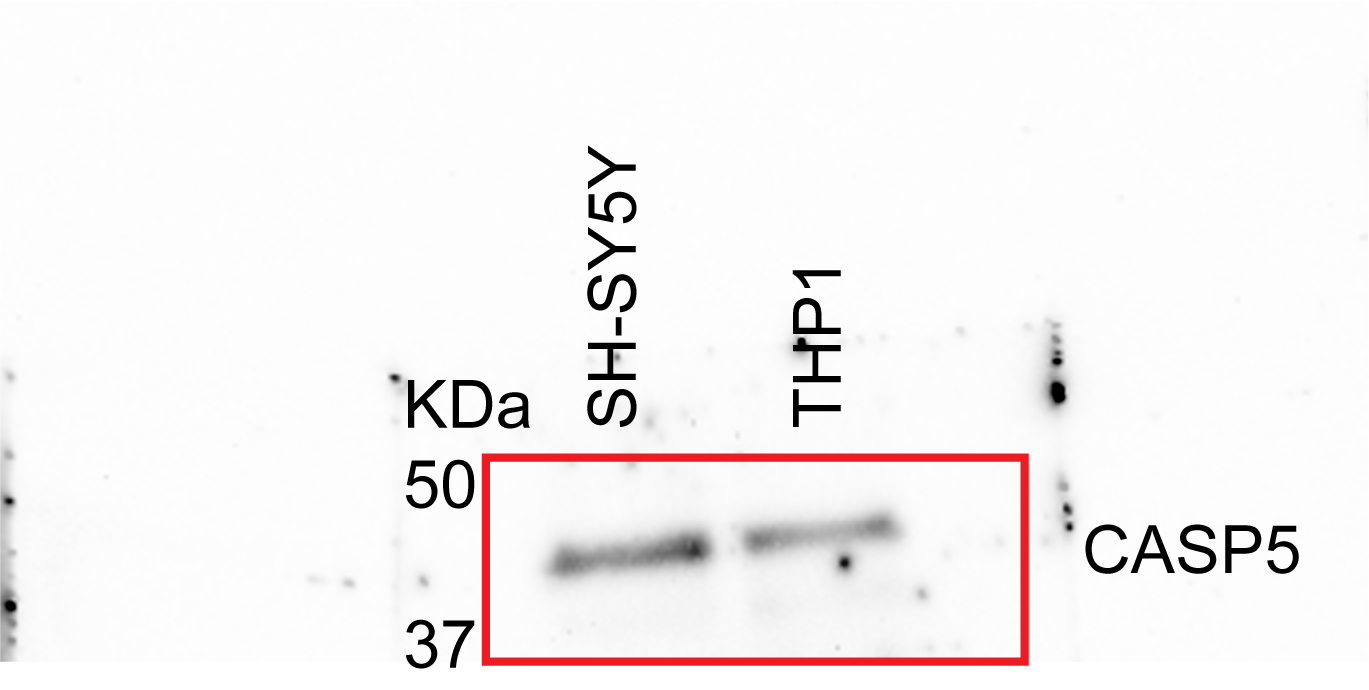

Supplement: Supplementary file 8 — Source Data for Figure 3 [file EMBJ-42-e113118-s008.zip › Source data Figure 3/3A/Western Blot CASP5.tif]

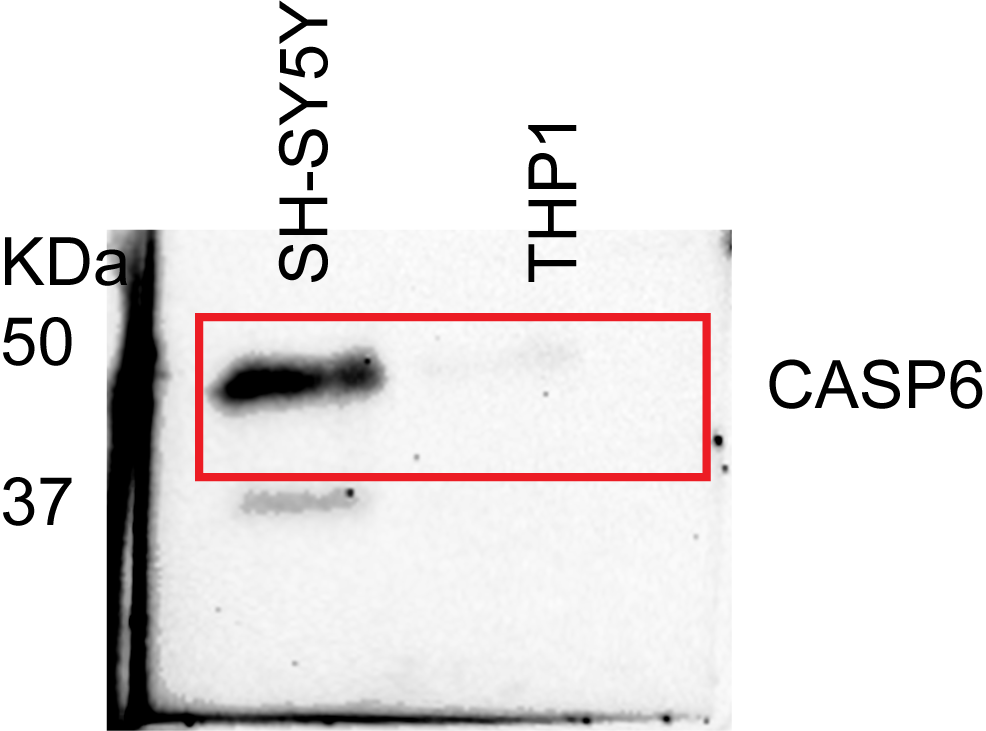

Supplement: Supplementary file 8 — Source Data for Figure 3 [file EMBJ-42-e113118-s008.zip › Source data Figure 3/3A/Western Blot CASP6.tif]

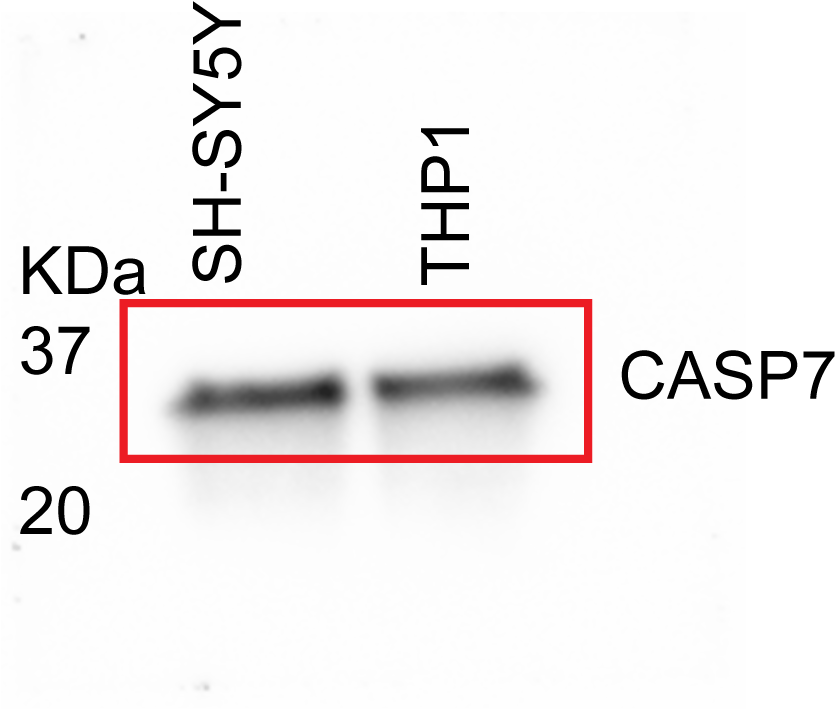

Supplement: Supplementary file 8 — Source Data for Figure 3 [file EMBJ-42-e113118-s008.zip › Source data Figure 3/3A/Western Blot CASP7.tif]

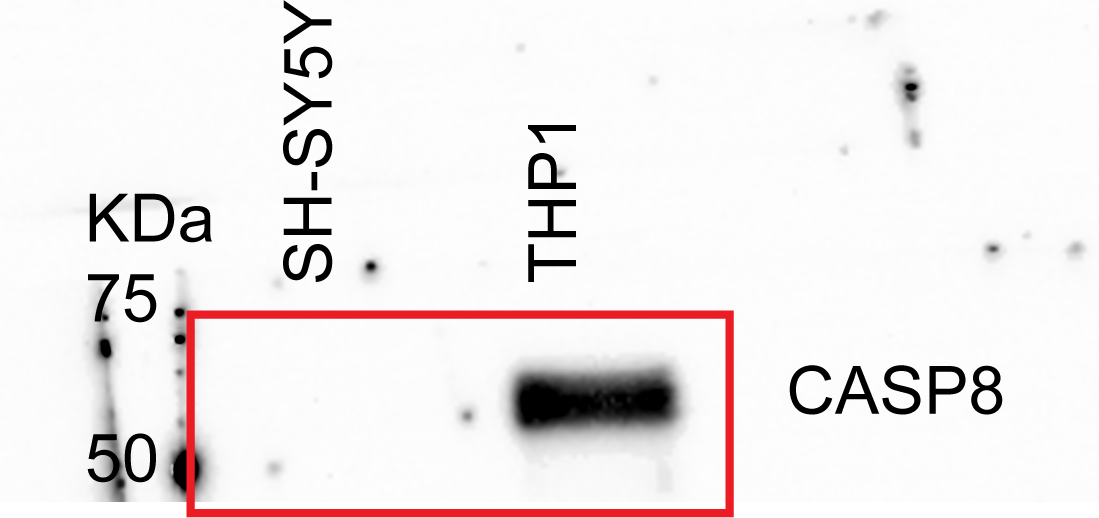

Supplement: Supplementary file 8 — Source Data for Figure 3 [file EMBJ-42-e113118-s008.zip › Source data Figure 3/3A/Western Blot CASP8.tif]

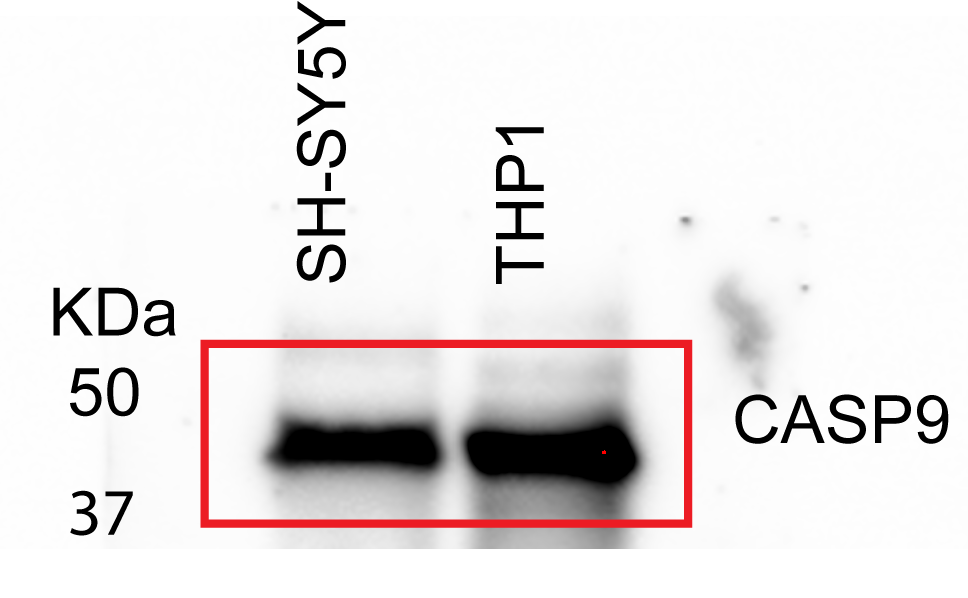

Supplement: Supplementary file 8 — Source Data for Figure 3 [file EMBJ-42-e113118-s008.zip › Source data Figure 3/3A/Western Blot CASP9.tif]

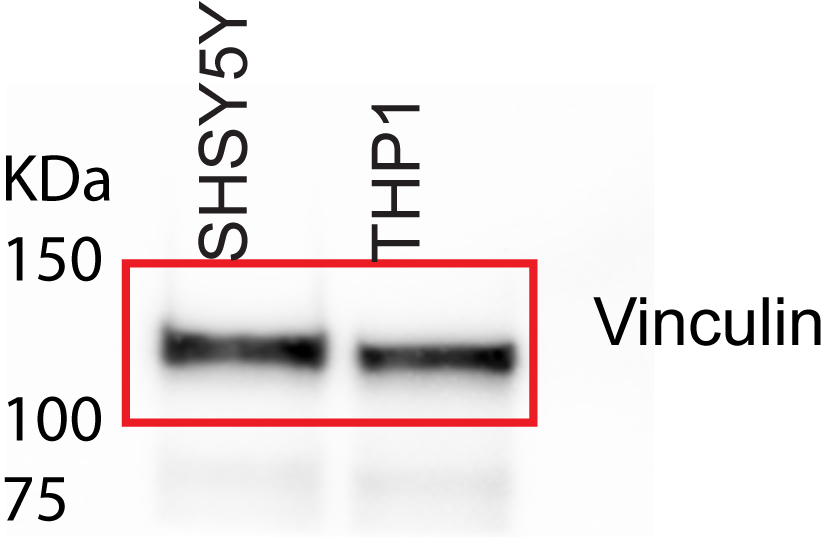

Supplement: Supplementary file 8 — Source Data for Figure 3 [file EMBJ-42-e113118-s008.zip › Source data Figure 3/3A/Western Blot Vinculin.tif]

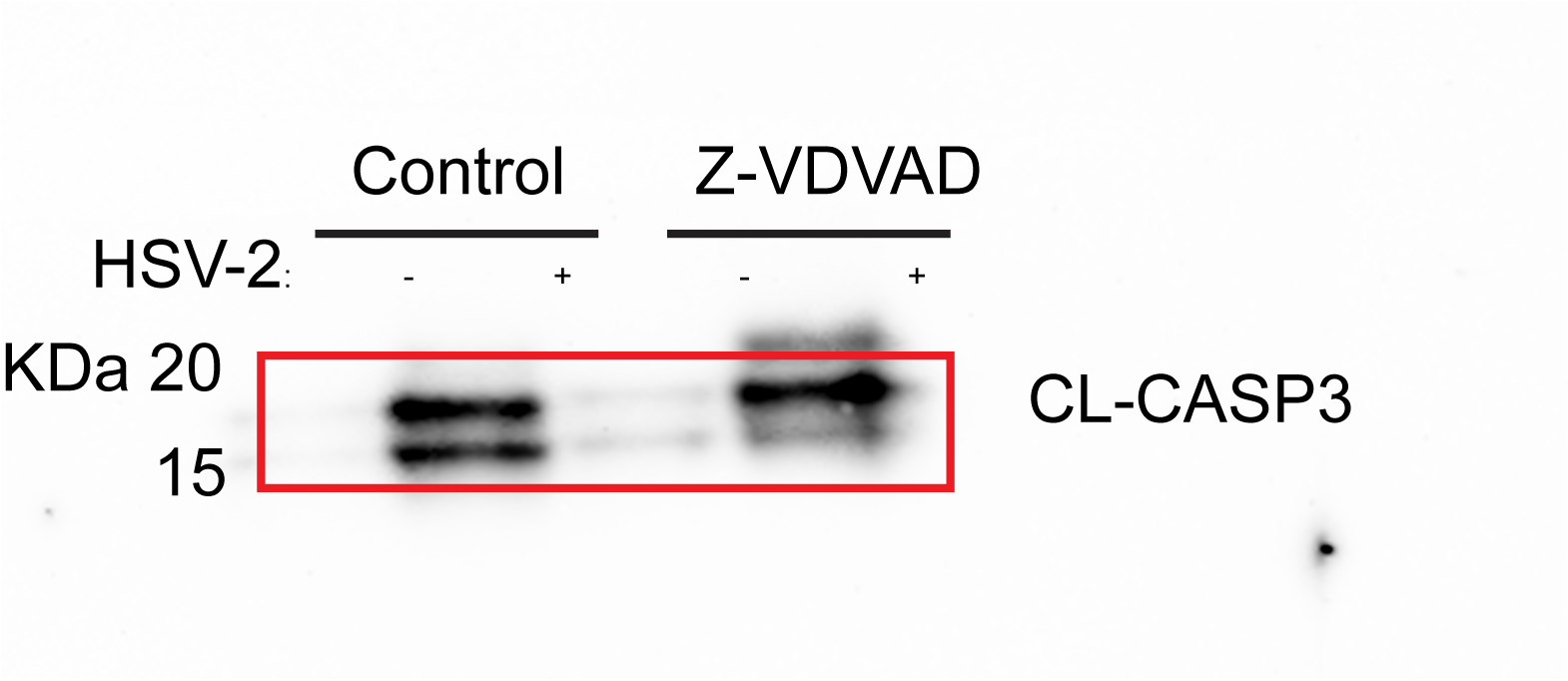

Supplement: Supplementary file 8 — Source Data for Figure 3 [file EMBJ-42-e113118-s008.zip › Source data Figure 3/3B/Western Blot CL-CASP3.tif]

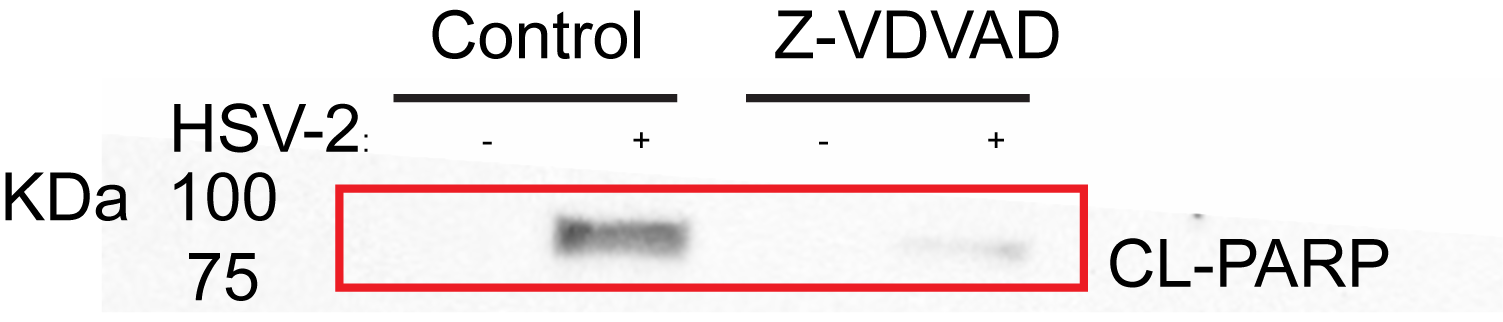

Supplement: Supplementary file 8 — Source Data for Figure 3 [file EMBJ-42-e113118-s008.zip › Source data Figure 3/3B/Western Blot CL-PARP.tif]

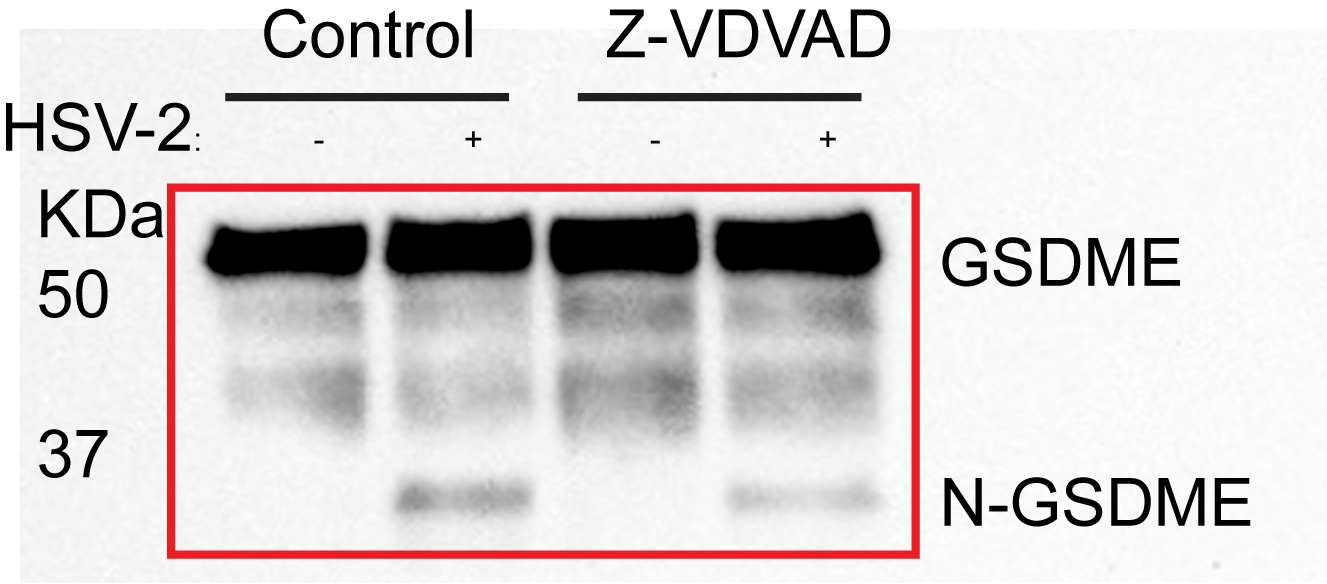

Supplement: Supplementary file 8 — Source Data for Figure 3 [file EMBJ-42-e113118-s008.zip › Source data Figure 3/3B/Western Blot GSDME.tif]

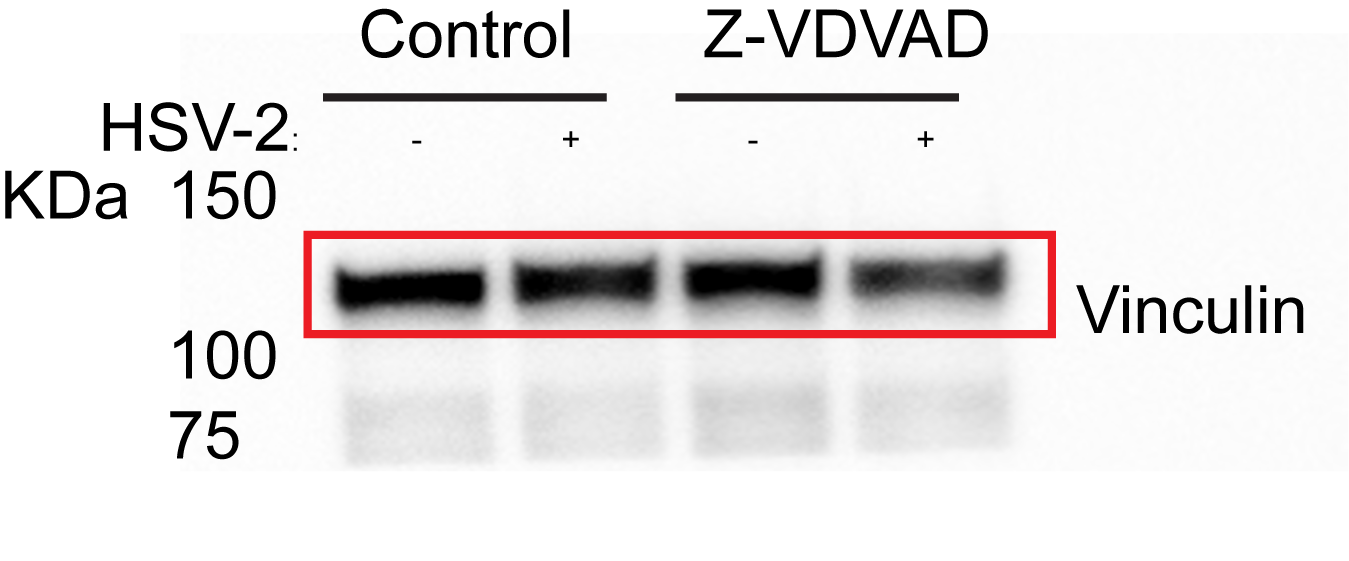

Supplement: Supplementary file 8 — Source Data for Figure 3 [file EMBJ-42-e113118-s008.zip › Source data Figure 3/3B/Western Blot Vinculin.tif]

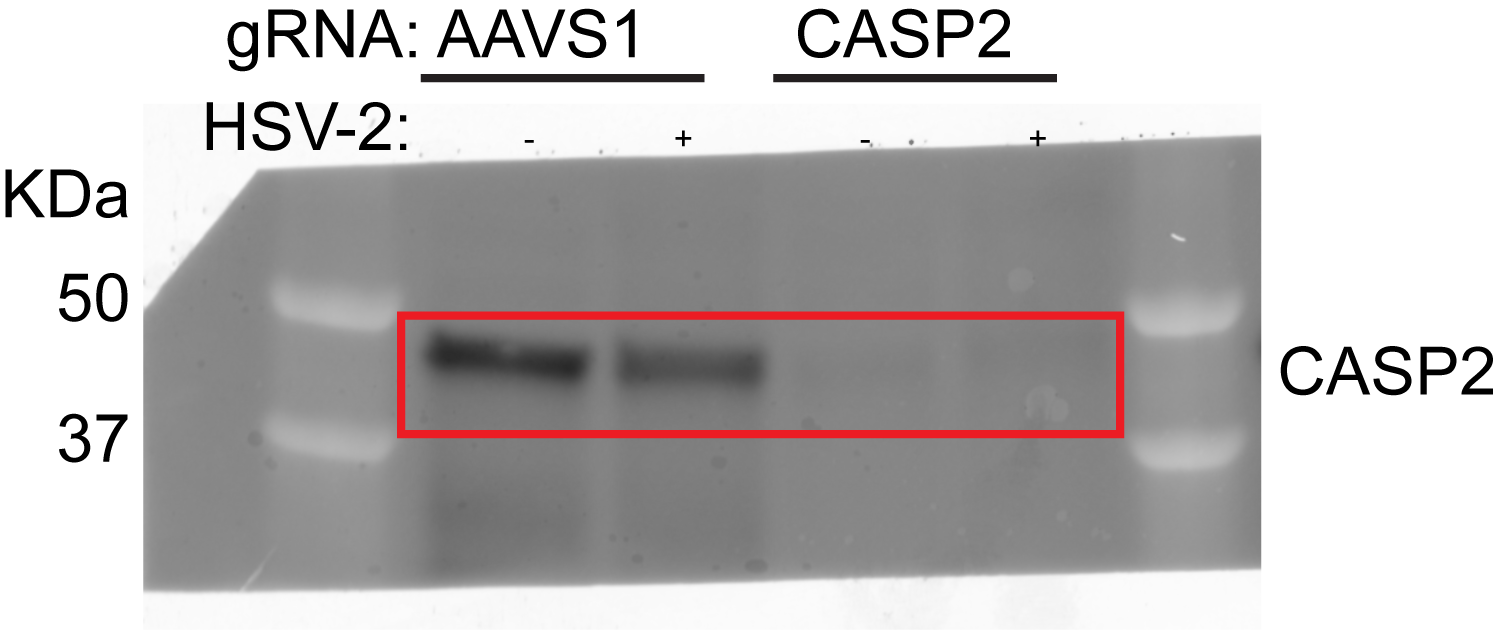

Supplement: Supplementary file 8 — Source Data for Figure 3 [file EMBJ-42-e113118-s008.zip › Source data Figure 3/3C/Western Blot CASP2.tif]

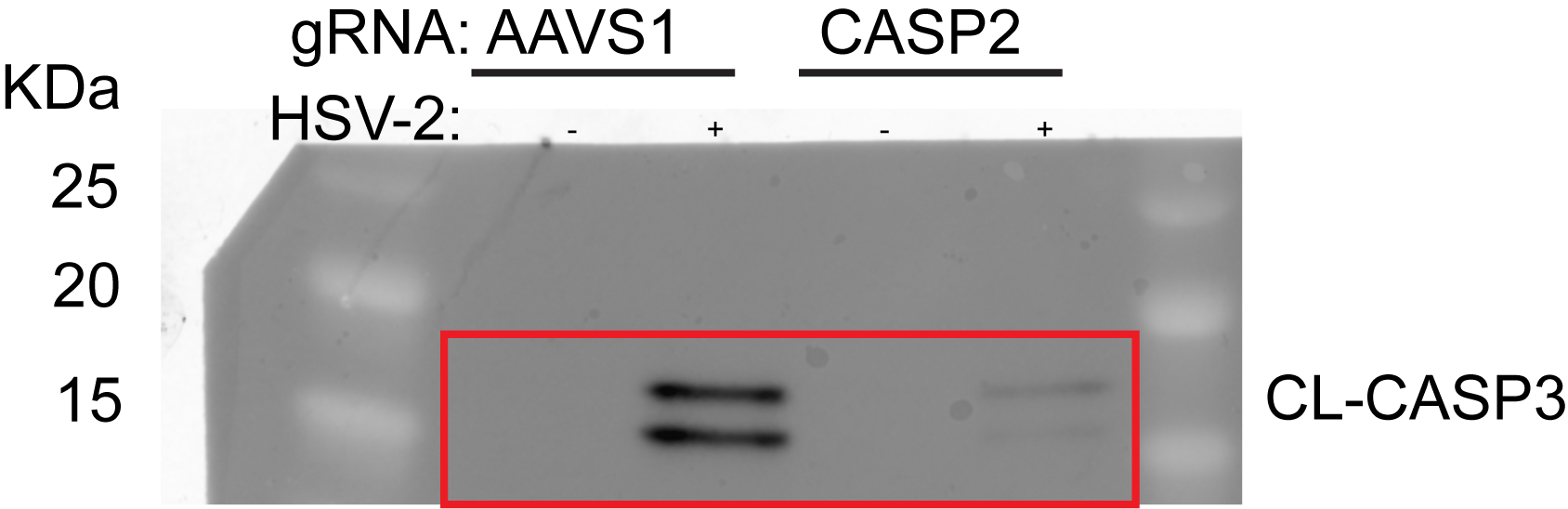

Supplement: Supplementary file 8 — Source Data for Figure 3 [file EMBJ-42-e113118-s008.zip › Source data Figure 3/3C/Western Blot CL-CASP3.tif]

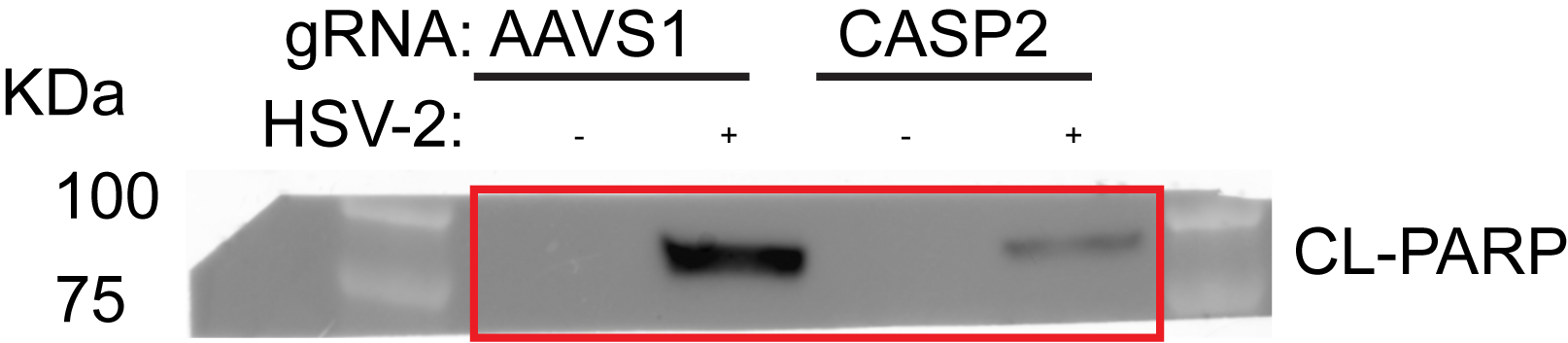

Supplement: Supplementary file 8 — Source Data for Figure 3 [file EMBJ-42-e113118-s008.zip › Source data Figure 3/3C/Western Blot CL-PARP.tif]

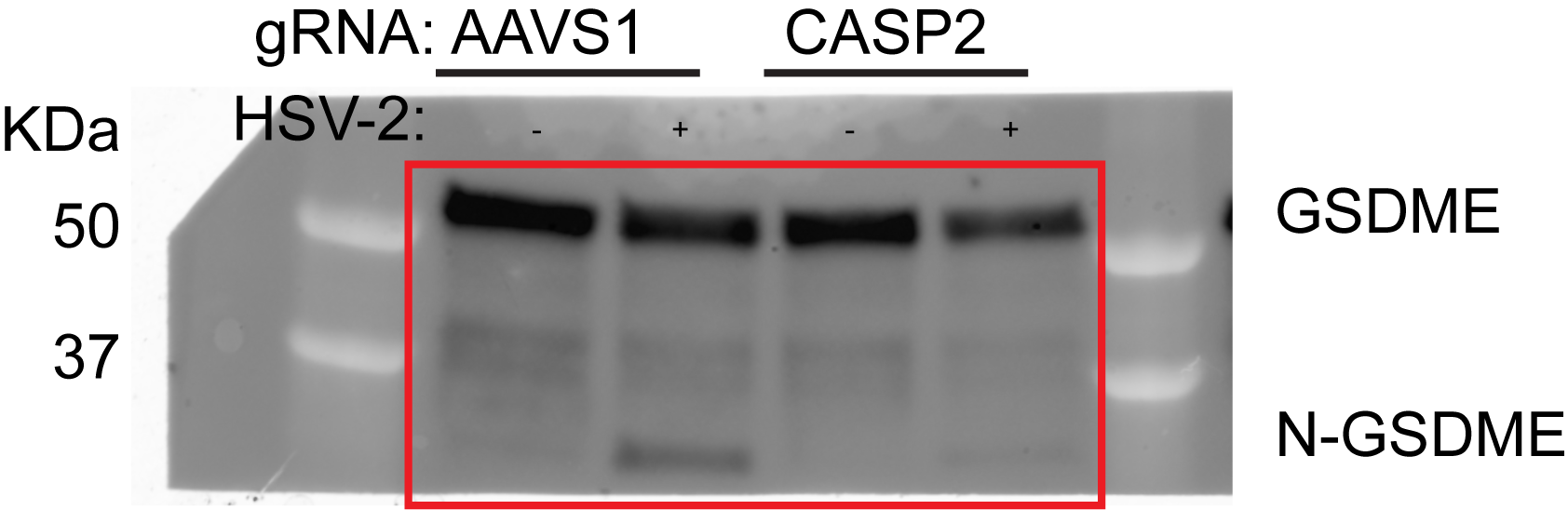

Supplement: Supplementary file 8 — Source Data for Figure 3 [file EMBJ-42-e113118-s008.zip › Source data Figure 3/3C/Western Blot GSDME.tif]

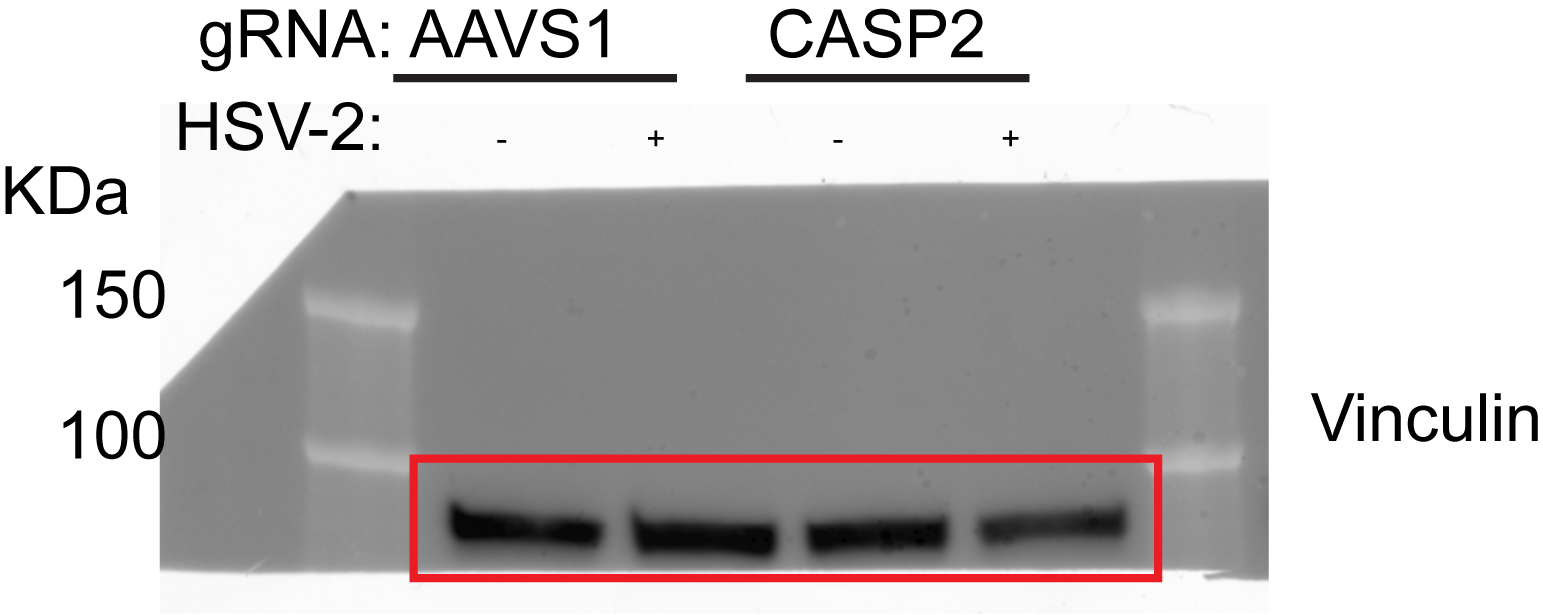

Supplement: Supplementary file 8 — Source Data for Figure 3 [file EMBJ-42-e113118-s008.zip › Source data Figure 3/3C/Western Blot Vinculin.tif]

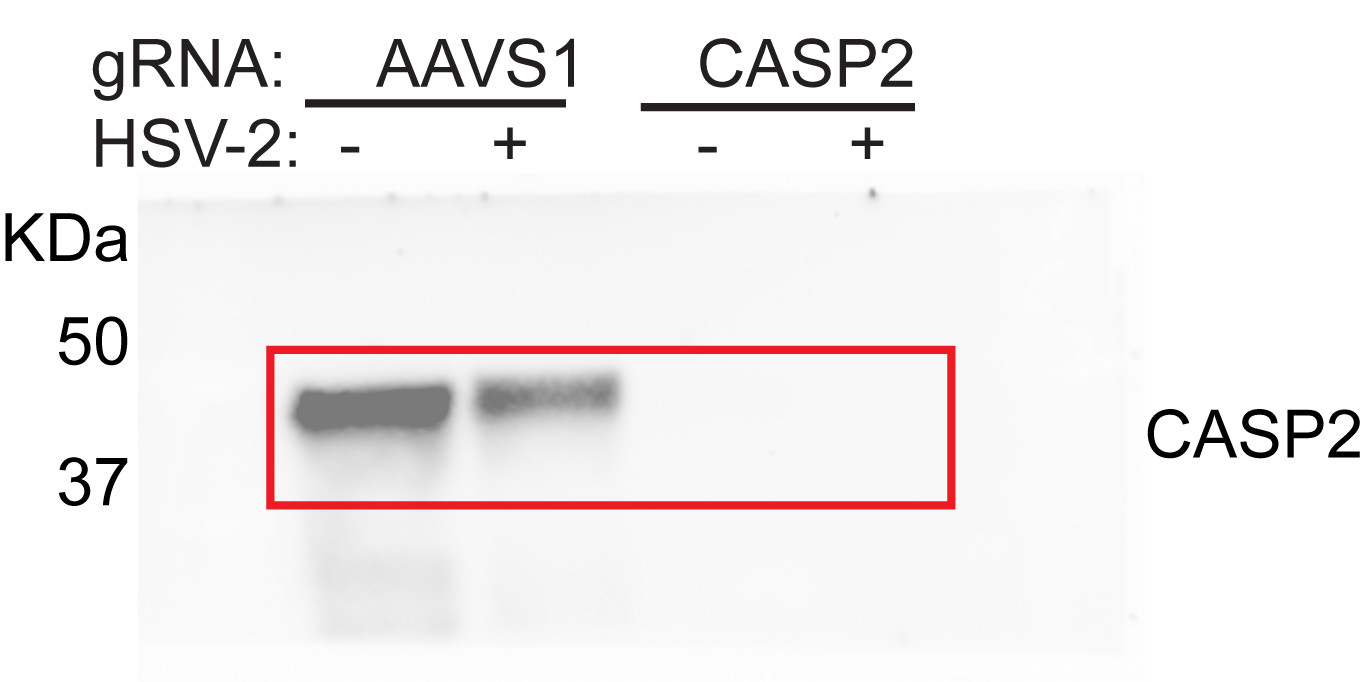

Supplement: Supplementary file 8 — Source Data for Figure 3 [file EMBJ-42-e113118-s008.zip › Source data Figure 3/3G/Western Blot CASP2.tif]

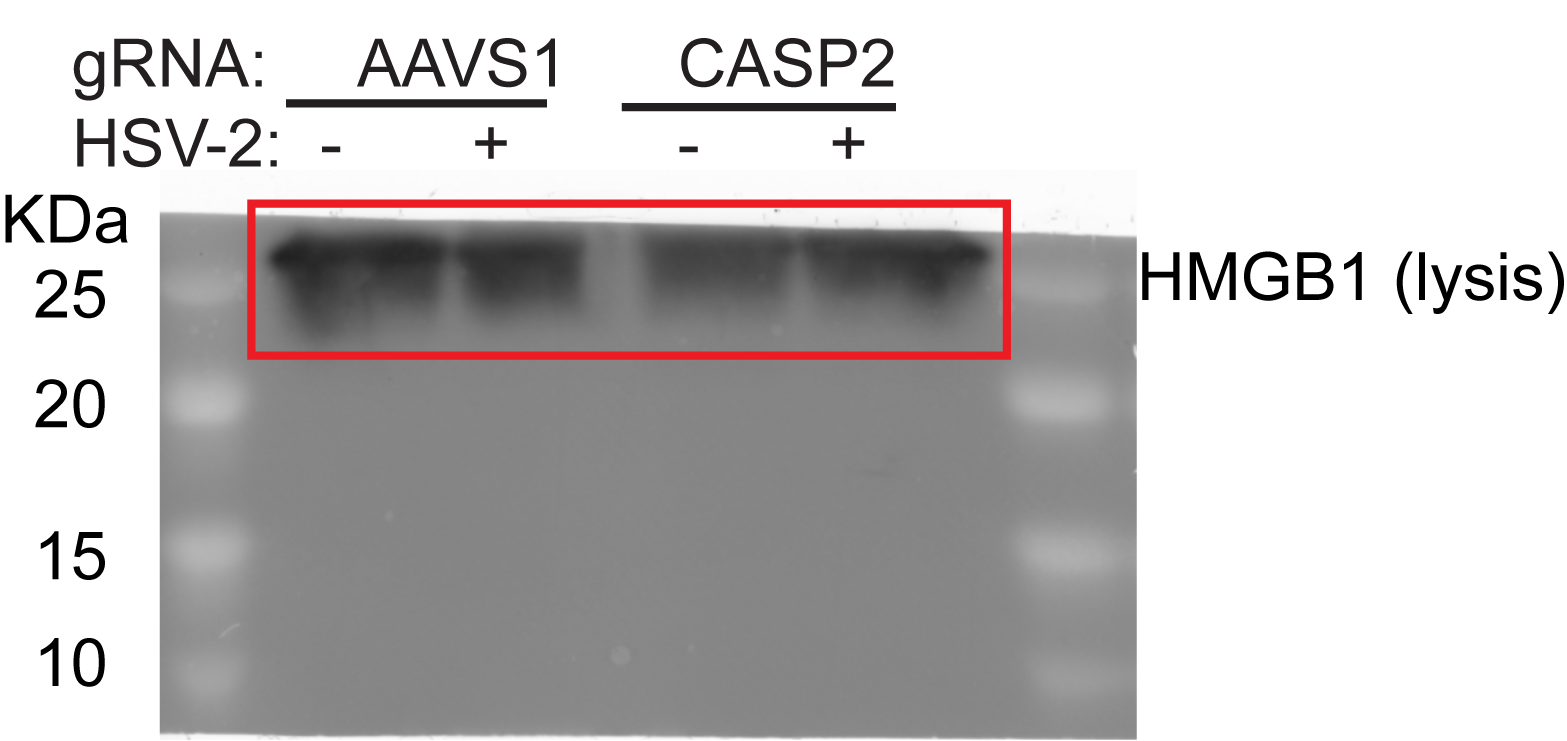

Supplement: Supplementary file 8 — Source Data for Figure 3 [file EMBJ-42-e113118-s008.zip › Source data Figure 3/3G/Western Blot HMGB1 (lysis).tif]

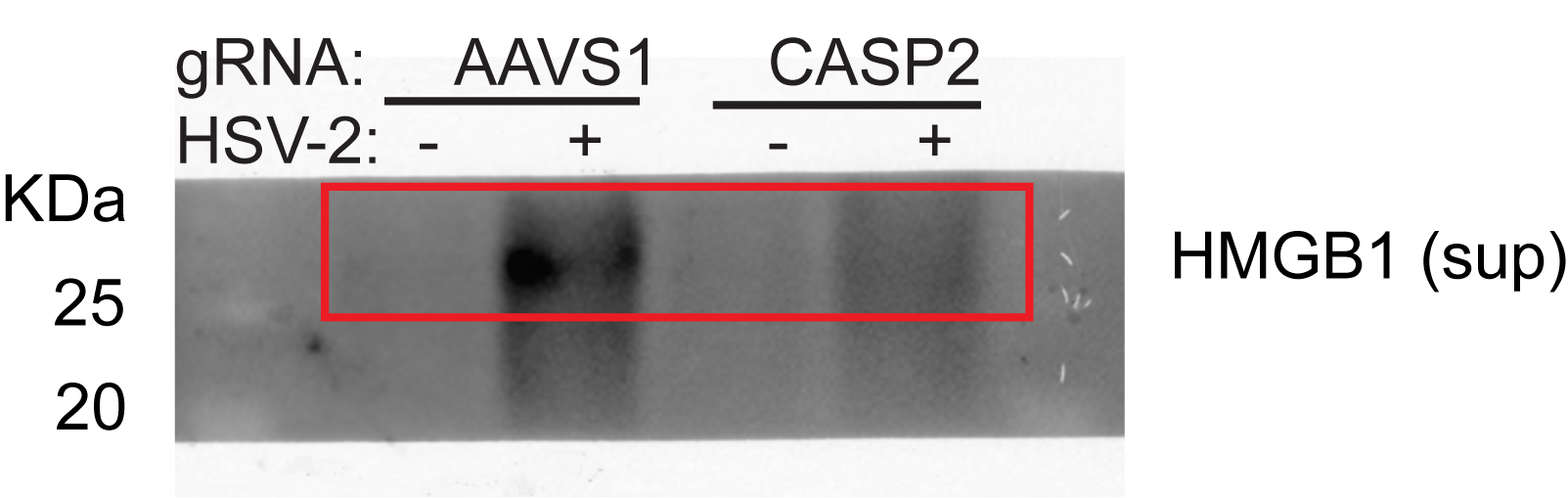

Supplement: Supplementary file 8 — Source Data for Figure 3 [file EMBJ-42-e113118-s008.zip › Source data Figure 3/3G/Western Blot HMGB1 (sup).tif]

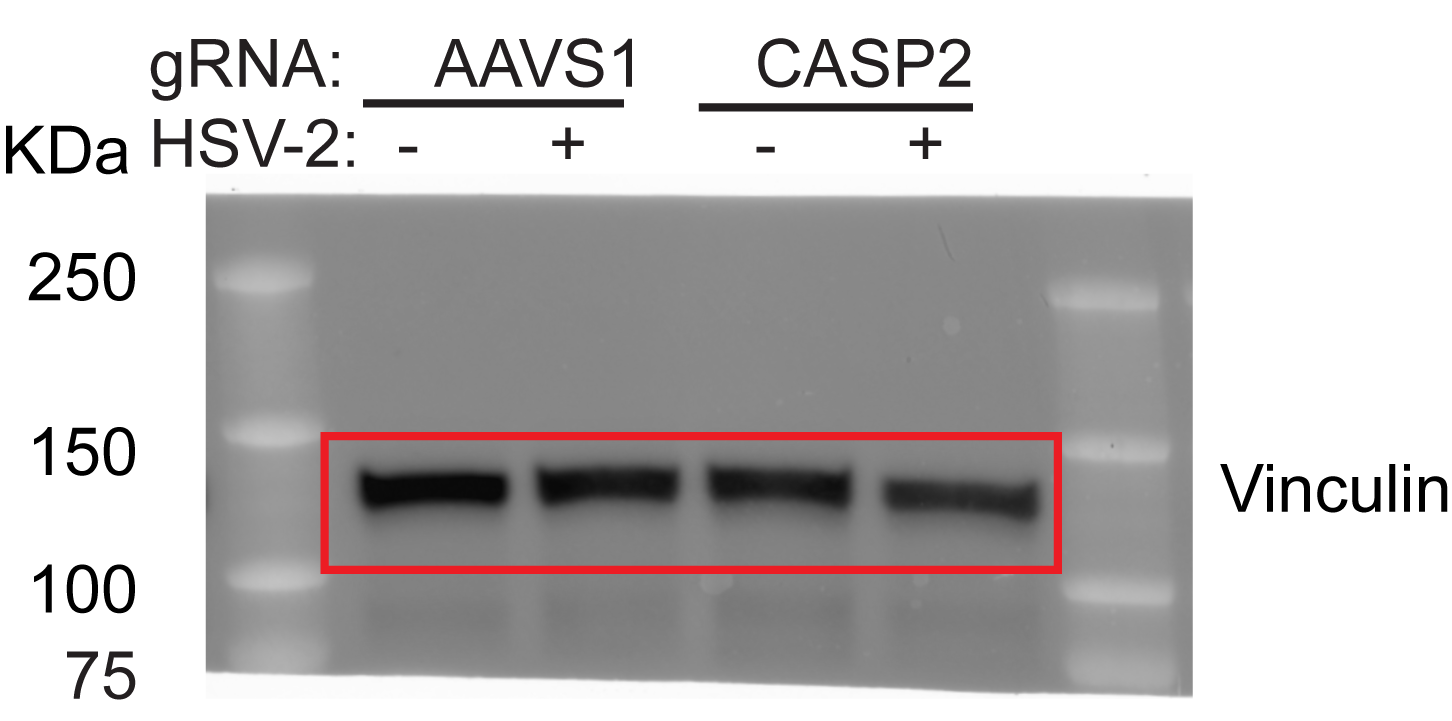

Supplement: Supplementary file 8 — Source Data for Figure 3 [file EMBJ-42-e113118-s008.zip › Source data Figure 3/3G/Western Blot Vinculin.tif]

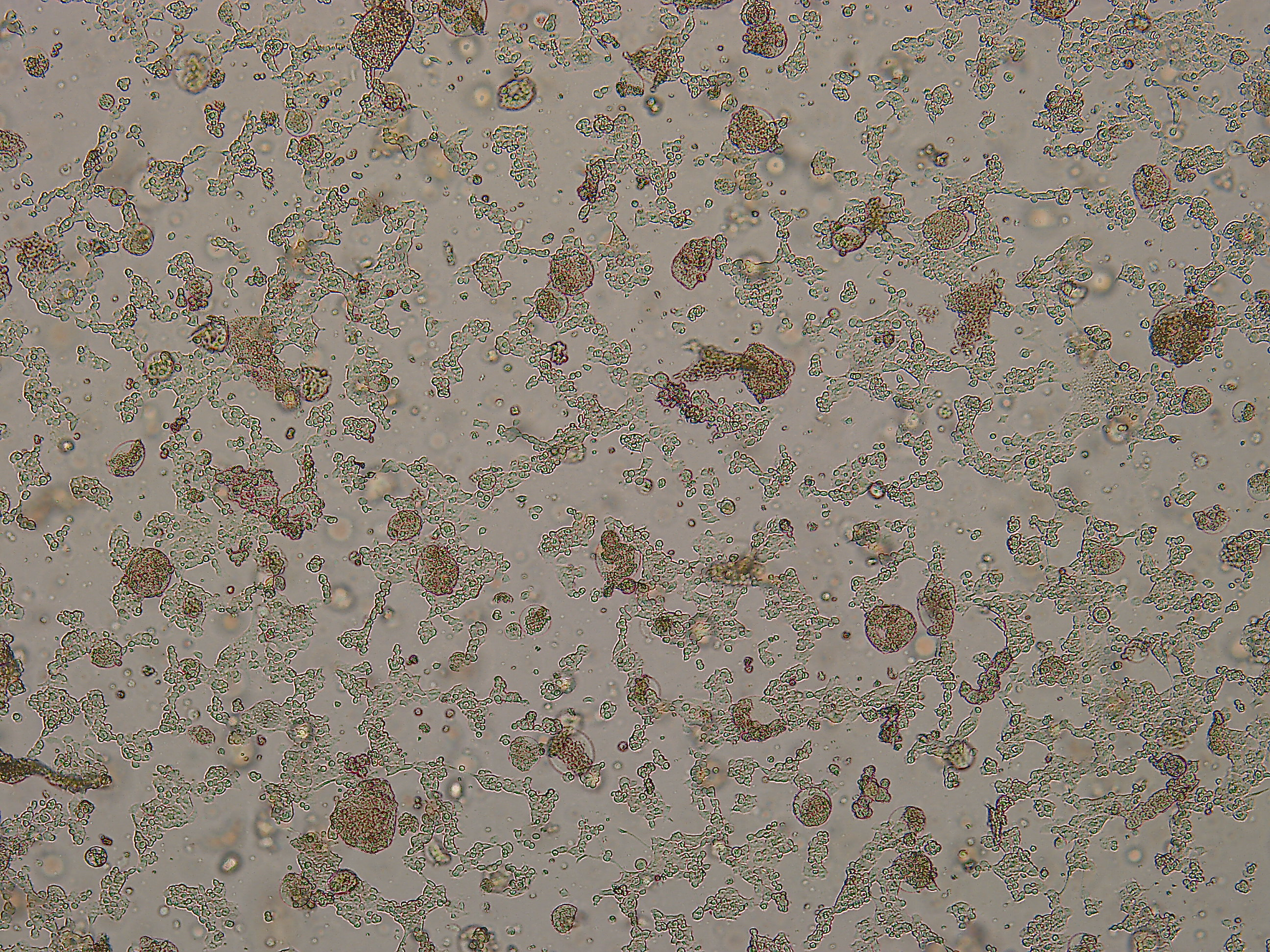

Supplement: Supplementary file 8 — Source Data for Figure 3 [file EMBJ-42-e113118-s008.zip › Source data Figure 3/3H/AAVS1 HSV2.tif]

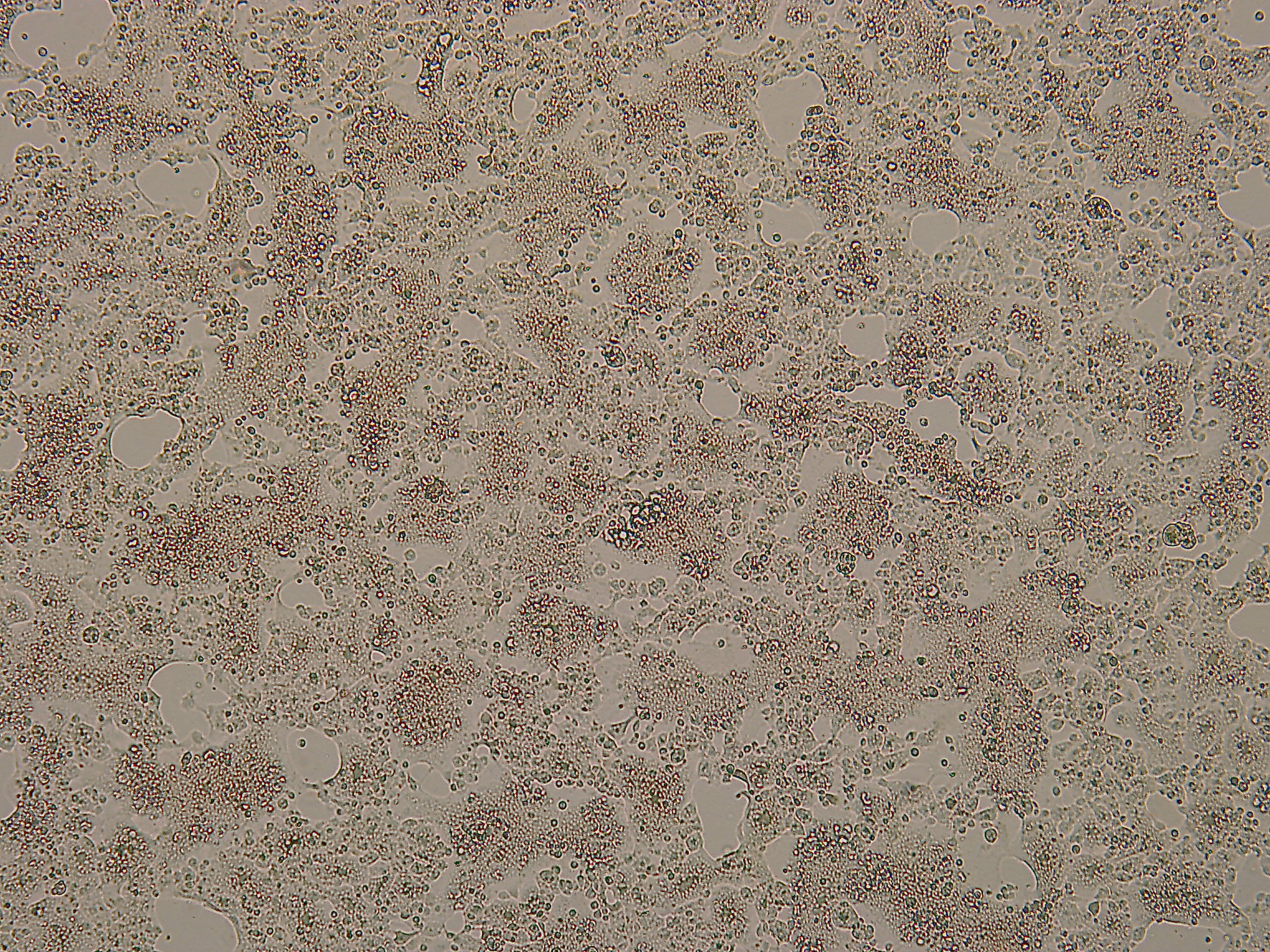

Supplement: Supplementary file 8 — Source Data for Figure 3 [file EMBJ-42-e113118-s008.zip › Source data Figure 3/3H/CASP2 ko 1# HSV2.tif]

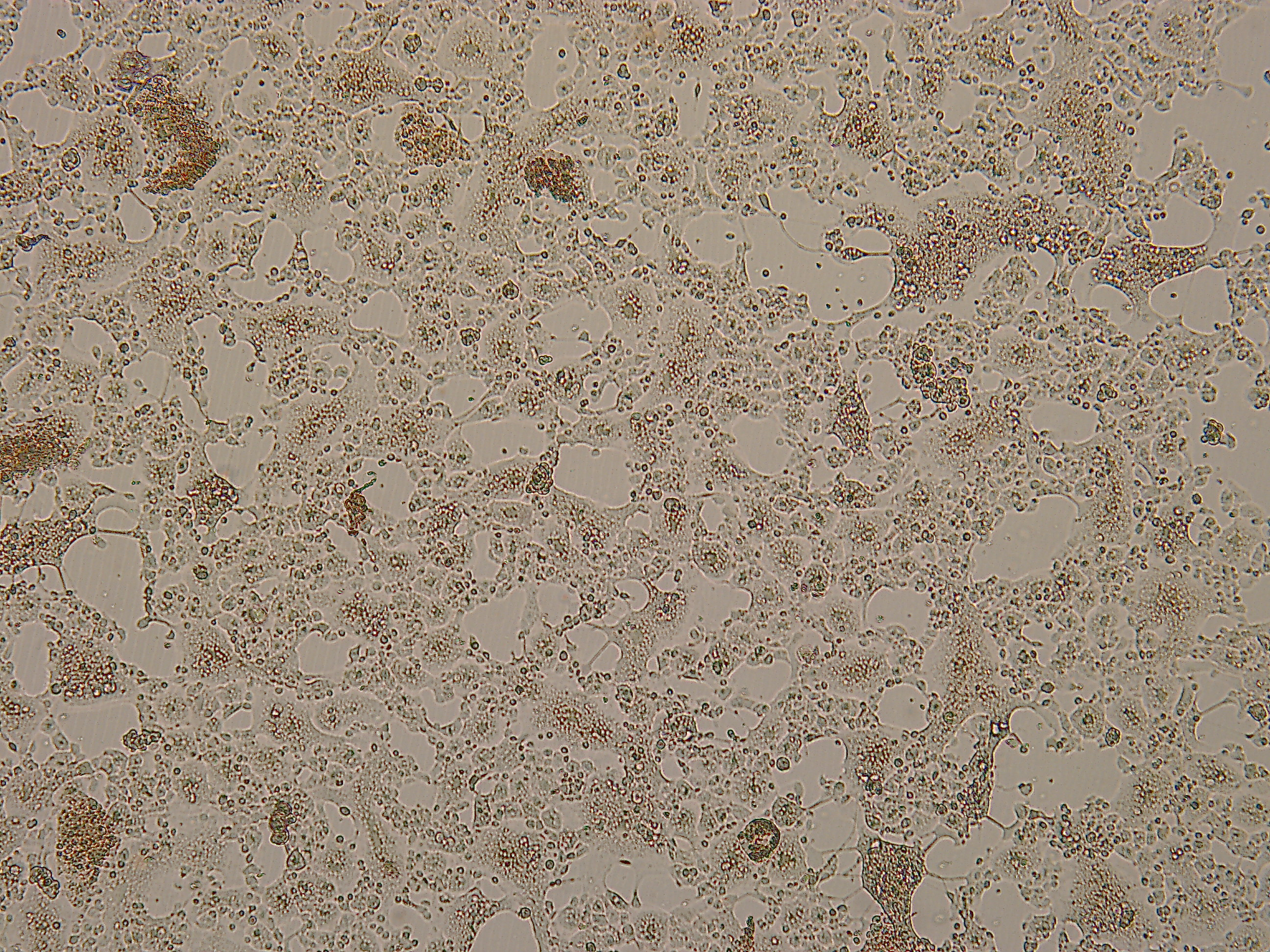

Supplement: Supplementary file 8 — Source Data for Figure 3 [file EMBJ-42-e113118-s008.zip › Source data Figure 3/3H/CASP2 KO 2# HSV2.tif]

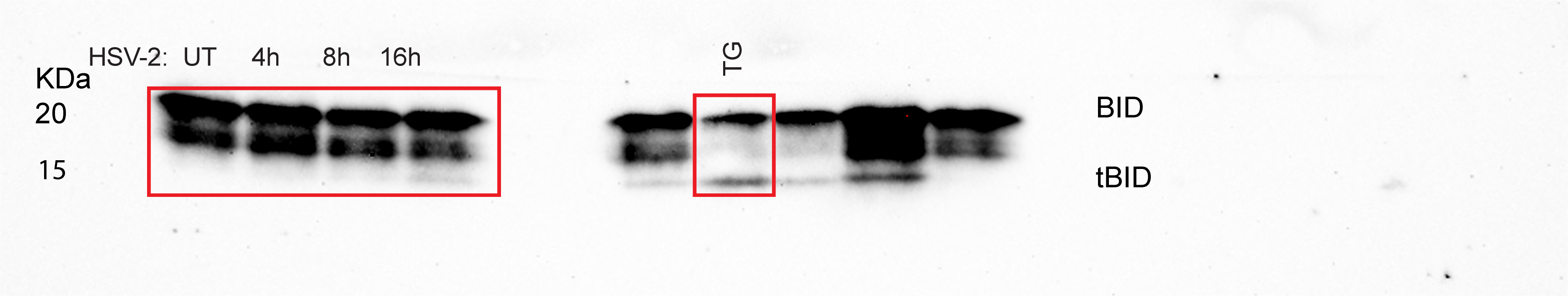

Supplement: Supplementary file 9 — Source Data for Figure 4 [file EMBJ-42-e113118-s007.zip › Source data Figure 4/4A/Western Blot BID.tif]

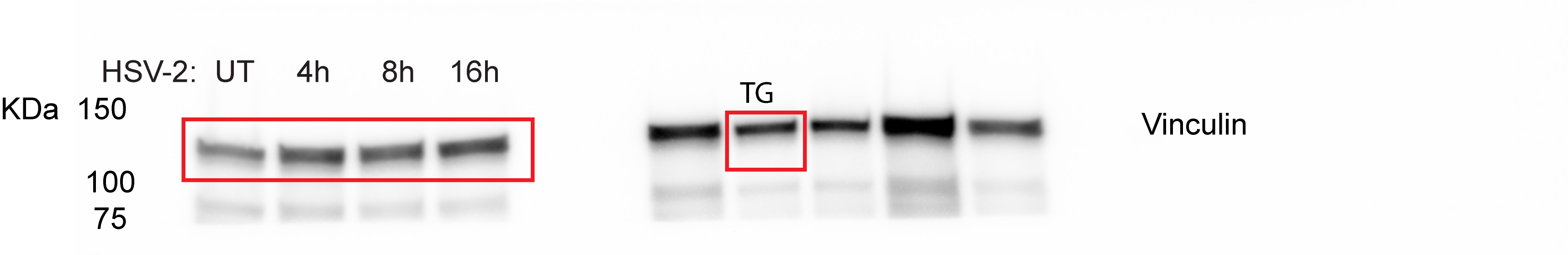

Supplement: Supplementary file 9 — Source Data for Figure 4 [file EMBJ-42-e113118-s007.zip › Source data Figure 4/4A/Western Blot Vinculin.tif]

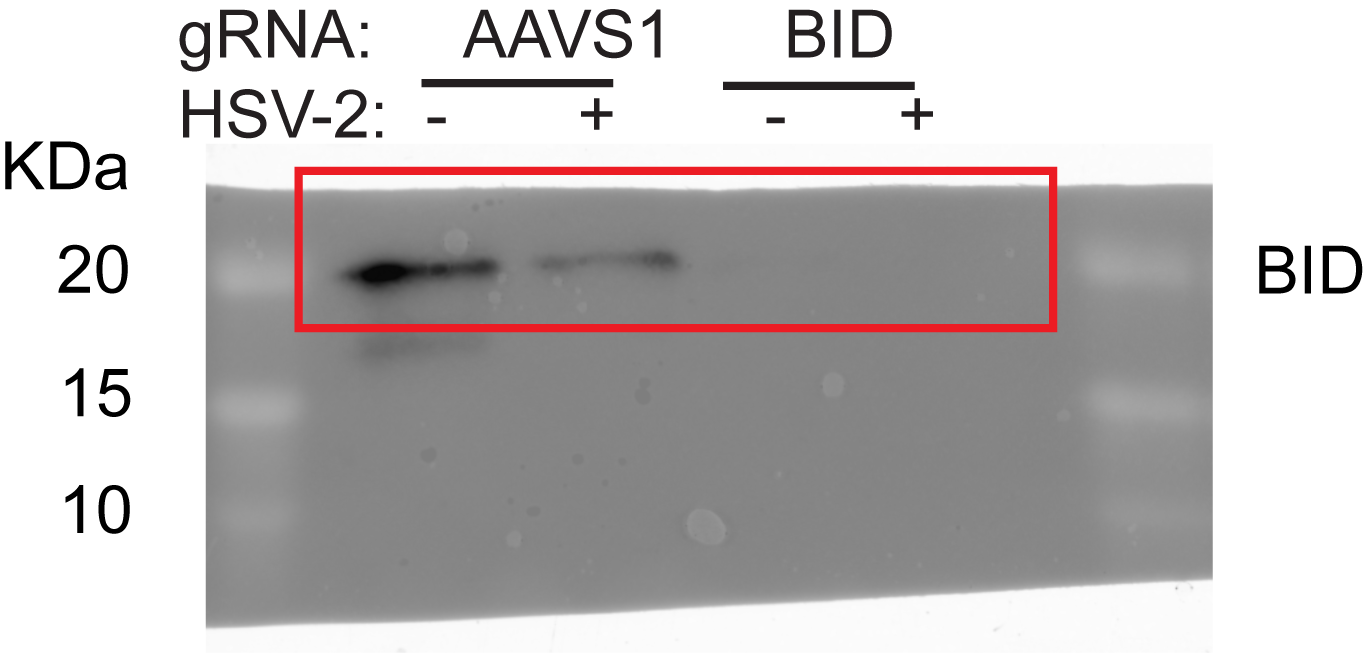

Supplement: Supplementary file 9 — Source Data for Figure 4 [file EMBJ-42-e113118-s007.zip › Source data Figure 4/4B/Western Blot BID.tif]

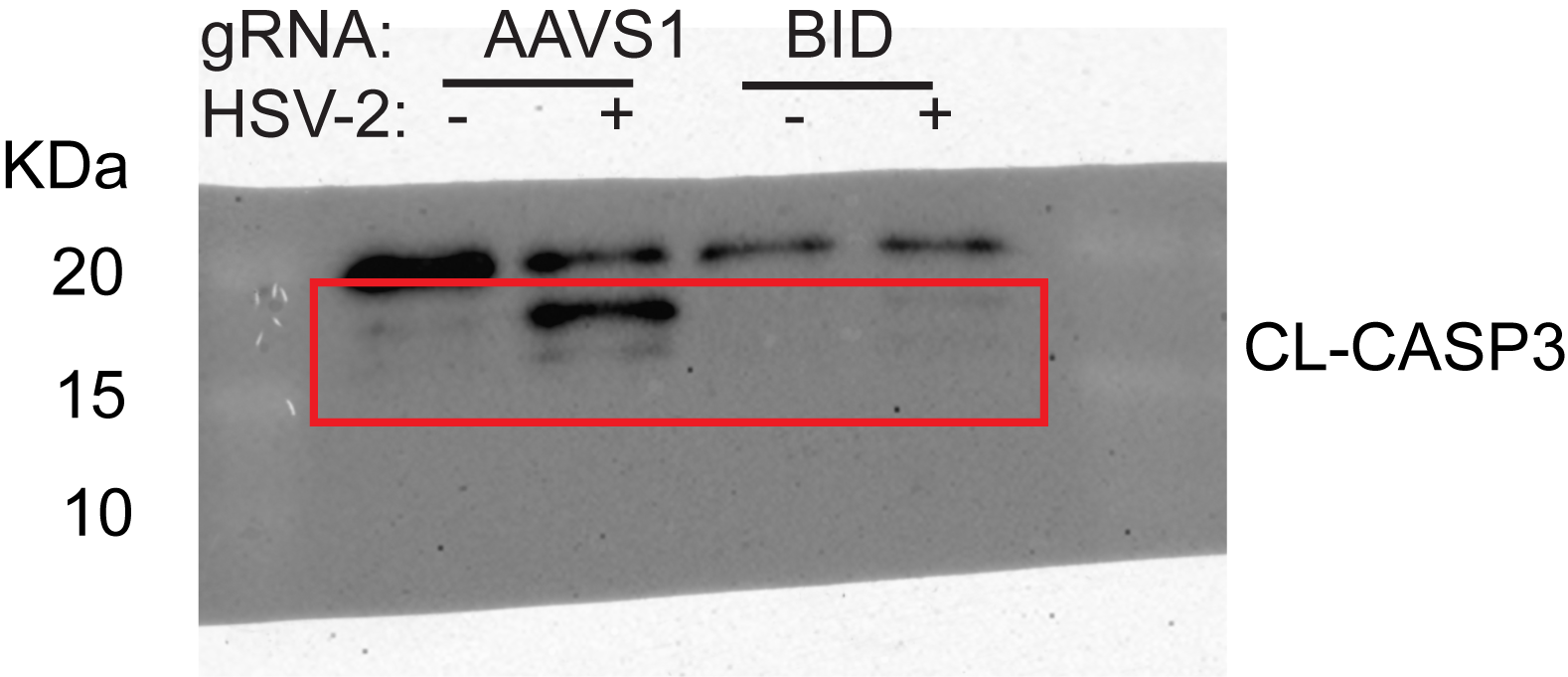

Supplement: Supplementary file 9 — Source Data for Figure 4 [file EMBJ-42-e113118-s007.zip › Source data Figure 4/4B/Western Blot CL-CASP3.tif]

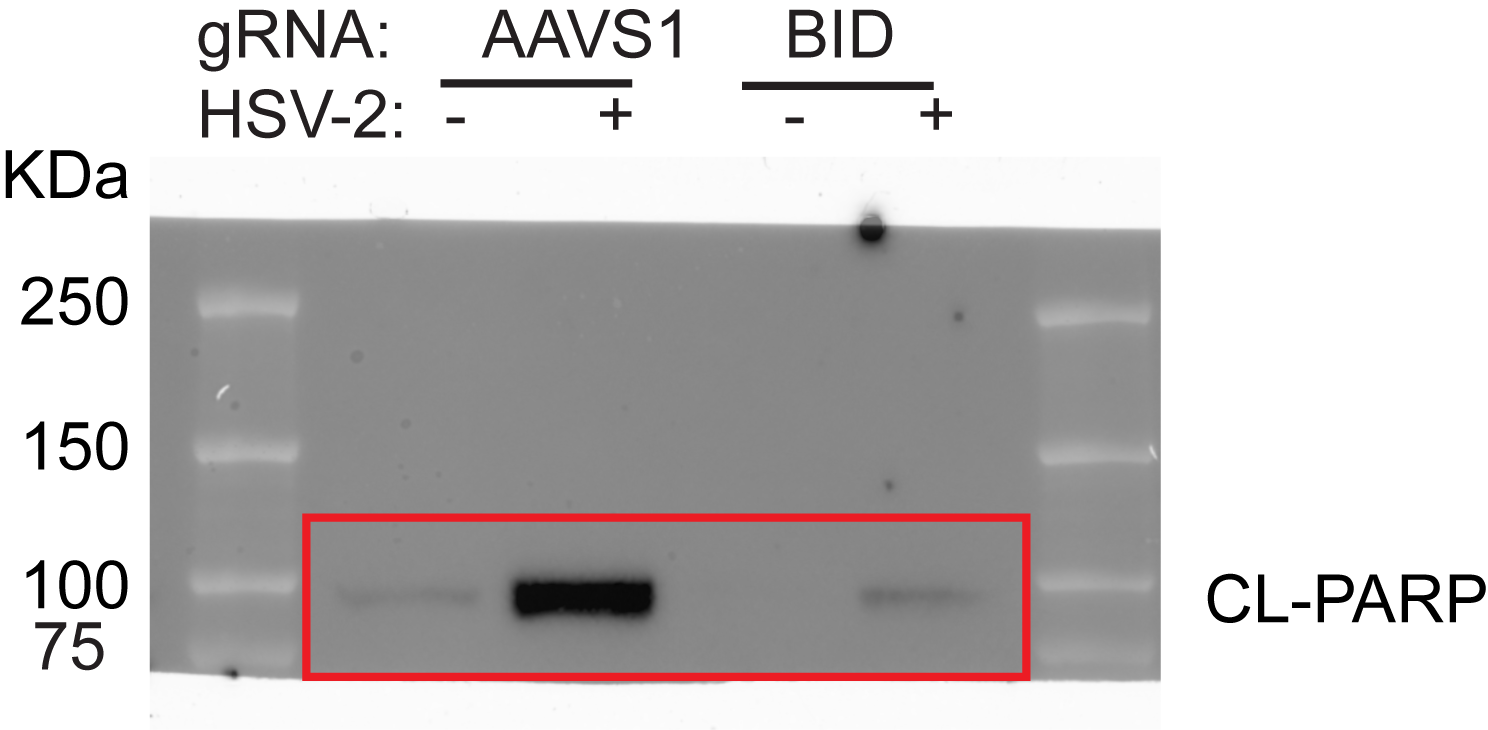

Supplement: Supplementary file 9 — Source Data for Figure 4 [file EMBJ-42-e113118-s007.zip › Source data Figure 4/4B/Western Blot CL-PARP.tif]

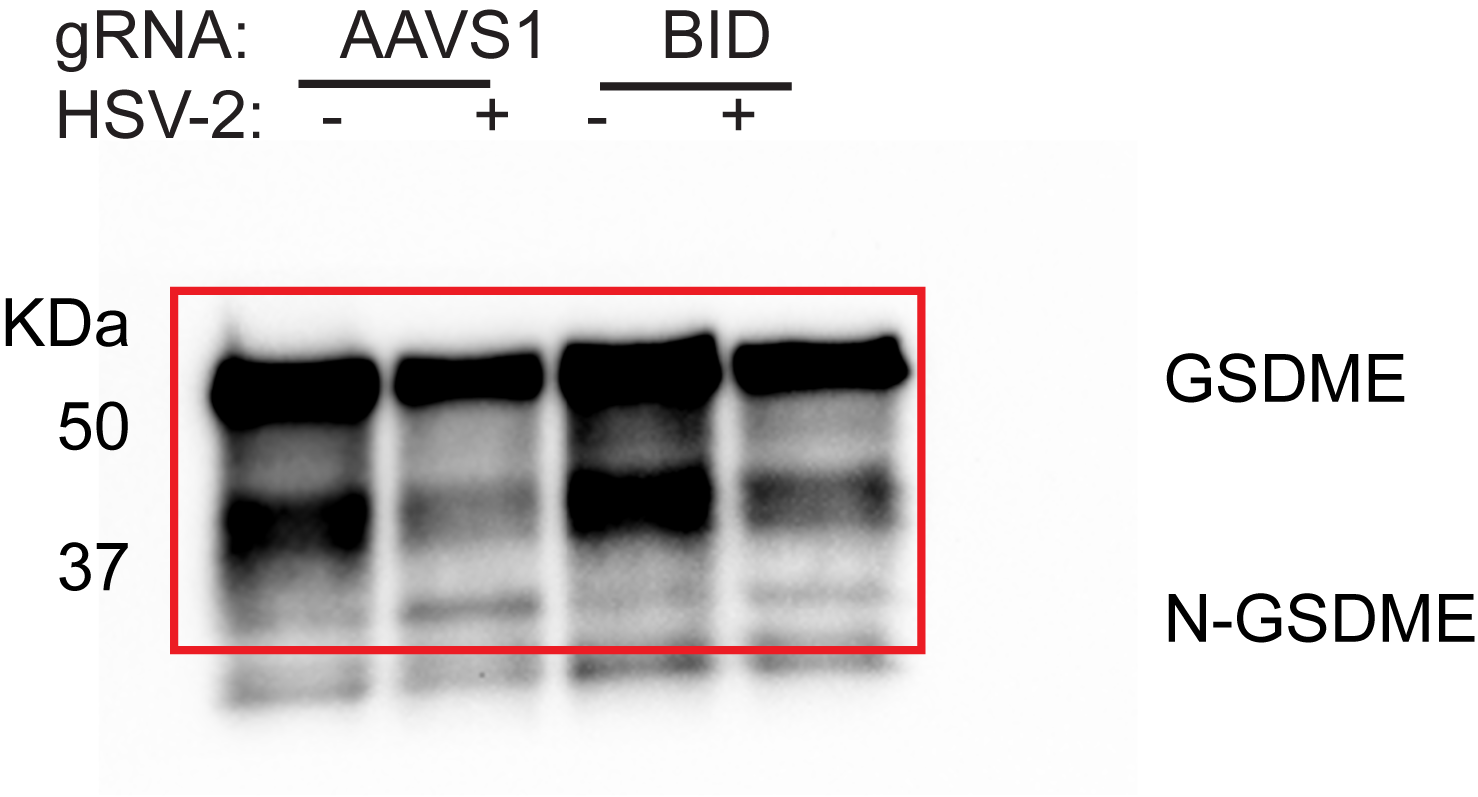

Supplement: Supplementary file 9 — Source Data for Figure 4 [file EMBJ-42-e113118-s007.zip › Source data Figure 4/4B/Western Blot GSDME.tif]

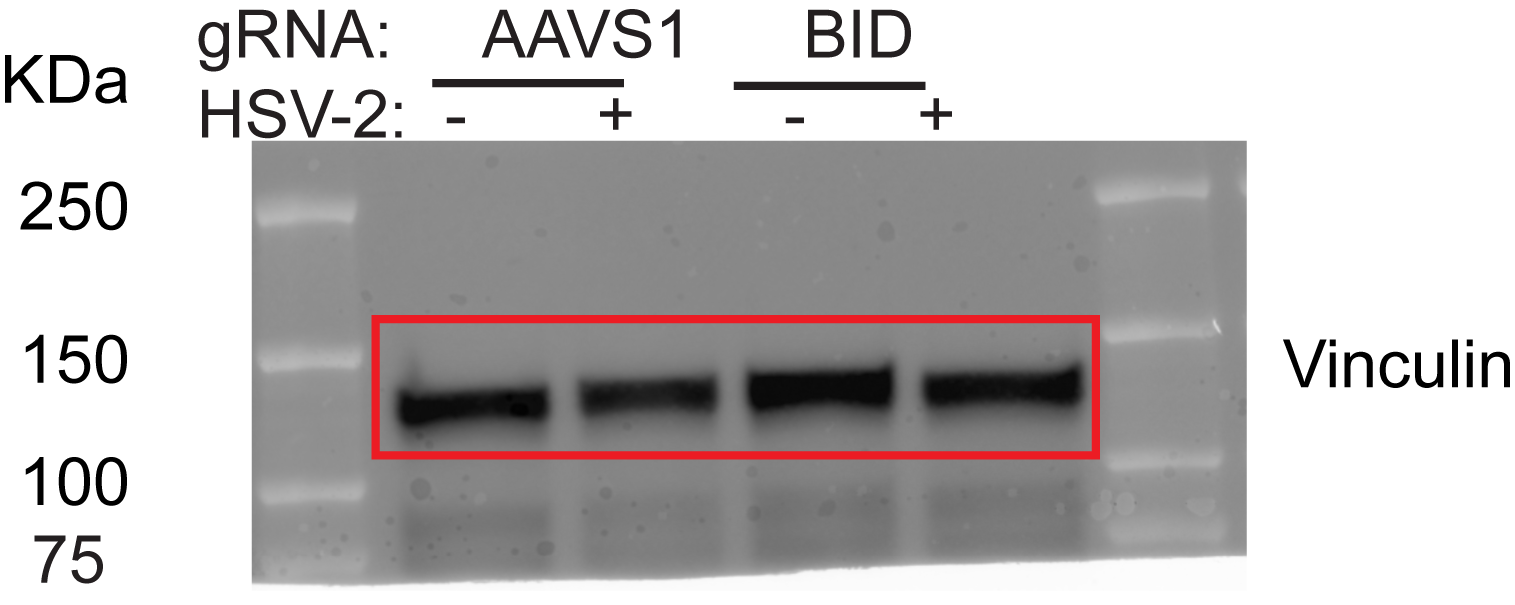

Supplement: Supplementary file 9 — Source Data for Figure 4 [file EMBJ-42-e113118-s007.zip › Source data Figure 4/4B/Western Blot Vinculin.tif]

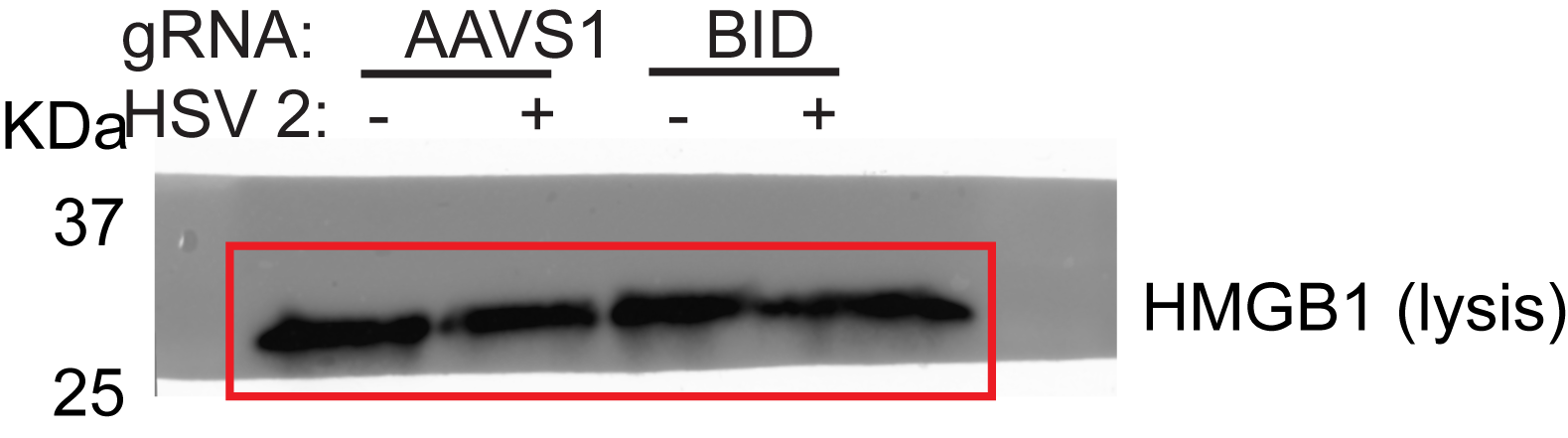

Supplement: Supplementary file 9 — Source Data for Figure 4 [file EMBJ-42-e113118-s007.zip › Source data Figure 4/4E/Western Blot HMGB1 (lysis).tif]

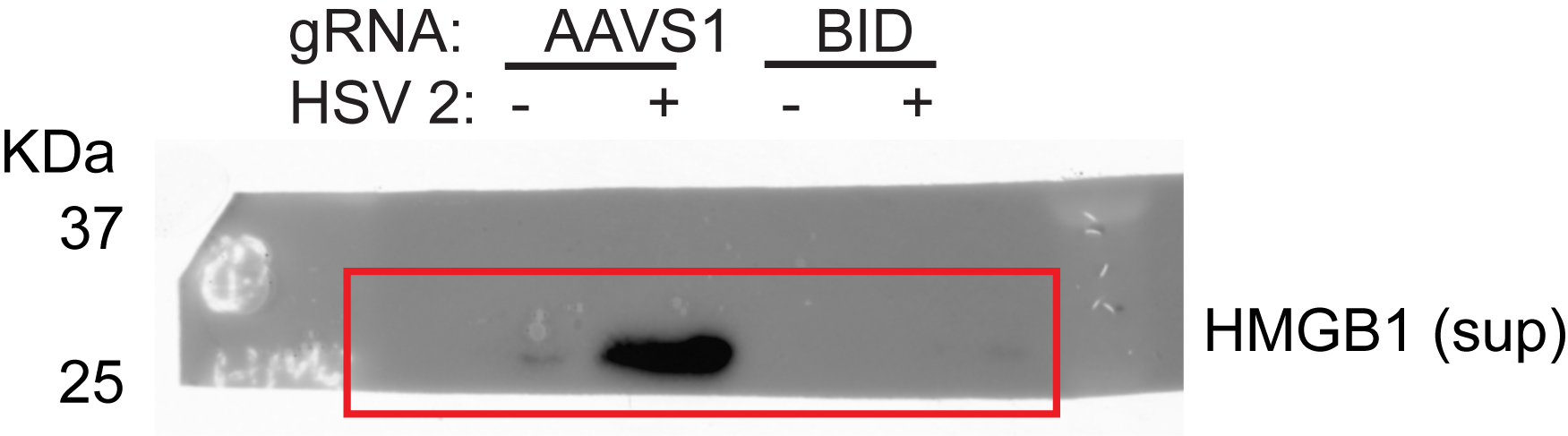

Supplement: Supplementary file 9 — Source Data for Figure 4 [file EMBJ-42-e113118-s007.zip › Source data Figure 4/4E/Western Blot HMGB1 (Sup).tif]

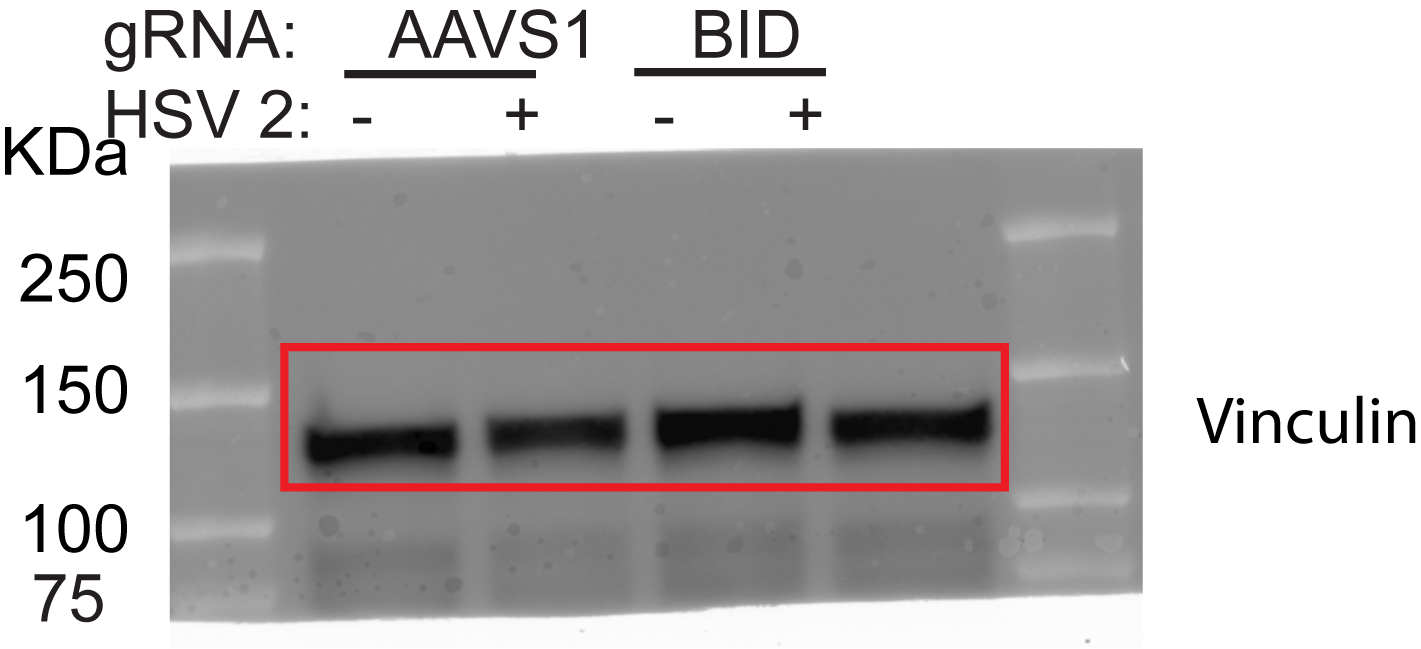

Supplement: Supplementary file 9 — Source Data for Figure 4 [file EMBJ-42-e113118-s007.zip › Source data Figure 4/4E/Western Blot Vinculin.tif]

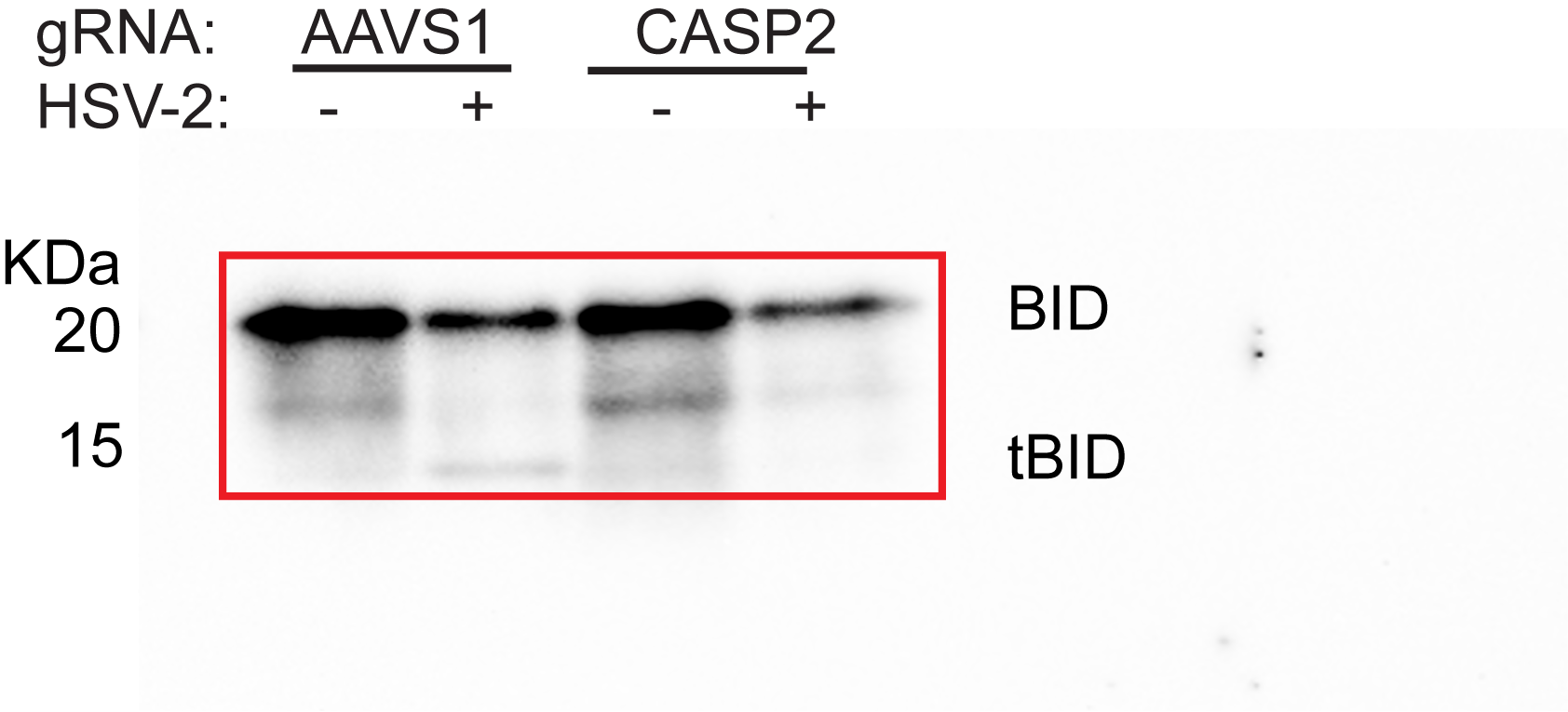

Supplement: Supplementary file 9 — Source Data for Figure 4 [file EMBJ-42-e113118-s007.zip › Source data Figure 4/4F/Western Blot BID.tif]

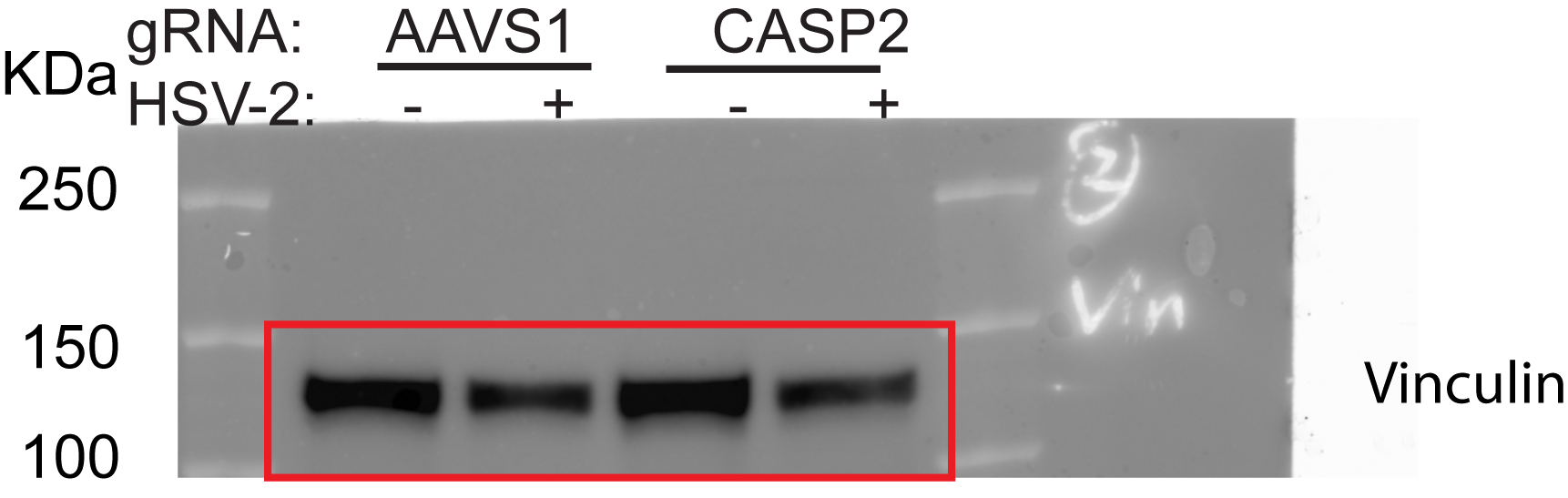

Supplement: Supplementary file 9 — Source Data for Figure 4 [file EMBJ-42-e113118-s007.zip › Source data Figure 4/4F/Western Blot Vinculin.tif]

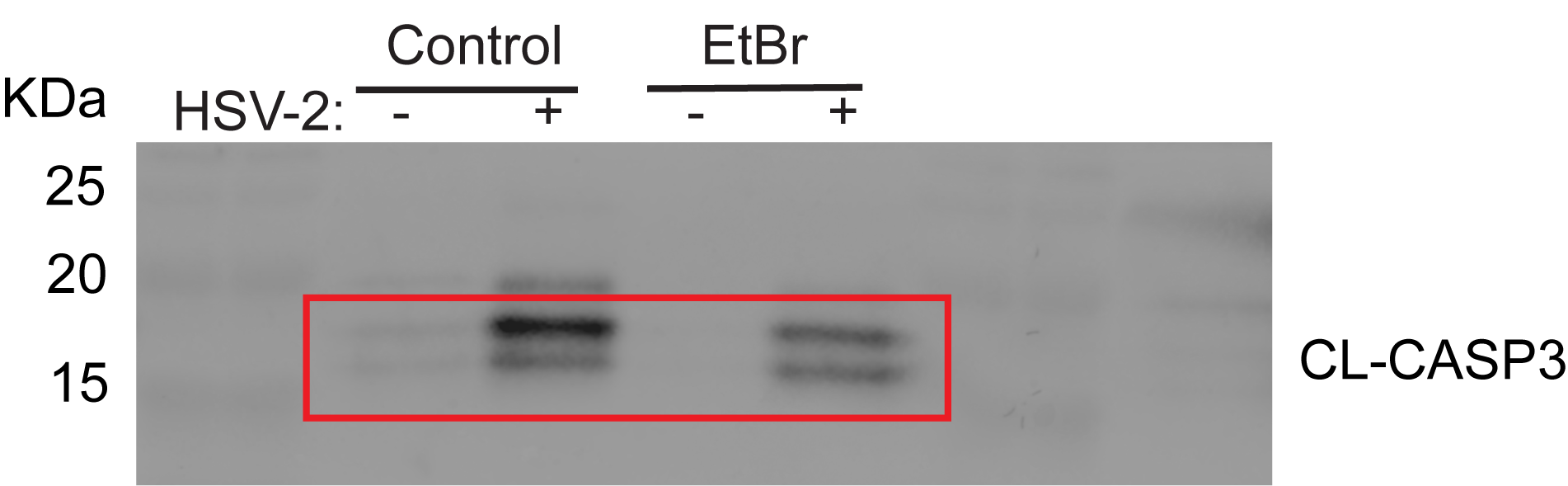

Supplement: Supplementary file 9 — Source Data for Figure 4 [file EMBJ-42-e113118-s007.zip › Source data Figure 4/4H/Western Blot CL-CASP3.tif]

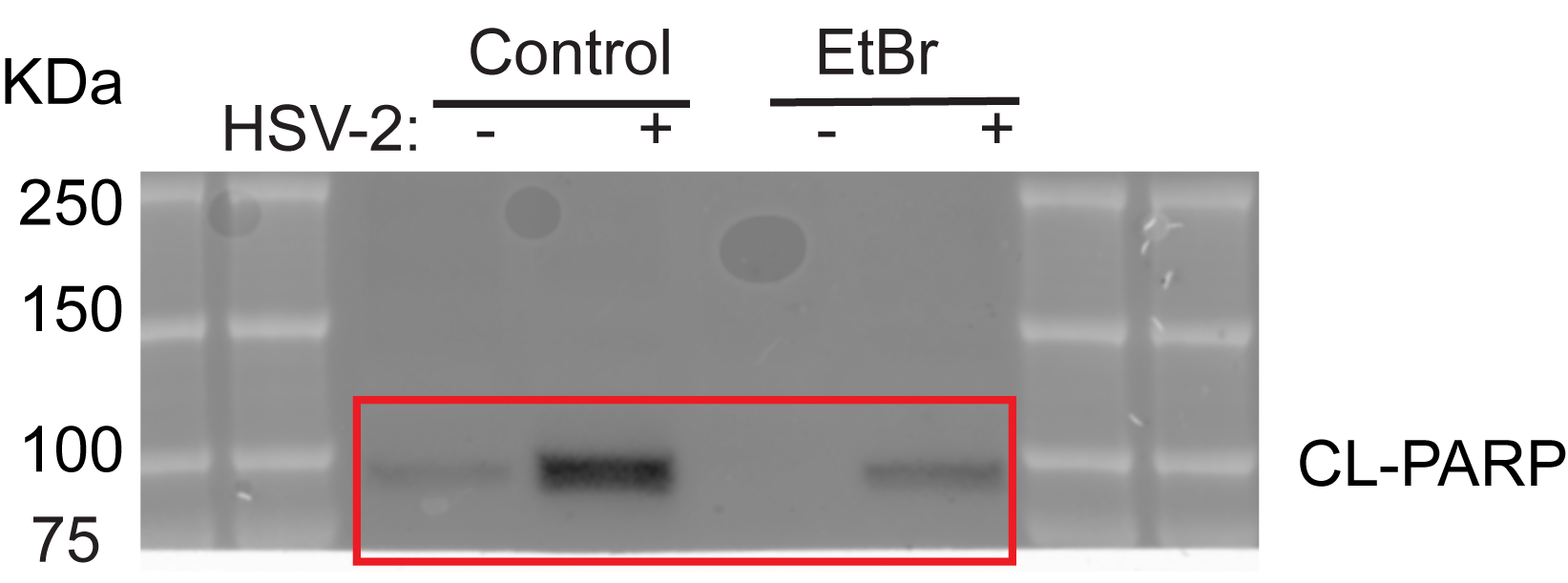

Supplement: Supplementary file 9 — Source Data for Figure 4 [file EMBJ-42-e113118-s007.zip › Source data Figure 4/4H/Western Blot CL-PARP.tif]

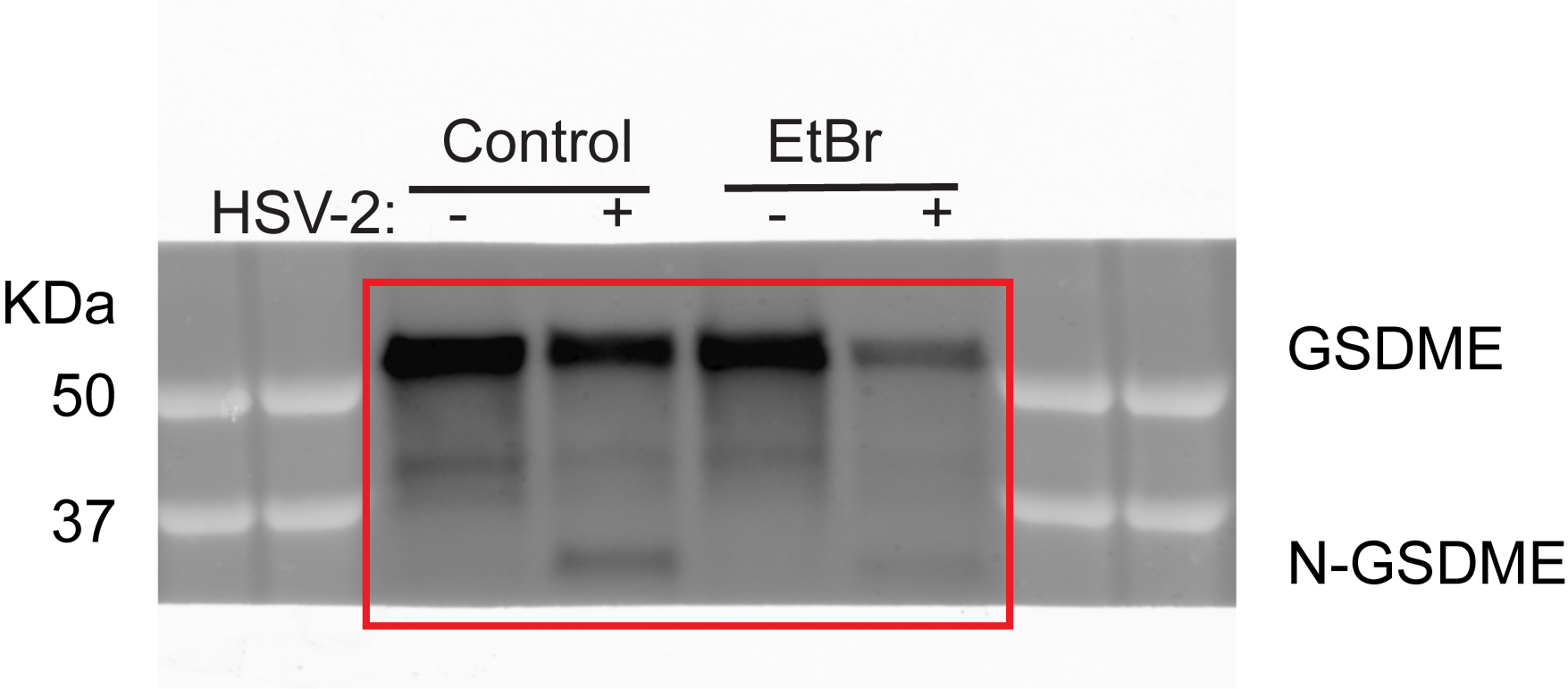

Supplement: Supplementary file 9 — Source Data for Figure 4 [file EMBJ-42-e113118-s007.zip › Source data Figure 4/4H/Western Blot GSDME.tif]

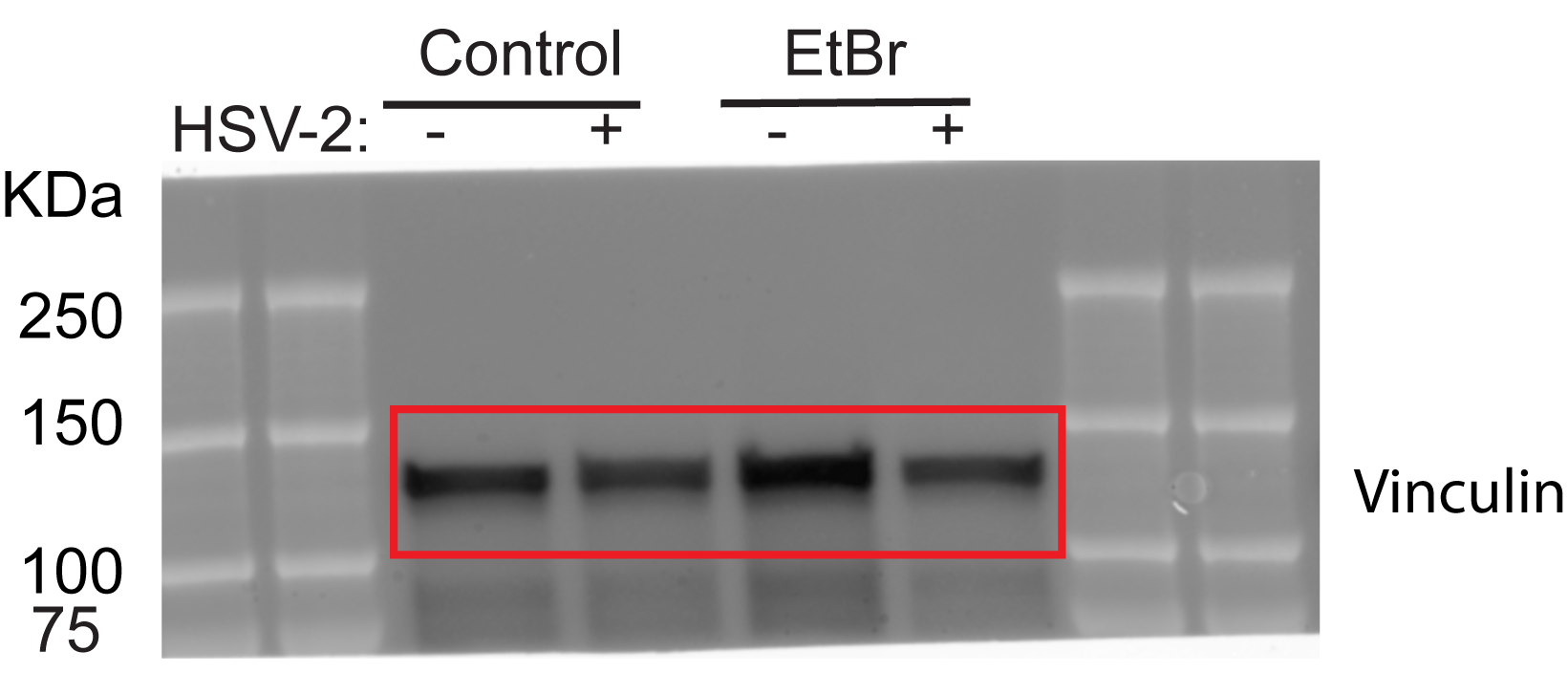

Supplement: Supplementary file 9 — Source Data for Figure 4 [file EMBJ-42-e113118-s007.zip › Source data Figure 4/4H/Western Blot Vinculin.tif]

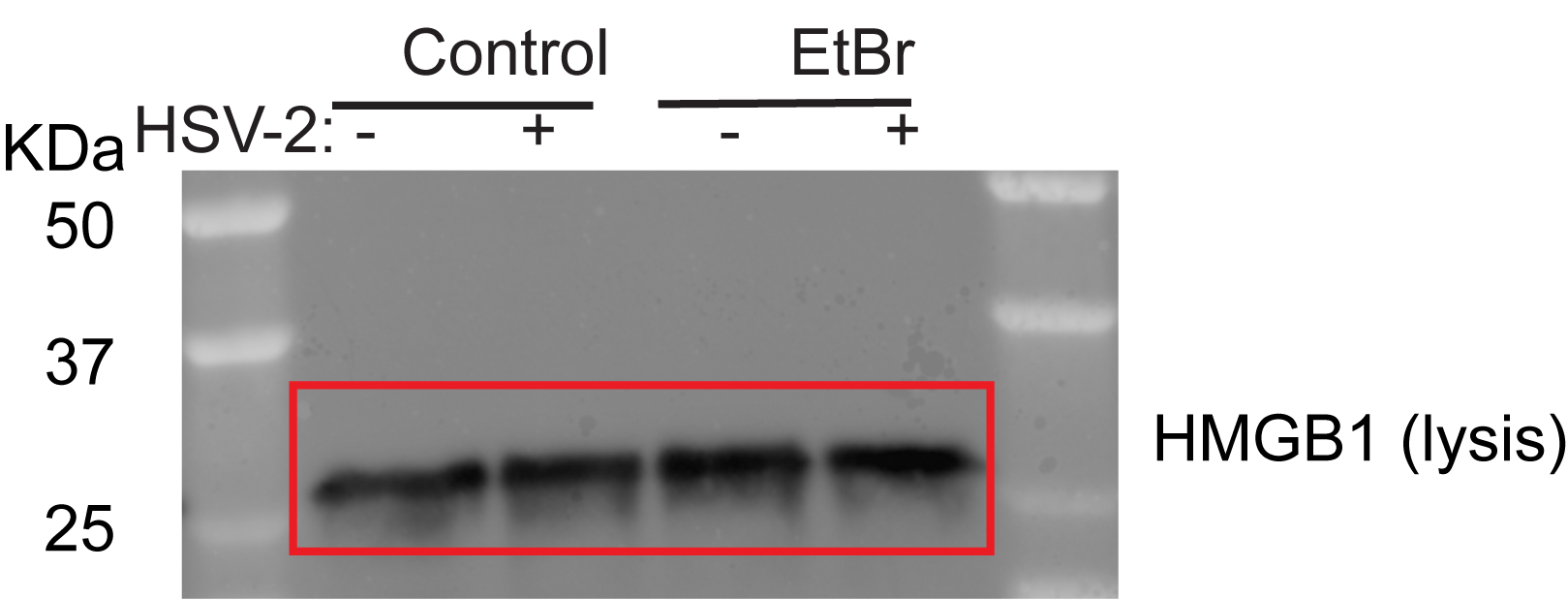

Supplement: Supplementary file 9 — Source Data for Figure 4 [file EMBJ-42-e113118-s007.zip › Source data Figure 4/4I/Western Blot HMGB1(lysis).tif]

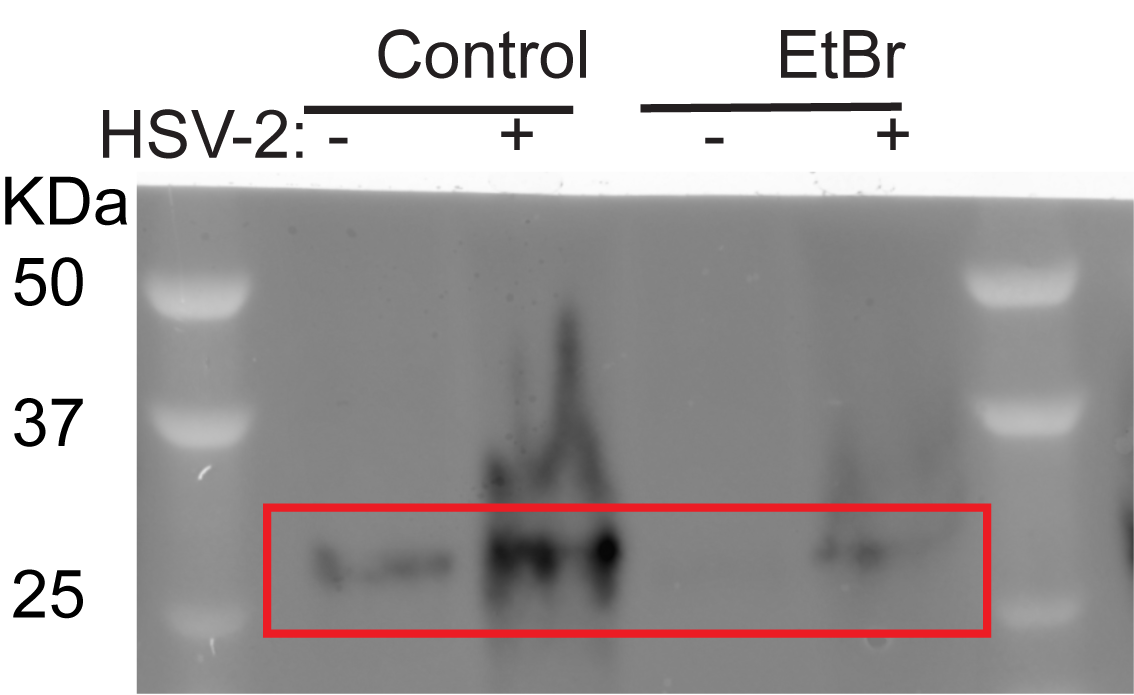

Supplement: Supplementary file 9 — Source Data for Figure 4 [file EMBJ-42-e113118-s007.zip › Source data Figure 4/4I/Western Blot HMGB1(sup).tif]

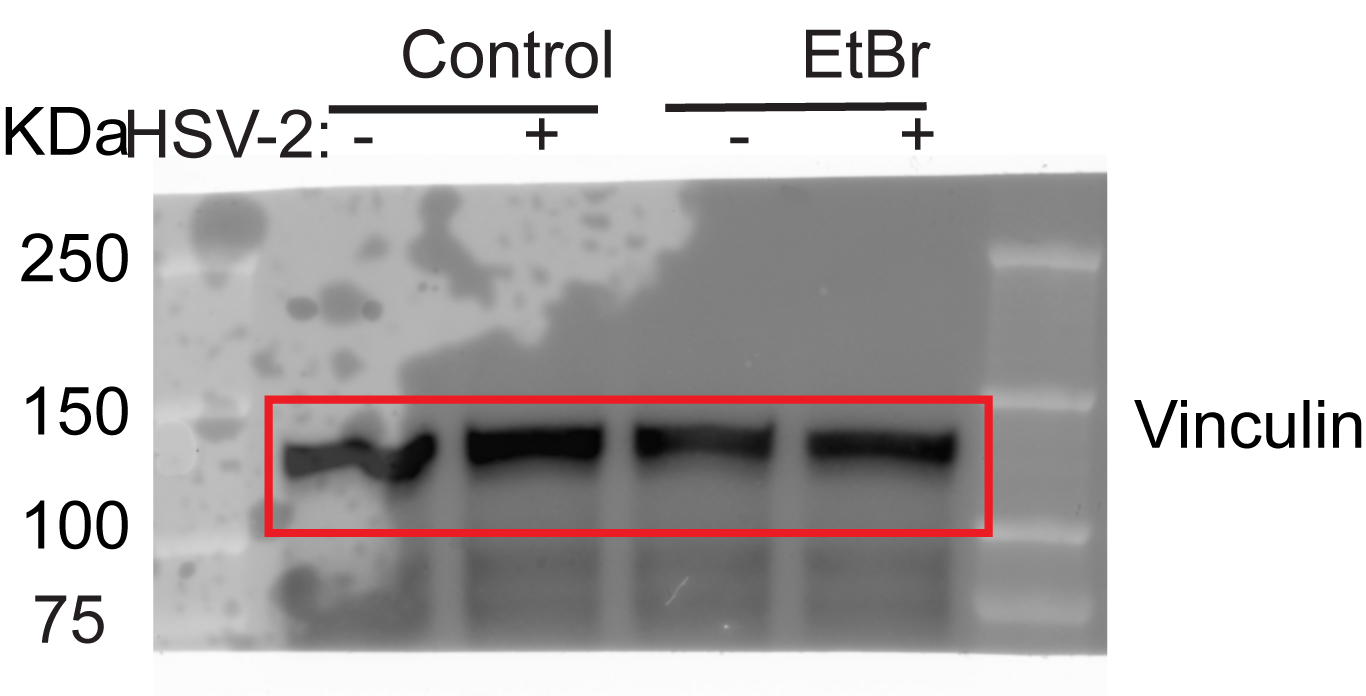

Supplement: Supplementary file 9 — Source Data for Figure 4 [file EMBJ-42-e113118-s007.zip › Source data Figure 4/4I/Western Blot Vinculin.tif]

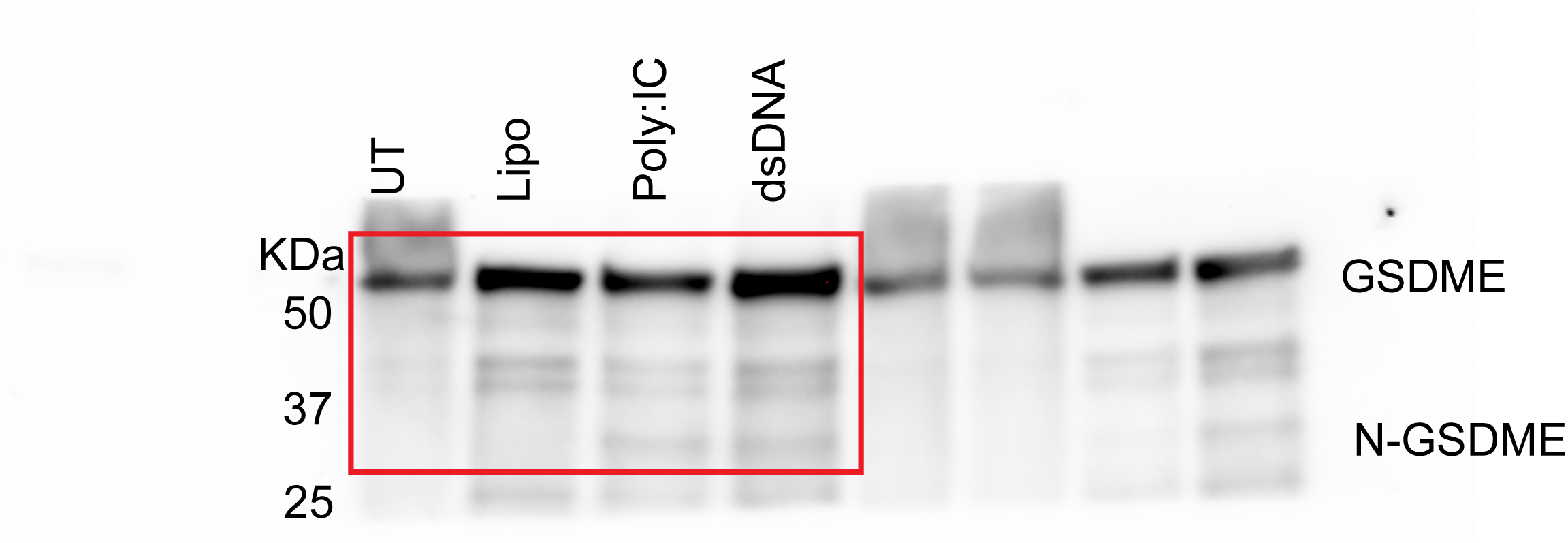

Supplement: Supplementary file 9 — Source Data for Figure 4 [file EMBJ-42-e113118-s007.zip › Source data Figure 4/4J/Western Blot GSDME .tif]

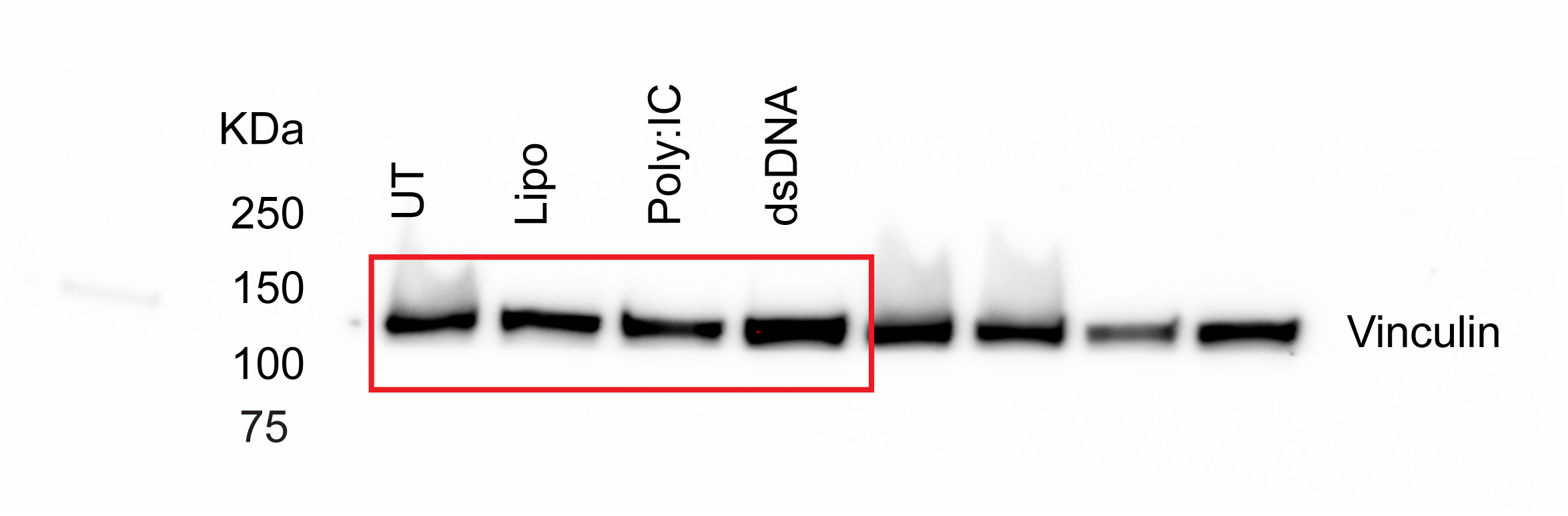

Supplement: Supplementary file 9 — Source Data for Figure 4 [file EMBJ-42-e113118-s007.zip › Source data Figure 4/4J/Western Blot Vinculin .tif]

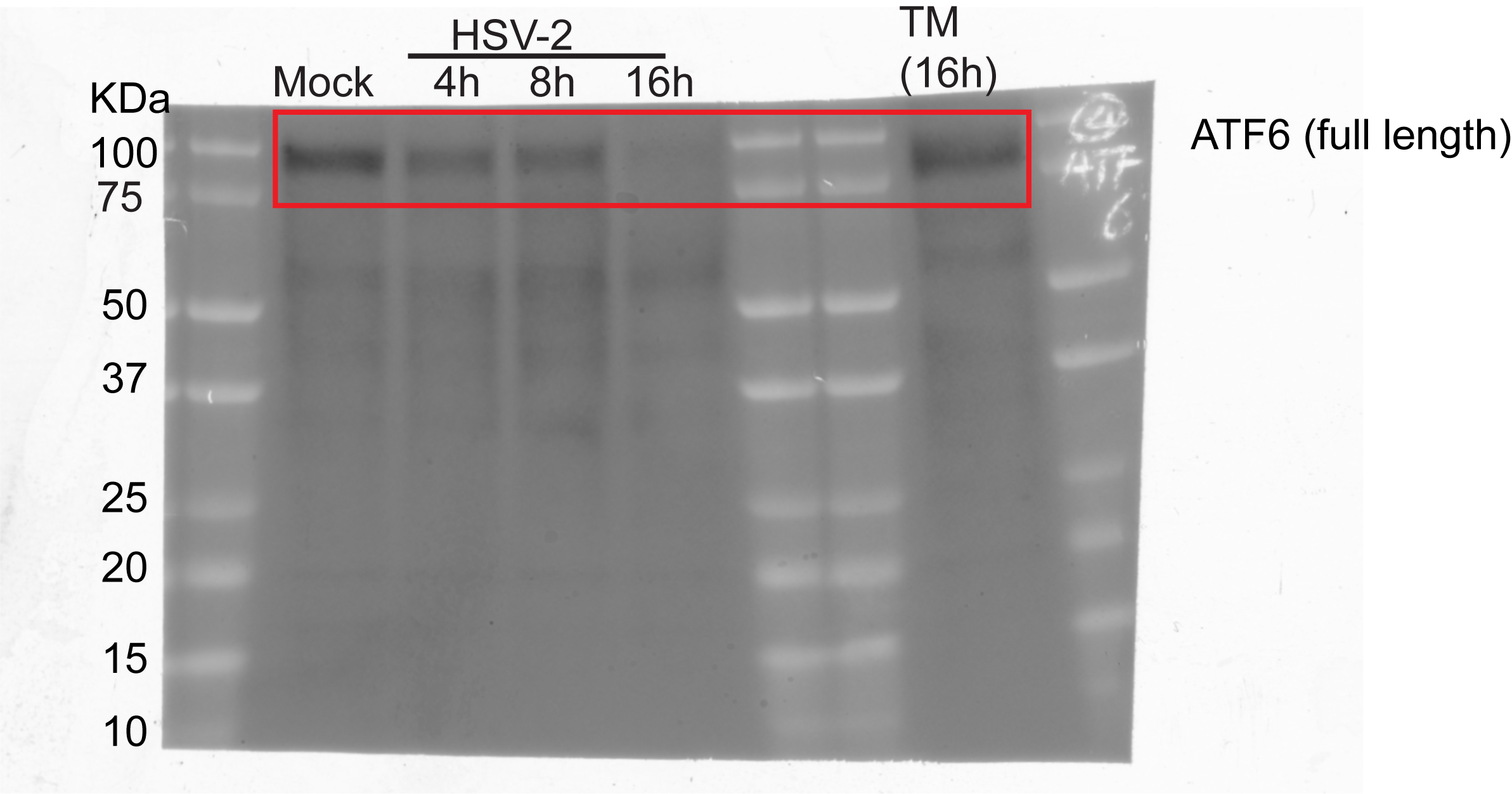

Supplement: Supplementary file 10 — Source Data for Figure 5 [file EMBJ-42-e113118-s004.zip › Source data Figure 5/5A/Western Blot ATF6.tif]

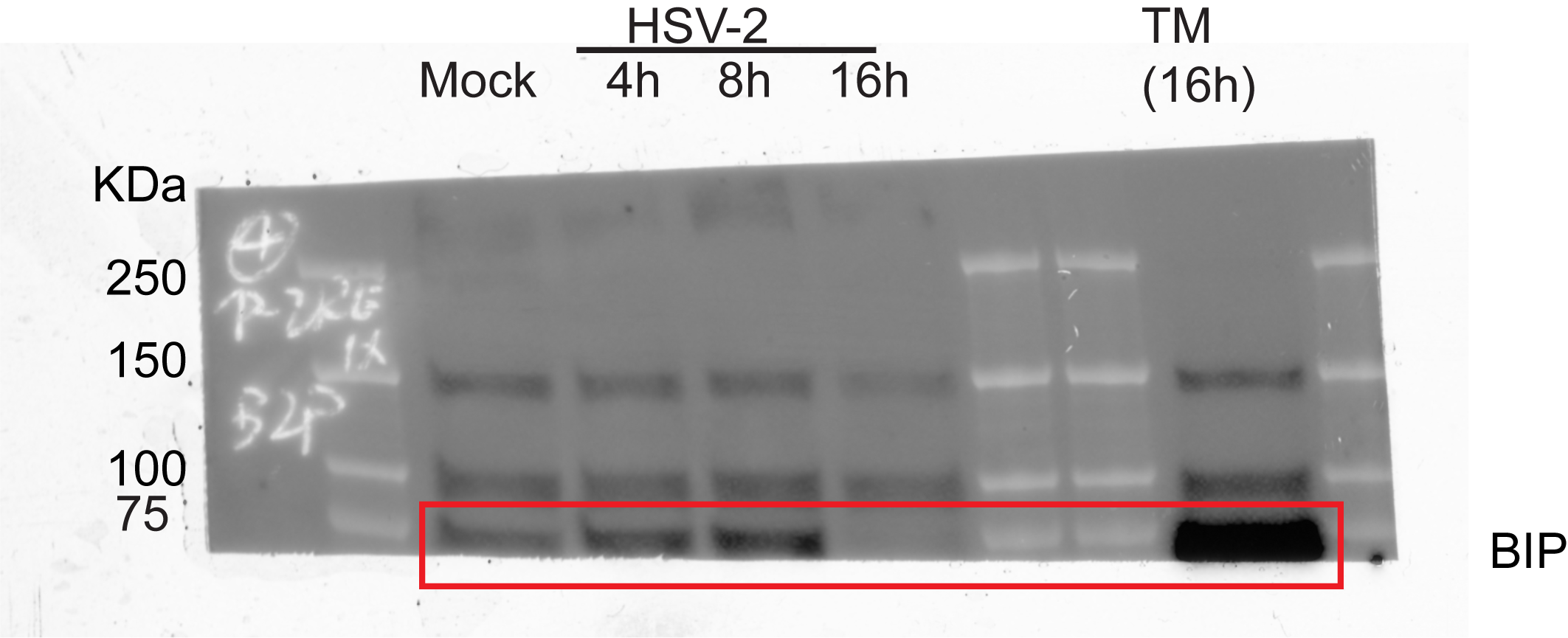

Supplement: Supplementary file 10 — Source Data for Figure 5 [file EMBJ-42-e113118-s004.zip › Source data Figure 5/5A/Western Blot BIP.tif]

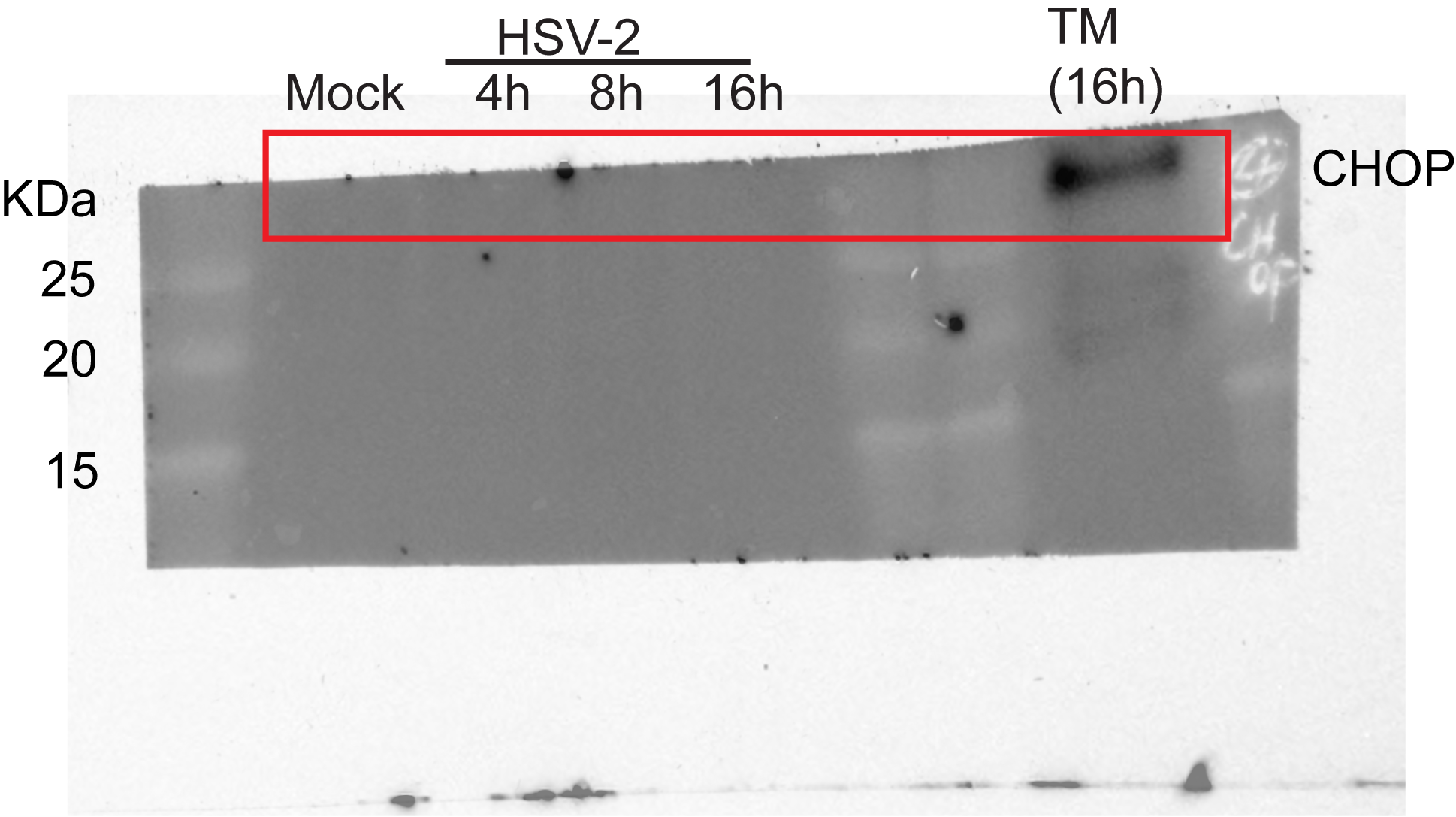

Supplement: Supplementary file 10 — Source Data for Figure 5 [file EMBJ-42-e113118-s004.zip › Source data Figure 5/5A/Western Blot CHOP.tif]

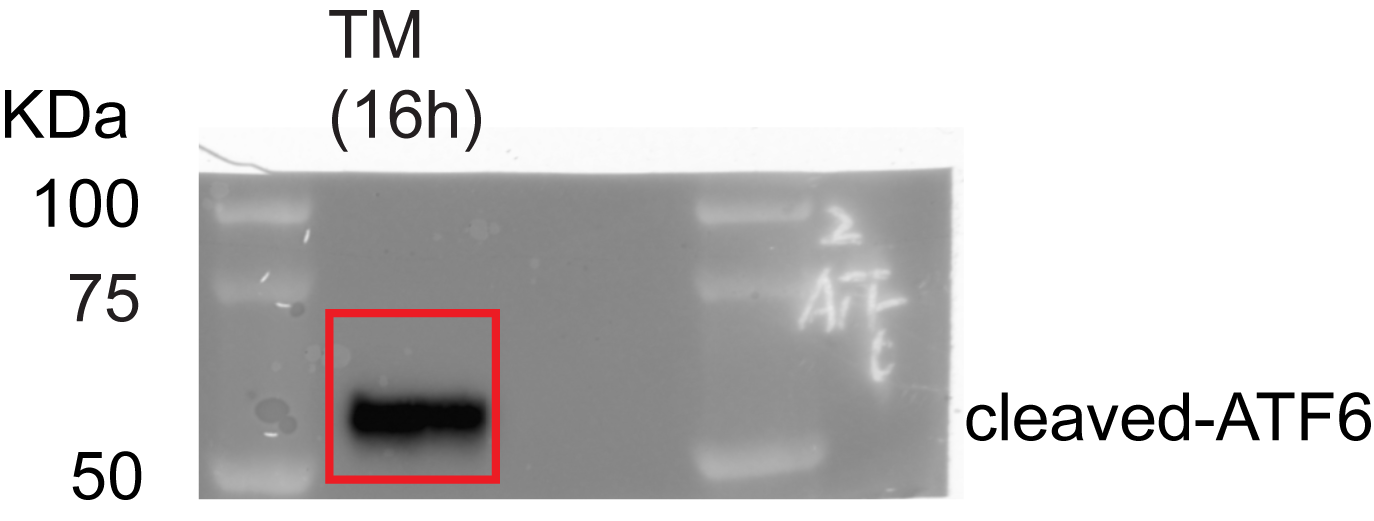

Supplement: Supplementary file 10 — Source Data for Figure 5 [file EMBJ-42-e113118-s004.zip › Source data Figure 5/5A/Western Blot cleaved ATF6.tif]

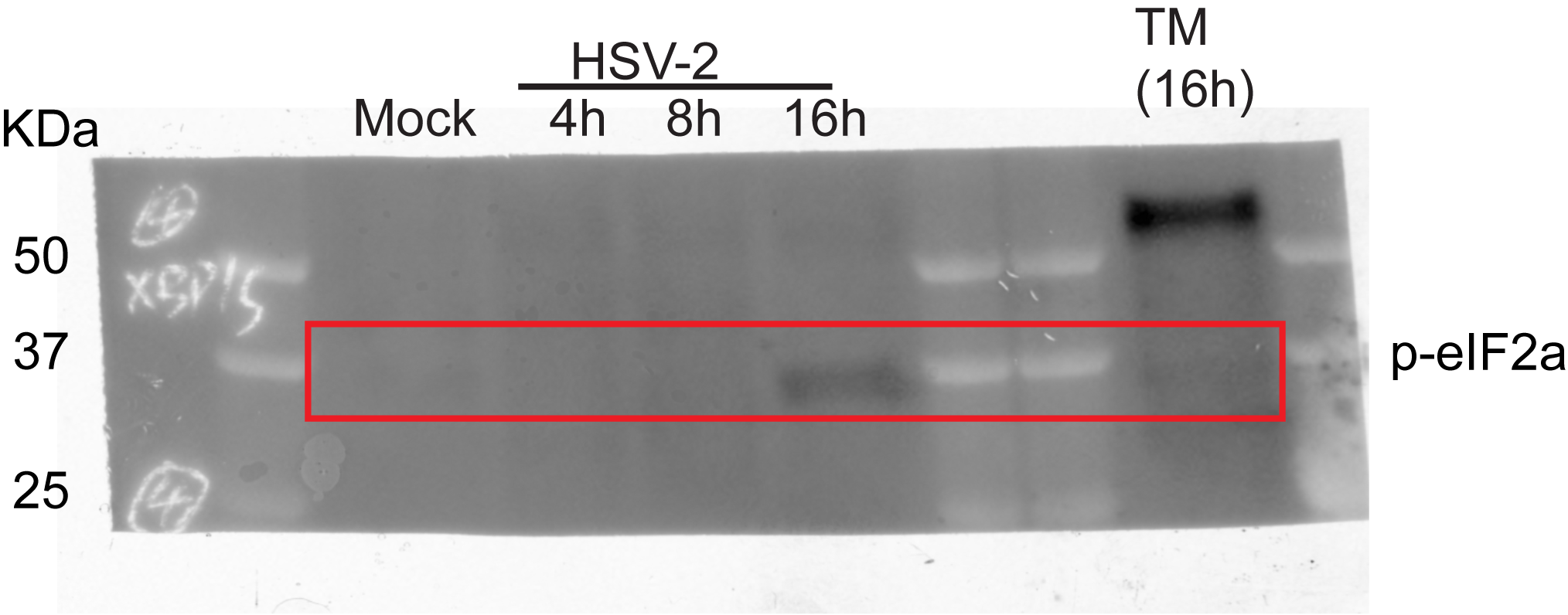

Supplement: Supplementary file 10 — Source Data for Figure 5 [file EMBJ-42-e113118-s004.zip › Source data Figure 5/5A/Western Blot p-eIF2a.tif]

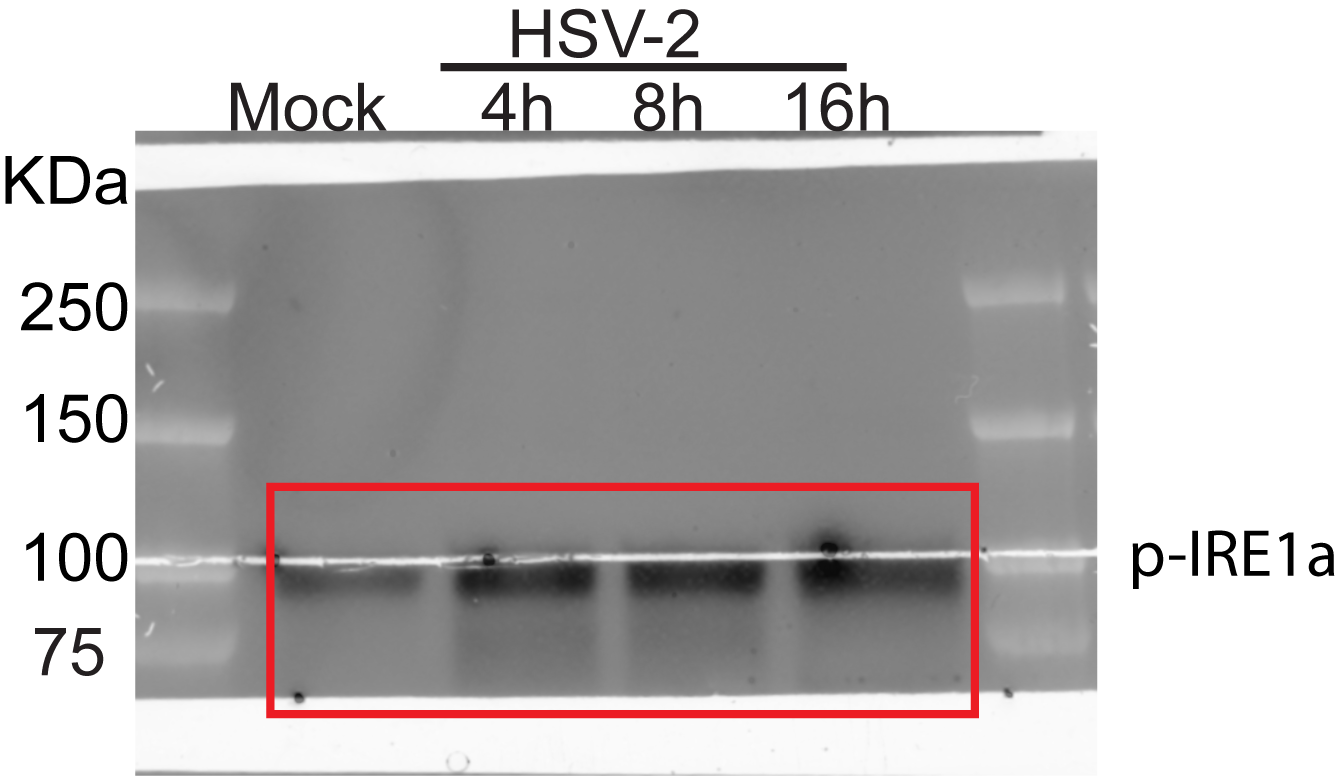

Supplement: Supplementary file 10 — Source Data for Figure 5 [file EMBJ-42-e113118-s004.zip › Source data Figure 5/5A/Western Blot p-IRE1a.tif]

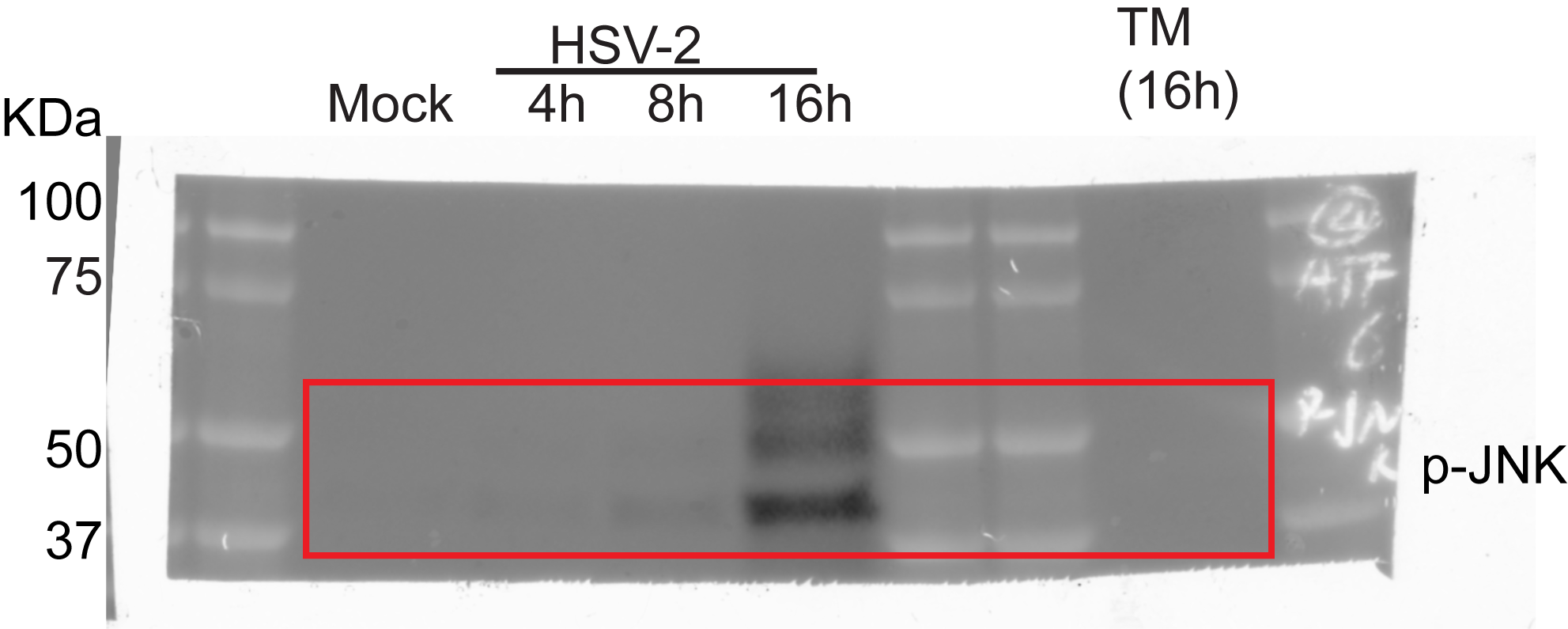

Supplement: Supplementary file 10 — Source Data for Figure 5 [file EMBJ-42-e113118-s004.zip › Source data Figure 5/5A/Western Blot p-JNK.tif]

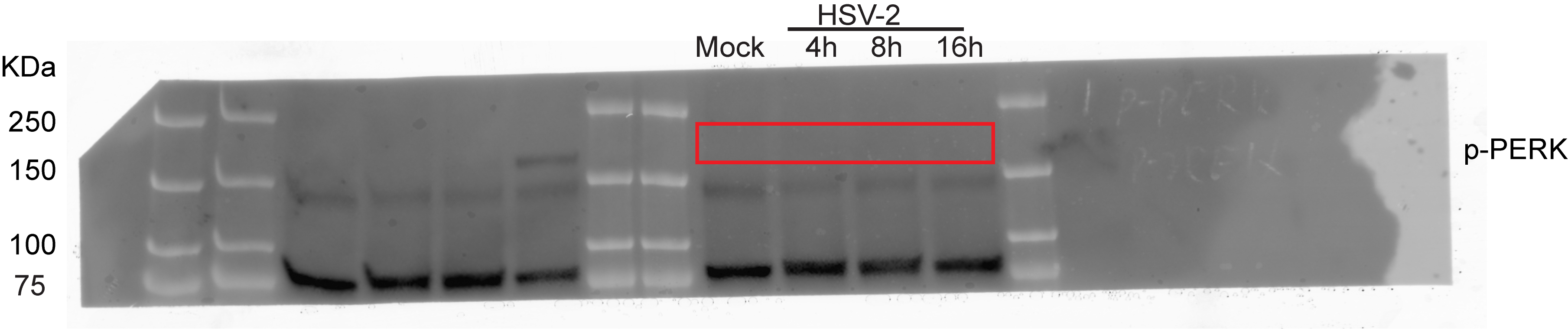

Supplement: Supplementary file 10 — Source Data for Figure 5 [file EMBJ-42-e113118-s004.zip › Source data Figure 5/5A/Western Blot p-PERK.tif]

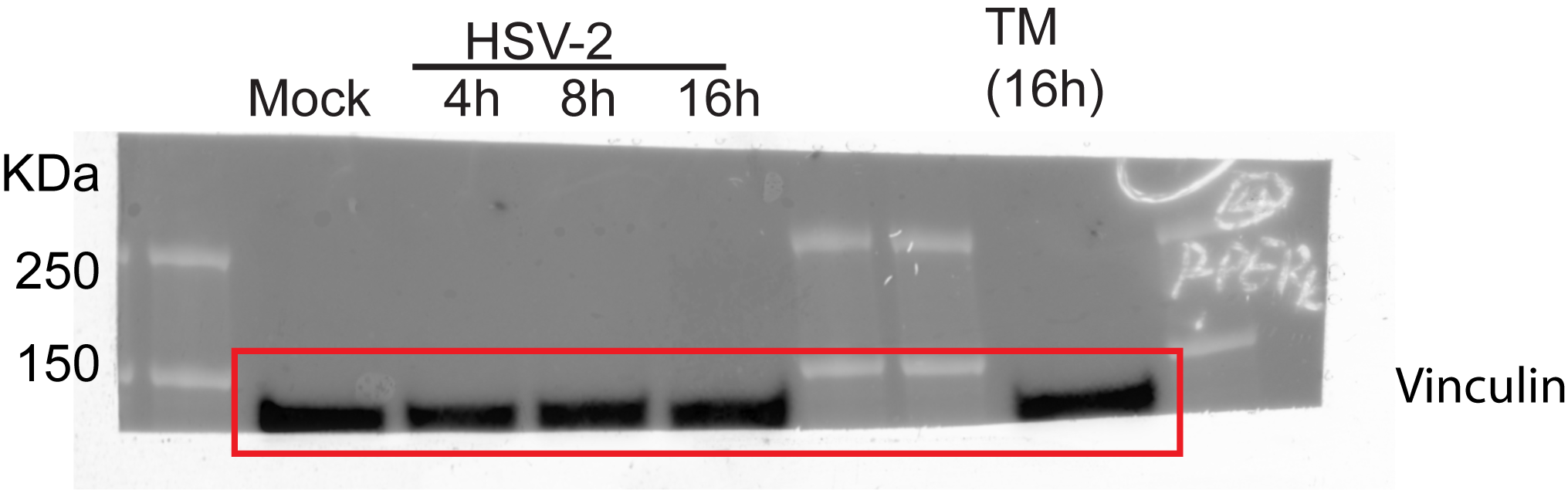

Supplement: Supplementary file 10 — Source Data for Figure 5 [file EMBJ-42-e113118-s004.zip › Source data Figure 5/5A/Western Blot Vinculin.tif]

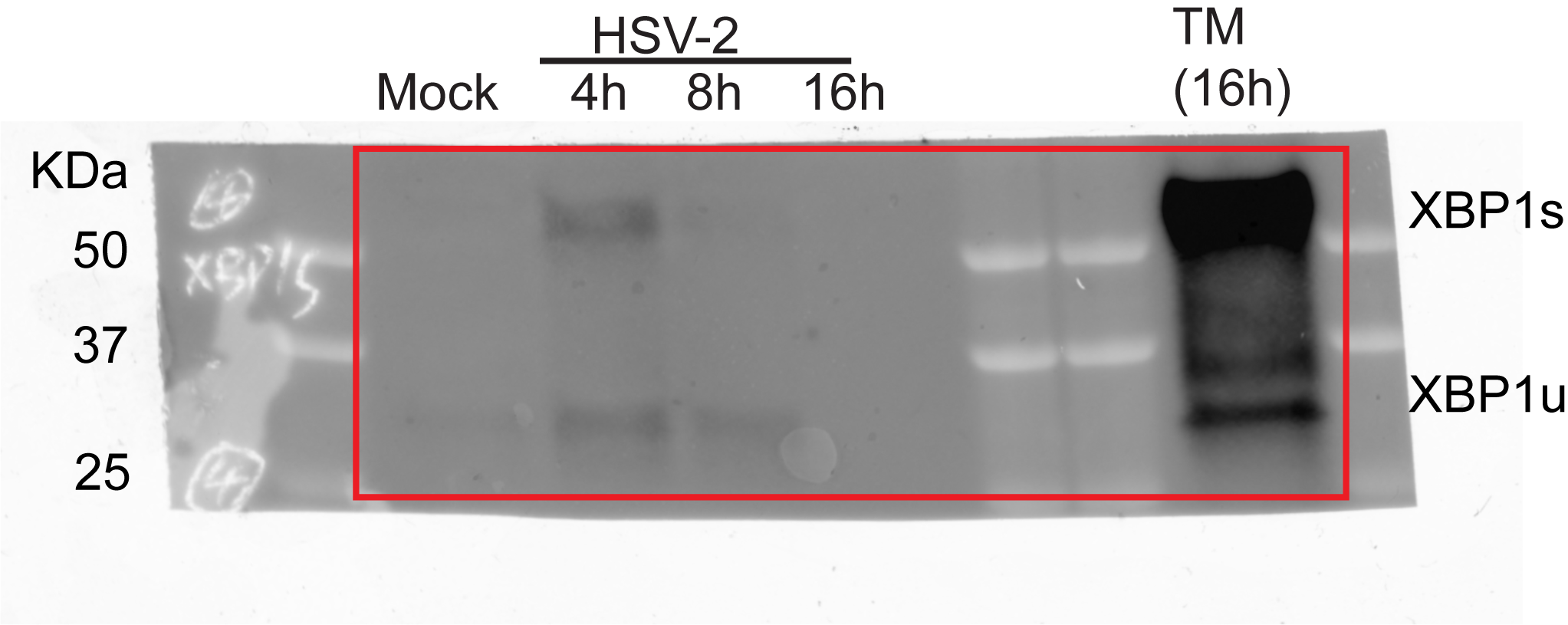

Supplement: Supplementary file 10 — Source Data for Figure 5 [file EMBJ-42-e113118-s004.zip › Source data Figure 5/5A/Western Blot XBP1s.tif]

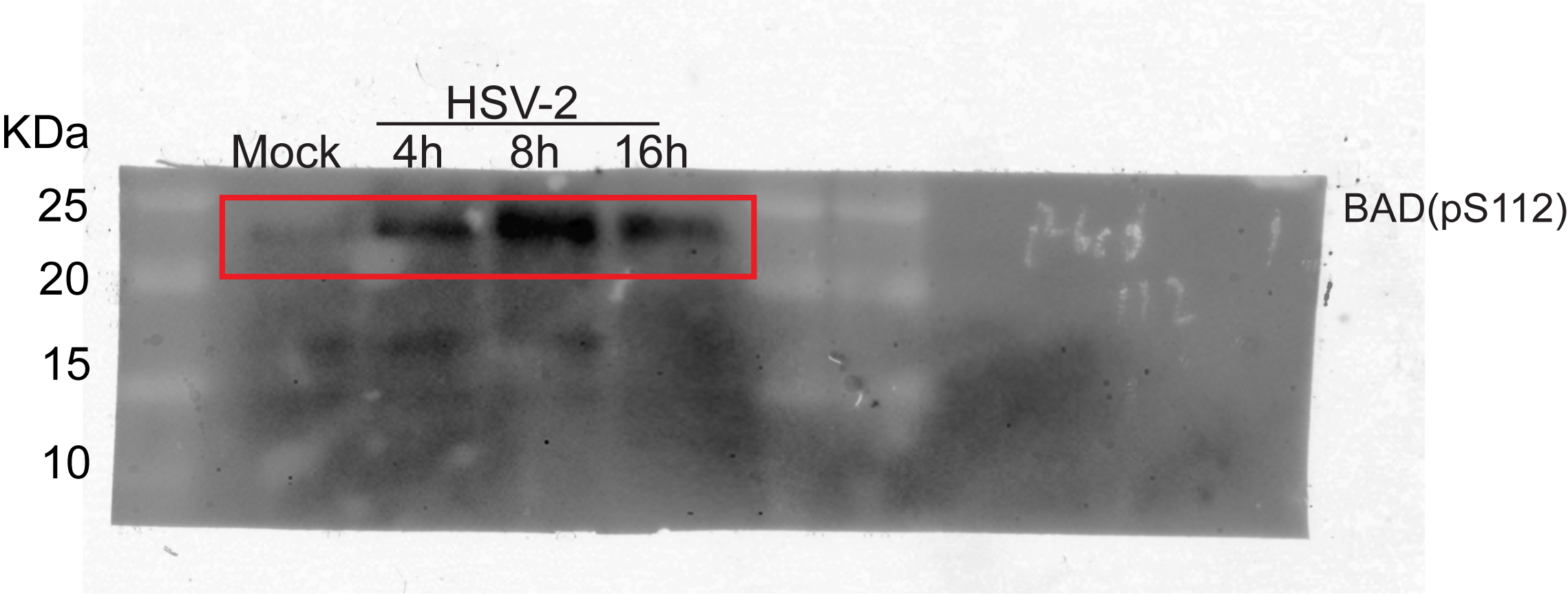

Supplement: Supplementary file 10 — Source Data for Figure 5 [file EMBJ-42-e113118-s004.zip › Source data Figure 5/5C/Western Blot BAD (pS112).tif]

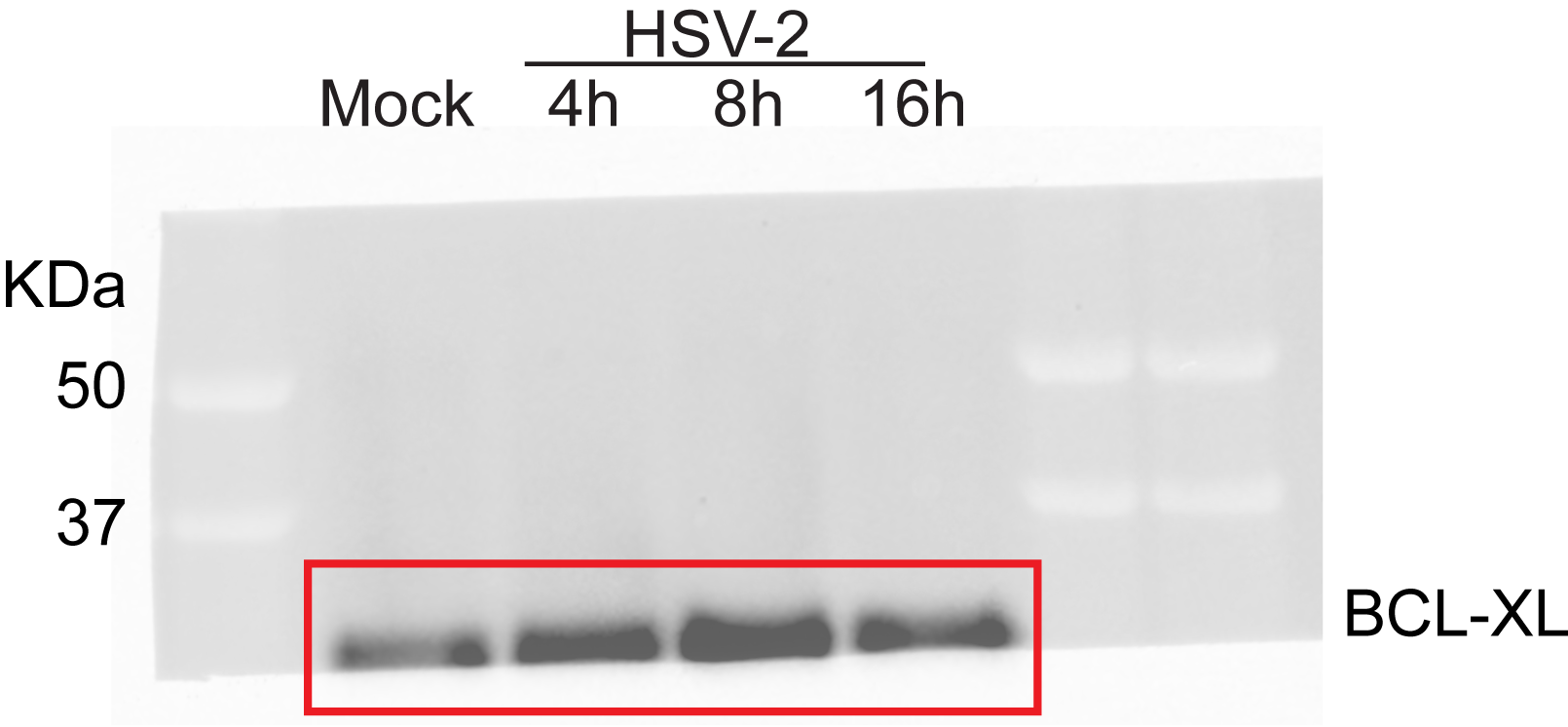

Supplement: Supplementary file 10 — Source Data for Figure 5 [file EMBJ-42-e113118-s004.zip › Source data Figure 5/5C/Western Blot BCL-XL.tif]

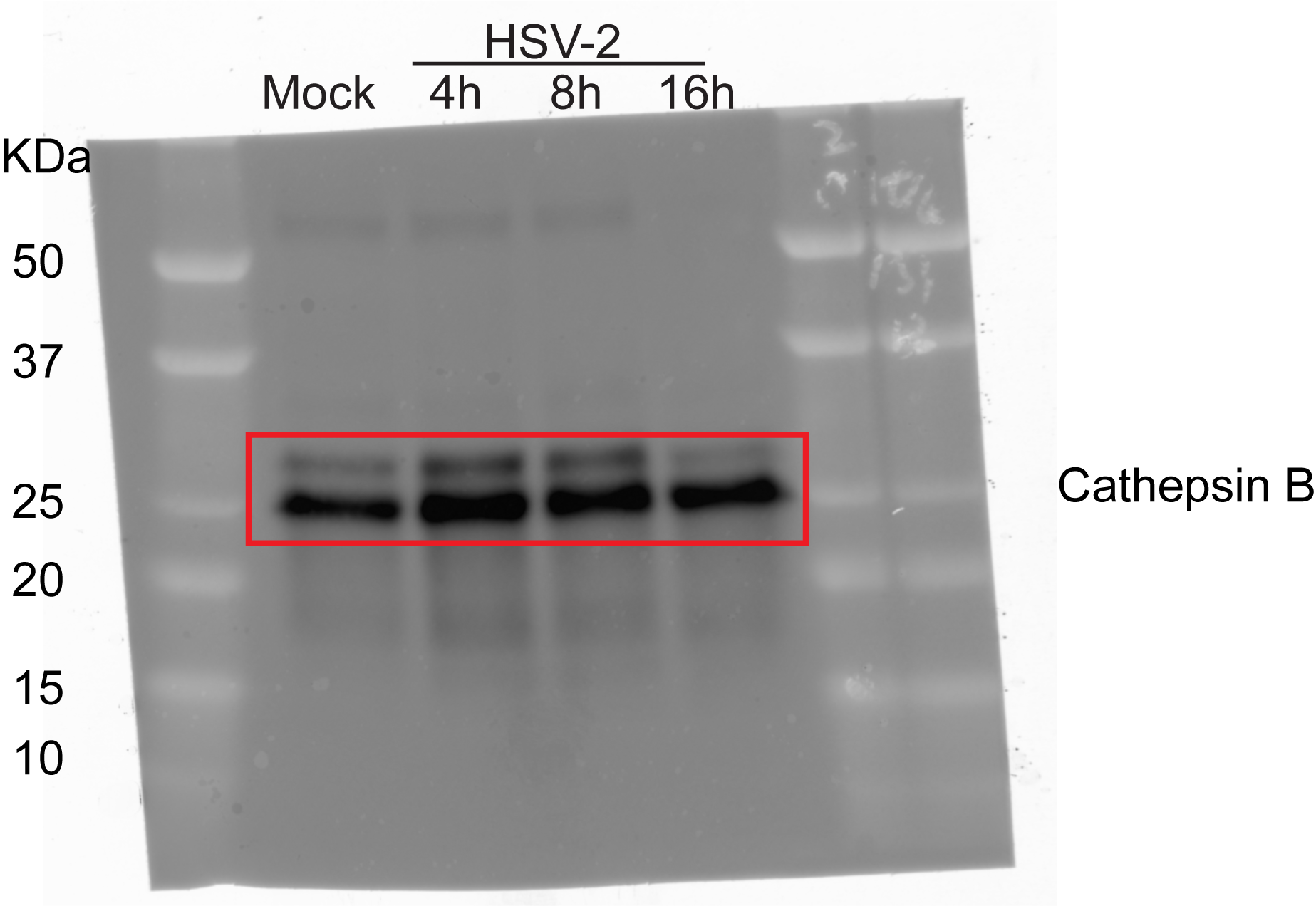

Supplement: Supplementary file 10 — Source Data for Figure 5 [file EMBJ-42-e113118-s004.zip › Source data Figure 5/5C/Western Blot Cathepsin B.tif]

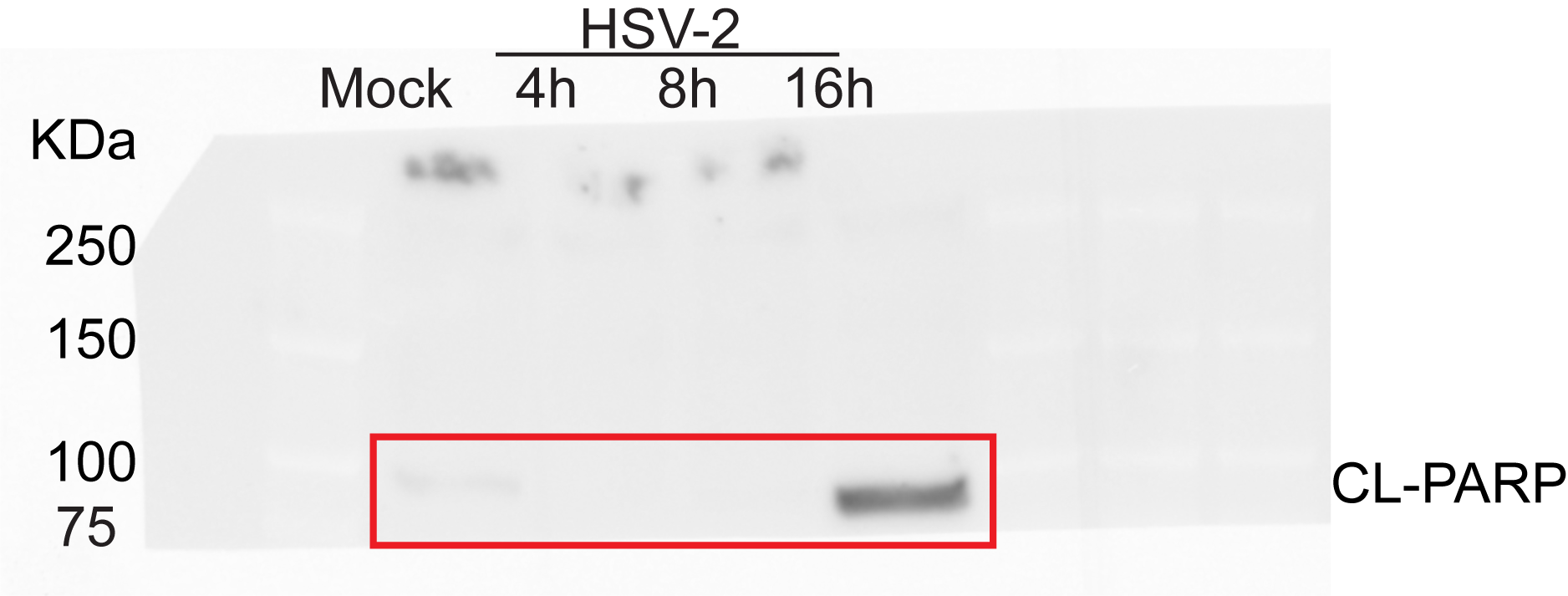

Supplement: Supplementary file 10 — Source Data for Figure 5 [file EMBJ-42-e113118-s004.zip › Source data Figure 5/5C/Western Blot CL-PARP.tif]

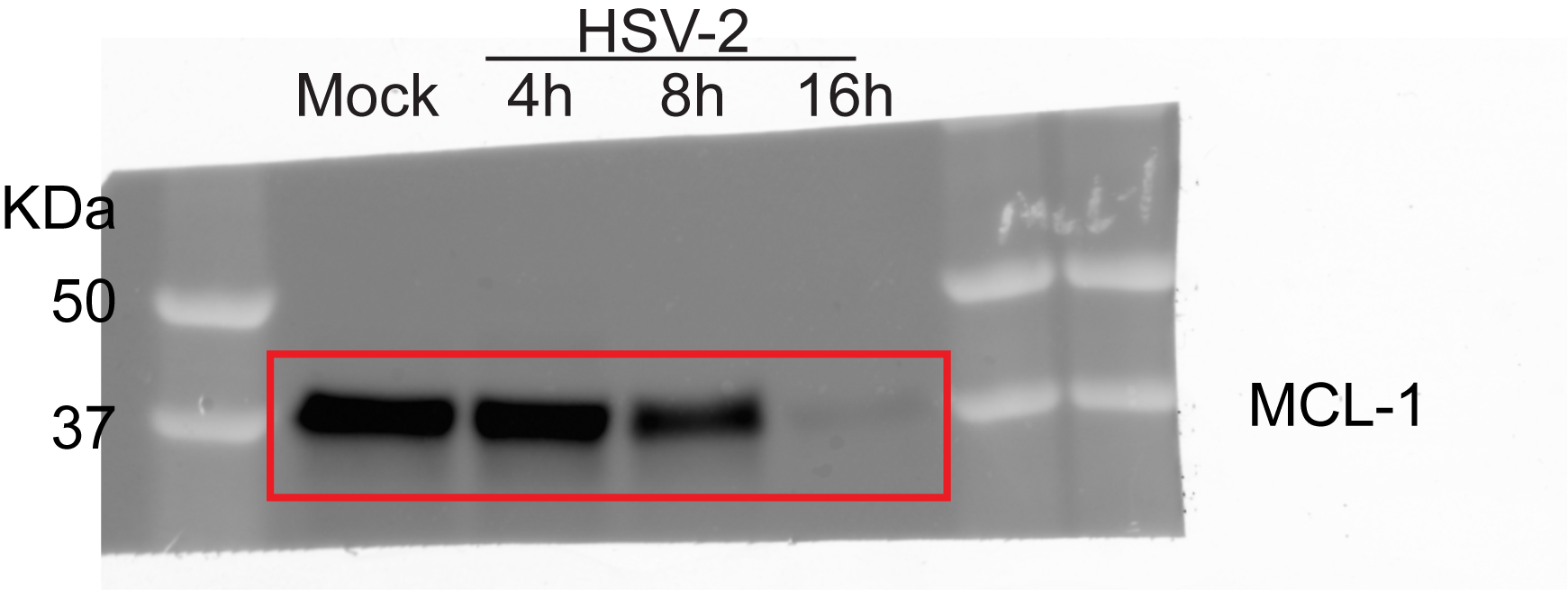

Supplement: Supplementary file 10 — Source Data for Figure 5 [file EMBJ-42-e113118-s004.zip › Source data Figure 5/5C/Western Blot MCL-1.tif]
