# Supplementary figures and images for: ER stress induces caspase‐2‐tBID‐GSDME‐dependent cell death in neurons lytically infected with herpes simplex virus type 2 (part 2 of 2)
Source: EMBO J. 2023 Aug 30;42(19):e113118. doi: 10.15252/embj.2022113118 (PMC10548179; doi:10.15252/embj.2022113118)

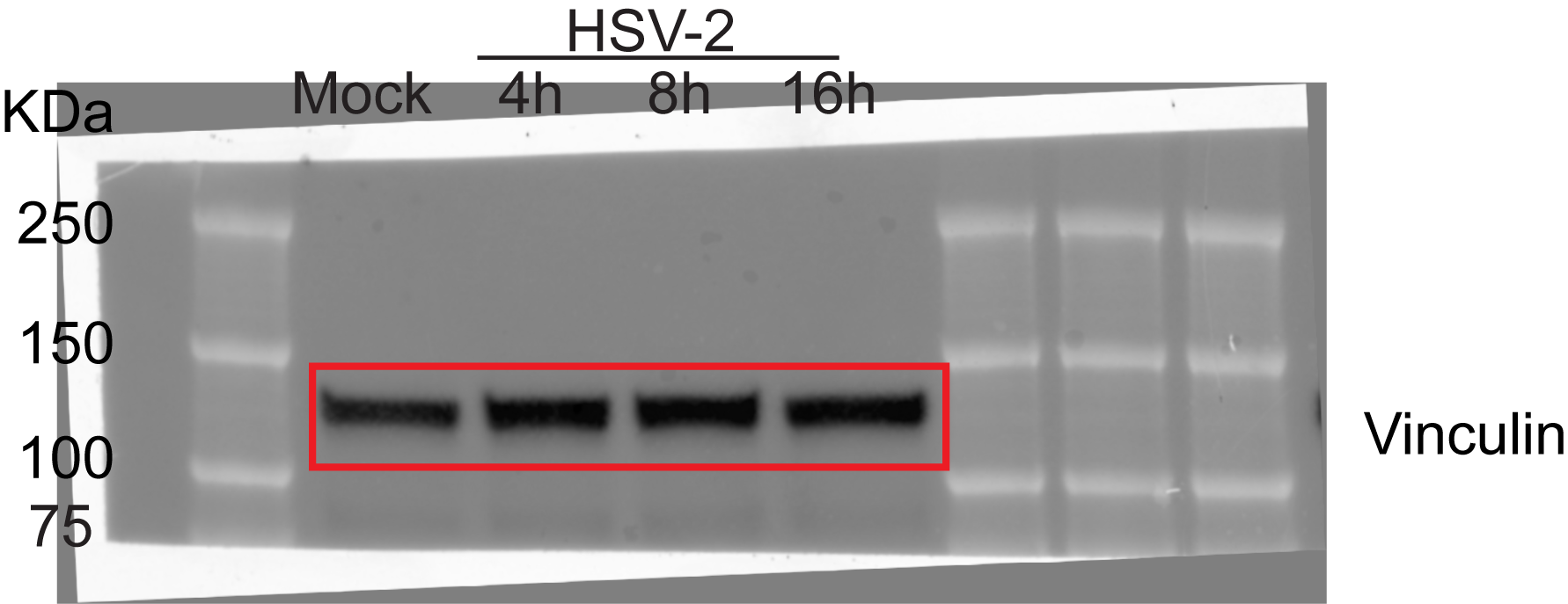

Supplement: Supplementary file 10 — Source Data for Figure 5 [file EMBJ-42-e113118-s004.zip › Source data Figure 5/5C/Western Blot Vinculin.tif]

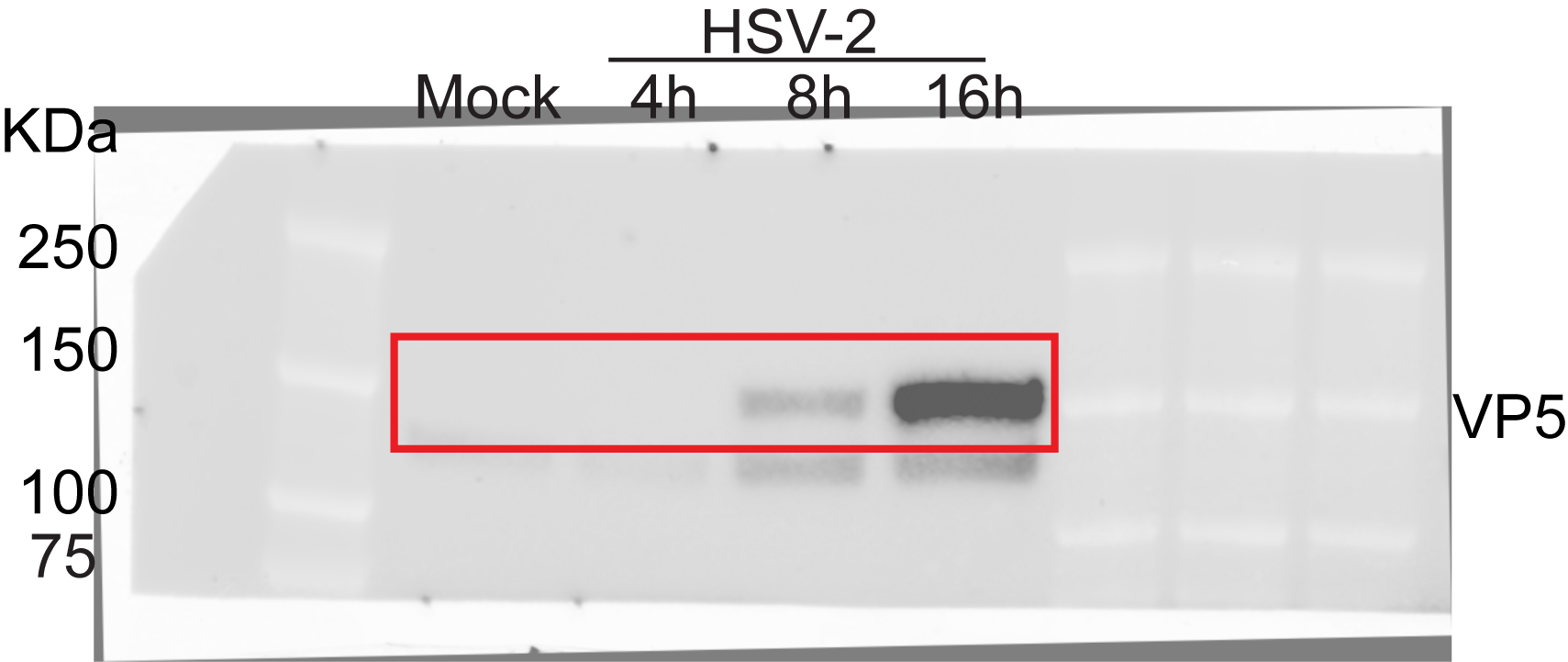

Supplement: Supplementary file 10 — Source Data for Figure 5 [file EMBJ-42-e113118-s004.zip › Source data Figure 5/5C/Western Blot VP5.tif]

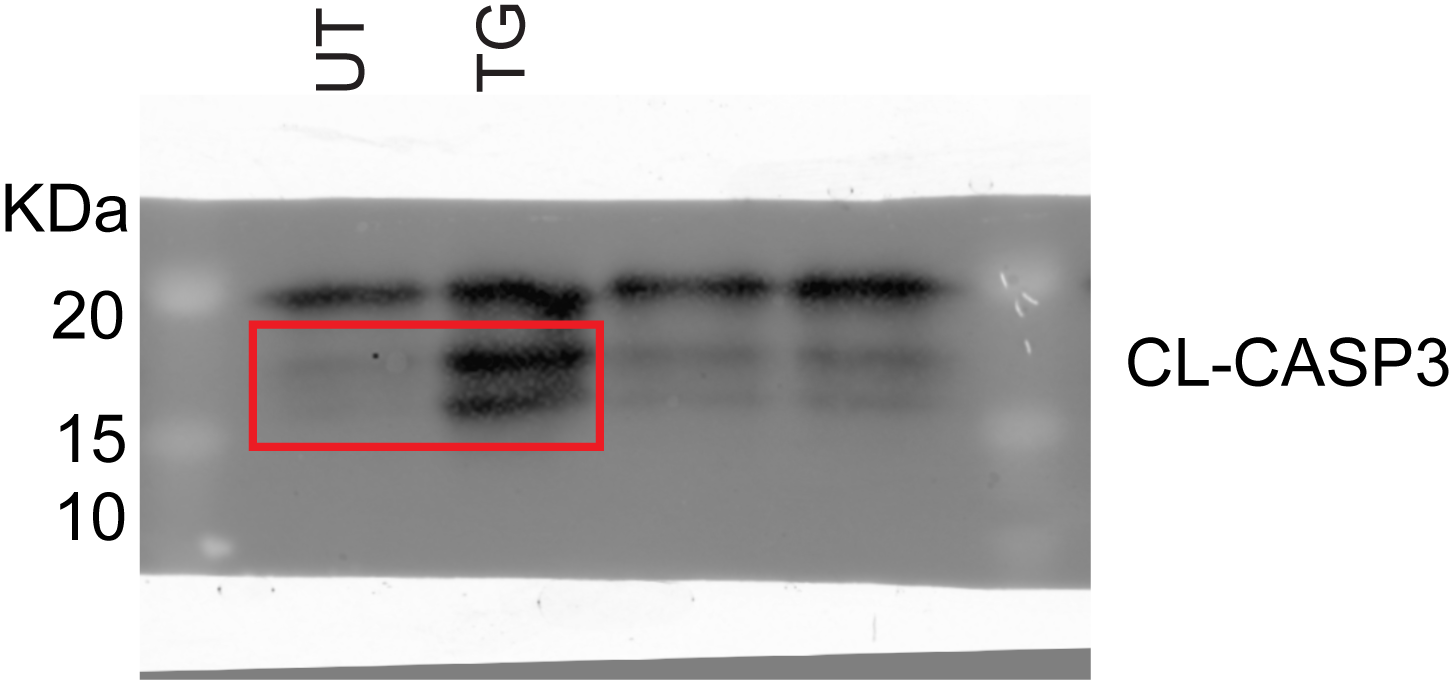

Supplement: Supplementary file 10 — Source Data for Figure 5 [file EMBJ-42-e113118-s004.zip › Source data Figure 5/5E/Western Blot CL-CASP3.tif]

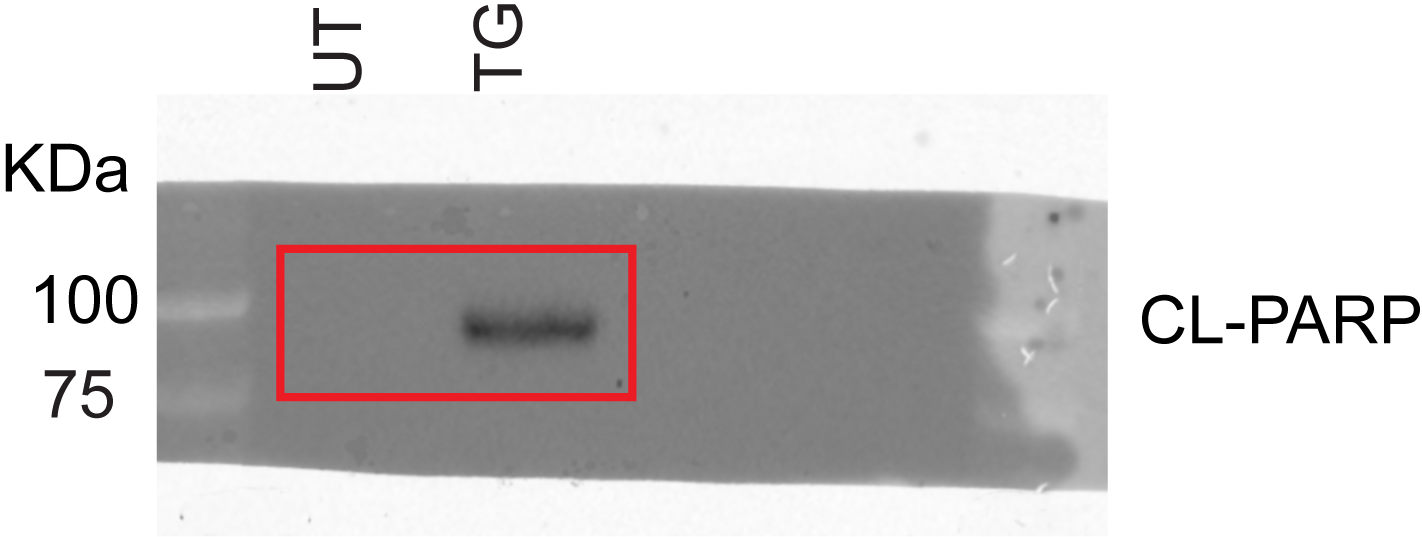

Supplement: Supplementary file 10 — Source Data for Figure 5 [file EMBJ-42-e113118-s004.zip › Source data Figure 5/5E/Western Blot CL-PARP.tif]

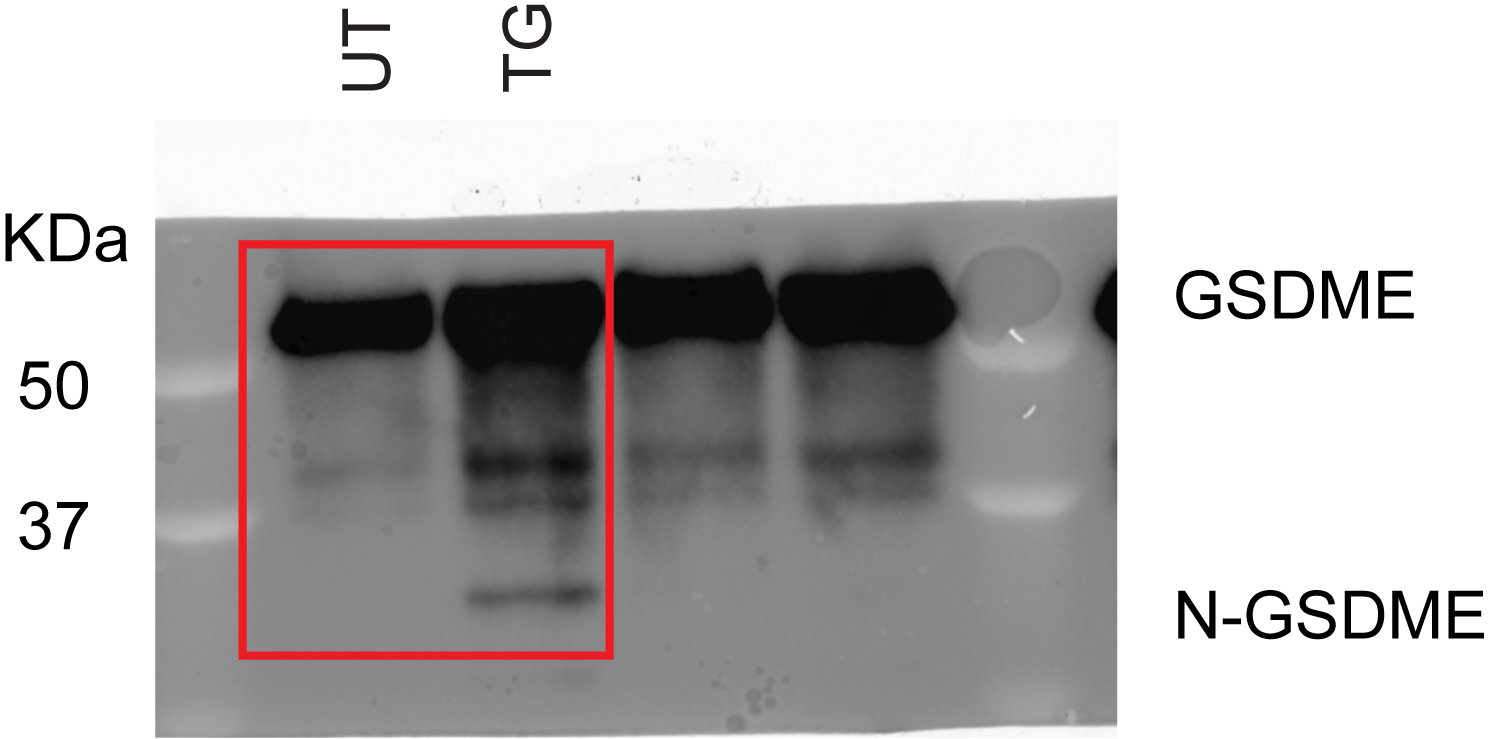

Supplement: Supplementary file 10 — Source Data for Figure 5 [file EMBJ-42-e113118-s004.zip › Source data Figure 5/5E/Western Blot GSDME.tif]

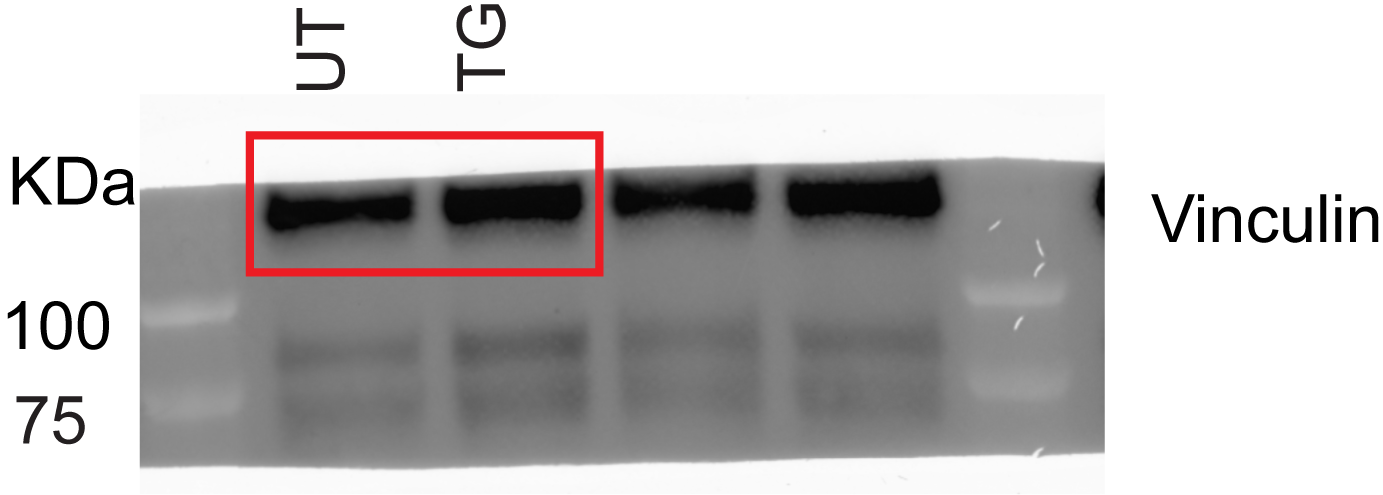

Supplement: Supplementary file 10 — Source Data for Figure 5 [file EMBJ-42-e113118-s004.zip › Source data Figure 5/5E/Western Blot Vinculin.tif]

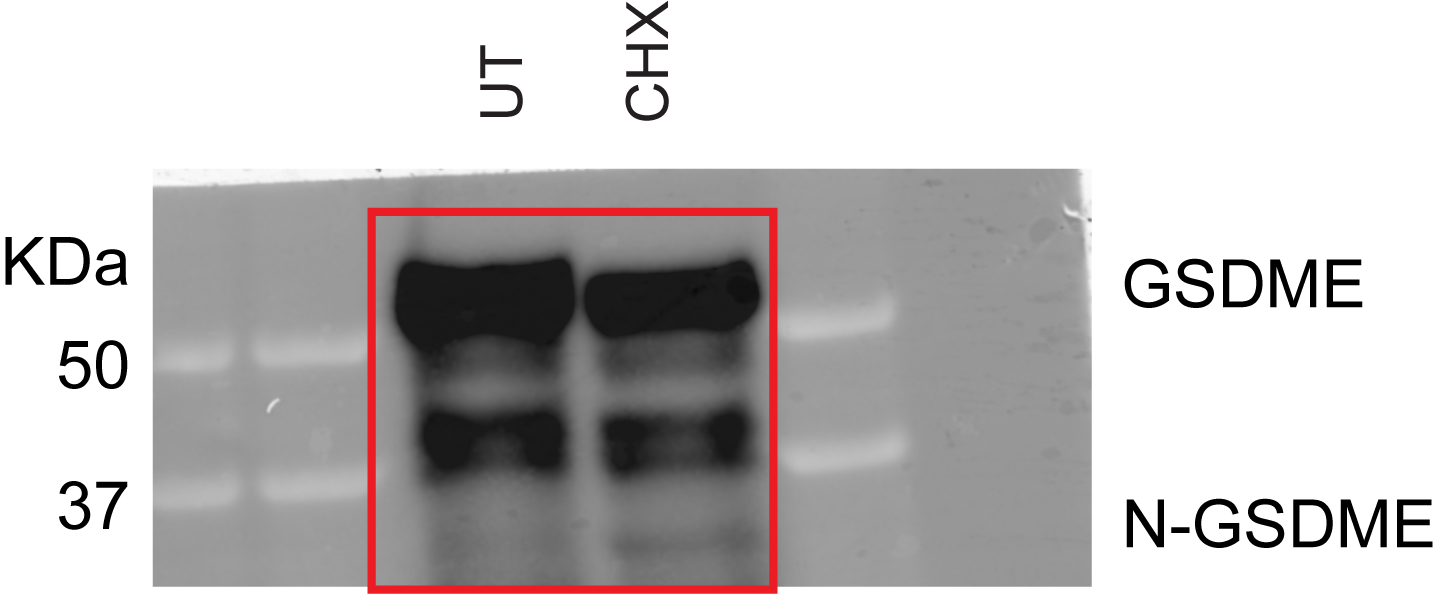

Supplement: Supplementary file 10 — Source Data for Figure 5 [file EMBJ-42-e113118-s004.zip › Source data Figure 5/5F/Western Blot GSDME.tif]

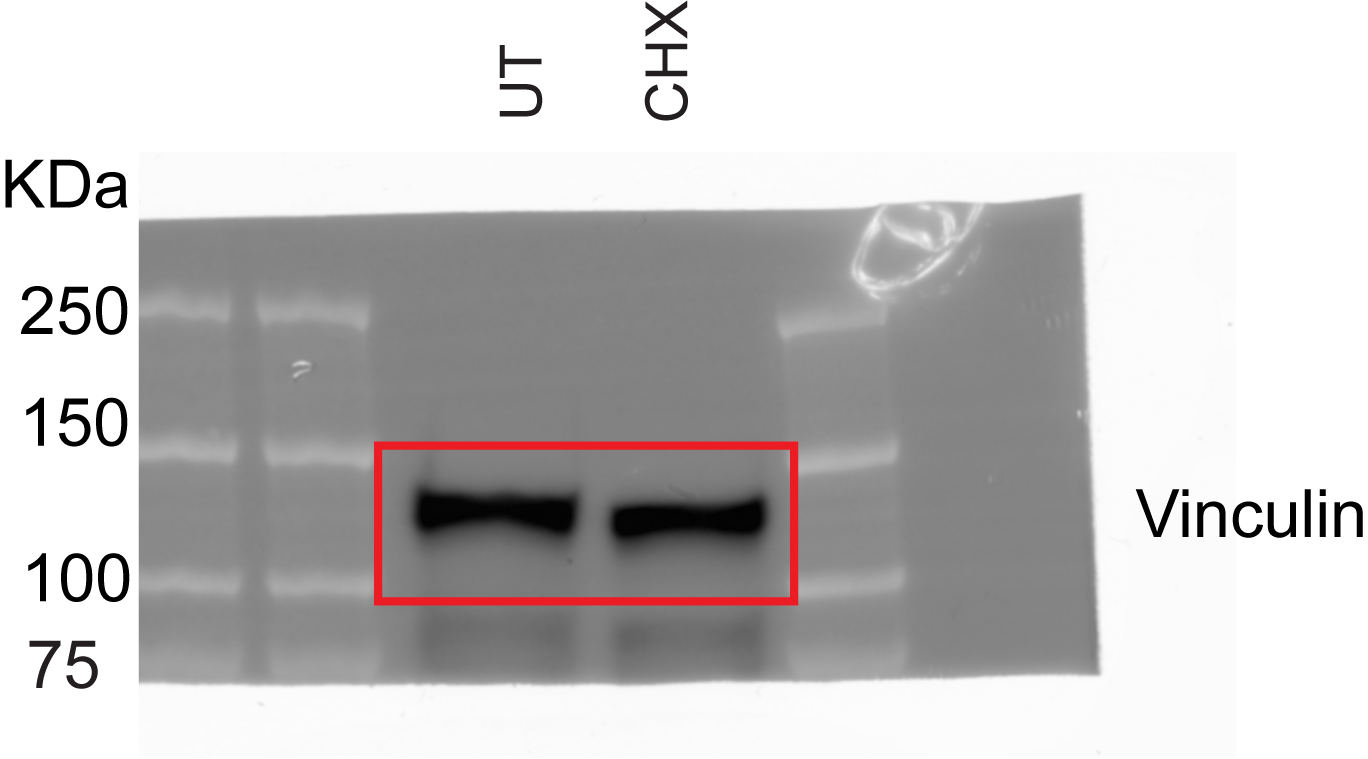

Supplement: Supplementary file 10 — Source Data for Figure 5 [file EMBJ-42-e113118-s004.zip › Source data Figure 5/5F/Western Blot Vinculin.tif]

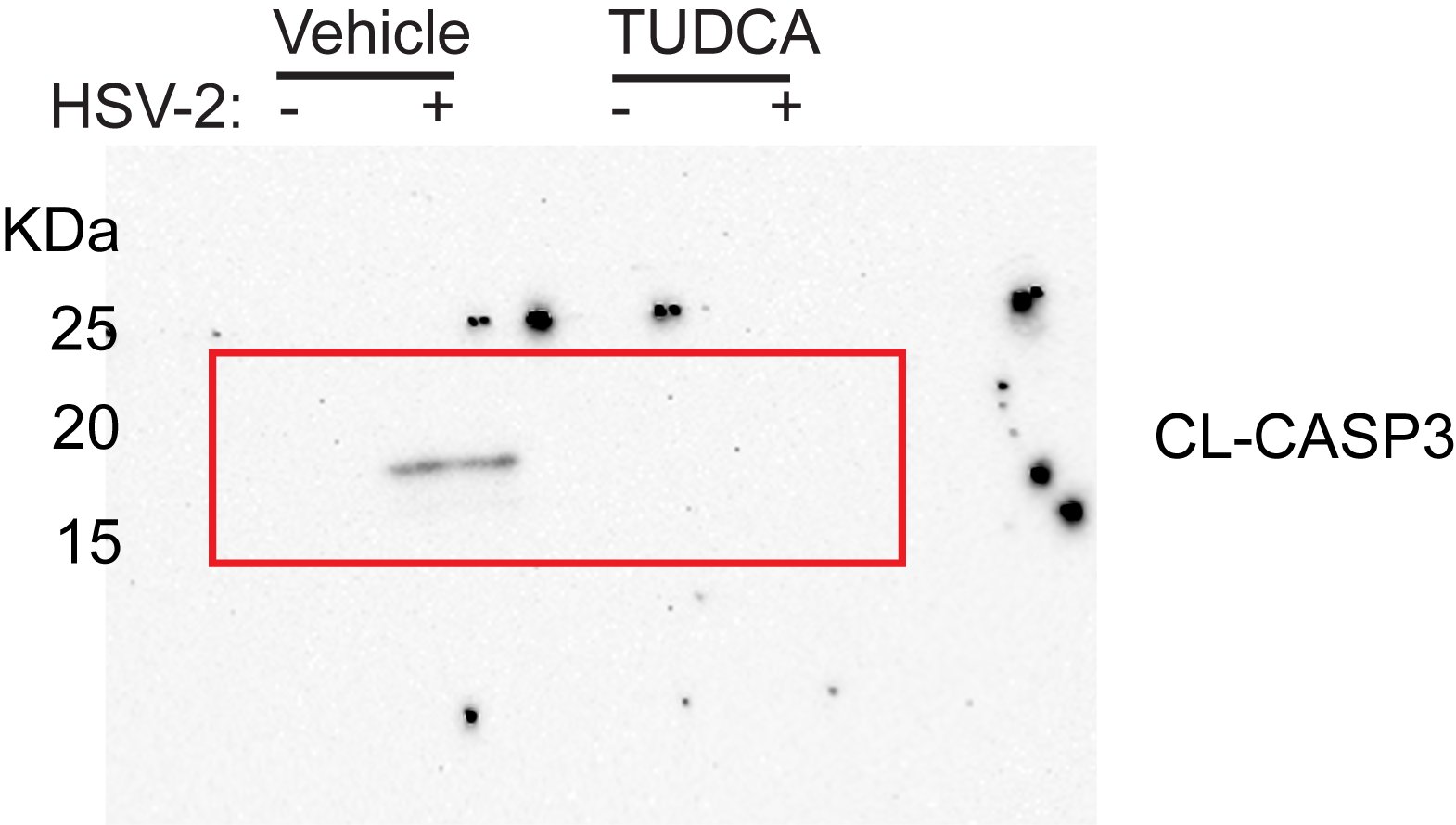

Supplement: Supplementary file 10 — Source Data for Figure 5 [file EMBJ-42-e113118-s004.zip › Source data Figure 5/5G/Western Blot CL-CASP3.tif]

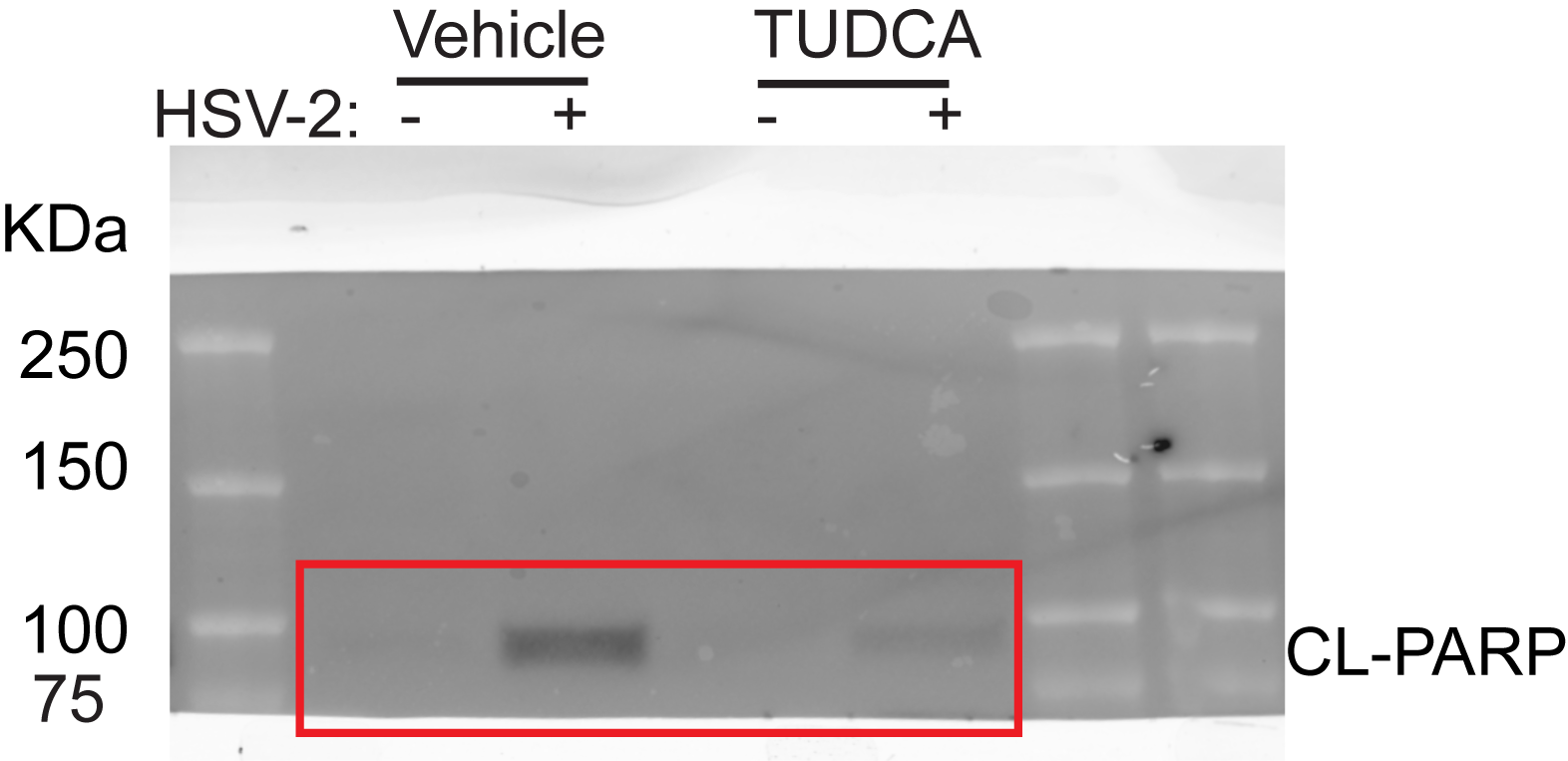

Supplement: Supplementary file 10 — Source Data for Figure 5 [file EMBJ-42-e113118-s004.zip › Source data Figure 5/5G/Western Blot CL-PARP.tif]

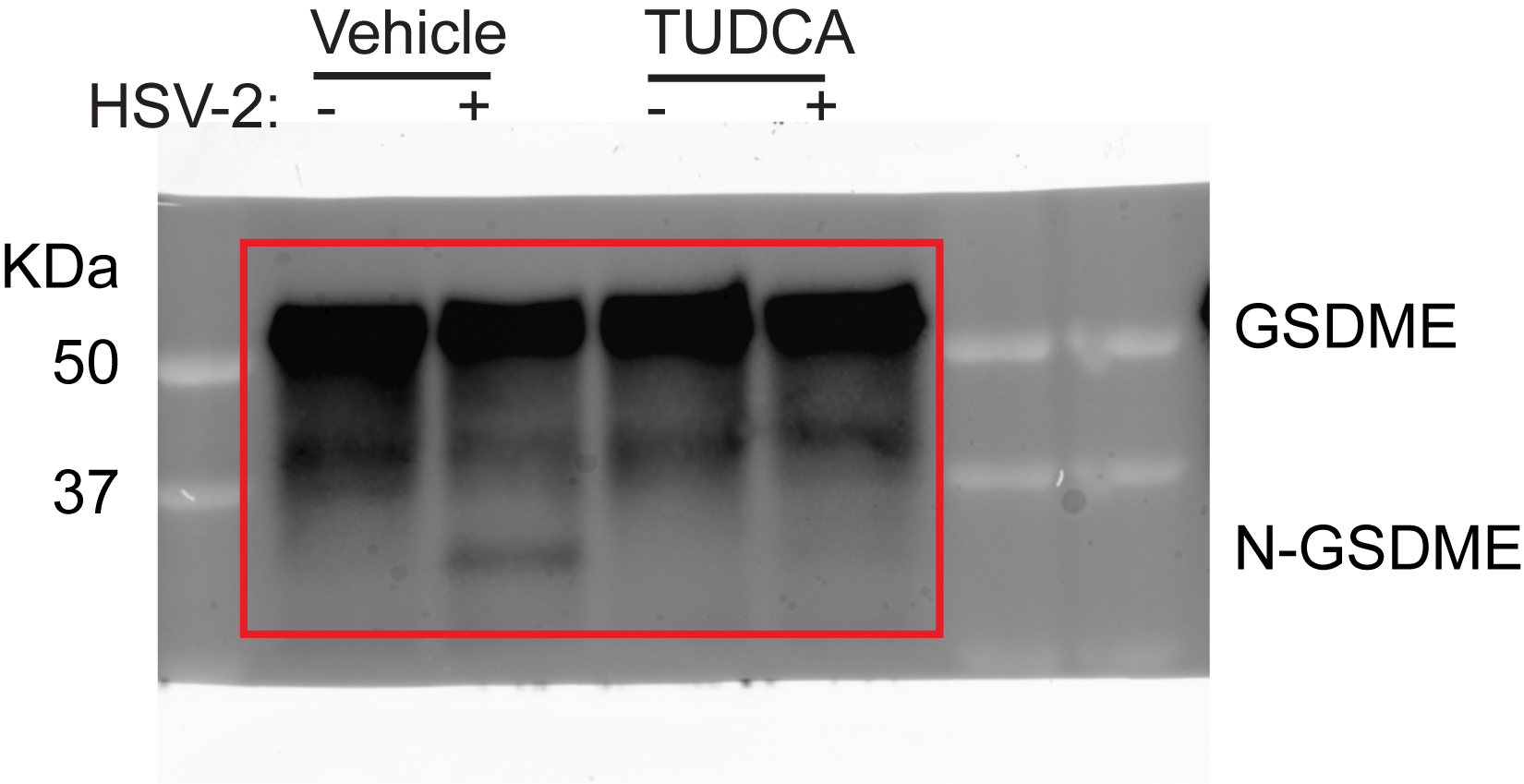

Supplement: Supplementary file 10 — Source Data for Figure 5 [file EMBJ-42-e113118-s004.zip › Source data Figure 5/5G/Western Blot GSDME.tif]

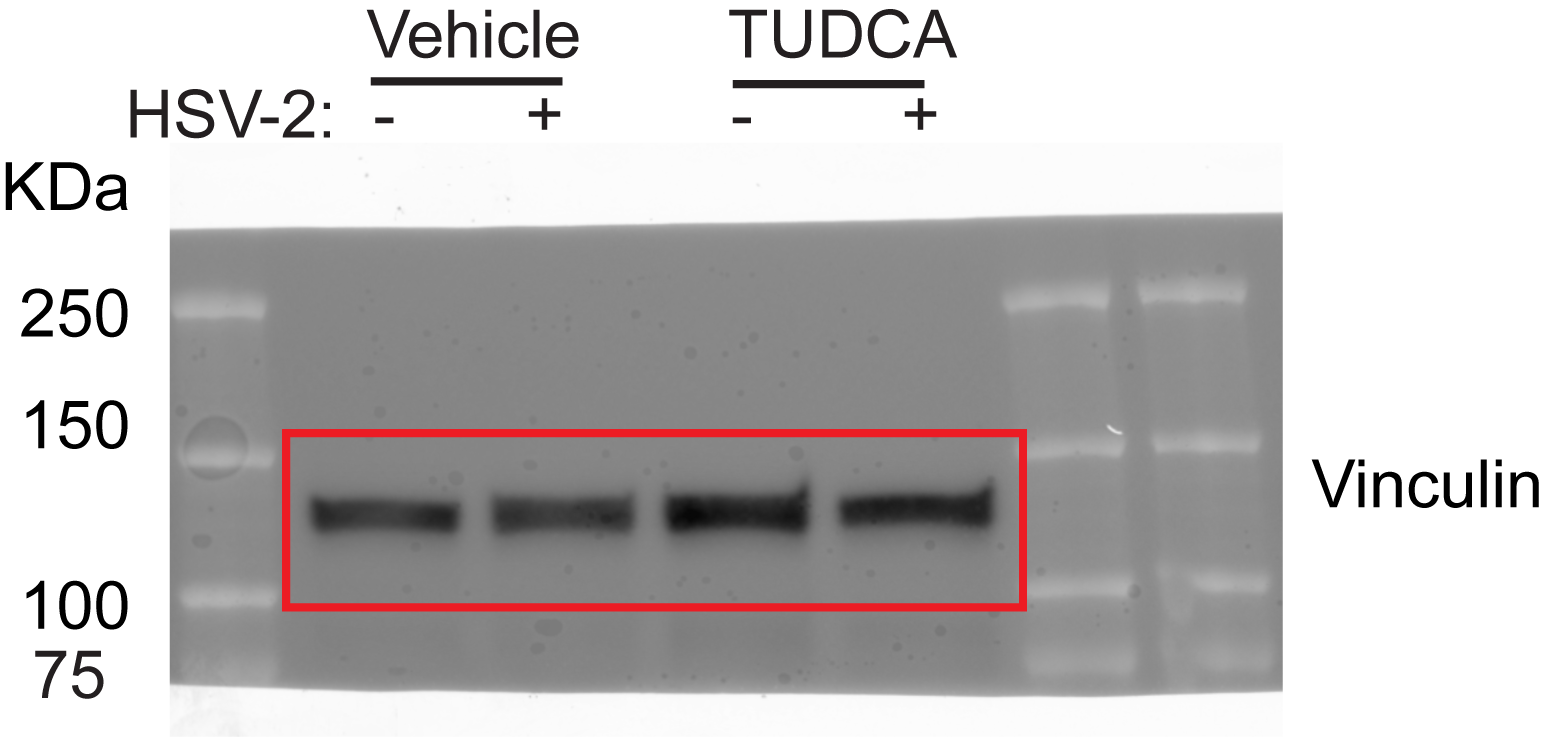

Supplement: Supplementary file 10 — Source Data for Figure 5 [file EMBJ-42-e113118-s004.zip › Source data Figure 5/5G/Western Blot Vinculin.tif]

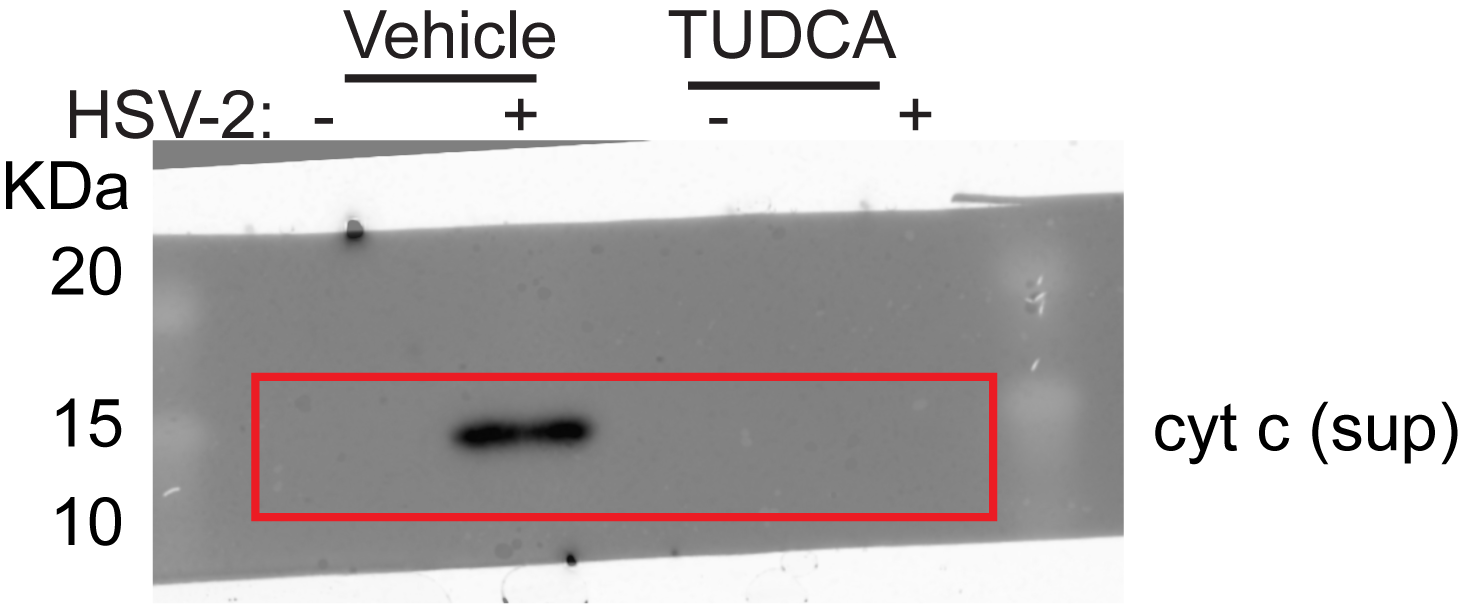

Supplement: Supplementary file 10 — Source Data for Figure 5 [file EMBJ-42-e113118-s004.zip › Source data Figure 5/5I/Western Blot Cyt c (sup).tif]

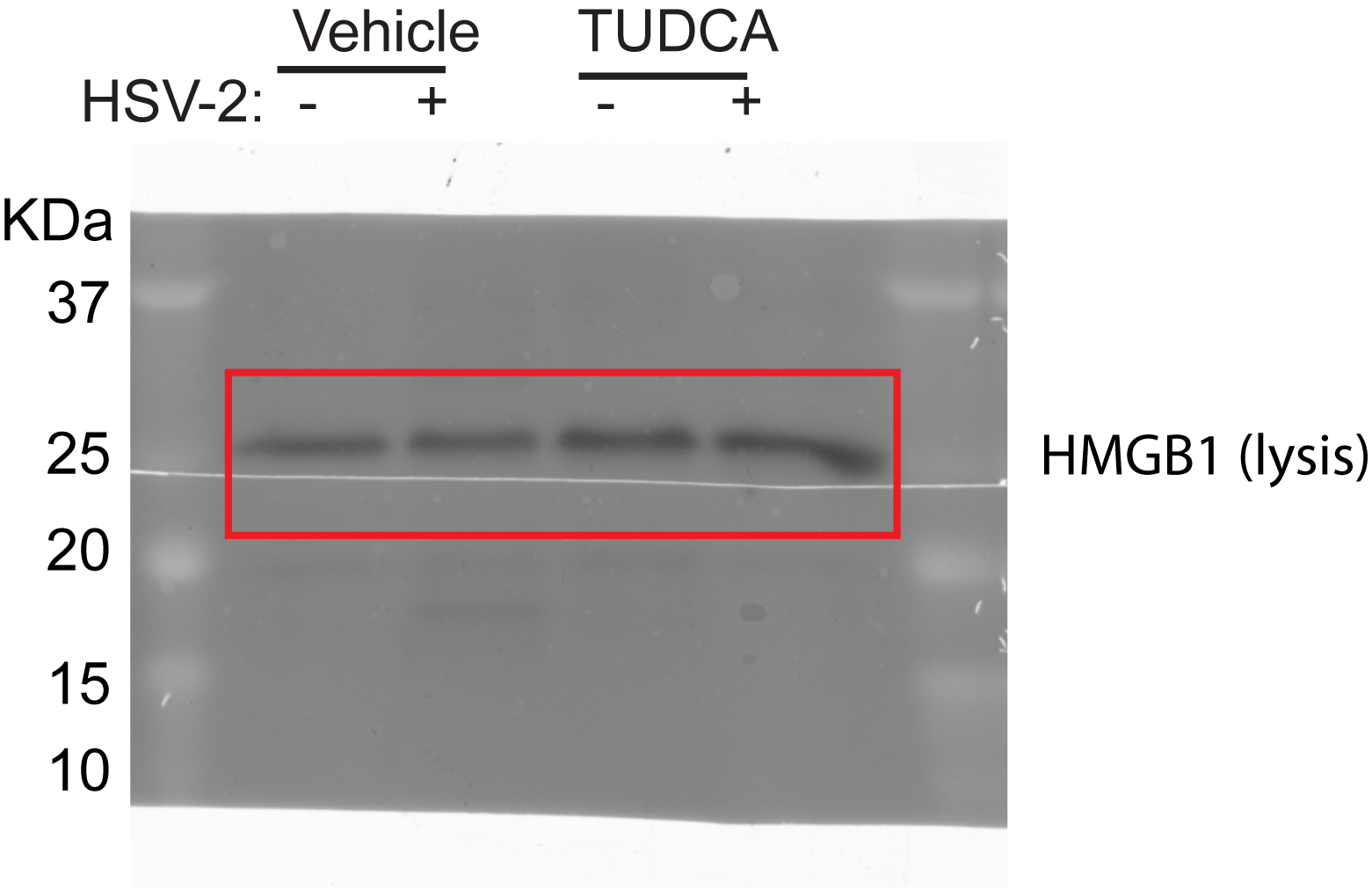

Supplement: Supplementary file 10 — Source Data for Figure 5 [file EMBJ-42-e113118-s004.zip › Source data Figure 5/5I/Western Blot HMGB1 (lysis).tif]

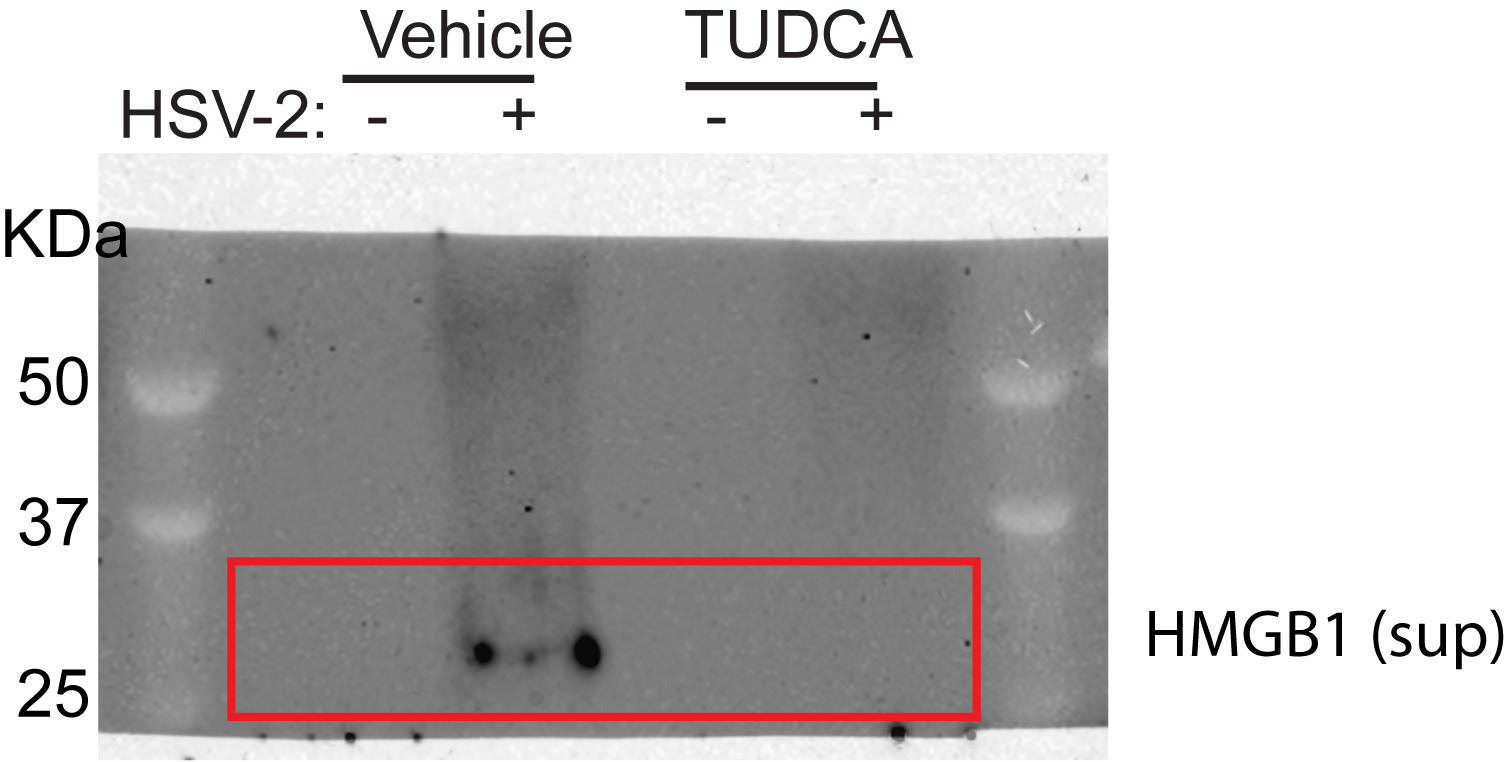

Supplement: Supplementary file 10 — Source Data for Figure 5 [file EMBJ-42-e113118-s004.zip › Source data Figure 5/5I/Western Blot HMGB1 (sup).tif]

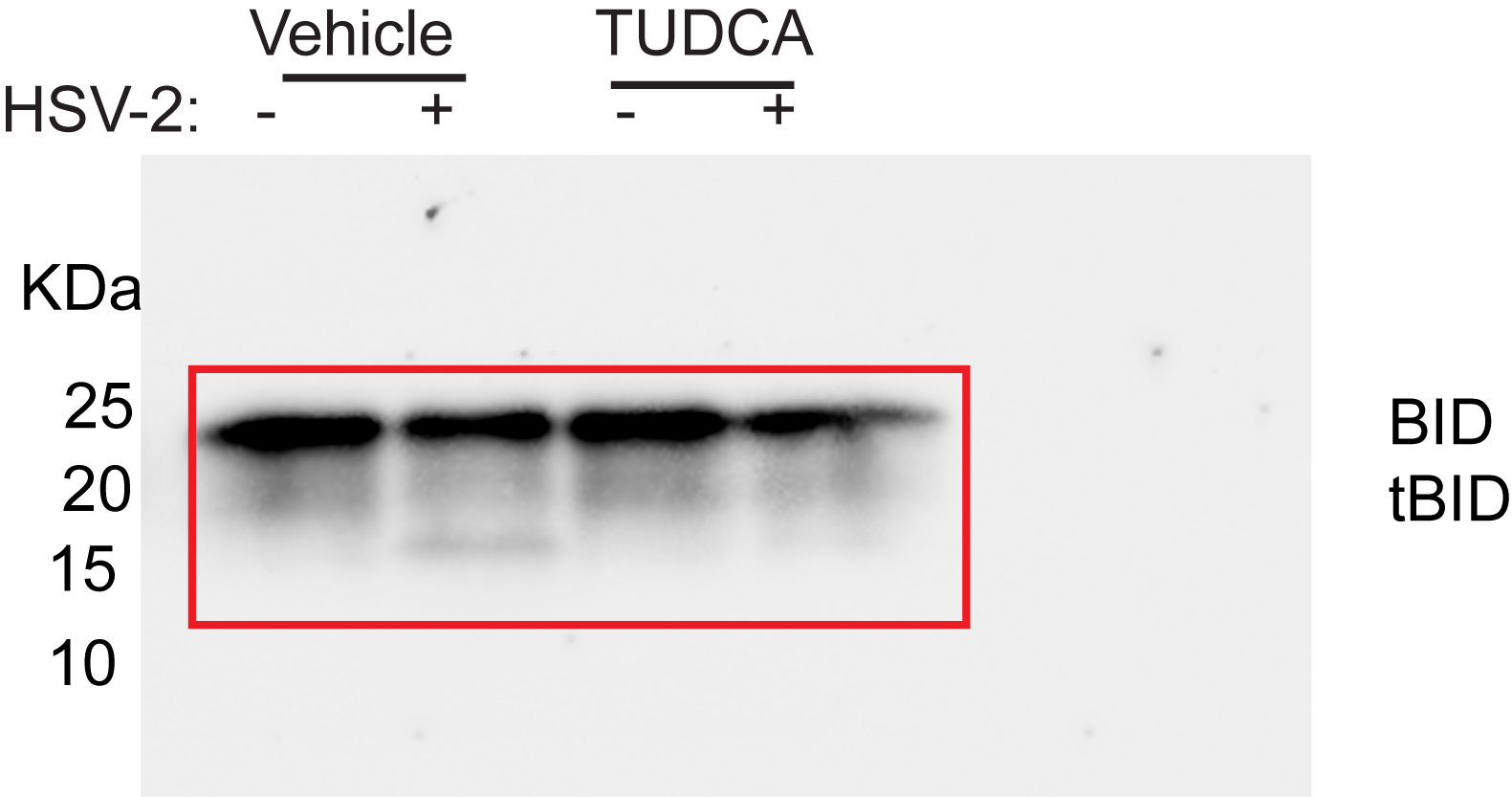

Supplement: Supplementary file 10 — Source Data for Figure 5 [file EMBJ-42-e113118-s004.zip › Source data Figure 5/5K/Western Blot BID.tif]

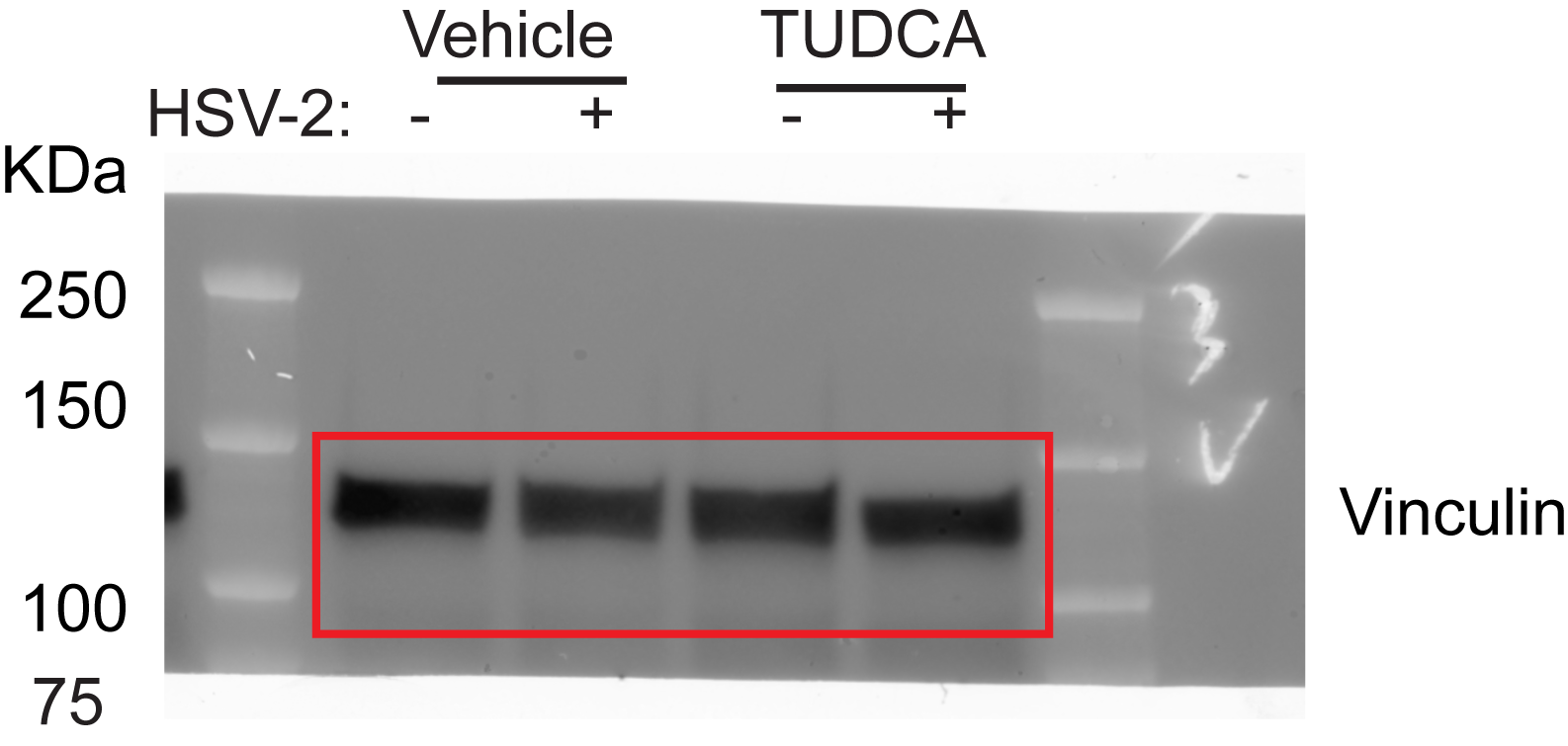

Supplement: Supplementary file 10 — Source Data for Figure 5 [file EMBJ-42-e113118-s004.zip › Source data Figure 5/5K/Western Blot Vinculin.tif]

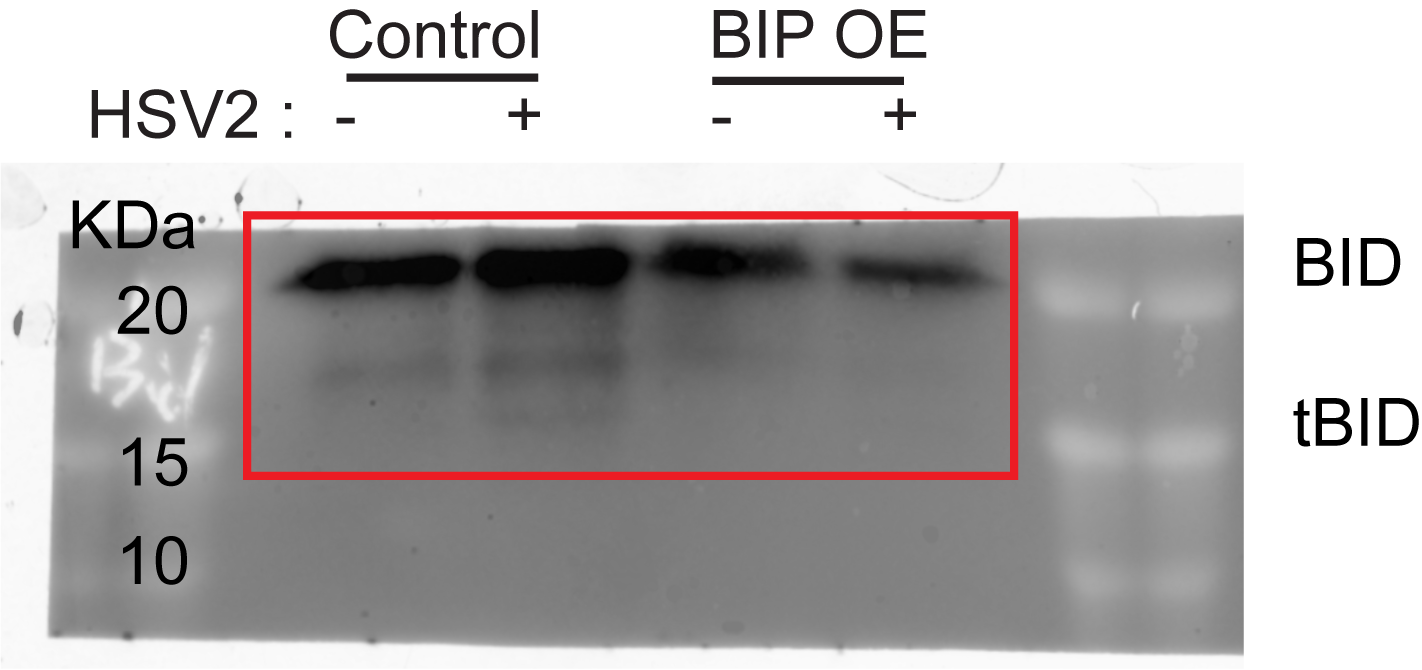

Supplement: Supplementary file 10 — Source Data for Figure 5 [file EMBJ-42-e113118-s004.zip › Source data Figure 5/5L/Western Blot BID.tif]

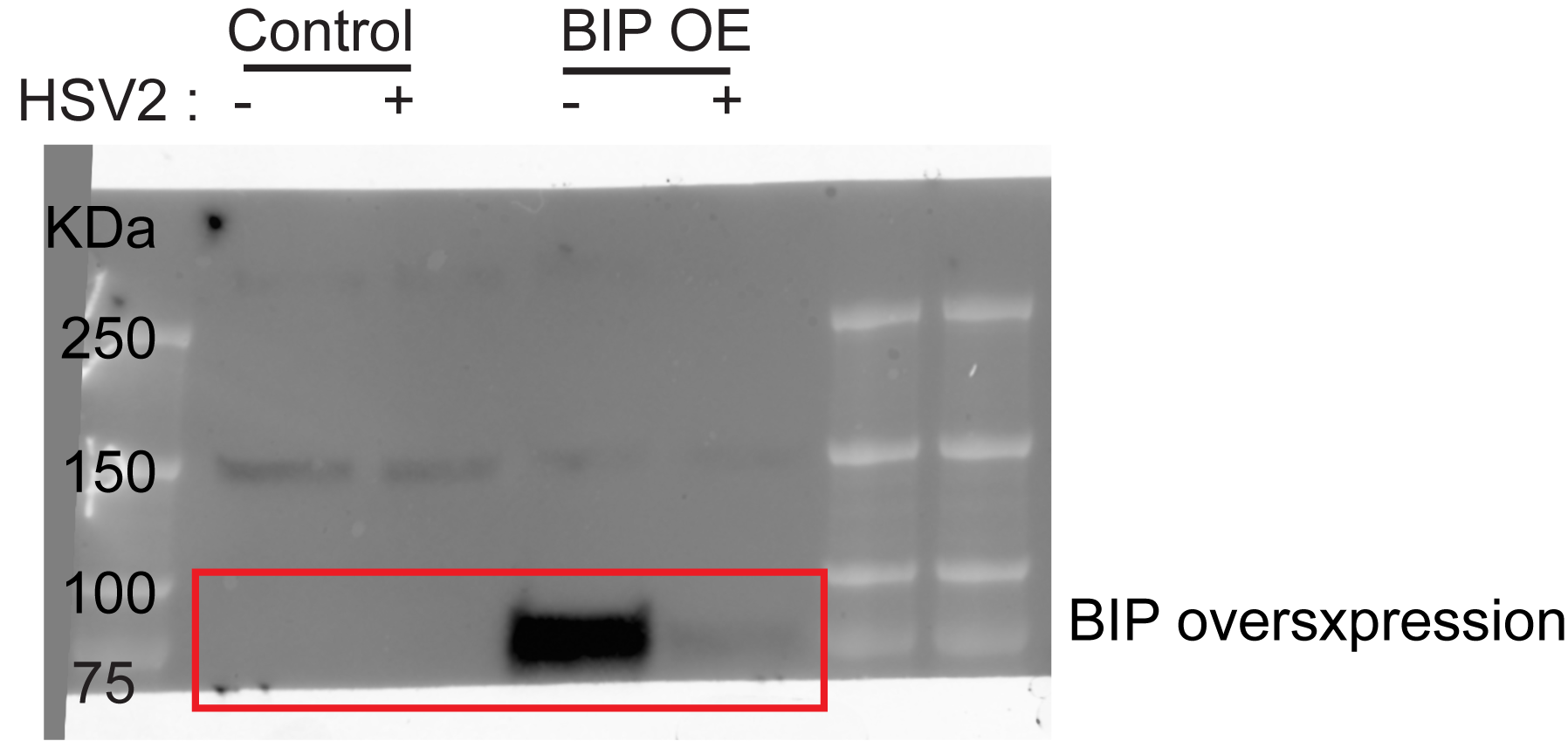

Supplement: Supplementary file 10 — Source Data for Figure 5 [file EMBJ-42-e113118-s004.zip › Source data Figure 5/5L/Western Blot BIP overexpression.tif]

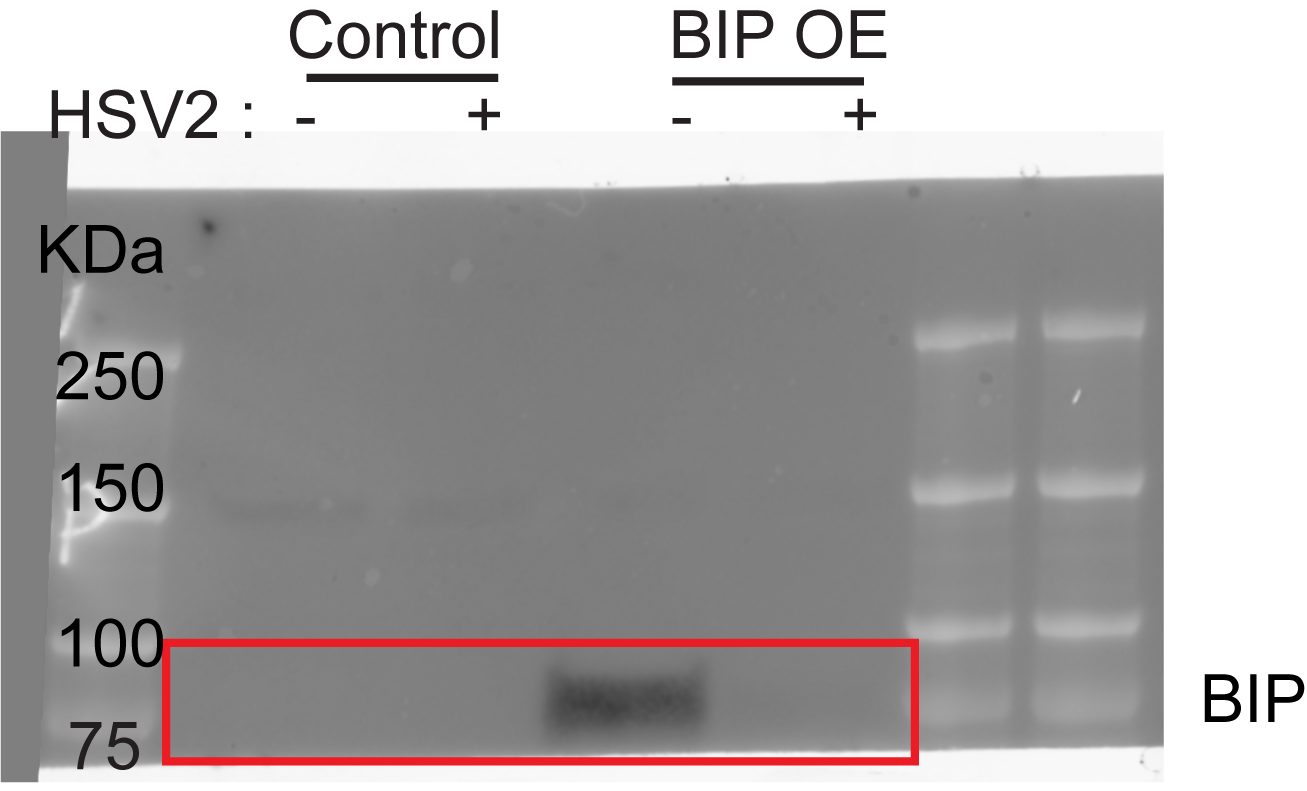

Supplement: Supplementary file 10 — Source Data for Figure 5 [file EMBJ-42-e113118-s004.zip › Source data Figure 5/5L/Western Blot BIP.tif]

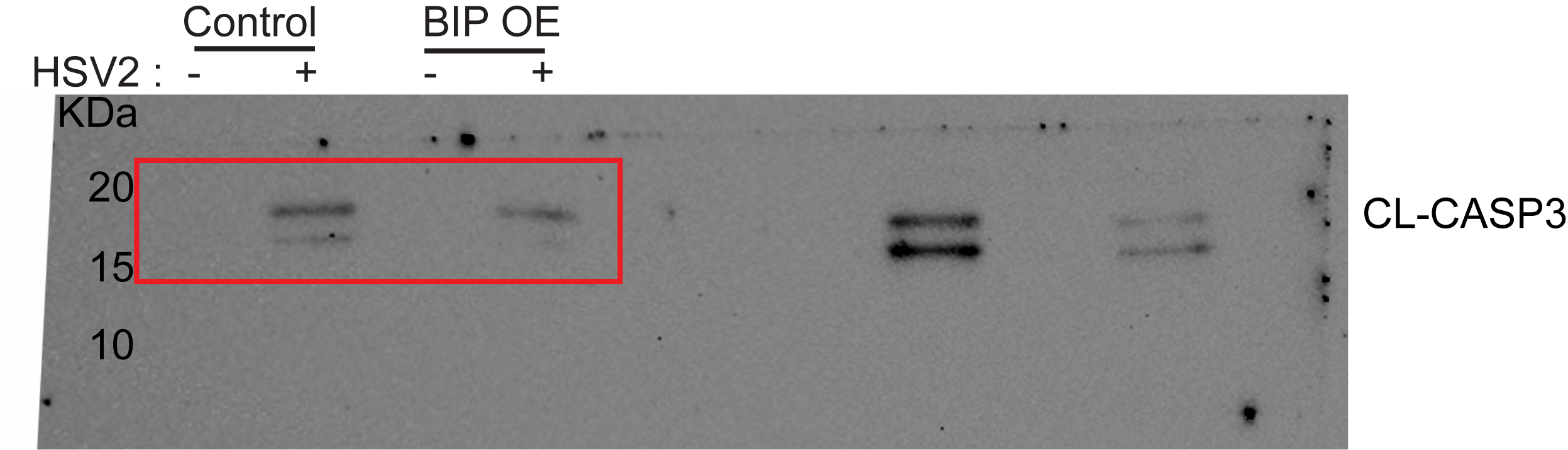

Supplement: Supplementary file 10 — Source Data for Figure 5 [file EMBJ-42-e113118-s004.zip › Source data Figure 5/5L/Western Blot CL-CASP3.tif]

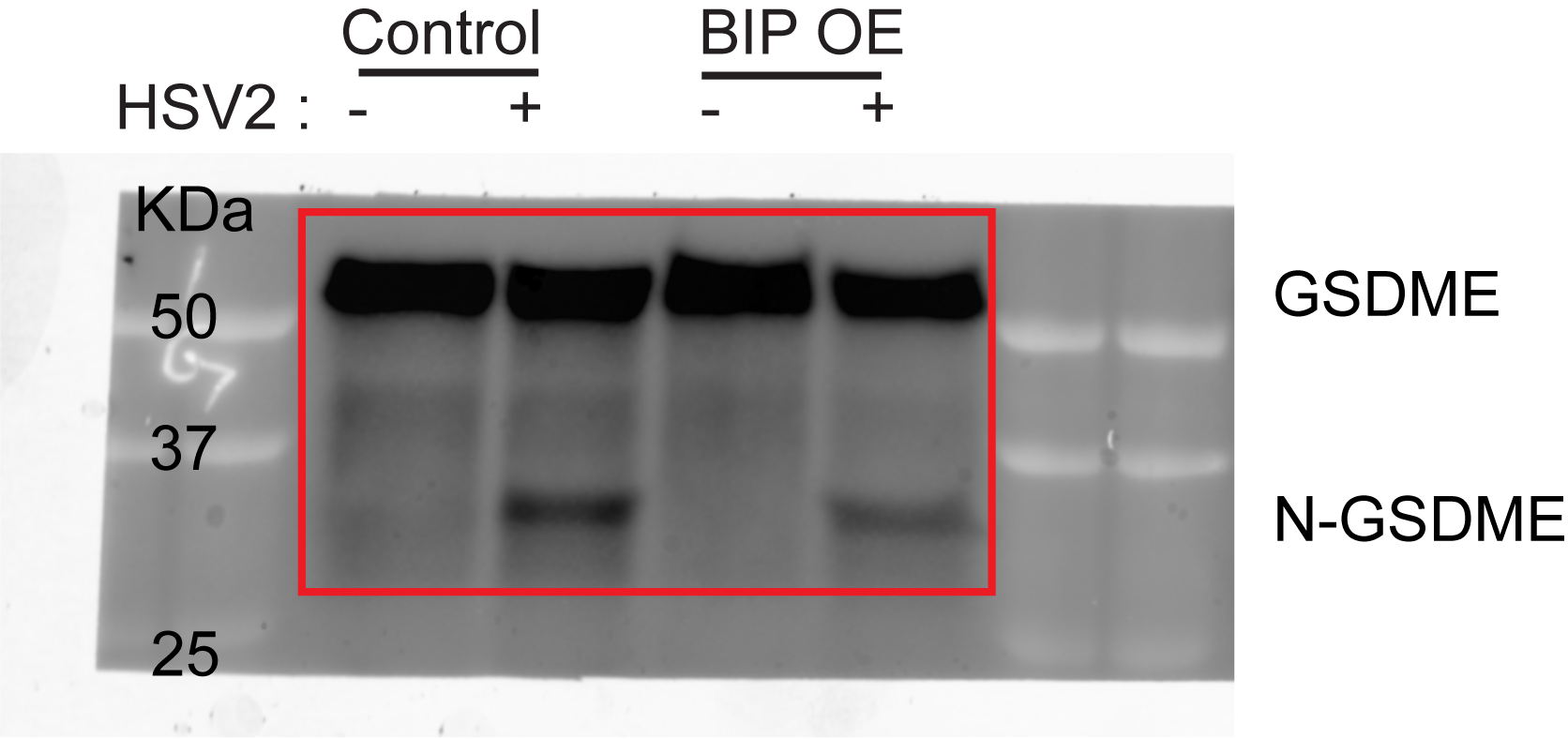

Supplement: Supplementary file 10 — Source Data for Figure 5 [file EMBJ-42-e113118-s004.zip › Source data Figure 5/5L/Western Blot GSDME.tif]

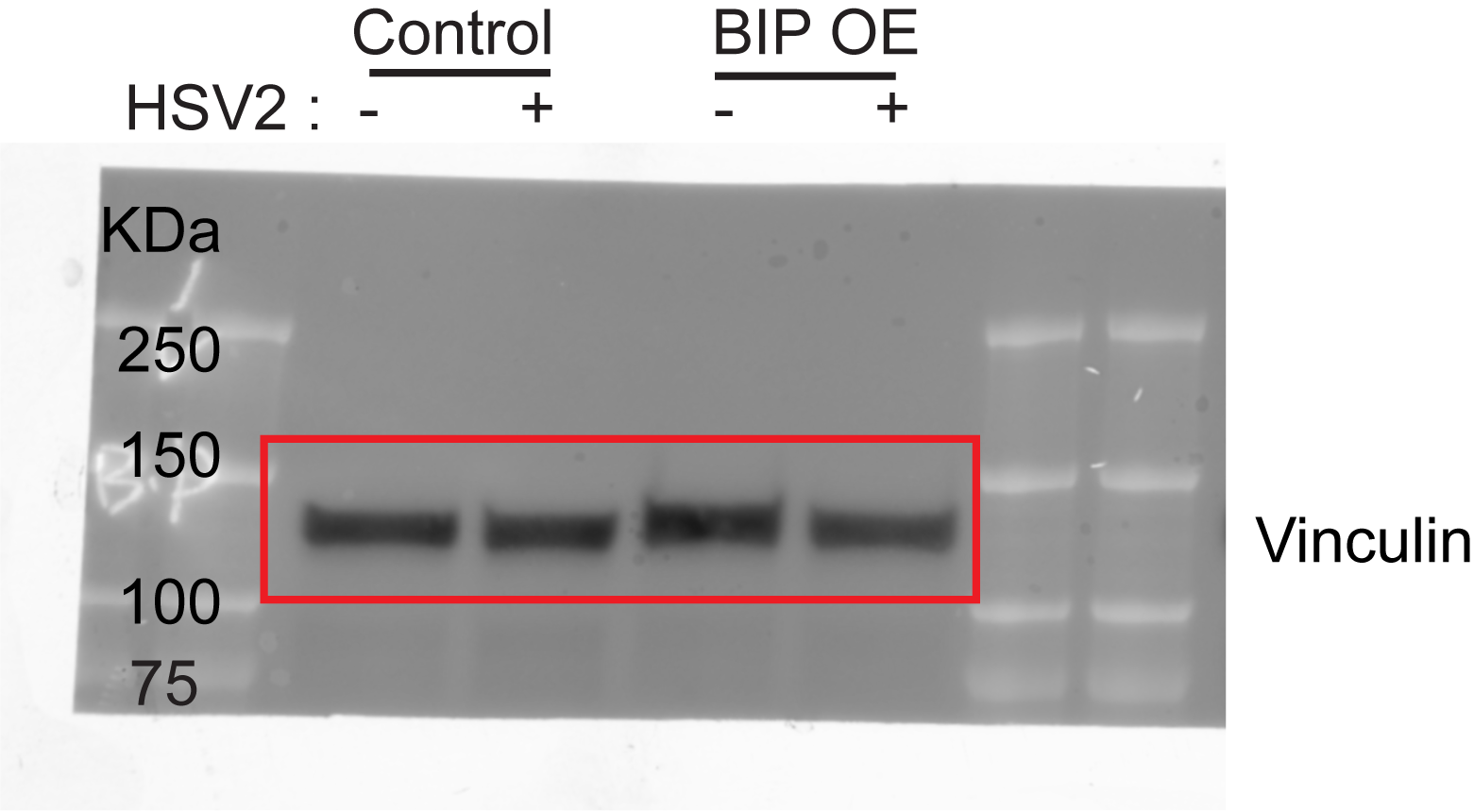

Supplement: Supplementary file 10 — Source Data for Figure 5 [file EMBJ-42-e113118-s004.zip › Source data Figure 5/5L/Western Blot Vinculin.tif]

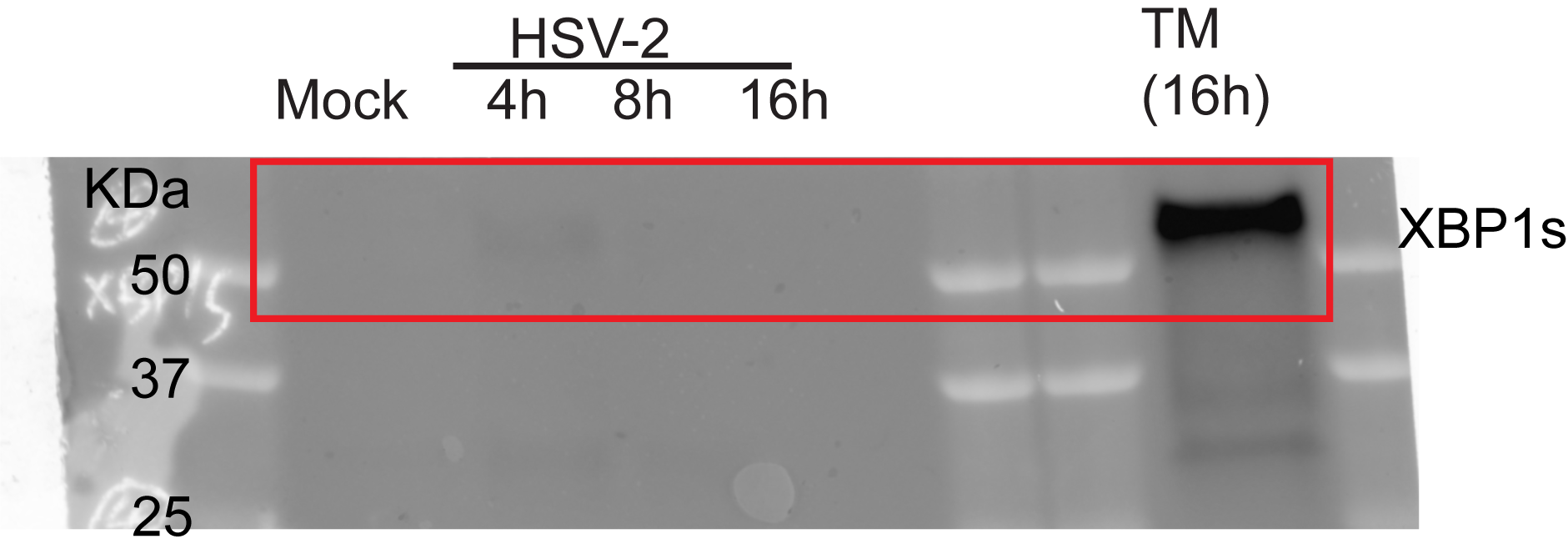

Supplement: Supplementary file 11 — Source Data for Figure 6 [file EMBJ-42-e113118-s002.zip › Source data Figure 6/6C/Western Blot XBP1s.tif]

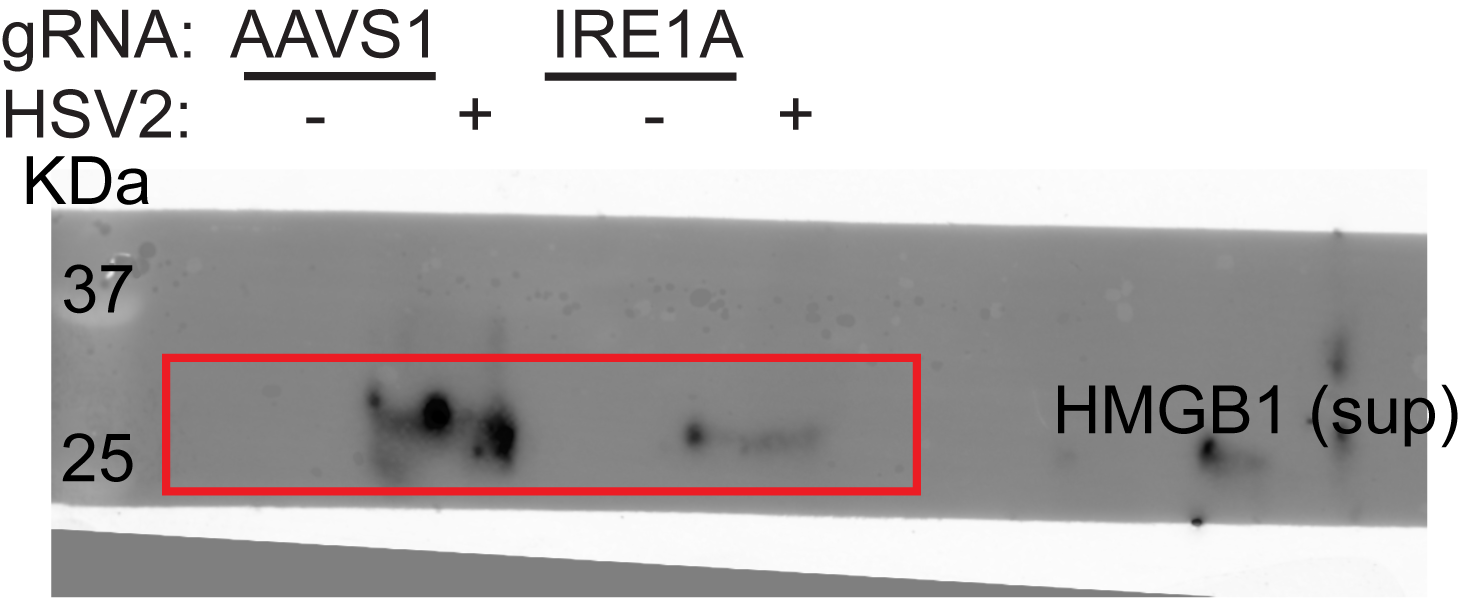

Supplement: Supplementary file 11 — Source Data for Figure 6 [file EMBJ-42-e113118-s002.zip › Source data Figure 6/6E/Weatern Blot HMGB1 (sup).tif]

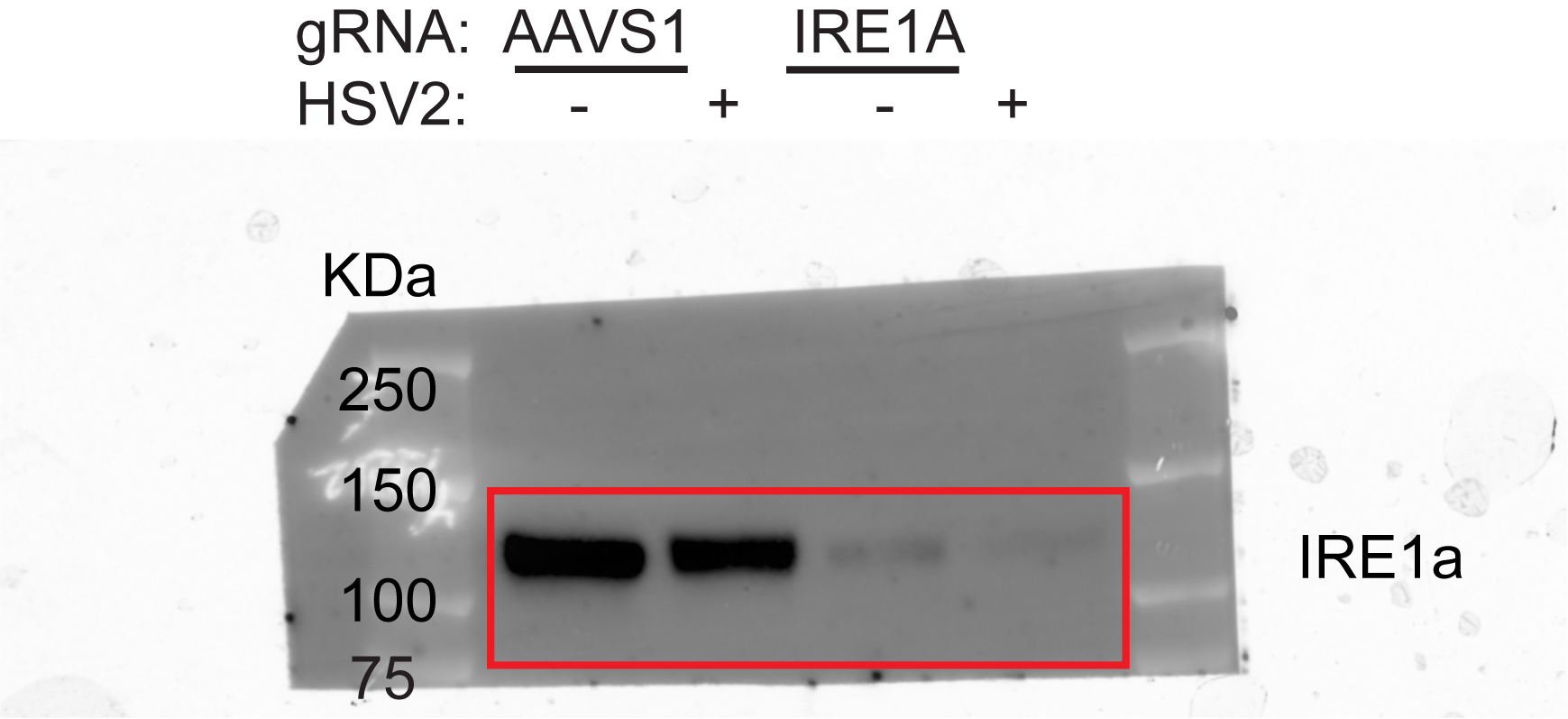

Supplement: Supplementary file 11 — Source Data for Figure 6 [file EMBJ-42-e113118-s002.zip › Source data Figure 6/6E/Weatern Blot IRE1a.tif]

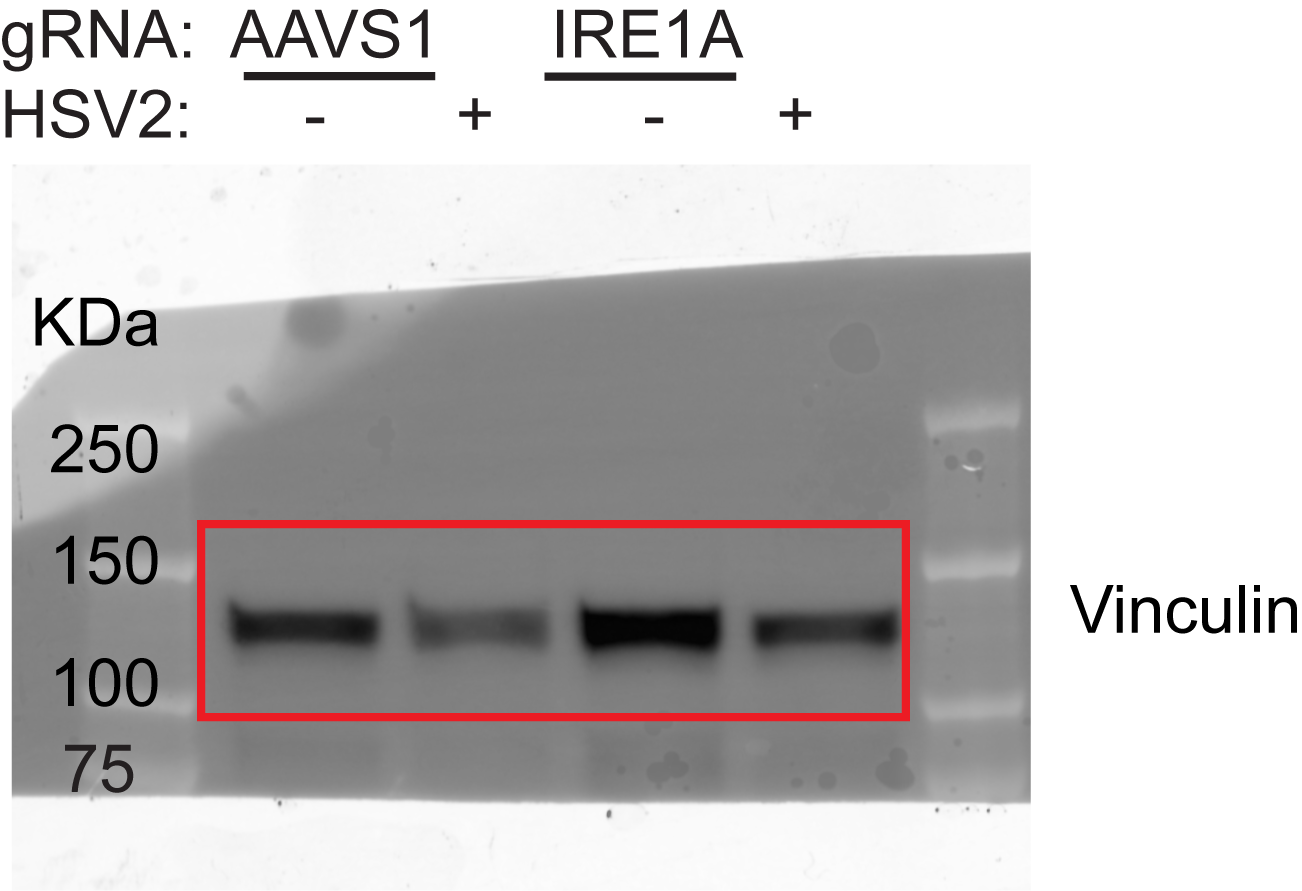

Supplement: Supplementary file 11 — Source Data for Figure 6 [file EMBJ-42-e113118-s002.zip › Source data Figure 6/6E/Weatern Blot Vinculin.tif]

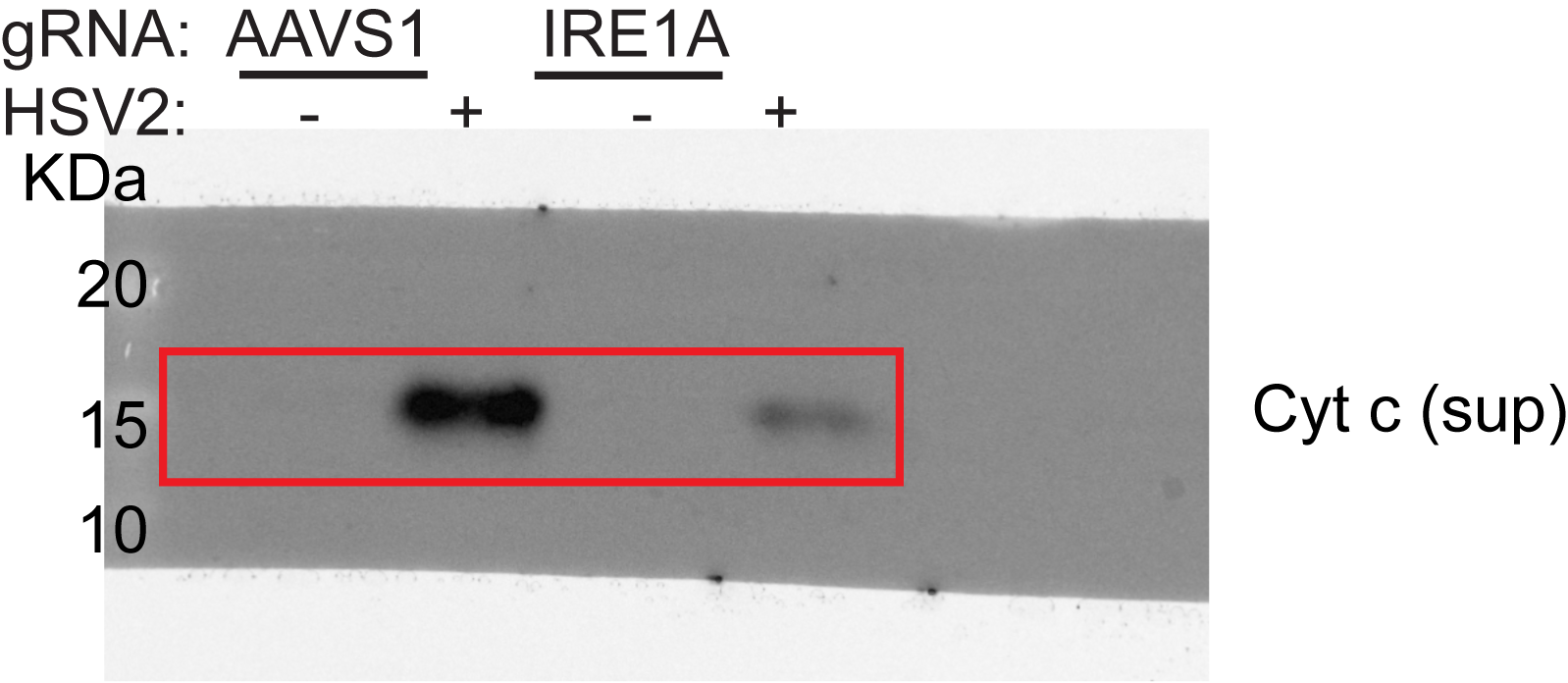

Supplement: Supplementary file 11 — Source Data for Figure 6 [file EMBJ-42-e113118-s002.zip › Source data Figure 6/6E/Western Blot Cyt c (sup).tif]

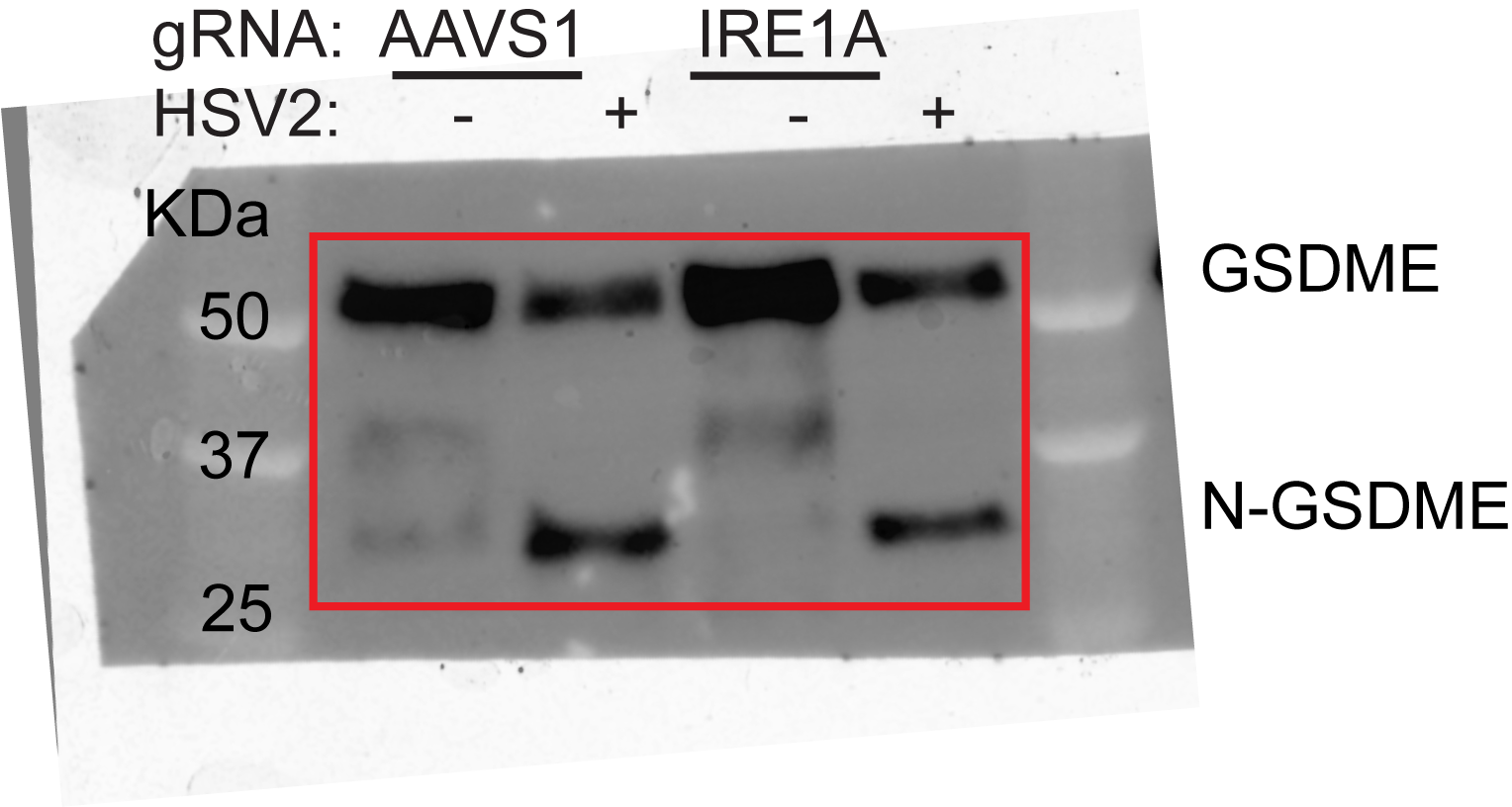

Supplement: Supplementary file 11 — Source Data for Figure 6 [file EMBJ-42-e113118-s002.zip › Source data Figure 6/6E/Western Blot GSDME.tif]

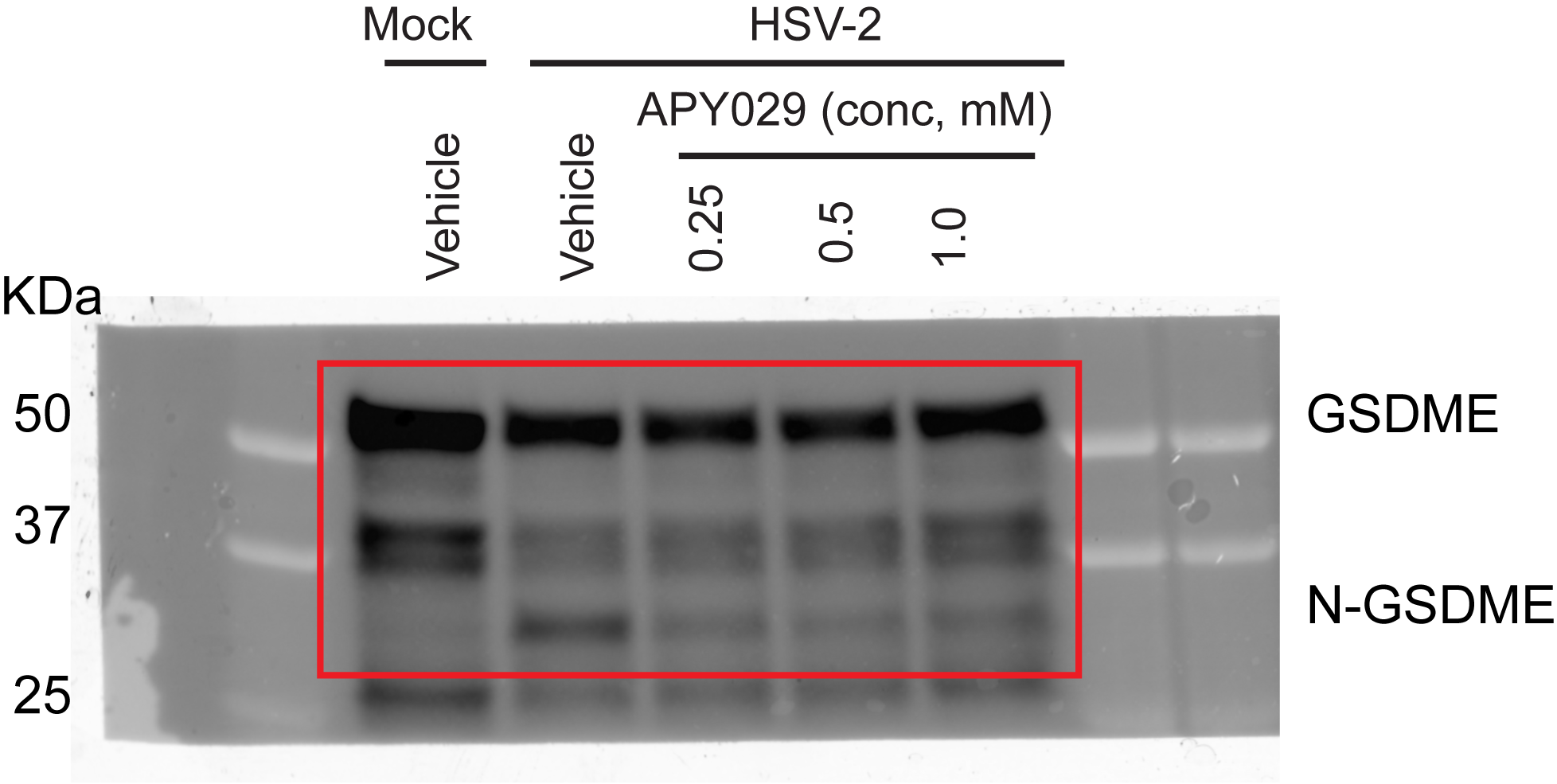

Supplement: Supplementary file 11 — Source Data for Figure 6 [file EMBJ-42-e113118-s002.zip › Source data Figure 6/6F/Weatern Blot GSDME.tif]

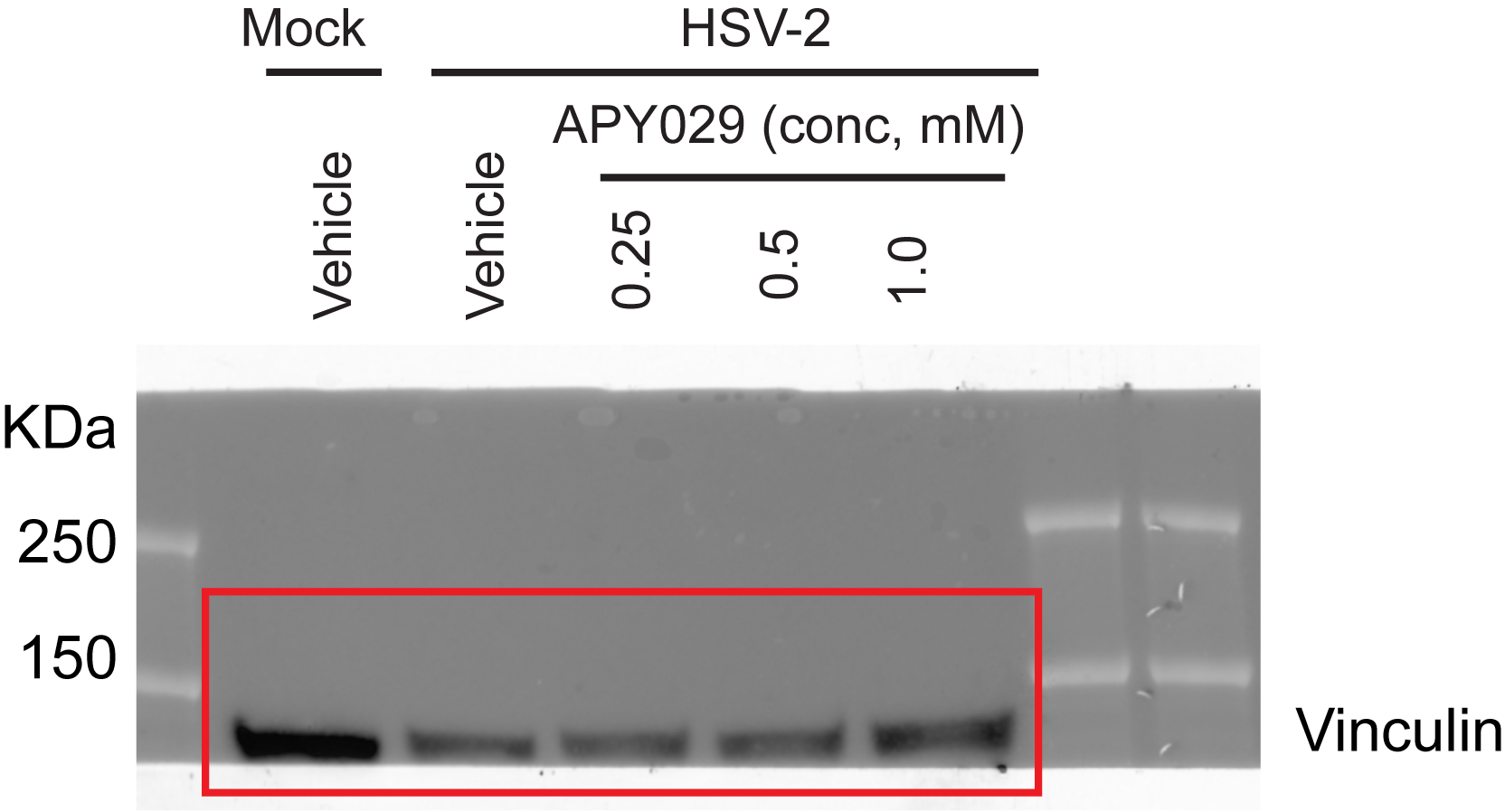

Supplement: Supplementary file 11 — Source Data for Figure 6 [file EMBJ-42-e113118-s002.zip › Source data Figure 6/6F/Weatern Blot Vinculin.tif]

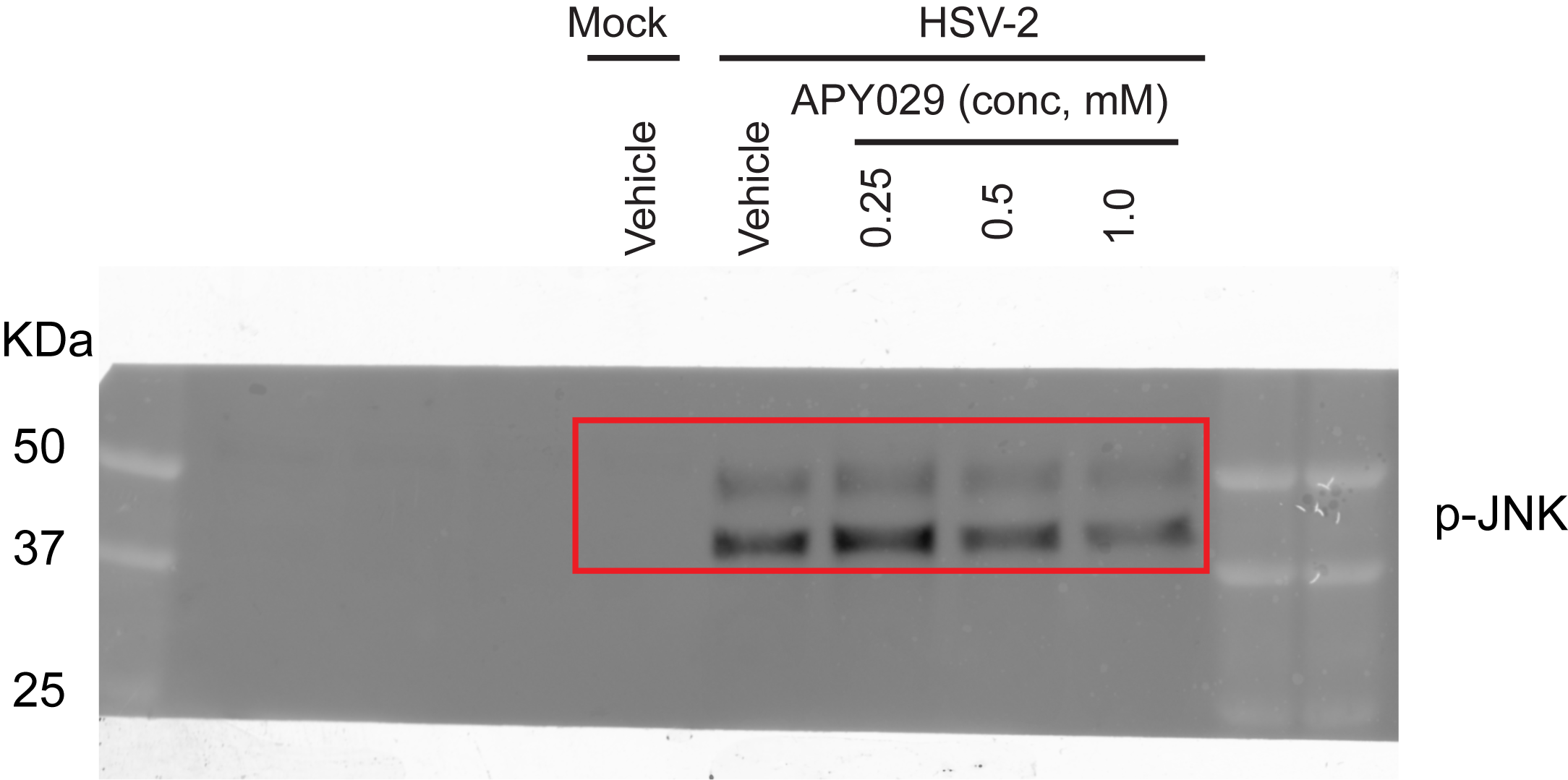

Supplement: Supplementary file 11 — Source Data for Figure 6 [file EMBJ-42-e113118-s002.zip › Source data Figure 6/6G/Weatern Blot p-JNK.tif]

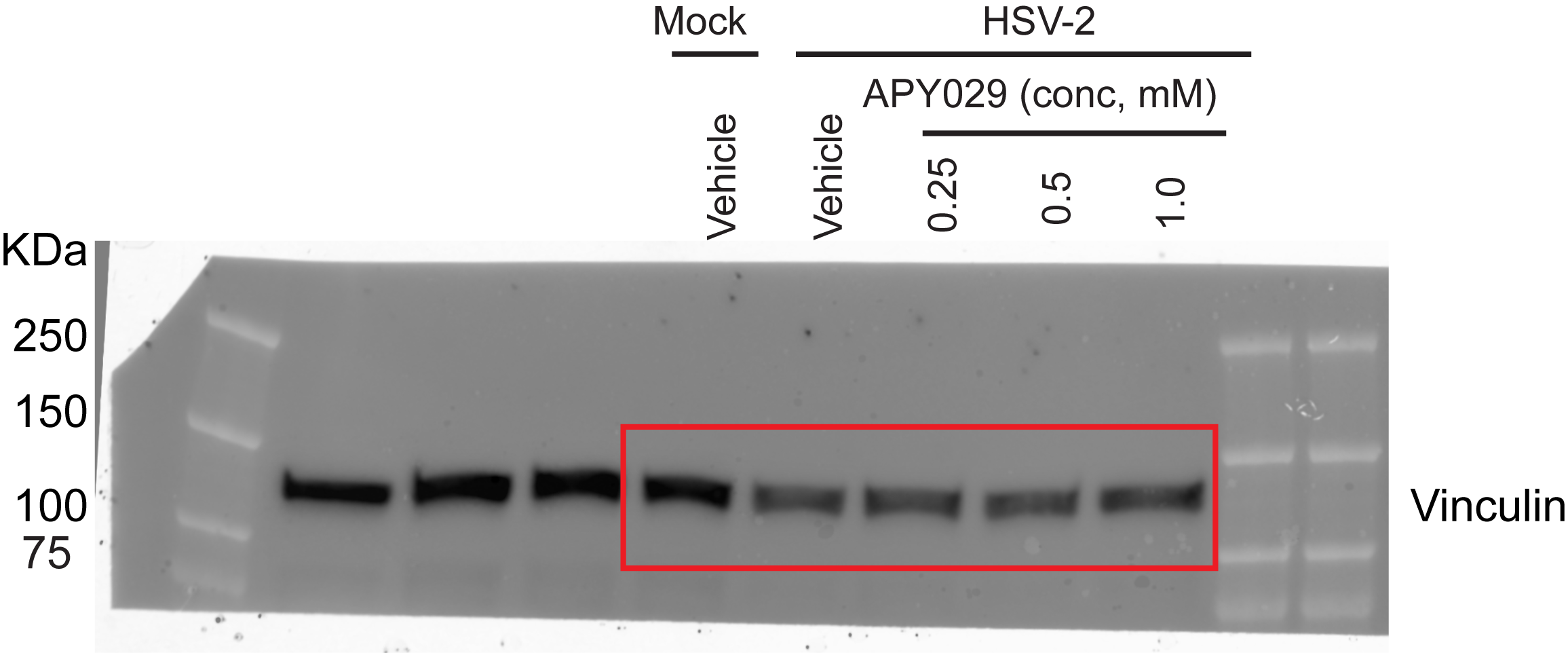

Supplement: Supplementary file 11 — Source Data for Figure 6 [file EMBJ-42-e113118-s002.zip › Source data Figure 6/6G/Weatern Blot Vinculin.tif]

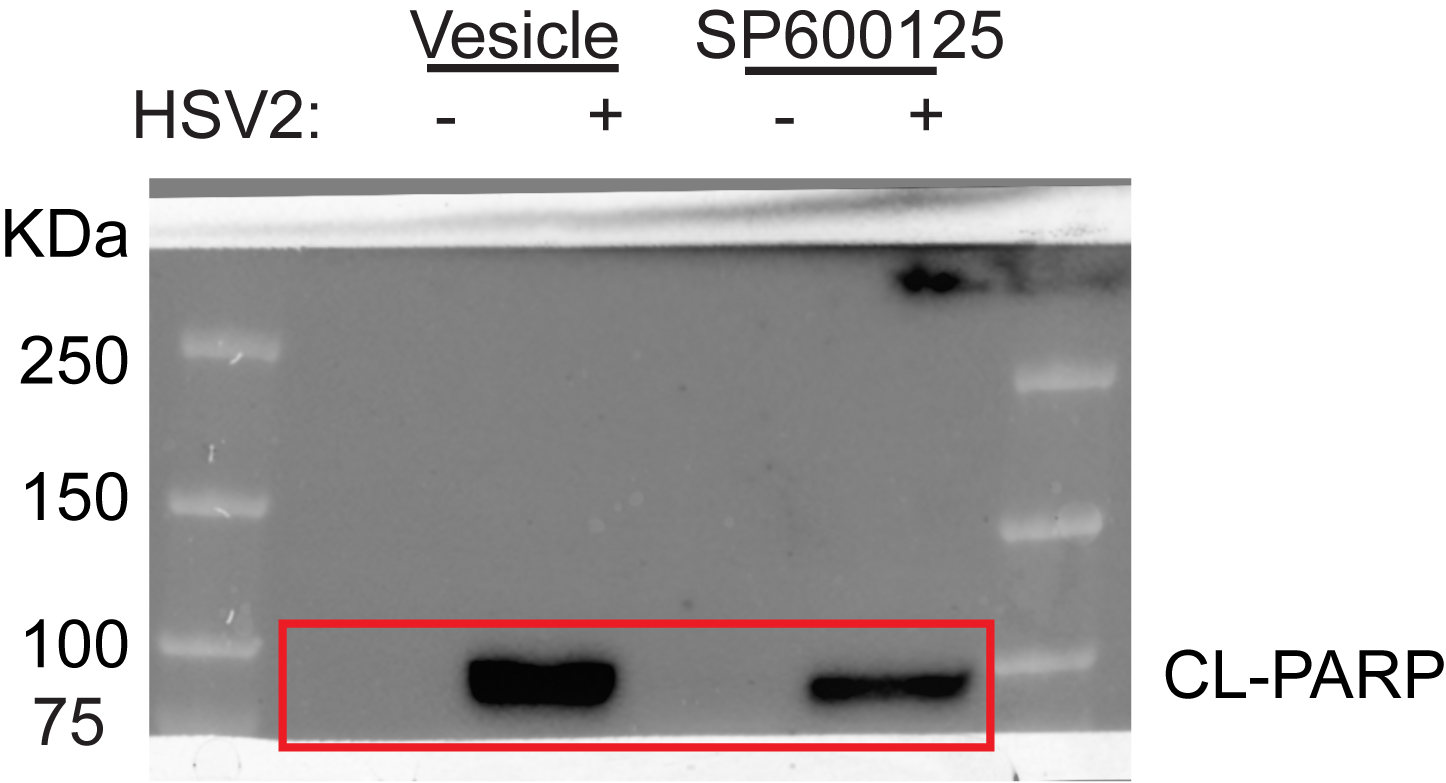

Supplement: Supplementary file 11 — Source Data for Figure 6 [file EMBJ-42-e113118-s002.zip › Source data Figure 6/6H/Weatern Blot CL-PARP.tif]

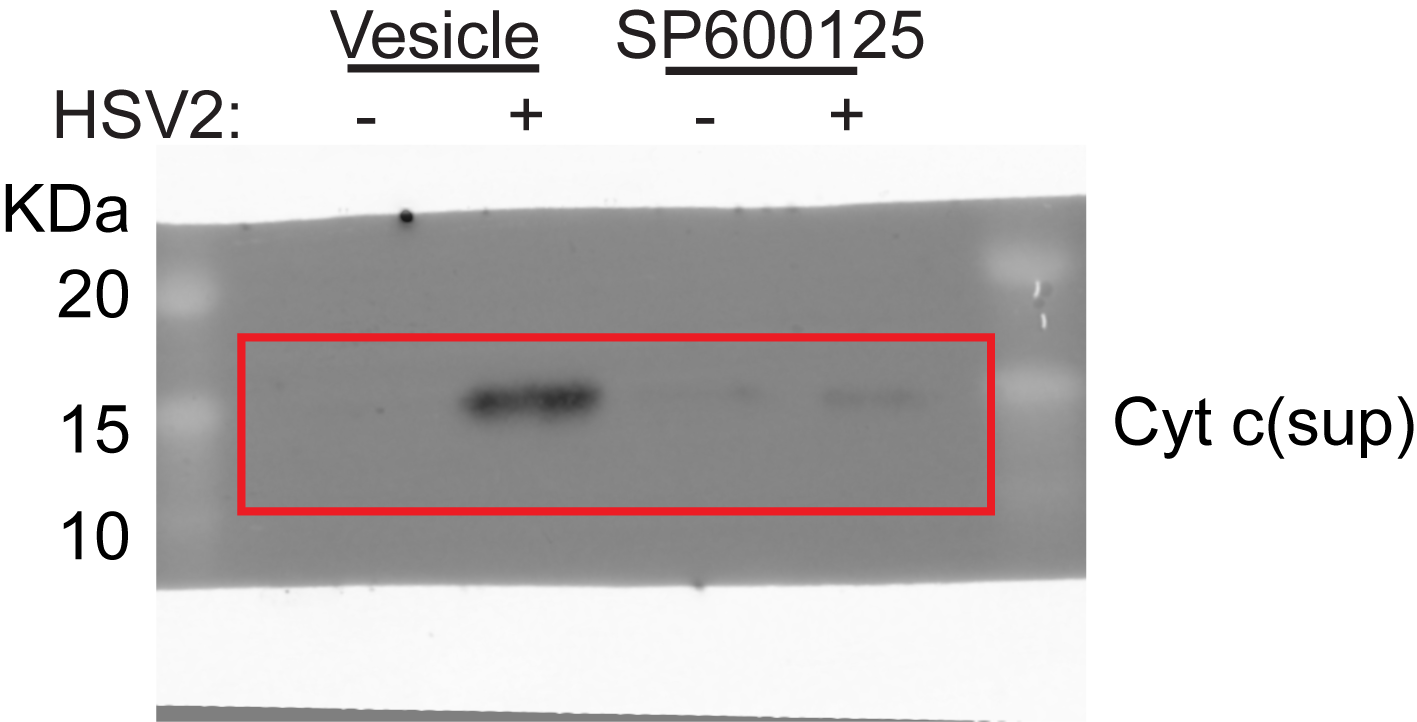

Supplement: Supplementary file 11 — Source Data for Figure 6 [file EMBJ-42-e113118-s002.zip › Source data Figure 6/6H/Weatern Blot Cyt c (sup).tif]

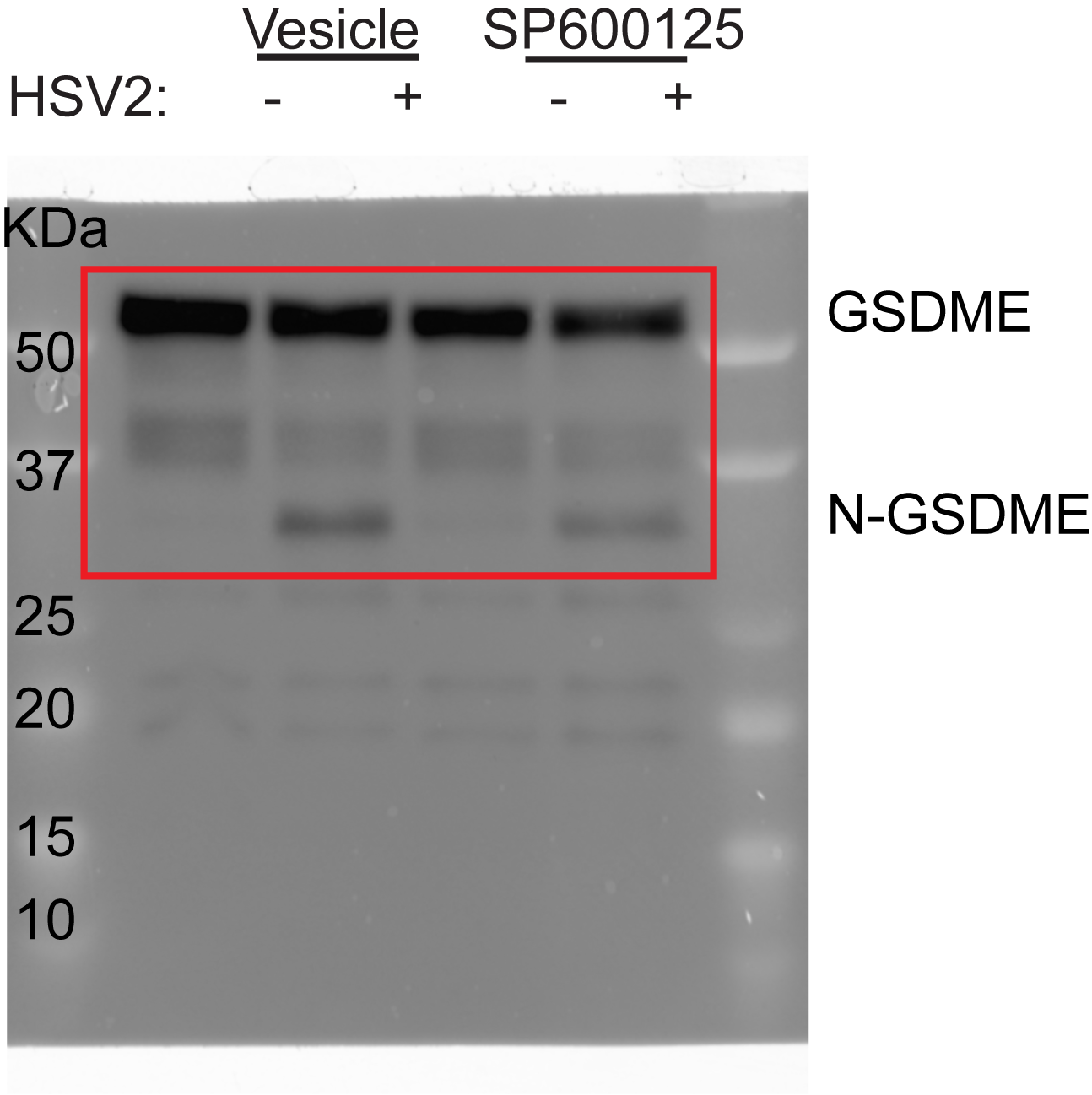

Supplement: Supplementary file 11 — Source Data for Figure 6 [file EMBJ-42-e113118-s002.zip › Source data Figure 6/6H/Weatern Blot GSDME.tif]

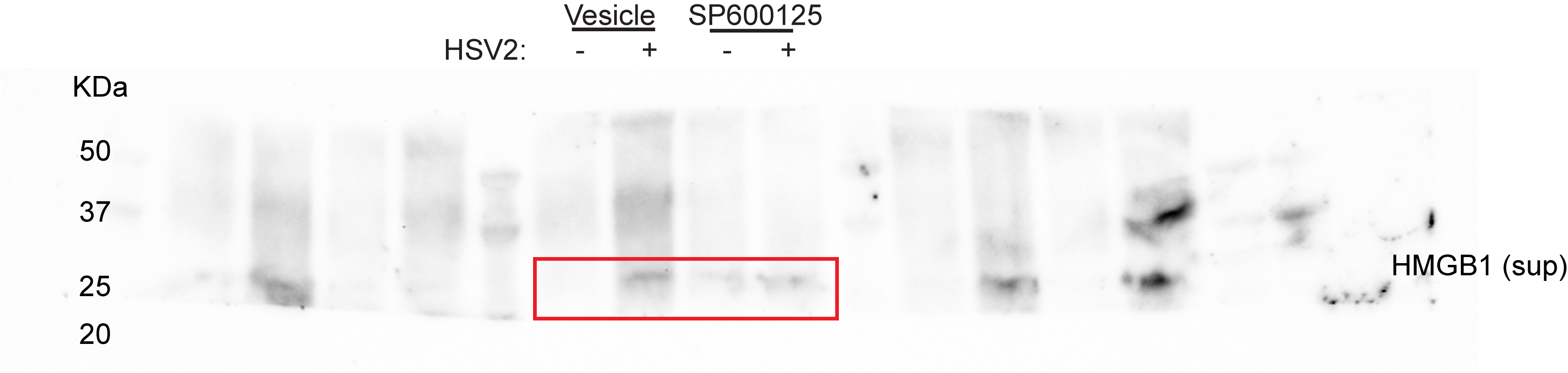

Supplement: Supplementary file 11 — Source Data for Figure 6 [file EMBJ-42-e113118-s002.zip › Source data Figure 6/6H/Weatern Blot HMGB1 (sup).tif]

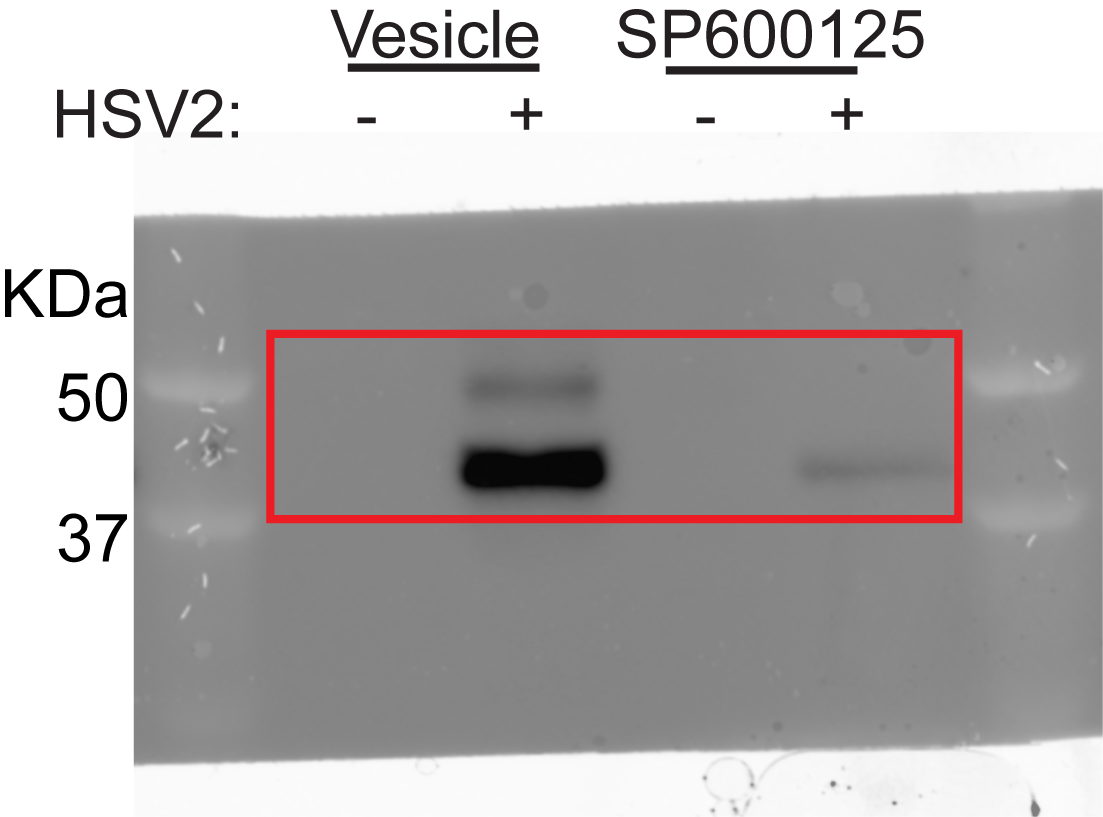

Supplement: Supplementary file 11 — Source Data for Figure 6 [file EMBJ-42-e113118-s002.zip › Source data Figure 6/6H/Weatern Blot p-JNK.tif]

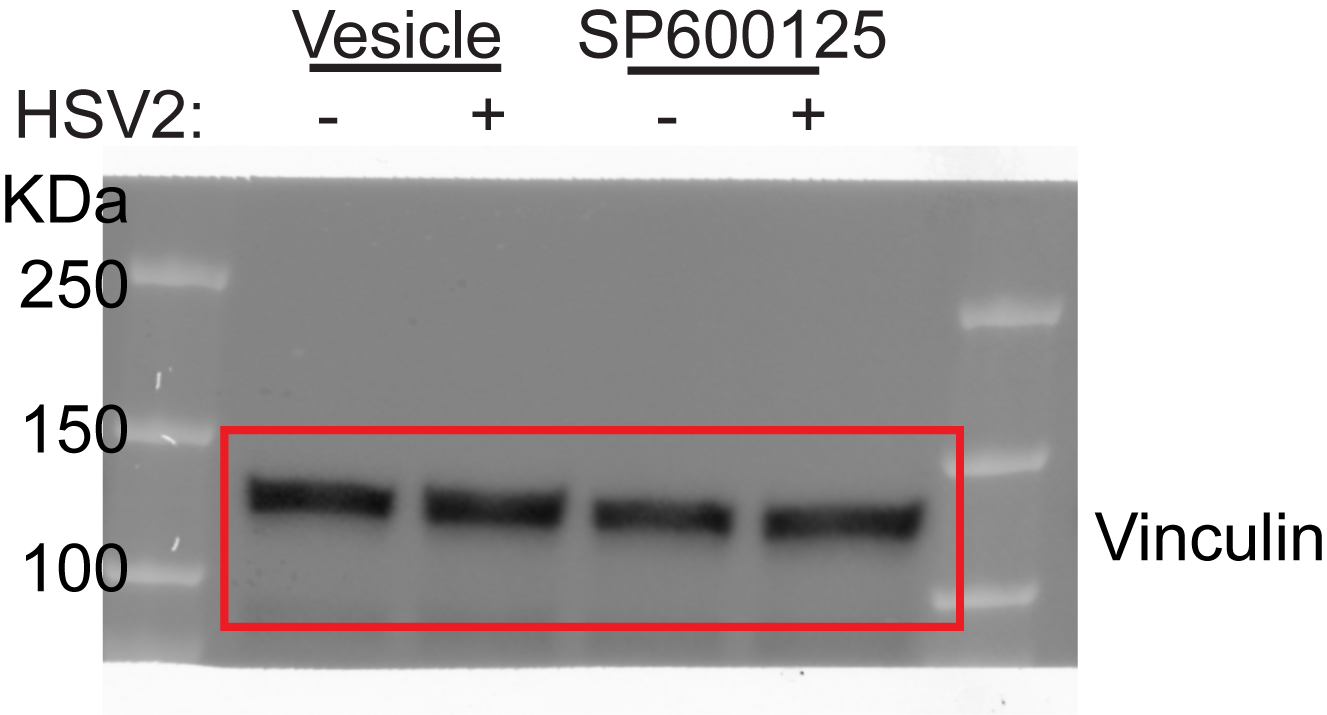

Supplement: Supplementary file 11 — Source Data for Figure 6 [file EMBJ-42-e113118-s002.zip › Source data Figure 6/6H/Weatern Blot Vinculin.tif]
