# Supplementary material for: ER stress induces caspase‐2‐tBID‐GSDME‐dependent cell death in neurons lytically infected with herpes simplex virus type 2
Source: EMBO J. 2023 Aug 30;42(19):e113118. doi: 10.15252/embj.2022113118 (PMC10548179; doi:10.15252/embj.2022113118)
Supplement: Supplementary file 12 [file EMBJ-42-e113118-s010.pdf]

THE EMBO JOURNAL

ER stress induces caspase-2-tBID-GSDME-dependent cell death in neurons lytically infected with herpes simplex virus type 2

Søren Paludan, Ryo Narita, Rasmus Bak, Georges Verjans, Line Reinert, Fanghui Ren, Ahmad Rashidi, Stefanie Stefanie Fruhwürth, Zongliang Gao, and Martin Thomsen
DOI: 10.15252/embj.2022113118

Corresponding author(s): Søren Paludan (srp@biomed.au.dk)

Review Timeline:

Submission Date:	22nd Nov 22
Editorial Decision:	18th Jan 23
Revision Received:	16th Jun 23
Editorial Decision:	2nd Aug 23
Revision Received:	14th Aug 23
Accepted:	14th Aug 23

Editor: Karin Dumstrei

Transaction Report:

(Note: With the exception of the correction of typographical or spelling errors that could be a source of ambiguity, letters and reports are not edited. Depending on transfer agreements, referee reports obtained elsewhere may or may not be included in this compilation. Referee reports are anonymous unless the Referee chooses to sign their reports.)

Hi Søren,

Thank you for submitting your point-by-point response. I appreciate the proposed changes and see that this will add new insight into the ER stress link.

I would therefore like to invite you to submit a revised manuscript along the lines as indicated in your response.

Let me know if we need to discuss anything further

When preparing your letter of response to the referees' comments, please bear in mind that this will form part of the Review Process File, and will therefore be available online to the community. For more details on our Transparent Editorial Process, please visit our website: https://www.embopress.org/page/journal/14602075/authorguide#transparentprocess

I thank you for the opportunity to consider your work for publication. I look forward to your revision.

with best wishes

Karin

Karin Dumstrei, PhD
Senior Editor
The EMBO Journal

Instructions for preparing your revised manuscript:

Please make sure you upload a letter of response to the referees' comments together with the revised manuscript.

Please also check that the title and abstract of the manuscript are brief, yet explicit, even to non-specialists.

When assembling figures, please refer to our figure preparation guideline in order to ensure proper formatting and readability in print as well as on screen:
https://bit.ly/EMBOPressFigurePreparationGuideline
See also guidelines for figure legends: https://www.embopress.org/page/journal/14602075/authorguide#figureformat

At EMBO Press we ask authors to provide source data for the main manuscript figures. Our source data coordinator will contact you to discuss which figure panels we would need source data for and will also provide you with helpful tips on how to upload and organize the files.

IMPORTANT: When you send the revision we will require
- a point-by-point response to the referees' comments, with a detailed description of the changes made (as a word file).
- a word file of the manuscript text.
- individual production quality figure files (one file per figure)
- a complete author checklist, which you can download from our author guidelines (https://www.embopress.org/page/journal/14602075/authorguide).
- Expanded View files (replacing Supplementary Information)
Please see out instructions to authors
https://www.embopress.org/page/journal/14602075/authorguide#expandedview

Please remember: Digital image enhancement is acceptable practice, as long as it accurately represents the original data and conforms to community standards. If a figure has been subjected to significant electronic manipulation, this must be noted in the figure legend or in the 'Materials and Methods' section. The editors reserve the right to request original versions of figures and the original images that were used to assemble the figure.

Further information is available in our Guide For Authors: https://www.embopress.org/page/journal/14602075/authorguide

We realize that it is difficult to revise to a specific deadline. In the interest of protecting the conceptual advance provided by the work, we recommend a revision within 3 months (18th Apr 2023). Please discuss the revision progress ahead of this time with the editor if you require more time to complete the revisions.

As a matter of policy, competing manuscripts published during this period will not negatively impact on our assessment of the conceptual advance presented by your study. However, we request that you contact the editor as soon as possible upon publication of any related work, to discuss how to proceed.

Use the link below to submit your revision:

https://emboj.msubmit.net/cgi-bin/main.plex

Referee #1:

In this study, the authors report the mechanism by which HSV-2 induces death of neuronal cells. The data presented support a model whereby Gasdermin E (GSDME) mediates cell death after caspase-3 and caspase-2 activation. Interestingly, the link to this death process was associated with a loss of MCL-1 and inactivation of BCL-2 family members, as has been reported for HSV-1 in other cell types. The novelty of this study lies in the detailed link to endoplasmic reticulum (ER) stress. This ER stress link has not been reported previously and should be explored in detail in a revision of this study in order to amplify the novelty of the work presented. Specific comments are indicated below:
1. In other contexts, ER stress induces translation "shutdown", a process that results in caspase-3 mediated GSDME activation in HSV-1 infected cells. While the authors verified that the protein synthesis inhibitor cycloheximide leads to pyroptosis, whether ER stress is causing translation shutdown should be explored. The authors are encouraged to examine this process.
2. What is the role of specific ER stress pathways in HSV-2 induced GSDME activation? The authors are encouraged to examine the function of PERK and IRE1, in particular.

Referee #2:

This study by Ren, et al, investigates the mechanism by which herpesvirus (HSV) infection causes neuronal cell death. The findings reported in this manuscript provide evidence for ER stress-induced pyroptotic cell death, dependent on Caspase-3 cleavage of Gasdermin E. Pyroptosis in this context required ER stress-dependent activation of caspase-2 and subsequent downstream signaling. Further, they report that the dying neurons released danger-associated molecules that could stimulate inflammatory responses in human microglia.

HSV2-driven neuronal cell death has been previously described but the type of cell death, and the associated consequences were not well defined. This study aimed to address that knowledge gap. The manuscript was clearly written, and the experiments well executed with most controls present. Strengths of the study include the use of multiple neuronal cell models, and creative approaches to triggering or ameliorating cell stress. While the data are generally consistent with their model, several substantive concerns limit my enthusiasm for the study in its current form. Major concerns include the absence of quantitative immunoblot analysis and notably, key evidence to establish viral drivers of endoplasmic reticulum stress is absent, which somewhat limits the novelty of the conclusions that the authors can support.

Major comments:
1. No image quantitation of immunoblots, e.g., no indication of replicability of immunoblots. Some of these assays, e.g., C3, could also be confirmed by enzymatic assays, but the blots should be quantitatively measured. This approach would also enable statistical analysis. Since the effects demonstrated are partial in many cases, careful quantitation is critical. This should not require new experiments, but rather image analysis of the existing replicates for each experiment. Quantitative immunoblot analysis is critical because the study conclusions rely so heavily on this method.
2. Depletion of GSDME protein should be confirmed to support phenotypes shown, e.g., Fig1 M and N, and should be standard for all depletion experiments. Confirmation of depletion was shown in other cases, like C2, but I could not find it for GSDME.
3. An LDH assay would more strongly support the effect of C2 in Fig. 3.
4. The authors rightly note the difference between CHX (~2 fold less) and ACV (~1.5 fold less) treatment on LDH release, calling the effect of ACV "minor". However, the data are shown as separate graphs and would benefit from a statistical analysis comparing the two conditions directly. This potential disconnect is interesting and suggests that some component of live HSV2 triggers ER stress. While the effect of HSV1 on the PERK pathway is established, I am not aware of how HSV2 drives ER stress, and some data to illuminate this point would strengthen the study, since many of the component parts demonstrated here have already been previously reported (eg. C3 known to cleave GSDME, C2 known to cause mitochondrial damage and C3 activation, etc.
5. The authors logically test different caspase inhibitors in Fig. EV3 for an effect on the GSDME cleavage phenotype but do not show any positive controls for the inhibitors that don't affect cleavage. Number of experimental replicates was not indicated in

the legend.
6. This statement is not convincingly supported based on the available data because of the lack of quantitation: "In summary, CASP2 is essential for cleavage of BID, tBID, in HSV-2- infected SH-SY5Y cells to mediate mitochondria-dependent pyroptosis, involving release of nucleic acids into the cytoplasm." While their data do support that nucleic acids in the cytosol can lead to GSDME cleavage (Fig. 4I), the effect of EtBr on GSDME and C3 cleavage in HSV2 infection was partial (Fig 4G); without quantitation and statistical analysis, it is insufficient to support their claim. Indeed, the authors did not observe accumulation of mtDNA.

Minor comments:
• Quite a few typographical errors, e.g., "endoplasmic reticulin" or "HSV-2-induced pyroptotic cell death....dependents on CASP2".
• Fig. 1E, make labeling consistent, e.g., HSV2 (in SHSY5Y) vs THP1
• The authors note that it is surprising that both C3 and C7 had to be disrupted to abolish GSDME cleavage, although it is quite well established in the literature that there is substantial biochemical and functional overlap in the substrate specificity of these two enzymes.
• The dependence of the THP1 inflammatory response (Fig 6 E-G) on STING was somewhat surprising. A comment in the Discussion on what component of the supernatant of infected neurons is likely to trigger STING signaling and how it would gain exposure to STING would be of interest to the reader.

Referee #3:

Summary:
This manuscript focuses on the manner by which HSV-2 causes death of neuronal cells. The authors report that lytic HSV infection of cell lines and primary neurons leads to pyroptotic cell death via gasdermin E pores. This cell death was reported to be downstream of virus-induced ER stress and cleavage of caspase-2 and caspase-3. The authors also report that the dying cells released alarmins that activated iPSC-derived microglia. Overall, I think the work presented in this manuscript is excellent and worthy of publication in EMBO, but I am not convinced that the authors can claim they are seeing pyroptosis since they have not shown any evidence of inflammasome activation or of the involvement of caspase-1, which is the signature pyroptosis caspase. Caspase-2 may be active in their system, but caspase-2 is no longer mentioned as an indicator of pyroptosis.

Regarding the significant of the work, although I feel this is more relevant to the virology field, the potential involvement of caspase-2 may make it relevant to any disease that involves inflammation.

Points for Consideration:
1. Results shown in Figure 1 are clear and the data support the conclusions drawn. One question: are HSV infections normally performed only for 24 hours? In other virus infections, maximal caspase activation and GSDM cleavage are more pronounced on days 3 or 4 p.i. even with higher MOIs of 1. I wonder if additional information might be gathered by letting the infections go a little longer. For example, in 1I and 1J, the cleaved GSDME band is only appearing at 16h, so perhaps longer infections would show more pronounced effects. I would be curious to see a 3 or 4 day time-course.

2. My concern with figure 1 is the term pyroptosis is assigned to the death observed, but I see no classical pyroptosis markers mentioned, such as activated caspase 1, an inflammasome sensor such as NLRP3 or AIM2, or downstream markers such as IL-1B or IL-18. Are the authors classifying this cell death as pyroptosis solely on the basis of GSDME and HMGB1 involvement? If so, I would like to see at least a demonstration that Caspase-1 is cleaved.

3. In Figure 2B, there doesn't seem to be any bands in the CASP1 blot, but CASP1 is not mentioned in the results with respect to this figure. Presumably, the antibody used recognizes uncleaved Caspase-1? Did the authors look for cleaved caspase-1? Again, if they are not finding cleaved caspase-1, how can they conclude pyroptosis is happening? The Figure legend for Figure 2B does not say whether this is casp1 or cleaved casp1 shown in the blot.

4. The data in Fig. 3 also point to apoptosis rather than pyroptosis. If Z-YVAD did not show any effect, but CASP3/7 inhibitors did show effects, this clearly implies that apoptosis is active and being blocked, as opposed to pyroptosis.

5. Figure 3A shows the presence of various caspases by Western, but there is no data presented to suggest these caspases are cleaved/active. It is not surprising to see pools of precursor caspases in various cell lines or tissues, but unless their cleavage is demonstrated using a cleaved caspase-specific antibody or chemical probe, we cannot assume these caspases are active or functioning.

6. There has not been a paper since 2013 discussing a role for caspase-2 in pyroptosis, and caspase-1 is broadly considered the hallmark caspase-1 that is active in pyroptosis. So although the authors have evidence for caspase-2 activity in their system, which is interesting on its own, I do not believe they can conclude this is indicative of pyroptosis taking place.

7. Regarding the data in Figure 4, I would not say it is required for this manuscript, but I suggest the authors think about the AIM2 inflammasome as HSV is known to stimulate AIM2. If they are seeing mtDNA release, there may be activation of AIM2-mediated pyroptosis, but this would still involve caspase-1.

Minor Points:
- Some very minor English/grammatical errors were noticed, for example, "Lytic HSV infection induce necrotic cell death..." and "To investigate for release of cytosolic content..."

- Most of the information provided in the top section of page 11 belongs in the discussion, i.e., the references to the work on IFI16 and NLRP3 as these were not studied in this paper.

Referee #1:

1. In other contexts, ER stress induces translation "shutdown", a process that results in caspase-3 mediated GSDME activation in HSV-1 infected cells. While the authors verified that the protein synthesis inhibitor cycloheximide leads to pyroptosis, whether ER stress is causing translation shutdown should be explored.

RE: Thank you for pointing this out, we agree with this comment and have tested whether ER stress cause translation shutdown using Coomassie blue staining. While cycloheximide treatment did affect cellular protein levels, the data did not suggest HSV-2 infection to lead to a major translational shutdown in SH-SY5Y cells (Figure R1 below). These data are presented in the revised manuscript as Fig. EV5F, and are described on page 12 line 4-6.

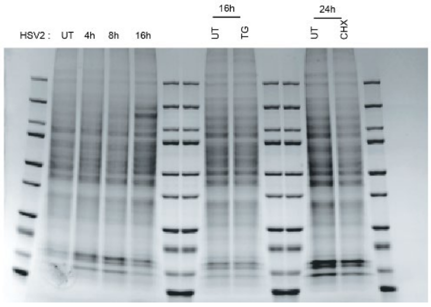

Fig R1. Effect of HSV-2 infection, thapsigargin and cyclohemimide treatment on total cellular protein levels. (*Fig.EV5Fin the manuscript*).

2. What is the role of specific ER stress pathways in HSV-2 induced GSDME activation? The authors are encouraged to examine the function of PERK and IRE1, in particular.

RE: We agree with this point and have therefore further characterized the mechanism of HSV-2-induced ER stress signaling to GSDME activation. *First*, we have added data on ATF6 and PERK to complete the analysis of the three major ER stress pathways. These data show that HSV-2-infected leads to degradation of ATF6 and does not activate PERK (as measured by p-PERK (Figure R2). *Second*, we have performed lentivirus-mediated overexpression of the ER stress pathway regulator BiP/GRP78, and found that it reduces HSV-2-mediated cleavage of GSDME (Figure R3). This data strengthen the results from the original manuscript using TUDCA as an ER stress inhibitor. *Third*, based on the above data we focused the analysis on IRE1α. CRISPR/Cas9 KO of *IRE1A* revealed a role for the IRE1α pathway in HSV-2-mediated GSDME cleavage. Since, IRE1α has both kinase and RNase activity, we used small molecules targeting the respective activities. Importantly, treatment with the IRE1α kinase inhibitor APY029 blocked HSV-2-induced GSDME cleavage and JNK phosphorylation, and treatment with the JNK inhibitor SP600125 strongly reduced HSV-2-induced GSDME cleavage (Fig R4). Inhibition of the IRE1α RNase inhibitors 4μ8C and STF-083010 had no/little effect on GSDME cleavage in infected cells, but forced activation of XBP1s splicing had an inhibitory effect on the response, consistent with previous reports on XBP1s and programmed cell death (e.g. PMID: 22669460) (Fig. R5). Collectively, these data suggest that HSV-2-infected neuronal cells activate both the IRE1α-JNK and the IRE1α-XBP1s pathways and the death-promoting activity of the IRE1α-JNK pathway is responsible for the pyroptosis. These new results are presented in the revised manuscript as Fig 5A, 5L, 6A-F, and EV6A-D, and described/discussed on page 11 line 17-19, 22-23.

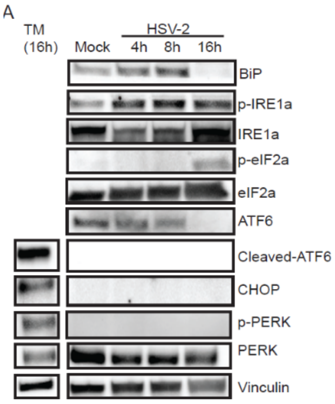

Fig R2. Lytic HSV-2 infection activates marker of for the IRE1a, but not the PERK and ATF6 pathways. (*Fig 5A in the manuscript*).

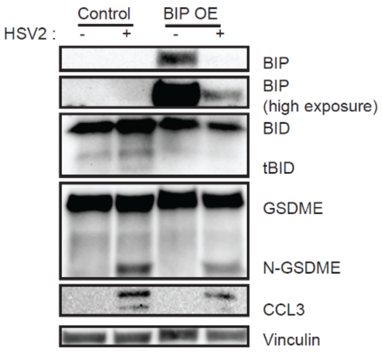

Fig R3. Lentiviral expression of BIP in SH-SY5Y cells reduces HSV-2-induced cleavage of BID, CASP3, and GSDME (*Fig 5L in the manuscript*).

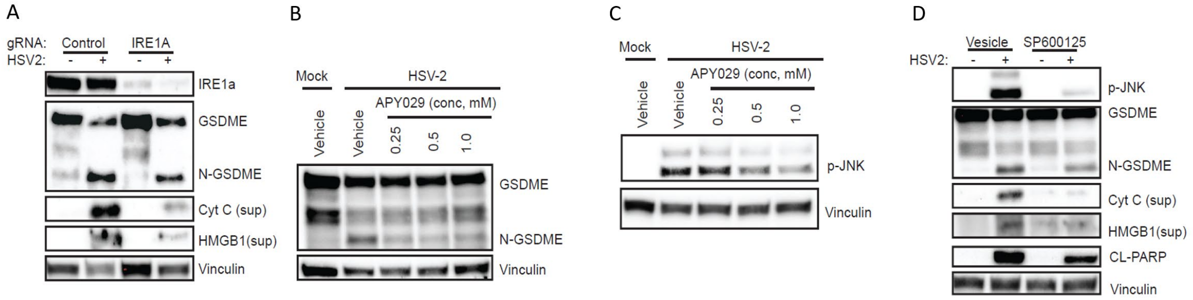

Fig R4. Inhibition of IRE1α expression or kinase activity blocks HSV-2-induced signaling to GSDME-driven pyroptosis ((*Fig 6E-H in the manuscript*).

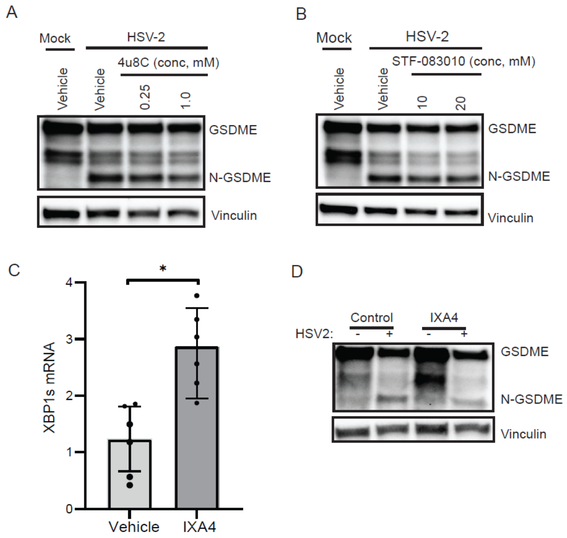

Fig R5. Modulation of IRE1α RNase activity had modest effect on HSV-2-induced signaling to GSDME-driven pyroptosis ((*Fig EV6A-D in the manuscript*).

Referee #2:

Major comments:

1. No image quantitation of immunoblots, e.g., no indication of replicability of immunoblots. Some of these assays, e.g., C3, could also be confirmed by enzymatic assays, but the blots should be quantitatively measured. This approach would also enable statistical analysis. Since the effects demonstrated are partial in many cases, careful quantitation is critical. This should not require new experiments, but rather image analysis of the existing replicates for each experiment. Quantitative immunoblot analysis is critical because the study conclusions rely so heavily on this method.

RE: Thanks for your suggestions. We agree that quantification of the immunoblots will make the data more clear. This is particularly the interpretation of blots for N-GSDME and tBID, which can be challenging (due to sometimes partial effects of a given treatment/genetic manipulation). Therefore, we have focused the quantification and statistical analysis of immunoblots on N-GSDME and tBID, which is compiled in an Excel sheet appended to the submission. If requested, this spreadsheet can be added to the manuscript. In addition, we agree that key results from immunoblotting for cleaved caspase 3 could be confirmed by caspase 3 activity assays. Therefore, we have examined for HSV-2-induced activation of Caspase 3, and its dependence on Caspase 2, BID, and ER stress (TUDCA treatment) (Fig R6). These data are shown in the revised manuscript as Fig 1F, 3C, 4C and EV5H, and the results and methods sections are updated accordingly (page 7 line 16-17; page 9 line 31-33; page 10 line 12-15; page 12, line 12-14; page 30 line 20-31).

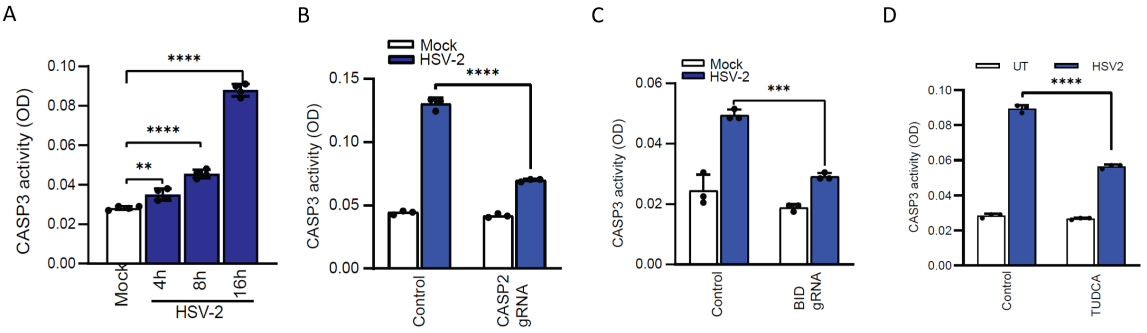

Fig R6. Activation of CASP3 activity in HSV-2-infected SH-SY5Y cells, and the effect of modulation of various factors involved in the signaling mechanism (*Fig. 1F, 3C, 4C and EV5H in the manuscript*).

2. Depletion of GSDME protein should be confirmed to support phenotypes shown, e.g., Fig1 M and N, and should be standard for all depletion experiments. Confirmation of depletion was shown in other cases, like C2, but I could not find it for GSDME.

RE: We thank for pointing this out, and we have now added the missing immunoblot for GSDME to Fig.1O. (See Fig R7 below).

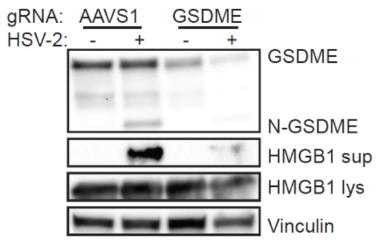

Fig R7. Efficiency of GSDME protein depletion following GSDME gRNA treatment. (*Fig 1O in the manuscript*).

3. An LDH assay would more strongly support the effect of C2 in Fig. 3.

RE: The authors agree on this point, and we have therefore tested the effect of CASP2 KO on HSV-2-induced LDH release. The data show that loss of CASP2 leads to a significant reduction in LDH release from HSV-2-infected SH-SY5Y cells (Fig. R8). The data is added to the revised manuscript as Fig. 3F, and described in the text on page 9 line 34 – page 10 line 2.

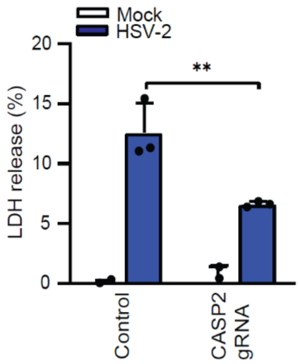

Fig R8. CASP2 depletion inhibits LDH release from HSVB-2-infected cells. (*Fig. 3F in the manuscript*).

4. The authors rightly note the difference between CHX (~2 fold less) and ACV (~1.5 fold less) treatment on LDH release, calling the effect of ACV "minor". However, the data are shown as separate graphs and would benefit from a statistical analysis comparing the two conditions directly. This potential disconnect is interesting and suggests that some component of live HSV2 triggers ER stress. While the effect of HSV1 on the PERK pathway is established, I am not aware of how HSV2 drives ER stress, and some data to illuminate this point would strengthen the study, since many of the component parts demonstrated here have already been previously reported (e.g. C3 known to cleave GSDME, C2 known to cause mitochondrial damage and C3 activation, etc.

RE: The author's description of the results on the effect of ACV on necrotic cell death markers, was collective for the data on LDH release and HMGB1 (Fig. EV1C-I.). While there was clearly some effect of ACV treatment on LDH release, there was no/minimal effect on HMGB1. This was in contrast to CHX treatment, where both readouts were affected. On the basis of this we chose to describe the two series of data with the wordings *"... both .... protein synthesis were required for GSDME cleavage in SH-SY5Y cells*" and "*ACV treatment, which inhibited .... and LDH release, had only a minor effect on GSDME cleavage and release of HMGB1*". We believe these data rightfully reflect the data.
Regarding question that more data is needed on the mechanism of HSV-activated ER stress-induced pyroptosis, we fully agree with this point. Therefore, we have added a full new main figure on this in the revised manuscript (Fig. 6). This includes new data inspired by the suggestions from reviewer 2. Please see response to reviewer 1, point 2 and Fig R2-R5 for selected new data.

5. The authors logically test different caspase inhibitors in Fig. EV3 for an effect on the GSDME cleavage phenotype but do not show any positive controls for the inhibitors that don't affect cleavage. Number of experimental replicates was not indicated in the legend.

RE: We would like to thank the reviewer for pointing this out. We have now added several positive controls (Fig. R9). For instance, LPS treatment (1ug/ml, 4h) followed by nigericin treatment (10uM,+1h) was used to induce cleavage CASP1 in THP1 cells, and hence to provide a positive control for the action of Z-YVAD. Stauroporine treatment (1uM, 8h) was used to induce cleavage of CASP6/8/9 in SH-SY5Y cells, which allowed us to demonstrate targeting of these caspases with the relevant inhibitors. The new data are presented as Fig. EV3A-D and described on page 9 line 12-23.

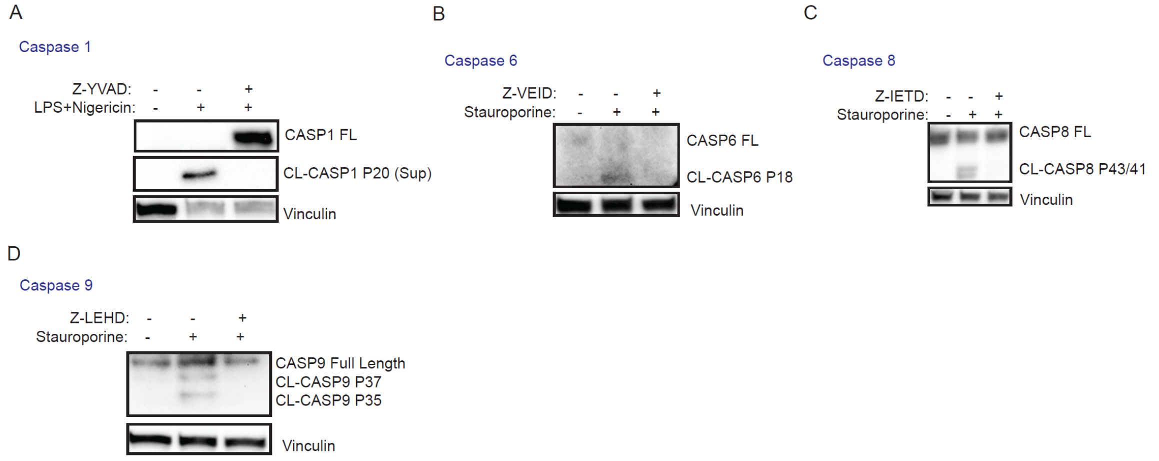

Fig R9. Positive controls for caspase inhibitors used in the study. (*Fig. EV3A-D in the manuscript*).

6. This statement is not convincingly supported based on the available data because of the lack of quantitation: "In summary, CASP2 is essential for cleavage of BID, tBID, in HSV-2- infected SH-SY5Y cells to mediate mitochondria-dependent pyroptosis, involving release of nucleic acids into the cytoplasm." While their data do support that nucleic acids in the cytosol can lead to GSDME cleavage (Fig. 4I), the effect of EtBr on GSDME and C3 cleavage in HSV2 infection was partial (Fig 4G); without quantitation and statistical analysis, it is insufficient to support their claim. Indeed, the authors did not observe accumulation of mtDNA.

RE: Thanks for the suggestion. We agree on the point raised, and have therefore, now made the requested quantifications and statistical analyses for GSDME cleavage and tBID (Appended Excel sheet). Presentation of the data in this manner, including all datasets, still support the conclusions drawn in the original manuscript.
As to the accumulation of mtDNA, the Smiley lab has reported that HSV infection leads to rapid elimination of mtDNA in host cells without affecting HSV infection (PMID: 17186027, PMID: 24371054). This may explain why we did not detect accumulation of mtDNA, although we still identified a functional role for the released mtDNA in cell death. A sentence on this issue (and the above references) have been added to the discussion (page 16 line 9-12).

Minor comments:
• Quite a few typographical errors, e.g., "endoplasmic reticulin" or "HSV-2-induced pyroptotic cell death....dependents on CASP2".
RE: The manuscript has been proof-read by a person with experience as language editor for two scientific journals.

• Fig. 1E, make labeling consistent, e.g., HSV2 (in SHSY5Y) vs THP1
RE: Thank you for identifying this mistake from our side. It has been corrected in the revised figures (now Fig. 1E).

• The authors note that it is surprising that both C3 and C7 had to be disrupted to abolish GSDME cleavage, although it is quite well established in the literature that there is substantial biochemical and functional overlap in the substrate specificity of these two enzymes.
RE: This is a valid point, and we have updated the text to be more aligned with the state-of-the-art in the field on this issue (page 15 line 26-29).

• The dependence of the THP1 inflammatory response (Fig 6 E-G) on STING was somewhat surprising. A comment in the Discussion on what component of the supernatant of infected neurons is likely to trigger STING signaling and how it would gain exposure to STING would be of interest to the reader.
RE: This is a good point, and the issue of what may be the DAMP driving the microglial activation should be discussed. This has been added to the revised manuscript (page 18 line 18-25).

Referee #3:

Summary:
This manuscript focuses on the manner by which HSV-2 causes death of neuronal cells. The authors report that lytic HSV infection of cell lines and primary neurons leads to pyroptotic cell death via gasdermin E pores. This cell death was reported to be downstream of virus-induced ER stress and cleavage of caspase-2 and caspase-3. The authors also report that the dying cells released alarmins that activated iPSC-derived microglia. Overall, I think the work presented in this manuscript is excellent and worthy of publication in EMBO, but I am not convinced that the authors can claim they are seeing pyroptosis since they have not shown any evidence of inflammasome activation or of the involvement of caspase-1, which is the signature pyroptosis caspase. Caspase-2 may be active in their system, but caspase-2 is no longer mentioned as an indicator of pyroptosis.

Regarding the significant of the work, although I feel this is more relevant to the virology field, the potential involvement of caspase-2 may make it relevant to any disease that involves inflammation.

Points for Consideration:
1. Results shown in Figure 1 are clear and the data support the conclusions drawn. One question: are HSV infections normally performed only for 24 hours? In other virus infections, maximal caspase activation and GSDM cleavage are more pronounced on days 3 or 4 p.i. even with higher MOIs of 1. I wonder if additional information might be gathered by letting the infections go a little longer. For

example, in 1I and 1J, the cleaved GSDME band is only appearing at 16h, so perhaps longer infections would show more pronounced effects. I would be curious to see a 3 or 4 day time-course.

RE: In the cell model used, HSV-2 infection is very aggressive, the programmed cell death signaling is strong at around 16 h p.i., and lysis is very actively occurring at 24 h. At later time points, the authors find that a too large proportion of the cells are dead, making meaningful interpretation of the data difficult. Therefore, the 24 h time point was chosen. In the less permissive cells tested, e.g. neuronal progenitor cells the lytic infection progresses more slowly, and the accompanying GSDME-cleavage exhibits a delayed kinetics (Fig 2A-C).

2. My concern with figure 1 is the term pyroptosis is assigned to the death observed, but I see no classical pyroptosis markers mentioned, such as activated caspase 1, an inflammasome sensor such as NLRP3 or AIM2, or downstream markers such as IL-1B or IL-18. Are the authors classifying this cell death as pyroptosis solely on the basis of GSDME and HMGB1 involvement? If so, I would like to see at least a demonstration that Caspase-1 is cleaved.

RE: The authors thank the reviewer for this comment, and apologize for not being sufficiently clear on what we define as pyroptosis. We agree that in classical GSDMD-dependent pyroptosis, inflammasome sensors like NLRP3 and AIM2 are well studied to activate caspase 1, leading to GSDMD cleavage and pyroptosis. However, the markers used in classical pyroptosis are not suitable to describe non-classical pyroptosis dependent on other gasdermins, such as GSDME. The definition of pyroptosis used in this study is "Gasdermin-mediated cell death", as proposed by others (PMID: 33692549). This is now specified in the revised manuscript (page 5 line 13-15). In the cell model used in this study CASP1, GSDMD are not expressed (Fig 1D, 3A), and we have now added new data showing that neither NLRP3 or AIM2 are expressed (Fig R10). Together with the body of data in the revised manuscript supporting caspase 3/7 and GSDME to be involved, we propose that lytic HSV-2 infection induces caspase 3/7-dependent GSDME-executed pyroptosis.

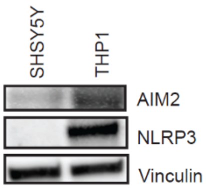

Fig R10. Expression of AIM2 and THP1 cells in SHSY5Y and THP1 cells (*Fig. EV4H in the manuscript*).

3. In Figure 2B, there doesn't seem to be any bands in the CASP1 blot, but CASP1 is not mentioned in the results with respect to this figure. Presumably, the antibody used recognizes uncleaved Caspase-1? Did the authors look for cleaved caspase-1? Again, if they are not finding cleaved caspase-1, how can they conclude pyroptosis is happening? The Figure legend for Figure 2B does not say whether this is casp1 or cleaved casp1 shown in the blot.

RE: As we read this comment, it is related to point 2 above. We are using the definition of pyroptosis as "Gasdermin-mediated cell death" (PMID: 33692549), and hence not limited to CASP1-GSDMD-mediated cell death. Therefore, the lack of CASP1 expression, or lack of effect of CASP1 inhibition, does not threaten the conclusions drawn in the manuscript. As stated in the point above, we have now clarified the definition of pyroptosis in the introduction (page 5 line 13-15).

4. The data in Fig. 3 also point to apoptosis rather than pyroptosis. If Z-YVAD did not show any effect, but CASP3/7 inhibitors did show effects, this clearly implies that apoptosis is active and being blocked, as opposed to pyroptosis.

RE: The authors agree that apoptotic events are activated in the lytically infected cells (e.g. cleavage and activation of caspase 3). However, we also observed release intracellular proteins, such as HMGB1 and LDH, indicators of necrosis. Flow Cytometry analysis of HSV-2-infected cells for Annexin V and PI showed significant increase in PI but not Annexin V positive cells, which indicates that cell damage is mainly caused by membrane disruption (Fig. 1H in revised manuscript), and not the full apoptosis pathway involving Annexin V surface exposure. Finally, the GSDME-dependent nature of the necrotic cell death leads us to conclude that it is pyroptosis.

5. Figure 3A shows the presence of various caspases by Western, but there is no data presented to

suggest these caspases are cleaved/active. It is not surprising to see pools of precursor caspases in various cell lines or tissues, but unless their cleavage is demonstrated using a cleaved caspase-specific antibody or chemical probe, we cannot assume these caspases are active or functioning.
RE: In the original version of the manuscript, we first used expression of caspases as initial criteria for further examination (Fig. 3A). Second, we used small molecule caspase inhibitors and CRISPR KO to functionally test the involvement of expressed caspases (Fig. 3 and EV3). For the caspases found to be involved in HSV-2-induced cell death, we presented data showing cleavage of CASP3 and activation of CASP2 e.g. (Fig. 1F and 3E). In the revised manuscript, we additionally demonstrate that the cleaved caspase 3 is indeed active (Fig. R6, related to reviewer #2 point 1). Based on this collected set of data, the authors are comfortable to conclude that CASP 3 (7), and 2 are involved in pyroptosis in neuronal cells lytically infected with HSV-2.

6. There has not been a paper since 2013 discussing a role for caspase-2 in pyroptosis, and caspase-1 is broadly considered the hallmark caspase-1 that is active in pyroptosis. So although the authors have evidence for caspase-2 activity in their system, which is interesting on its own, I do not believe they can conclude this is indicative of pyroptosis taking place.
RE: We agree that caspase-1-regulated inflammasome-driven-pyroptosis is broadly studied. However, the present work centers on inflammasome-independent pyroptosis, mediated by GSDME. For this process, much less is known, and it is in most cases independent of caspase 1. Regarding CASP2, we report a role for this caspase in relaying signaling from ER stress to mitochondrial disruption, including for instance mediating BID cleavage (Fig. 4F). Downstream of the mitochondria, our data suggest CASP3/7 to be responsible for cleavage of GSDME (Fig. EV1L-M). Both cleavage of CASP3 and GSDME are dependent on CASP2 (Fig. 3C). Based on these and other data, we find it well justified to conclude that HSV-2-induced pyroptosis is dependent on CASP2.

7. Regarding the data in Figure 4, I would not say it is required for this manuscript, but I suggest the authors think about the AIM2 inflammasome as HSV is known to stimulate AIM2. If they are seeing mtDNA release, there may be activation of AIM2-mediated pyroptosis, but this would still involve caspase-1.
RE: As we explained above, and documented by data in Fig. EV3A and E, caspase-1 does not mediate the pyroptosis observed in our model. Moreover, similar to NLRP3, AIM2 was not expressed in the SH-SY5Y cells (Fig R10). Therefore, since these data exclude an AIM2-inflammasome as the effector of GSDMD-dependent pyroptosis, we have not focused much on NLRP3/AIM2-CASP1-dependent pyroptosis.

Minor Points:
- Some very minor English/grammatical errors were noticed, for example, "Lytic HSV infection induce necrotic cell death..." and "To investigate for release of cytosolic content..."
RE: The manuscript has been proof-read by a person with experience as language editor for two scientific journals.

- Most of the information provided in the top section of page 11 belongs in the discussion, i.e., the references to the work on IFI16 and NLRP3 as these were not studied in this paper.
RE: The authors find this to be a valid point of criticism, and we have therefore deleted the more discussing part of the text in the top section of page 11, and added some of it to the discussion (page 16 line7-9).

1st Revision - Editorial Decision 2nd Aug 2023

Dear Søren,

Thank you for submitting your revised manuscript to The EMBO Journal. Your study has now been seen by the original referees #1 and 3 and their comments are provided below. As you can see, referee #3 raises some relevant concerns if what you see is indeed pyroptosis based on the definition of pyroptosis as Gasdermin-mediated cell death. I do realize that the terminology is shifting and debated in this field between inflammasome-dependent cell death vs Gasdermins as the defining features of pyroptosis.

I have discussed this issue with both referees and would like to propose that you in the introduction discuss this issue in a balanced way. This would then allow you define the terminology you will use in the study while acknowledging differing opinions in the field.

Please also use the discussion to further discuss this point.

When you submit the revised version, please also take care of the following editorial points:

- We can only have 5 keywords

- You are missing a Data Availability section. This is the place to enter accession numbers etc. If no data is generated that needs to be deposited in a databasethen please state: Data Availability: This study includes no data deposited in external repositories.

- COI: needs to be renamed to "Disclosure Statement and Competing Interests section"

- Please remove the Authors Contributions from the manuscript. The 'Author Contributions' section is replaced by the CRediT contributor roles taxonomy to specify the contributions of each author in the journal submission system. Please use the free text box in the 'author information' section of the manuscript submisssion system to provide more detailed descriptions (e.g., 'X provided intracellular Ca++ measurements in fig Y').

- Individual panels of EV3 need to be called out

- The synopsis image is too large it should be 550 wide by 400.

- Please upload the synopsis text as a separate file and remove from MS.

- The EV Figures need to be upload as separate files with the figure legends in the manuscript text. Please note that you can only have 5 EV figures. The remaining two EV figures would have to go into an appendix with a ToC. Please note that call outs for Appendix figures should be Appendix Figure S1, etc.

- EXPERIMENTAL PROCEDURES should be labelled as Materials and Methods?

- Please also upload source data as per email from 25th January from Hannah Sonntag. We would also like to ask for source data for EV3&EV4.

For main figures please upload one file/folder (zipped) per figure labelled Source Data figure 1 etc.

For the EV figures: zip all source data files in one file labelled Source Data EV figures

For Appendix figures: zip all source data files in one file labelled Source Data Appendix figures

If you have source data for both appendix and EV figures zip both files in one labelled Source Data Appendix and EV figures

- Our publisher has also done their pre-publication check on your manuscript. When you log into the manuscript submission system you will see the file "Data Edited Manuscript file". Please look at the word file and the comments regarding the figure legends and respond to the issues.

- Please check if Vinculin is blot reused in Figure 4B and 4E and in Figure 5A and 6C. If so then indicate in figure legend.

That should be all. Let me know if we need to discuss anything further

With best wishes

Karin

Karin Dumstrei, PhD
Senior Editor
The EMBO Journal

Instructions for preparing your revised manuscript:

Guide For Authors: https://www.embopress.org/page/journal/14602075/authorguide

Use the link below to submit your revision:

https://emboj.msubmit.net/cgi-bin/main.plex

Referee #1:

In this revised manuscript, the authors have addressed each of my concerns. The model presented is supported by the evidence in this study and I commend the authors on this discovery.

I have no additional concerns.

Referee #3:

As mentioned in previous reviews, I am happy with the quality of the work included in this manuscript. However, I cannot accept the authors' conclusion that they are seeing pyroptosis as they have yet to demonstrate involvement of any component of pyroptosis other than GSDM.

In response to this concern in the previous review, the authors have provided a single review article that they say defined pyroptosis as "Gasdermin-mediated cell death". Upon examination of that review, this definition is not actually stated. I agree that there is significant crosstalk between different forms of cell death, but speaking as someone who has been studying pyroptosis for 10 years, I am confident that no one defines pyroptosis based solely on GSDM involvement. Pyroptosis is inflammasome-mediated cell death, and there is no evidence in this manuscript of inflammasome involvement.

As mentioned in previous reviews, the authors are confusing cell death terminology. Even in the rebuttal letter, in point #4, they state "GSDME-dependent nature of the necrotic cell death leads us to conclude that it is pyroptosis". Pyroptosis is not necrotic, it is programmed cell death, and anyone who truly understands pyroptosis would not refer to pyroptosis as necrotic cell death.

Not all GSDM-mediated death is considered pyroptosis, so we cannot conclude pyroptosis is happening just because there is GSDM cleavage.

Reviewer 3

As mentioned in previous reviews, I am happy with the quality of the work included in this manuscript. However, I cannot accept the authors' conclusion that they are seeing pyroptosis as they have yet to demonstrate involvement of any component of pyroptosis other than GSDM.

In response to this concern in the previous review, the authors have provided a single review article that they say defined pyroptosis as "Gasdermin-mediated cell death". Upon examination of that review, this definition is not actually stated. I agree that there is significant crosstalk between different forms of cell death, but speaking as someone who has been studying pyroptosis for 10 years, I am confident that no one defines pyroptosis based solely on GSDM involvement. Pyroptosis is inflammasome-mediated cell death, and there is no evidence in this manuscript of inflammasome involvement.

As mentioned in previous reviews, the authors are confusing cell death terminology. Even in the rebuttal letter, in point #4, they state "GSDME-dependent nature of the necrotic cell death leads us to conclude that it is pyroptosis". Pyroptosis is not necrotic, it is programmed cell death, and anyone who truly understands pyroptosis would not refer to pyroptosis as necrotic cell death.

Not all GSDM-mediated death is considered pyroptosis, so we cannot conclude pyroptosis is happening just because there is GSDM cleavage.

Editorial comment: I have discussed this issue with both referees and would like to propose that you in the introduction discuss this issue in a balanced way. This would then allow you define the terminology you will use in the study while acknowledging differing opinions in the field.
Please also use the discussion to further discuss this point.

RE: The authors are aware of the non-unanimous use of the term pyroptosis. While it was originally defined to be caspase-1-dependent cell death, this developed into inflammasome-dependent cell death when this supermolecular structure for caspase-1 activation was discovered. Later, this further developed into GSDMD-dependent cell death when cleaved GSDMD was discovered to form the caspase-1-cleaved pore mediating cell death. Most recently, the identification of several gasdermins that can form death-inducing pores upon cleavage has led some investigators to expand the term pyroptosis to all forms of gasdermin-induced cell death. This includes several of the previous articles on cell death induced by virus replication / cell stress (e.g. PMID: 33979579; 37367489; 37212338; 37202386), and also reviews in leading journal (e.g. PMID: 36543934; 37352727; 33692549). We have mainly been following the above literature, but fully acknowledge that the the definition of the term "pyroptosis" is not broadly agreed upon. Therefore, we agree that in order to avoid confusion, our work should be very clear on the definition used. This has been added to the revised manuscript in the introduction on page 4 line 13-16 and line 25-28., and we have also added a passage to clarify the use of the terms in the discussion page 14 line 17-18. Moreover, in order not to "take side" in an ongoing discussion on terminology, which is important – but not a major goal for the present work - we have also changed the wording in the title, abstract, and synopsis to not include the term "pyroptosis" but rather "*GSDME-mediated cell death*" or just "*cell death*".

Editorial points:

- We can only have 5 keywords

The list of keywords has been updated.

- You are missing a Data Availability section. This is the place to enter accession numbers etc. If no data is generated that needs to be deposited in a databasethen please state: Data Availability: This study includes no data deposited in external repositories.
Description of data availability has been added.

- COI: needs to be renamed to "Disclosure Statement and Competing Interests section"
Done

- Please remove the Authors Contributions from the manuscript. The 'Author Contributions' section is replaced by the CRediT contributor roles taxonomy to specify the contributions of each author in the journal submission system. Please use the free text box in the 'author information' section of the manuscript submisssion system to provide more detailed descriptions (e.g., 'X provided intracellular Ca++ measurements in fig Y').
The Author contribution section has been deleted, and we have given full information in the CRediT contributor roles taxonomy through the journal submission system.

- Individual panels of EV3 need to be called out
On page 8 there all call-outs to the individual panels of Fig EV3.

- The synopsis image is too large it should be 550 wide by 400.
The synopsis image has been modified and is now 550w x 515h.

- Please upload the synopsis text as a separate file and remove from MS.
We have now submitted the synopsis as a separate file.

- The EV Figures need to be upload as separate files with the figure legends in the manuscript text. Please note that you can only have 5 EV figures. The remaining two EV figures would have to go into an appendix with a ToC. Please note that call outs for Appendix figures should be Appendix Figure S1, etc.
In the present submission, we have uploaded the EV figures and added their legends to the manuscript text. Regarding the limit of fine EV figure, we have changed the previous EV Fig 2 and 7 to Appendix Figure S1 and S2, respectively. The manuscript is updated accordingly.

- EXPERIMENTAL PROCEDURES should be labelled as Materials and Methods?
Done!

- Please also upload source data as per email from 25th January from Hannah Sonntag. We would also like to ask for source data for EV3&EV4.
Now included in the submitted source data.

For main figures please upload one file/folder (zipped) per figure labelled Source Data figure 1 etc.
Done!

For the EV figures: zip all source data files in one file labelled Source Data EV figures
Done!

For Appendix figures: zip all source data files in one file labelled Source Data Appendix figures
Done!

If you have source data for both appendix and EV figures zip both files in one labelled Source Data Appendix and EV figures

The source data for appendix and EV figures are in separate folders.

- Our publisher has also done their pre-publication check on your manuscript. When you log into the manuscript submission system you will see the file "Data Edited Manuscript file". Please look at the word file and the comments regarding the figure legends and respond to the issues.

The manuscript included three comments:

"AB1: Please check corrections to p value definition, here and elsewhere". I have done this now and the changes made are correct.

"AB2: Please check corrections. I have done this, and the correction is correct".

*"AB3: Please define the number and the nature, i.e. biological or technical, of the replicates. Please define the bars and error bars, e.g. mean ± SD. Please define the annotated p-value(s), e.g. *P < 0.05. Please define the statistical test."* This information has now need added to the updated legend for EV Figure 5.

- Please check if Vinculin is blot reused in Figure 4B and 4E and in Figure 5A and 6C. If so then indicate in figure legend.

Vinculin blots in Figure 4B has been reused in 4E. This information has been added to the figure legend. The Vinculin blots in Figure 5A and 6C are different.

Hi Søren,

Thank you for submitting your revised manuscript to the EMBO Journal. I have now had a chance to look at it and all looks good. I am therefore very pleased to accept that manuscript for publication here!

PS I can't find the synopsis text - can you send it to me via email?

Congratulations on a nice study!

Best Karin

Karin Dumstrei, PhD
Senior Editor
The EMBO Journal

Please note that it is EMBO Journal policy for the transcript of the editorial process (containing referee reports and your response letter) to be published as an online supplement to each paper. If you do NOT want this, you will need to inform the Editorial Office via email immediately. More information is available here: https://www.embopress.org/page/journal/14602075/authorguide#transparentprocess

Your manuscript will be processed for publication in the journal by EMBO Press. Manuscripts in the PDF and electronic editions of The EMBO Journal will be copy edited, and you will be provided with page proofs prior to publication. Please note that supplementary information is not included in the proofs.

You will be contacted by Wiley Author Services to complete licensing and payment information. The required 'Page Charges Authorization Form' is available here: https://www.embopress.org/pb-assets/embo-site/tej_apc.pdf - please download and complete the form and return to embopressproduction@wiley.com

EMBO Press participates in many Publish and Read agreements that allow authors to publish Open Access with reduced/no publication charges. Check your eligibility: https://authorservices.wiley.com/author-resources/Journal-Authors/open-access/affiliation-policies-payments/index.html

Should you be planning a Press Release on your article, please get in contact with embojournal@wiley.com as early as possible, in order to coordinate publication and release dates.

If you have any questions, please do not hesitate to call or email the Editorial Office. Thank you for your contribution to The EMBO Journal.

** Click here to be directed to your login page: https://emboj.msubmit.net

EMBO Press Author Checklist

Corresponding Author Name: Soren R Paludan
Journal Submitted to: The EMBO Journal
Manuscript Number: EMBOJ-2022-113118R

USEFUL LINKS FOR COMPLETING THIS FORM
The EMBO Journal - Author Guidelines
EMBO Reports - Author Guidelines
Molecular Systems Biology - Author Guidelines
EMBO Molecular Medicine - Author Guidelines

Reporting Checklist for Life Science Articles (updated January
This checklist is adapted from Materials Design Analysis Reporting (MDAR) Checklist for Authors. MDAR establishes a minimum set of requirements in transparent reporting in the life sciences (see Statement of Task: 10.31222/osf.io/9sm4x). Please follow the journal's guidelines in preparing your
Please note that a copy of this checklist will be published alongside your article.

Abridged guidelines for figures
1. Data
The data shown in figures should satisfy the following conditions:
- the data were obtained and processed according to the field's best practice and are presented to reflect the results of the experiments in an accurate and unbiased manner.
- ideally, figure panels should include only measurements that are directly comparable to each other and obtained with the same assay.
- plots include clearly labeled error bars for independent experiments and sample sizes. Unless justified, error bars should not be shown for technical
- if n<5, the individual data points from each experiment should be plotted. Any statistical test employed should be justified.
- Source Data should be included to report the data underlying figures according to the guidelines set out in the authorship guidelines on Data

2. Captions
Each figure caption should contain the following information, for each panel where they are relevant:
- a specification of the experimental system investigated (eg cell line, species name).
- the assay(s) and method(s) used to carry out the reported observations and measurements.
- an explicit mention of the biological and chemical entity(ies) that are being measured.
- an explicit mention of the biological and chemical entity(ies) that are altered/varied/perturbed in a controlled manner.
- the exact sample size (n) for each experimental group/condition, given as a number, not a range;
- a description of the sample collection allowing the reader to understand whether the samples represent technical or biological replicates (including how many animals, litters, cultures, etc.).
- a statement of how many times the experiment shown was independently replicated in the laboratory.
- definitions of statistical methods and measures:
  - common tests, such as t-test (please specify whether paired vs. unpaired), simple χ2 tests, Wilcoxon and Mann-Whitney tests, can be unambiguously identified by name only, but more complex techniques should be described in the methods section;
  - are tests one-sided or two-sided?
  - are there adjustments for multiple comparisons?
  - exact statistical test results, e.g., P values = x but not P values < x;
  - definition of 'center values' as median or average;
  - definition of error bars as s.d. or s.e.m.

Please complete ALL of the questions below.
Select "Not Applicable" only when the requested information is not relevant for your study.

Materials

Newly Created Materials	Information included in the manuscript?	In which section is the information available? (Reagents and Tools Table, Materials and Methods, Figures, Data Availability Section)
New materials and reagents need to be available; do any restrictions apply?	Not Applicable	

Antibodies	Information included in the manuscript?	In which section is the information available? (Reagents and Tools Table, Materials and Methods, Figures, Data Availability Section)
For **antibodies** provide the following information:<br>- Commercial antibodies: RRID (if possible) or supplier name, catalogue number and or/clone number<br>- Non-commercial: RRID or citation	Yes	Materials and Methods

DNA and RNA sequences	Information included in the manuscript?	In which section is the information available? (Reagents and Tools Table, Materials and Methods, Figures, Data Availability Section)
**Short novel DNA or RNA including primers, probes**: provide the sequences.	Yes	Materials and Methods

Cell materials	Information included in the manuscript?	In which section is the information available? (Reagents and Tools Table, Materials and Methods, Figures, Data Availability Section)
**Cell lines:** Provide species information, strain. Provide accession number in repository **OR** supplier name, catalog number, clone number, and/**OR** RRID.	Yes	Materials and Methods
**Primary cultures:** Provide species, strain, sex of origin, genetic modification status.	Yes	Materials and Methods
Report if the cell lines were recently **authenticated** (e.g., by STR profiling) and tested for mycoplasma contamination.	Yes	Materials and Methods

Experimental animals	Information included in the manuscript?	In which section is the information available? (Reagents and Tools Table, Materials and Methods, Figures, Data Availability Section)
**Laboratory animals or Model organisms:** Provide species, strain, sex, age, genetic modification status. Provide accession number in repository **OR** supplier name, catalog number, clone number, **OR** RRID.	Not Applicable	
**Animal observed in or captured from the field:** Provide species, sex, and age where possible**.**	Not Applicable	
Please detail **housing and husbandry conditions**.	Not Applicable	

Plants and microbes	Information included in the manuscript?	In which section is the information available? (Reagents and Tools Table, Materials and Methods, Figures, Data Availability Section)
**Plants:** provide species and strain, ecotype and cultivar where relevant, unique accession number if available, and source (including location for collected wild specimens).	Not Applicable	
**Microbes:** provide species and strain, unique accession number if available, and source.	Yes	Materials and Methods

Human research participants	Information included in the manuscript?	In which section is the information available? (Reagents and Tools Table, Materials and Methods, Figures, Data Availability Section)
If collected and within the bounds of privacy constraints report on age, sex and gender or ethnicity for all study participants.	Not Applicable	

Core facilities	Information included in the manuscript?	In which section is the information available? (Reagents and Tools Table, Materials and Methods, Figures, Data Availability Section)
If your work benefited from core facilities, was their service mentioned in the acknowledgments section?	Not Applicable	

Design

Study protocol	Information included in the manuscript?	In which section is the information available? (Reagents and Tools Table, Materials and Methods, Figures, Data Availability Section)
If study protocol has been **pre-registered, provide DOI in the manuscript**. For clinical trials, provide the trial registration number **OR** cite DOI.	Not Applicable	
Report the **clinical trial registration number** (at ClinicalTrials.gov or equivalent), where applicable.	Not Applicable	

Laboratory protocol	Information included in the manuscript?	In which section is the information available? (Reagents and Tools Table, Materials and Methods, Figures, Data Availability Section)
Provide DOI OR other citation details if **external detailed step-by-step protocols** are available.	Yes	Materials and Methods

Experimental study design and statistics	Information included in the manuscript?	In which section is the information available? (Reagents and Tools Table, Materials and Methods, Figures, Data Availability Section)
Include a statement about **sample size** estimate even if no statistical methods were used.	Yes	Materials and Methods
Were any steps taken to minimize the effects of subjective bias when allocating animals/samples to treatment (e.g. **randomization procedure**)? If yes, have they been described?	Not Applicable	
Include a statement about **blinding** even if no blinding was done.	Not Applicable	
Describe **inclusion/exclusion criteria** if samples or animals were excluded from the analysis. Were the criteria pre-established?<br><br>If sample or data points were omitted from analysis, report if this was due to attrition or intentional exclusion and provide justification.	Not Applicable	
For every figure, are **statistical tests** justified as appropriate? Do the data meet the assumptions of the tests (e.g., normal distribution)? Describe any methods used to assess it. Is there an estimate of variation within each group of data? Is the variance similar between the groups that are being statistically compared?	Yes	Materials and Methods

Sample definition and in-laboratory replication	Information included in the manuscript?	In which section is the information available? (Reagents and Tools Table, Materials and Methods, Figures, Data Availability Section)
In the figure legends: state number of times the experiment was **replicated** in laboratory.	Yes	Figure legends
In the figure legends: define whether data describe **technical or biological replicates**.	Yes	Figure legends

Ethics

Ethics	Information included in the manuscript?	In which section is the information available? (Reagents and Tools Table, Materials and Methods, Figures, Data Availability Section)
Studies involving **human participants**: State details of **authority granting ethics approval** (IRB or equivalent committee(s), provide reference number for approval.	Not Applicable	
Studies involving **human participants**: Include a statement confirming that **informed consent** was obtained from all subjects and that the experiments conformed to the principles set out in the WMA Declaration of Helsinki and the Department of Health and Human Services Belmont Report.	Not Applicable	
Studies involving **human participants:** For publication of **patient photos**, include a statement confirming that consent to publish was obtained.	Not Applicable	
Studies involving experimental **animals**: State details of **authority granting ethics approval** (IRB or equivalent committee(s), provide reference number for approval. Include a statement of compliance with ethical regulations.	Not Applicable	
Studies involving **specimen and field samples:** State if relevant **permits** obtained, provide details of authority approving study; if none were required, explain why.	Yes	Materials and methods

Dual Use Research of Concern (DURC)	Information included in the manuscript?	In which section is the information available? (Reagents and Tools Table, Materials and Methods, Figures, Data Availability Section)
Could your study fall under dual use research restrictions? Please check biosecurity documents and list of **select agents and toxins** (CDC): https://www.selectagents.gov/sat/list.htm	Not Applicable	
If you used a select agent, is the security level of the lab appropriate and reported in the manuscript?	Not Applicable	
If a study is subject to dual use research of concern regulations, is the name of the **authority granting approval and reference number** for the regulatory approval provided in the manuscript?	Not Applicable	

Reporting

The MDAR framework recommends adoption of discipline-specific guidelines, established and endorsed through community initiatives. Journals have their own policy about requiring specific guidelines and recommendations to complement MDAR.

Adherence to community standards	Information included in the manuscript?	In which section is the information available? (Reagents and Tools Table, Materials and Methods, Figures, Data Availability Section)
State if relevant guidelines or checklists (e.g., **ICMJE, MIBBI, ARRIVE, PRISMA**) have been followed or provided.	Not Applicable	
For **tumor marker prognostic studies,** we recommend that you follow the **REMARK** reporting guidelines (see link list at top right). See author guidelines, under 'Reporting Guidelines'. Please confirm you have followed these guidelines.	Not Applicable	
For **phase II and III randomized controlled trials**, please refer to the **CONSORT** flow diagram (see link list at top right) and submit the CONSORT checklist (see link list at top right) with your submission. See author guidelines, under 'Reporting Guidelines'. Please confirm you have submitted this list.	Not Applicable	

Data Availability

Data availability	Information included in the manuscript?	In which section is the information available? (Reagents and Tools Table, Materials and Methods, Figures, Data Availability Section)
Have **primary datasets** been deposited according to the journal's guidelines (see 'Data Deposition' section) and the respective accession numbers provided in the Data Availability Section?	Not Applicable	
Were **human clinical and genomic datasets** deposited in a public access-controlled repository in accordance to ethical obligations to the patients and to the applicable consent agreement?	Not Applicable	
Are **computational models** that are central and integral to a study available without restrictions in a machine-readable form? Were the relevant accession numbers or links provided?	Not Applicable	
If publicly available data were reused, provide the respective **data citations in the reference list**.	Not Applicable	
